# Targeting MAPK14 by Lobeline Upregulates Slurp1‐Mediated Inhibition of Alternative Activation of TAM and Retards Colorectal Cancer Growth

**DOI:** 10.1002/advs.202407900

**Published:** 2025-01-22

**Authors:** Mingxia Zhao, Lisha Zhou, Qinchang Zhang, Meijing Wang, Yue Dong, Yue Wang, Ruixue Pei, Enguang He, Yanyan Liang, Yujun Shen, Guoliang Deng, Hongqi Chen, Dongdong Sun, Yuxian Shen, Yang Sun, Haibo Cheng

**Affiliations:** ^1^ School of Basic Medical Sciences Biopharmaceutical Research Institute Anhui Medical University Hefei 230032 China; ^2^ State Key Laboratory of Pharmaceutical Biotechnology Nanjing Drum Tower Hospital the Affiliated Hospital of Nanjing University Medical School School of Life Sciences Nanjing University 163 Xianlin Avenue Nanjing 210023 China; ^3^ Jiangsu Collaborative Innovation Center of Traditional Chinese Medicine in Prevention and Treatment of Tumor The First Clinical College Nanjing University of Chinese Medicine 138 Xianlin Avenue Nanjing 210023 China; ^4^ Department of General Surgery Shanghai Jiao Tong University Affiliated Sixth People's Hospital Shanghai 200233 China; ^5^ Jiangsu Key Laboratory of New Drug Research and Clinical Pharmacy Xuzhou Medical University 209 Tongshan Road Xuzhou 221004 China

**Keywords:** colorectal cancer, lobeline, MAPK14, Slurp1, TAMs polarization

## Abstract

Colorectal cancer (CRC) usually creates an immunosuppressive microenvironment, thereby hindering immunotherapy response. Effective treatment options remain elusive. Using scRNA‐seq analysis in a tumor‐bearing murine model, it is found that lobeline, an alkaloid from the herbal medicine lobelia, promotes polarization of tumor‐associated macrophages (TAMs) toward M1‐like TAMs while inhibiting their polarization toward M2‐like TAMs. Additionally, lobeline upregulates mRNA expression of secreted Ly‐6/UPAR‐related protein 1 (*Slurp1*) in cancer cells. The inhibitory effects of lobeline on tumor load and TAM polarization are almost completely eliminated when Slurp1‐deficient MC38 cells are subcutaneously injected into mice, suggesting that lobeline exerts an antitumor effect in a Slurp1‐dependent manner. Furthermore, using target‐responsive accessibility profiling, MAPK14 is identified as the direct target protein of lobeline. Mechanistically, upon binding to MAPK14 in colon cancer cells, lobeline prevents nuclear translocation of MAPK14, resulting in decreased levels of phosphorylated p53. Consequently, negative transcriptional regulation of SLURP1 by p53 is suppressed, leading to enhanced transcription and secretion of SLURP1. Finally, combination therapy using lobeline and anti‐PD1 exhibits stronger antitumor effects. Taken together, these findings suggest that remodeling the immunosuppressive microenvironment using small‐molecule lobeline may represent a promising therapeutic strategy for CRC.

## Graphical abstract

This study provides a detailed description of the mechanism by which lobeline inhibits CRC; that is, it demonstrates that lobeline regulates TAM polarization through the MAPK14/p53/*Slurp1* signaling pathway. Moreover, the combination of lobeline and anti‐PD1 has a stronger antitumor effect in CRC. Lobeline‐mediated antitumor immune activation may be a promising therapeutic strategy for CRC.

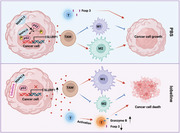

## 1 Introduction

Colorectal cancer (CRC), a malignant epithelial tumor, is the third most prevalent type of cancer and the second leading cause of cancer‐related mortality globally.^[^ ^1^ ^]^ With the emergence of more effective therapeutic approaches, particularly immunotherapies, there has been significant improvement in patient prognosis over the past two decades.^[^ ^2^ ^]^ Cancer immunotherapy has revolutionized cancer treatment by utilizing checkpoint inhibitors and adoptive cell therapy to manipulate the immune system's ability to recognize and attack cancer cells. However, despite the clinical success of immunotherapies, such as chimeric antigen receptor T‐cell therapy and the use of antibodies to target cytotoxic T lymphocyte antigen‐4, programmed cell death protein 1 (PD‐1), and its ligand PD‐L1, only a subset of patients have exhibited complete response to these treatments, currently immunosuppressive cells or proteins contribute to this limited efficacy.^[^ ^3^ ^]^ As a predominant cause of immune suppression in the tumor microenvironment (TM), M2‐like TAMs express PD1, PD‐L1, B7‐H4, and TIM3 and secrete TGF‐β, VEGF‐α, and IL‐10, which inhibit tumor immunity and promote tumor development and migration.^[^ ^4^ ^]^ In contrast, M1‐like TAMs secrete IL‐1β, TNF‐α, CXCL19, and other cytokines that enhance antitumor immunity.^[^ ^5^ ^]^ This plasticity of TAMs has emerged as a potential target for cancer treatment.

Lobelia is commonly used in CRC treatment, but its active component and the details of its mode of action are still not fully understood. Lobeline, the primary piperidine alkaloid of lobelia, is a natural product that exhibits various pharmacological properties, including its therapeutic effects for smoking cessation,^[^ ^6^ ^]^ anxiety and depression in mice,^[^ ^7^ ^]^ and alcohol abuse.^[^ ^8^ ^]^ In addition, lobeline has the potential to inhibit CRC, which is indicated by its ability to reverse P‐gp‐dependent multidrug resistance in Caco‐2 cells.^[^ ^9^ ^]^ However, the biological function and molecular mechanisms of lobeline in TME have not been elucidated.

Mitogen‐activated protein kinase 14 (MAPK14) is probably the most characteristic isoform of the p38MAPK family member. Activation of MAPK14 is induced by most stress stimuli, including oxidative stress, and heat or osmotic shock, but it is also induced by the exposure of cells to cytokines, chemokines, or growth factors. Once MAPK14 becomes activated, it can translocate into the nucleus and phosphorylate many substrates on Ser or Thr residues, such as p53, Elk, Creb1, etc, leading to the regulation of gene transcription.^[^ ^10^ ^]^ The function of MAPK14 in CRC is controversial.^[^ ^11^ ^]^ Mice with MAPK14‐deficient intestinal epithelial cells are more susceptible to colitis‐associated colon tumorigenesis. In contrast, pharmacological inhibition using PH797804 or knockdown of MAPK14 in colon tumor cells reduces tumor burden in mice.^[^ ^11^ ^]^ Recent research has demonstrated that MAPK14 is a prognostic biomarker associated with the level of immune infiltration of immune cells, including CD8^+^ T cells, CD4^+^ T cells, dendritic cells (DCs), TAMs, etc.^[^ ^12^ ^]^ In addition, inhibiting p38 MAPK activity with regorafenib represses M2 polarization of macrophages, which suppresses MAPK14‐regulated Creb1 phosphorylation to downregulate Klf4 transcription.^[^ ^13^ ^]^

SLURP1 belongs to the LY6/PLAUR protein family and serves as an autocrine or paracrine factor that regulates the growth, differentiation, and migration of keratinocytes.^[^ ^14^ ^]^ SLURP1 can inhibit the proliferation of colon cancer, breast cancer, skin cancer, and other tumor cell lines.^[^ ^15^ ^]^ Recent research has found that recombinant SLURP1 protein can activate the calcium signaling pathway in T cells,^[^ ^16^ ^]^ suggesting that SLURP1 may play a role in regulating immune cell function.

In this study, we investigated the antitumor efficacy and immunomodulatory effects of lobeline in CRC mice models. Specifically, lobeline regulates the polarization of TAMs toward M1‐like TAMs and inhibits their polarization toward M2‐like TAMs. Further experiments revealed that lobeline targets MAPK14 and regulates TAMs polarization through the MAPK14/p53/*Slurp1* signaling pathway. Lobeline‐mediated antitumor immune activation might be a promising therapeutic strategy for colorectal cancer.

## 2 Results

### 2.1 Lobeline Reduces Tumor Load in MC38 Xenograft C57BL/6 Mice and CRC Organoids

**Figure 1:**
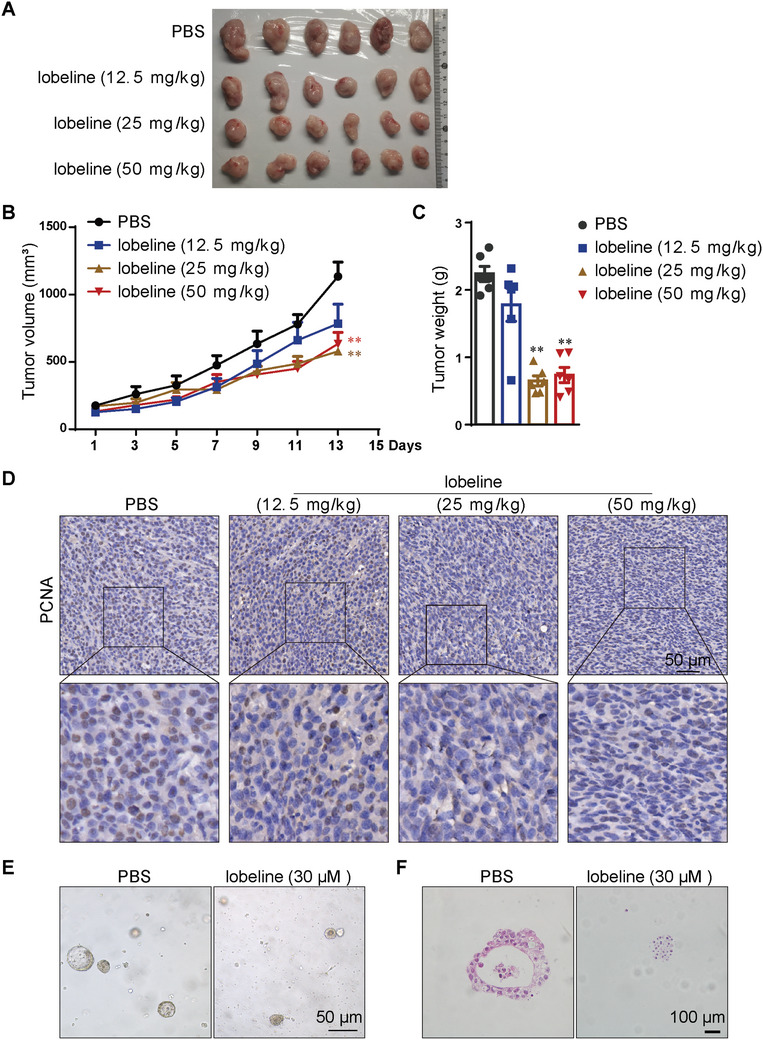
Lobeline reduces tumor load in MC38 xenograft C57BL/6 mice and colorectal cancer organoids. C57BL/6 mice were subcutaneously injected with 1 × 10^6^ MC38 CRC cells, and treated with different doses of lobeline (12.5, 25, and 50 mg kg^−1^, i.p. every day) when the tumor grew to 100–150 mm^3^. A) Representative images. B) Mean tumor volumes of different groups. C) Tumor weight. D) IHC staining of PCNA. Scale bar = 50 µm. After 3 days of subculture, human colon cancer organoids were incubated with lobeline (30 µmol L^−1^) for 48 h, following which images were captured. *N* = 6 mice each group in (A–D). E) Organoid volume, scale bar = 50 µm. F) HE staining of organoid sections. scale bar = 100 µm. Data are presented as mean ± SEM. *p*‐values are determined by a two‐tailed Student's *t*‐test. ^*^ *p* < 0.05, ^**^ *p* < 0.01 versus PBS.

Although lobelia is an anti‐colorectal‐cancer medicine commonly used in clinical practice, its mechanism of action remains unclear. Moreover, previous research has not reported the in vivo role and mechanism of lobeline^___^one of the primary active alkaloids found in lobelia^___^in colon cancer. To address these gaps in the research, we treated C57BL/6 mice with different doses of lobelia after transplanting them with MC38 cancer cells, using 5‐Fu as a positive control. The results showed that lobelia significantly inhibited tumor volume and reduced tumor weight (Figure S1, Supporting Information). Subsequently, we investigated the impact of lobeline on the growth of MC38 xenografts in mice. We observed that lobeline suppressed tumor growth in a dose‐dependent manner, with optimal efficacy at concentrations of 25 mg kg^−1^ (**Figure** **1**A–C). The PCNA protein level decreased with increased lobeline concentration in mice tumors (Figure 1D), indicating that lobeline inhibited the proliferation of colon cancer cells in a dose‐dependent manner. We observed similar antitumor effects of lobeline in the CT26 xenograft model (Figure S2, Supporting Information). Furthermore, we examined the pharmacological effects of lobeline on human CRC organoids and found that it effectively impeded their growth (Figure 1E,F). Collectively, these findings indicate that lobeline effectively suppresses CRC proliferation.

### 2.2 ScRNA‐seq Analysis Reveals that Lobeline Retards the Growth of Transplanted CRC in Mice

**Figure 2:**
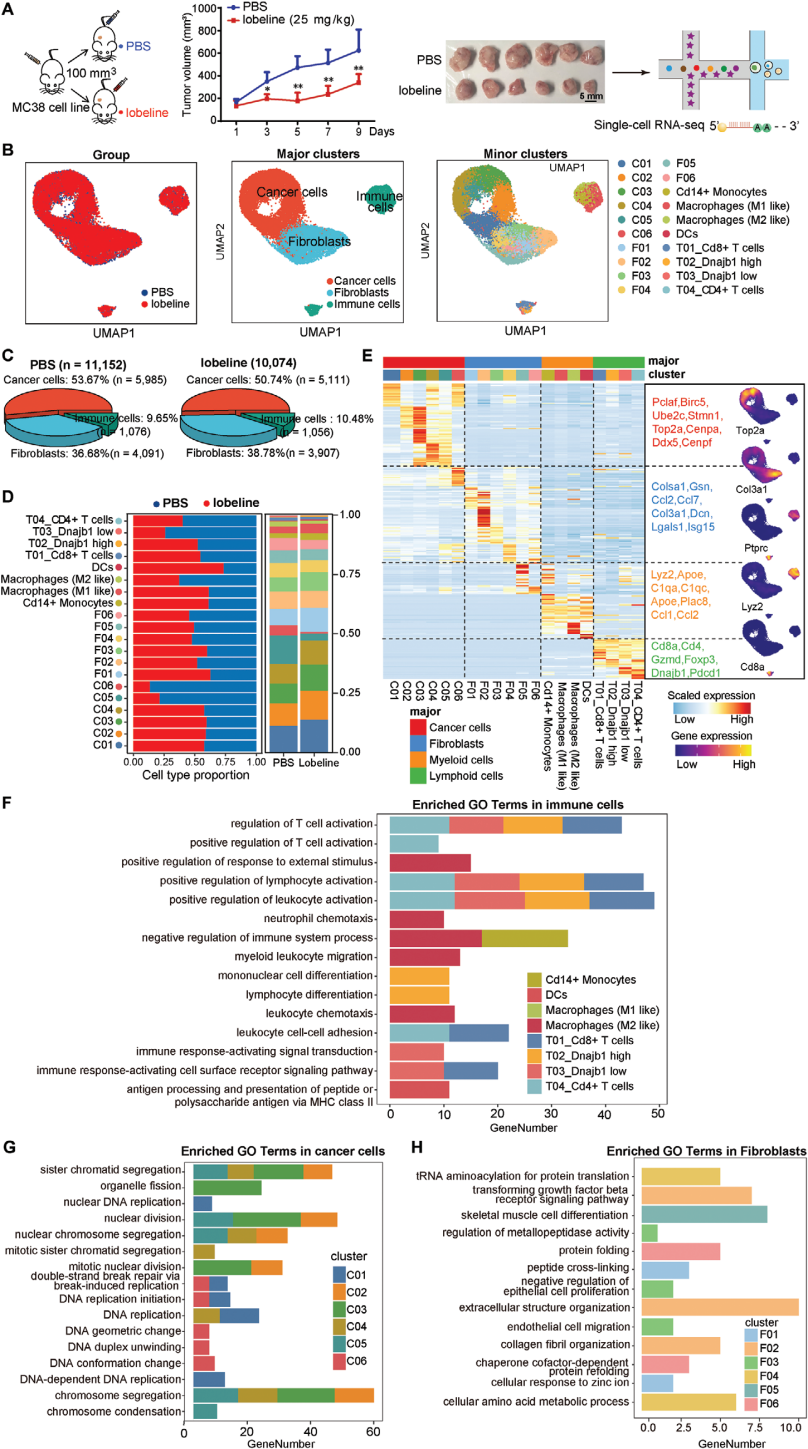
ScRNA‐seq analysis revealed that lobeline retarding the growth of transplanted CRC in mice. A) Schematic diagram of the scRNA‐seq experiment. The mice were subcutaneously injected with 1 × 10^6^ MC38 cancer cells, and treated with PBS or lobeline (25 mg kg^−1^ i.p. every day) when the tumors grew between 100–150 mm^3^. Nine days later, the tumors were removed, and then a single‐cell suspension was prepared for testing. Scale bar=5 mm. *N* =6 mice. B) UMAP (uniform manifold approximation and projection) for dimension reduction of 21226 cells clustered into 3 major clusters (middle) and 20 minor clusters (right) in two groups (left). C) 3D pie chart of the major cluster proportion in PBS and lobeline. D) Bar plots showing the percentage (%) of cell types in PBS and lobeline. E) Heatmap showing the classification of cell subsets and selected marker genes. Red: high expression; blue: low expression. F–H) GO analysis for immune cells, cancer cells, and fibroblasts. Data are represented as mean ± SEM. P‐values are determined by a two‐tailed Student's *t*‐test, ^*^ *p* < 0.05. ^**^ *p* < 0.01.

To further elucidate the mechanism by which lobeline inhibits tumor load, we collected xenograft tumors from PBS and lobeline (25 mg kg^−1^) treatment groups for single‐cell sequencing (10×Genomics) (**Figure** **2**A). After quality control and filtering, 11152 and 10074 transcriptomes of single cells for the PBS and lobeline treatments, respectively, were obtained for downstream analysis. Three major cell types^___^cancer cells, fibroblasts, and immune cells^___^were defined according to their gene‐expression signatures (Figure 2B). We further divided cancer cells into six subsets (Figure S3, Supporting Information), fibroblasts into six subsets (Figure S4, Supporting Information), and immune cells into monocytes (Cd14^+^ monocytes, M1 macrophages, M2 macrophages, and DCs) and T cells (CD8^+^ T cells, CD4^+^ T cells, *Dnajb1* high cells, and *Dnajb1* low cells) (Figure S5, Supporting Information). We compared the changes in the number of various cell types in the PBS and lobeline treatment groups. In addition to a decrease in the percentage of cancer cells (from 53.67% to 50.74%), the percentage of immune cells (from 9.65% to 10.48%) increased in the lobeline‐treated group (Figure 2C). The proportions of antitumor immune cells (CD14^+^ monocytes, M1 macrophages, DCs, and CD8^+^ T cells) increased, but the tumor‐associated immune cells (M2‐like macrophages) decreased (Figure 2D). Unsupervised clustering with UMAP annotated 20 sub‐cell clusters with known or putative markers (Figure 2E). The cell clusters of C01‐C06 were classified as clusters of cancer cells, due to their functional heterogeneity and expression of the proliferation markers. The clusters F01‐F06 were identified as clusters of fibroblasts based on the known markers *Col3a1*, *Gsn*, and *Colsa1* and the extracellular matrix gene *Dcn* (i.e., *decorin*). Because the remaining cluster was Ptprc positive (Cd45^+^), it was identified as a cluster of immune cells. The immune cells included myeloid cells and T cells, which were identified with the known marker Lyz2 (for myeloid cells) and the known markers Cd8a and Cd4 (for T cells). Table S1 (Supporting Information) shows each characterized by specific marker genes. Our scRNA‐seq data revealed that these cell clusters corresponded to major known cell types, providing a valuable resource for research on the molecular mechanisms underlying lobeline's role in TME. As the pathway‐enrichment results shown in Figure 2F indicated activated T‐cell function, which corresponded to an increased number of CD8^+^ T cells. Additionally, Figure 2G shows, our enrichment analysis demonstrated a complex interaction between subpopulations of cancer cells involved in DNA‐replication processes and tumors. This suggests the presence of instability and genetic variation in tumor cells, which promote tumor occurrence and development. We performed GO analysis for each subgroup using 50 differential genes to describe their phenotypes and functions (Table S2, Supporting Information). As the pathway‐enrichment results are shown in Figure 2H, we found that the fibroblast subpopulation was enriched in collagen‐related pathways. By synthesizing ECM components and secreting matrix metalloproteinases (MMPs), fibroblasts can alter the physical and chemical properties of the tumor microenvironment while supporting the invasive activity and metastasis of tumor cells. These data indicated differences between the landscape of PBS and that of lobeline in cancer cells, fibroblasts, and immune cells, suggesting that lobeline may have effects on TME. Taken together, our results reveal that lobeline treatment significantly alters the TME in the transplanted subcutaneous tumor.

### 2.3 Lobeline Promotes SLURP1 Transcription and Translation

**Figure 3:**
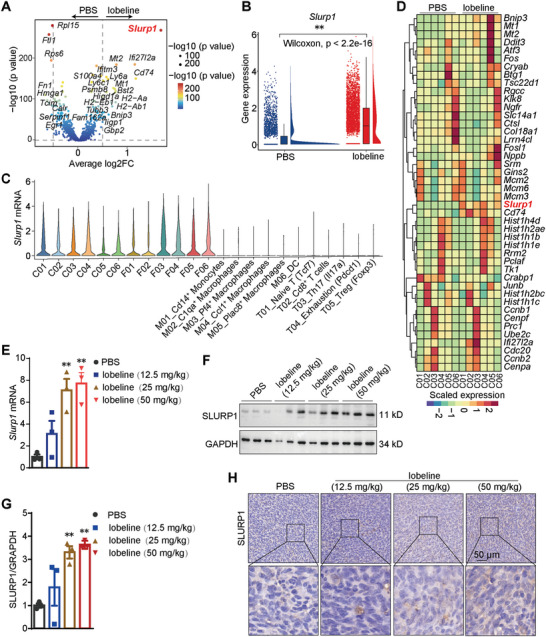
SLURP1 transcription and translation are promoted by lobeline. A) Volcano plot showing transcriptome dynamics between PBS and lobeline. Significantly differentially expressed genes are colored according to experimental groups. B) Raincloud plots show expression differences for *Slurp1* between PBS and lobeline. C) Violin plots show the mRNA of *Slurp1* in minor clusters. D) Heatmap showing the different genes in cancer cell subclusters between PBS and lobeline. Mice were subcutaneously injected with 1 × 10^6^ MC38 cancer cells and subcutaneously injected with PBS or different doses of lobeline (12.5, 25, and 50 mg kg^−1^). E) qPCR for *Slurp1* expression in tumors. F,G) Protein levels of SLURP1 were analyzed by Western blot. GAPDH was shown as the loading control. H) The expression of SLURP1 in tumor tissues was detected by IHC staining. *N* = 6 mice each group in (E–H). Scale bar = 50 µm. *p*‐values are determined by a two‐tailed Student's *t*‐test, ^**^ *p* < 0.01.

To further investigate the mechanism of lobeline‐mediated inhibition of tumor growth, we analyzed differentially expressed genes using single‐cell transcriptome sequencing data. Our findings demonstrated a significant upregulation of *Slurp1* in the tumors from mice treated with lobeline compared to those from the PBS‐treated group (**Figure** **3**A,B). Subgroup analysis revealed that *Slurp1* exhibited its highest expression levels in the cancer cell subsets and its second highest in the fibroblast subsets and that its expression was minimal in the other cell types (Figure 3C). Moreover, gene‐heatmap analysis indicated a higher expression of Slurp1 in all six cancer cell subsets in mice treated with lobeline than in those subsets in mice treated with PBS (Figure 3D). Consistent with these observations, Slurp1 mRNA levels exhibited a substantial elevation in tumors from the lobeline‐treated group (Figure 3E). The protein level of SLURP1 correspondingly increased in the tumors of mice treated with lobeline (Figure 3F–H). Collectively, our findings provide compelling evidence that treatment with lobeline results in a significant enhancement of both the mRNA and protein levels of SLURP1 in cancer cells.

### 2.4 *Slurp1* Knockout Reverses the Amelioration of Lobeline on Tumor Load

**Figure 4:**
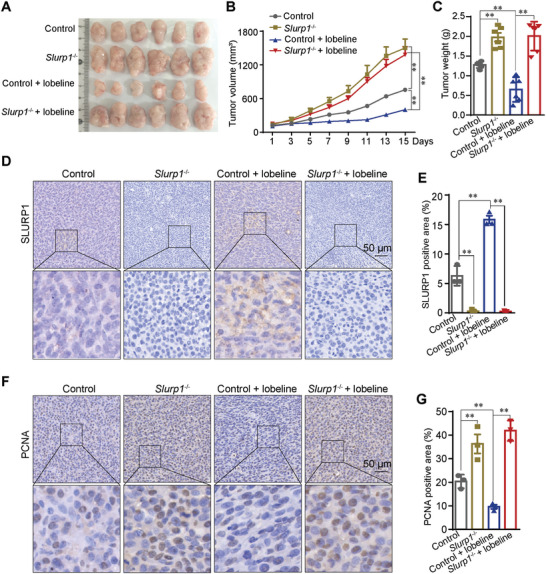
*Slurp1* knockout reverses the amelioration of lobeline on tumor load. Wild‐type mice were injected with *Slurp1* knockout MC38 cells or control cells subcutaneously (1 × 10^6^), and treated with lobeline (25 mg kg^−1^, i.p. every day) when the tumor grew to 100–150 mm^3^. A) Tumor photos. B) Tumor growth curve. C) Tumor weight. *N* = 6 mice in each group in (A–C). D–G) IHC staining of SLURP1 and PCNA. *N* = 3 in (D–G). Scale bar = 50 µm. *n* = 6 per group. *p*‐values are determined by two‐way ANOVA with Student׳s *t*‐test, ^**^ *p* < 0.01.

The upregulation of Slurp1 in lobeline‐treated mice suggests its potential involvement in regulating the antitumor effect. Therefore, we investigated whether the knockdown of Slurp1 could reverse the antitumor effect of lobeline. We obtained MC38 cell lines with a genomic knockout of *Slurp1* (*Slurp1* ^−/−^) using CRISPR/Cas9, as depicted in Figure S6A (Supporting Information). The efficiency of SLURP1 knockout was confirmed by Western blot analysis (Figure S6B, Supporting Information). C57BL/6 mice were subcutaneously injected with 1 × 10^6^ *Slurp1* ^−/‐^ or control MC38 cells, and treated with lobeline (25 mg kg^−1^, i.p. every day) when the tumor reached a size of 100–150 mm^3^. The results showed that the tumor volume and weight were significantly higher in mice subcutaneously injected with *Slurp1* ^−/−^ MC38 cells than in those injected with control MC38 cells. However, although treatment with lobeline led to a significant reduction in tumor volume and weight, this inhibitory effect was abolished in mice subcutaneously injected with *Slurp1* ^−/−^ MC38 cells (**Figure** **4**A–C). In contrast to the control group, in the *Slurp1* ^−/−^ group, the SLURP1 protein level decreased significantly and the PCNA protein level increased significantly (Figure 4D–G). These data suggest that is involved in the antitumor process induced by lobeline and that knockout of *Slurp1* almost completely eliminates this effect.

### 2.5 Colon‐Cancer‐Cell‐Derived SLURP1 is Involved in the Process of Lobeline Regulation of Macrophage Polarization

**Figure 5:**
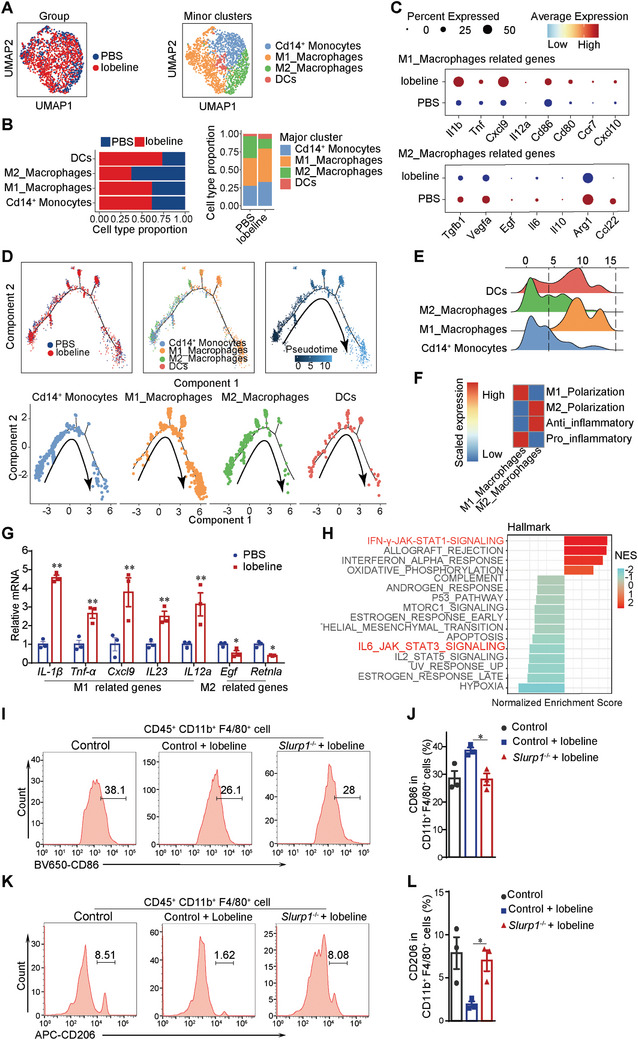
Colon cancer cells‐derived SLURP1 is involved in the process of lobeline regulating macrophage polarization. A) The UMAP projection of myeloid subclusters in PBS and lobeline. B) The percentage (%) of myeloid cell subpopulation in PBS and lobeline. C) Dot plots showing M1 and M2 macrophages‐related genes across the myeloid subtypes. D) Pseudo‐time trajectory analysis of myeloid cells based on groups and subtypes, pseudo‐time (above), and faceted plots showing cell types in pseudo‐time series (under). E) Ridge plots showing the differentiation of monocyte and macrophage subtypes along the pseudo‐time. F) Heat map of macrophage polarization and anti‐inflammatory function. G) qPCR for M1 and M2 macrophages‐related gene expression in tumors. *N* = 3 in (G). H) Representative KEGG pathways enriched in myeloid cells. Pathways associated with macrophage polarization are highlighted in red. I–L) Histograms showing the number of M1 and M2 macrophages detected by flow cytometry in mice tumors. *N* = 3 each group in (I–L). All data are expressed as mean ± SEM from 3 independent experiments. *p*‐values are determined by Student׳s *t*‐test (G) and two‐way ANOVA (I‐L), ^*^ *p* < 0.05, ^**^ *p* < 0.01.

As mentioned above, lobeline treatment profoundly affected the proportion of myeloid cells in the tumor (Figure 2D,E). Therefore, we further divided myeloid cells into four subclusters based on gene expression, including CD14^+^ monocytes, M1‐like macrophages, M2‐like macrophages, and DCs (**Figure** **5**A). In contrast to the PBS group, in the lobeline group, the number of M1‐like macrophages, CD14^+^ monocytes, and DCs increased significantly and the number of M2‐like macrophages decreased significantly (Figure 5B). We therefore compared the differentially expressed genes of M1‐like and M2‐like macrophages between the PBS and lobeline groups. We found that M1‐like‐macrophage‐related genes, such as *Il1b*, *Tnf*, *Cxcl9*, *Il12a*, *CD86*, *CD80*, *Ccr7* and *Cxcl9* were upregulated in lobeline group. Accordingly, we observed significant downregulation of M2‐like‐macrophage‐related genes, such as *Tgfβ1*, *Vegfα*, *Egf*, *Il6*, *Il10*, *Arg1* and *Ccl22* (Figure 5C). These data suggest that lobeline enhances the function of M1‐like macrophages and inhibits that of M2‐like macrophages. The results of our cell‐trajectory analysis showed that TAMs in the lobeline group tended to polarize toward M1‐like macrophages (Figure 5D–F). We selected tumor tissues of mice for our qPCR experiment, and the results of this experiment were consistent with the data from single‐cell sequencing (Figure 5G). The results of our KEGG analysis, which we performed next, indicated that lobeline significantly activated the IFN‐γ‐JAK‐STAT1 signaling pathway, which promotes the polarization of TAMs toward M1‐like macrophages. Lobeline also inhibited the IL‐6‐JAK‐STAT3 signaling pathway, which promotes the polarization of TAMs toward M2‐like macrophages (Figure 5H). Furthermore, we observed the number of M1‐ and M2‐like macrophages in both the tumors with and those without Slurp1 knockout. Our flow cytometry results showed that in mice treated with lobeline, the number of CD45^+^CD11b^+^F4/80^+^CD86^+^ cells (M1‐like macrophages) increased significantly whereas the number of CD45^+^CD11b^+^F4/80^+^CD206^+^ cells (M2‐like macrophages) decreased significantly. These changes were reversed in the tumor tissues of mice injected with Slurp1^−/−^ MC38 cells (Figure 5I–L). Collectively, our results suggest that colon‐cancer‐cell‐derived SLURP1 is involved in the process of the lobeline's regulation of macrophage polarization.

### 2.6 MAPK14 is the Target Protein of Lobeline

**Figure 6:**
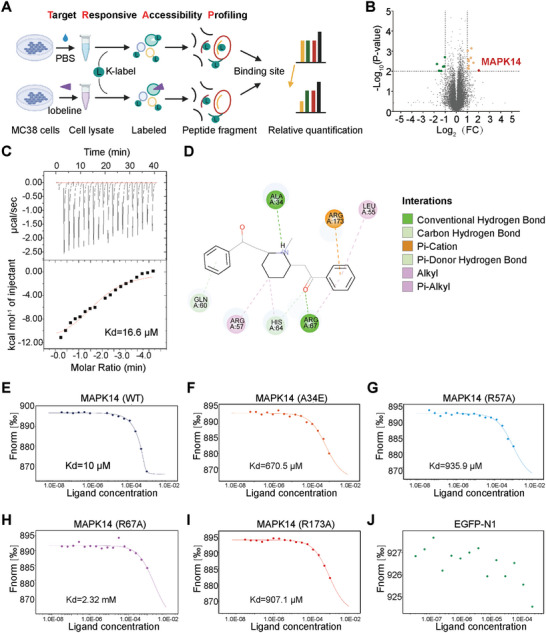
MAPK14 is the target protein of lobeline. A) The Flow chart shows the process of searching for lobeline target protein by TRAP experiment. B) Volcano map showing lobeline binding proteins. FC = Fold change (ratio of lobeline to DMSO). C) Isothermal titration calorimetry (ITC) results of lobeline and MAPK14‐WT protein. D) Docking prediction of the binding sites of lobeline and MAPK14 (PDBID: 5ETI). E–I) 293T cells transfected with MAPK14 (WT) or point mutant plasmids for 48 h and the protein lysate was taken for MST detection. J) EGFP‐N1 was transfected as a control.

To investigate the specific target protein of Lobeline that exhibits antitumor activity, we employed the previously established Target discovery via a target‐responsive accessibility profiling approach (TRAP) approach in our laboratory.^[^ ^17^ ^]^ We incubated MC38 cells with lobeline (30 nmol L^−1^) and the PBS control and then performed cell lysis and treatment according to the TRAP workflow. A total of 16 potential target proteins for lobeline were identified (**Figure** **6**A; Table S3, Supporting Information). Among these, MAPK14 was selected for further study (Figure 6B). Subsequently, we used ITC to confirm the binding affinity between lobeline and MAPK14, it indicated an affinity of 16.6 nmol L^−1^ (Figure 6C). Furthermore, based on the crystal structure of MAPK14 as presented in the PDB library, we predicted potential binding sites between lobeline and MAPK14. The results suggested that the lobeline may interact with specific residues in the domain of MAPK14, including A34, L55, R57, Q60, H64, R67, and R173 (Figure 6D). To validate these putative binding sites between lobeline and MAPK14, we transfected mutant plasmids encoding A34E, R57A, R67A, and R173A variants into MC38 cells. Interestingly, the affinity between these mutant plasmids was nearly a hundred times higher than that observed with wild‐type plasmid (WT) controls (Figure 6E‐J), indicating that the residues A34, R57, R67, and R173 might be crucial for mediating the interaction between MAPK14 and lobeline. VMAT2,^[^ ^18^ ^]^ CHRNA2,^[^ ^19^ ^]^ and CHRNA4^[^ ^20^ ^]^ are three of the previously reported targets of lobeline, however, the expression of VMAT2 was not detected in MC38 cells (data was not shown). To investigate whether these targets are involved in the inhibitory effect of lobeline on SLURP1 expression, we employed small interfering RNA to knock down *Chrna2* and *Chrna4* in order to examine their impact on the lobeline's regulation (Figure S7, Supporting Information). The results showed that neither CHRNA2 nor CHRNA4 plays a role in lobeline‐mediated inhibition of CRC through SLURP1.

### 2.7 MAPK14 Inhibits SLURP1 Transcription and Translation Via p53

**Figure 7:**
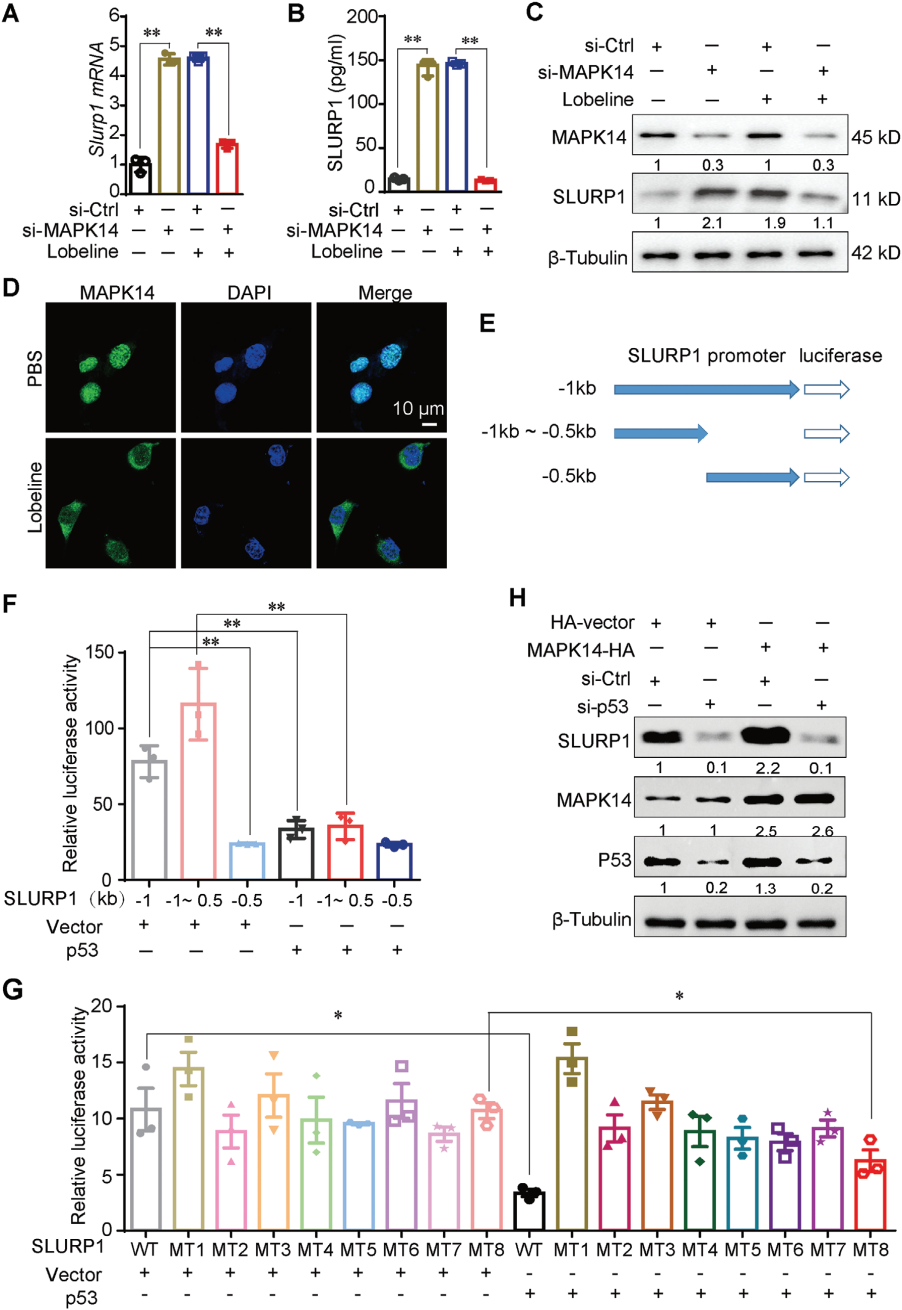
MAPK14 inhibits SLURP1 transcription and translation via p53. MC38 cells were transfected with si‐MAPK14 and then treated with or without 30 nmol L^−1^ lobeline for 48 h. A) *Slurp1* mRNA level was detected by qPCR. B) Cell supernatant was collected for ELISA assay to detect the secretion level of SLURP1.N= 3 in (A,B). C) MAPK14 and SLURP1 protein levels were detected by Western blot. D) After the MC38 cells were treated with lobeline (30 nmol L^−1^) for 48 h, the position relationship between MAPK14 and the nucleus was detected by immunofluorescence staining. E,F) A schematic diagram of human Slurp1 promoter and its truncates cloned to the luciferase reporter. The luciferase reporter was co‐transfected into 293T cells alone or with p53 plasmid, the luciferase activity was measured 36 h after transfection. G) Effect of mutation of p53 binding sites on Slurp1 transcription. The mutants were co‐transfected into 293T cells with FLAG‐p53 plasmid. 36 h after transfection, the luciferase activity was measured. *N* = 3 in (F,G). H) MC38 cells were transfected with MAPK14‐HA plasmid and/or sh‐p53 for 48 h, then the cells were collected for the Western blot experiment. All data are expressed as mean ± SEM. *p*‐values are determined by two‐way ANOVA and Student׳s *t*‐test. ^*^ *p* < 0.05, ^**^ *p* < 0.01.

We have identified MAPK14 as the target protein of lobeline and observed that lobeline enhances the gene transcription of *Slurp1*. MAPK14 is reported to be a transcriptional regulator involved in regulating the expression of multiple genes.^[^ ^10^ ^]^ Thus, we investigated whether MAPK14 is involved in *Slurp1* transcription. Knocking down MAPK14 using small interfering RNA resulted in an increase in the mRNA and protein levels of SLURP1 in MC38 cells, indicating that MAPK14 inhibits SLURP1 transcription and protein secretion. However, knocking down MAPK14 did not affect the mRNA and secreted protein levels of *Slurp1* in the group treated with lobeline, indicating that the lobeline's negative regulation of *Slurp1* transcription is mediated by MAPK14 (**Figure** **7**A–C). Studies have demonstrated that the activation of MAPK14 leads to its nuclear translocation and subsequent regulation of gene transcription through phosphorylated transcription factors.^[^ ^10^ ^]^ After treating MC38 cells with lobeline for 48 h, we observed a significant decrease in nuclear levels of MAPK14 but an increase in its cytoplasmic levels (Figure 7D). Our analysis using the TFBIND database (https://tfbind.hgc.jp/) revealed multiple potential p53 binding sites in the promoter region (−1.0 kb) of SLURP1, suggesting that p53 regulates SLURP1 transcription. To determine the specific position at which MAPK14 regulates SLURP1 transcription, we constructed two truncated SLURP1 promoters with luciferase reporter for a dual luciferase reporter assay. Cotransfection experiments were performed using 293T cells transfected with either full‐length or truncated SLURP1 promoters along with a FLAG‐p53 construct. After 48 h of transfection, the cells were harvested to measure luciferase activity. In contrast to the vector control, p53 significantly suppressed the transcriptional activity in the SLURP1 promoter within the region spanning −1.5 to −0.5 kb, but had no observable in the region at −0.5 kb in the SLURP1 promoter (Figure 7E,F). Subsequently, we generated mutants with deletions of all eight binding sites in this region (Table S4, Supporting Information). Our findings revealed that p53 binding site 8 in the SLURP1 promoter region exhibited stronger transcriptional repression (Figure 7G). Furthermore, the knockdown of p53 using short hairpin RNA demonstrated a reduction in MAPK14‐mediated inhibition of SLURP1 expression (Figure 7H) These results collectively suggest that MAPK14 inhibits both transcription and translation of SLURP1 through its interaction with p53.

### 2.8 The Combination of Lobeline and Anti‐PD1 Antibody Shows a Stronger Antitumor Effect

**Figure 8:**
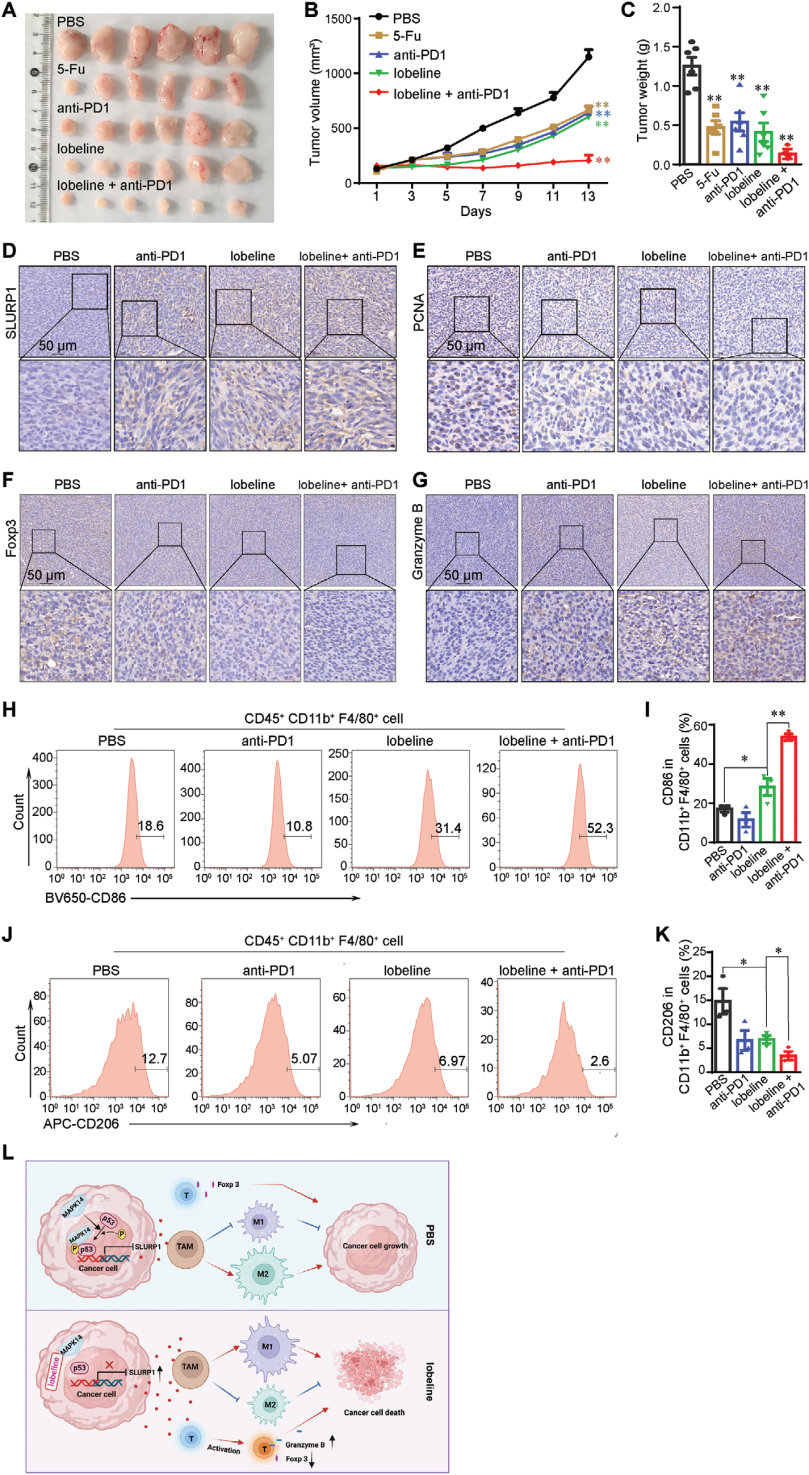
The combination of lobeline and PD1 antibody shows a stronger antitumor effect. C57BL/6 mice were subcutaneously inoculated with 1 × 10^6^ MC38 colon cancer cells, and treated with anti‐PD1 (5  mg kg^−1^ i.p. once every three days) and/or lobeline (25  mg kg^−1^ i.p. every day) when the tumors grew to 100 mm^3^. All results presented are of day 13 post‐inoculation. A) Representative images of tumors. B) Mean tumor volumes of different groups. C) Mean tumor weights of different groups. *N* = 6 mice in each group in (A–C). D–G) IHC for SLURP1, PCNA, Foxp3, and Granzyme B in tumor sections. H–K) Histograms showing the number of M1‐like and M2‐like macrophages detected by flow cytometry in mice tumors. *N* = 3 in (D–K). L) Graphic illustration of the antitumor mechanism of lobeline. In the tumor microenvironment, MAPK14 translocates to the cancer cell nucleus and phosphorylates p53, which activates the negative transcriptional regulation of Slurp1 and inhibits its protein expression and secretion. Low levels of SLURP1 inhibit M1‐like macrophage polarization and promote M2‐like macrophage polarization, which leads to tumor growth. After administration of lobeline, lobeline binds to MAPK14 in cancer cells, inhibiting MAPK14 entry into the nucleus and reducing phosphorylated p53 levels, thereby inhibiting p53's negative transcriptional regulation of *Slurp1* and promoting *Slurp1* transcription and protein secretion. The secreted SLURP1 acts on TAMs, promoting its polarization toward M1‐like macrophages, and inhibiting its polarization toward M2‐like macrophages. SLURP1 activates T cells, down‐regulating Foxp3 and up‐regulating Granzyme B proteins’ level, thereby inhibiting tumor growth. All data are expressed as mean ± SEM from 3 independent experiments. *p*‐values are determined by two‐way ANOVA and Student׳s *t*‐test, ^*^ *p* < 0.05, ^**^ *p* < 0.01.

In view of the regulation of macrophage polarization in monotherapy, we proceeded to investigate the efficacy of lobeline in combination with anti‐PD1 immunotherapy. Mice bearing MC38 tumors (≈100 mm^3^) were randomly divided into five groups (*n* = 6) and treated with PBS, 5‐Fu, lobeline, anti‐PD1, or lobeline + anti‐PD1. Our results demonstrated that mice treated with a combination of lobeline and anti‐PD1 exhibited better antitumor effects than those receiving either lobeline or anti‐PD1 monotherapy (**Figure** **8**A–C). Furthermore, the combined treatment significantly upregulated SLURP1 expression in tumors and suppressed cancer cell proliferation (Figure 8D,E). In tumors from mice treated with lobeline, the protein levels of Foxp3 decreased whereas those of granzyme B increased; this effect was amplified by combining lobeline with PD‐1 blockade (Figure 8F,G). These results indicated that lobeline plays a role in both reducing immunosuppression and enhancing immune response in T cells. Additionally, our results indicated an upregulation of M1 macrophages and a downregulation of M2 macrophages in the combination treatment group (Figure 8H–K). In conclusion, our findings suggested the ability of lobeline to inhibit colon cancer through modulation of macrophage polarization via the MAPK14/p53/*Slurp1* signaling pathway (Figure 8J) and demonstrate that lobeline and PD‐1 blockade synergistically inhibit tumor progression.

## 3 Discussion

This study demonstrated that lobeline reduces the tumor load of CRC and modulates macrophage polarization to enhance antitumor immunity. The MAPK14/p53/*Slurp1* signaling pathway may play a critical role in the lobeline‐induced regulation of TAM polarization. Our study provides a rationale for combining lobeline with anti‐PD1 therapeutic efficacy in clinical settings.

It has been reported that bioactive natural compounds derived from traditional Chinese medicine exhibit significant potential for tumor treatment. For example, curcumin may be effective in the treatment of CRC,^[^ ^21^ ^]^ andrographolide in the treatment of colitis and cancer,^[^ ^22^ ^]^ and cannabidiol and trifolirhizin in the treatment of colon cancer.^[^ ^23^ ^]^ Although lobelia is commonly used in CRC treatment, its active component and the details of its mechanism of action still require further clarification. Lobeline, an active molecule found in lobelia, possesses various functions including emesis induction, respiratory stimulation, smoking cessation support, and reversal of P‐gp‐dependent multidrug resistance in cancer cells,^[^ ^6^, ^7^, ^8^, ^9^ ^]^ indicating its potential as an antitumor agent. However, previous research has not reported the effects of lobeline on tumors in vivo. Our study is the first to demonstrate that lobeline significantly inhibits tumor growth in both MC38 xenograft C57BL/6 mice models and CRC organoids.

Lobeline attenuates LPS‐induced nonspecific pulmonary inflammation in RAW 264.7 cells,^[^ ^24^ ^]^ but its role in macrophage polarization remains unclear. The advancement of scRNA‐seq technology has greatly facilitated the investigation of the TME.^[^ ^25^ ^]^ Utilizing scRNA‐seq, we defined three major cell types based on their gene expression signatures, including cancer cells, fibroblasts, and immune cells. Notably, the lobeline‐treated group exhibited an increased number of immune cells. Moreover, lobeline upregulated both the quantity and antitumor functionality of CD8^+^ T cells as evidenced by elevated expression levels of Granzyme B within tumors. Additionally, lobeline not only enhanced the population of M1‐like tumor‐associated macrophages (TAMs) but also reduced the presence of M2‐like TAMs, indicating that it had a significant impact on TAM polarization in CRC. Furthermore, lobeline decreased the protein level of Foxp3, which indicates that lobeline plays a role in reducing immunosuppression in T cells. Our results indicated that lobeline has immunomodulatory effects in CRC.

As an inflammation modulator, SLURP1 inhibits the TNF‐α‐induced upregulation of inflammatory cytokines by suppressing NF‐κB nuclear translocation.^[^ ^26^ ^]^ Recent research has demonstrated that SLURP1 not only inhibits cell proliferation but also has the potential to arrest the colon cancer cell cycle at the G1/S interface. Moreover, SLURP1 secreted by *Salmonella* resulted in significant tumor regression in a mouse CT26 tumor model, which indicates that SLURP1 might be a potential tumor suppressor in CRC.^[^ ^27^ ^]^ The present study demonstrated that colon‐ cancer‐cell‐derived SLURP1 regulates the polarization state of TAMs, thereby enhancing antitumor response.

MAPK14, a member of the p38MAPK family, plays a role in stress responses and has been implicated in a variety of pathological conditions including inflammation, aberrant apoptosis, and cancer metastasis.^[^ ^28^ ^]^ It has been demonstrated that inhibiting p38MAPK activity with regorafenib represses M2 polarization of macrophages, which in turn suppresses p38MAPK‐regulated Creb1 phosphorylation to downregulate Klf4 transcription. MAPK14 can be activated by phosphorylation and enters the nucleus, where it activates transcription by phosphorylating transcription p53.^[^ ^10c^ ^]^ Here, we identified MAPK14 as the target protein of lobeline, and MAPK14 inhibited the transcription and secretion of SLURP1via p53. When lobeline binds to MAPK14, both the entry of MAPK14 into the nucleus and the transcriptional inhibition of MAPK14 in SLURP1 are reduced, thus promoting the expression of SLURP1.

This study found that lobeline enhances the presence of CD14^+^ monocytes, DCs, and *Dnajb1* high cells in the tumor microenvironment while reducing the number of *Dnajb1* low cells. Some studies have also found that the *Dnajb1* gene may be related to tumor immune tolerance. It may affect the interaction between tumor cells and immune cells, regulating immunosuppression and immune evasion in the tumor microenvironment. The regulatory effects and mechanisms of lobeline on these immune cells need to be investigated further. In addition, *Slurp1* is also expressed in fibroblasts except for cancer cells. Therefore, research should also investigate the role of *Slurp1* in fibroblasts.

In this study, we found that lobeline effectively suppresses the growth of human‐colon‐cancer organoids, indicating its potential for clinical application in treating colon cancer. Mechanistically, the MAPK14/p53/*Slurp1* signaling pathway may play a critical role in lobeline‐induced regulation of TAMs polarization and the subsequent reduction of CRC tumor load, providing a mechanistic rationale for employing lobelia as a therapeutic agent against CRC. Notably, considering the wide expression and tumorigenic function of MAPK14 across a wide variety of cancers, including breast cancer, lung cancer, and colon cancer among others, it suggests that lobeline could potentially target MAPK14 in multiple malignancies beyond CRC. Furthermore, because downstream proteins p53 and SLURP1 are expressed in diverse cell types as well, this mechanism holds promise for broader applications.

This study provides a detailed description of the mechanism by which lobeline inhibits CRC; that is, it demonstrates that lobeline regulates TAM polarization through the MAPK14/p53/*Slurp1* signaling pathway. Moreover, the combination of lobeline and anti‐PD1 has a stronger antitumor effect in CRC. Lobeline‐mediated antitumor immune activation might be a promising therapeutic strategy for CRC.

## 4 Experimental Section

### Reagent

Lobeline hydrochloride was purchased from MedChemExpress (HYB0979; New Jersey, USA). Antibodies of PCNA (Cat^#^ 13110) and MAPK14 (Cat^#^ 8960T) were purchased from Cell Signaling Technology (Danvers, MA, USA). SLURP1 was purchased from ImmunoWay Biotechnology Company (Plano, TX, USA). Granzyme B (Cat^#^ 13588‐1‐AP) was purchased from ProteinTech group (Chicago, IL, USA). Flow cytometry antibodies of CD45‐APC‐cy7, CD11b‐PE, F4/80‐BV421, CD86‐BV650, and CD206‐APC were purchased from BD Pharmingen (San Diego, CA, USA). Purified anti‐mouse PD1 (Cat^#^ BE0146) was purchased from BioXcell (Lebanon, NH, USA). Ad‐*Surp1*‐shRNA and si‐MAPK14 were from Tsingke Biotech (Beijing, China). Matrigel (Cat^#^ 356231) was purchased from Corning (Corning, NY, USA). Organoid growth medium (Cat^#^ KCW‐2) was purchased from KingBiotech (Chongqing, China).

### Mice

Female C57BL/6 or BALB/c mice (6–8 weeks old) were purchased from GemPharmatech Co. Ltd (Nanjing, China). Mice were maintained in a specific‐pathogen‐free (SPF) facility with 12 h light/dark cycles and had ad libitum access to food and water. The animals were treated humanely and all experimental procedures were carried out in accordance with the Guide for the Care and Use of Laboratory Animals, with the approval of the Animal Care and Use Committee of Anhui Medical University (20231043) (Hefei, China).

### Cells

MC38 cells were obtained from the Cell Resource Center of the Institutes of Biomedical Sciences at Fudan University (Shanghai, China). CT26 and 293T cells were obtained from the School of Life Sciences, Nanjing University. *Slurp1* knock‐out MC38 cell was established by Tsingke Biotech (Beijing, China). Cells were maintained in the appropriate culture medium suggested by suppliers.

### Colon Cancer Xenograft Model

MC38 cells or CT26 cells were subcutaneously inoculated into C57BL/6 mice (1 × 10^6^) or BALB/c mice (1 × 10^6^), respectively. From day 3 post‐inoculation, tumor dimensions were measured every other day and the tumor size was calculated as 0.5 × length × width^2^. When the tumor volume reached about 100 mm^3^, the mice were randomly grouped. The mice were euthanized when the tumor size was larger than 20 mm at the longest axis.

### Organoid Culture

The human colorectal cancer organoids were constructed and cultivated by Chongqing KingBiotech. The patient tissue samples, obtained through surgery, were minced into as small fragments as possible using sterile scissors. The tissue pieces were thoroughly mixed with Matrigel (Corning, Cat^#^ 356231) on ice at a ratio of ≈1:4. The processing that followed was carried out in accordance with the published protocols. To summarize, the Matrigel suspension was rapidly injected into a multiwell plate, resulting in the formation of hemispherical droplets. These droplets were subsequently maintained at 37 °C for 15–20 min to achieve solidification. An appropriate amount of KingcultureTM Organoid Growth medium (Cat^#^ KCW‐2) was added to each well, and the culture medium was replaced every 2–4 days. The study was approved by the Research and Ethical Committee of the Affiliated Hospital of Nanjing University of Chinese Medicine and complied with all pertinent ethical regulations (2020‐NL‐094‐02).

### Quantitative Reverse‐Transcriptase Polymerase Chain Reaction (qRT‐PCR)

The levels of M1‐like TMAs related (*Il1b*, *Tnf*, *Cxcl9*, *Il12*a, and *Il23*) and M2‐like TMAs related (*Egf* and *Retntla*) mRNAs were analyzed by qRT‐PCR. Total RNA was isolated using TriZol reagent (Takara), and cDNA was synthesized using the PrimeScript RT reagent kits (Transgene) according to the manufacturers׳ instructions as previously reported. The qRT‐PCR was performed with SYBR Premix Ex TaqTM (Takara) and CFX96 Real‐time system (ThermoFisher). The murine primer sequences used in PCR are listed in Table S5 (Supporting Information).

### Single Cell Dissociation from Mice for scRNA‐seq

Solid tumors from mice were digested with collagenase IV and DNAase (Beyotime) to obtain single‐cell suspensions. Single‐cell suspensions with a concentration of 1000 cells µL^−1^ were loaded on the 10× genomics chromium controller single‐cell instrument following the 10× genomics manufacturer's protocol.

### scRNA‐seq Data Pre‐Processing and Quality Control

CellRanger v4.0.0 pipeline (10× Genomics) was used to process the scRNA‐seq data, which was based on the mouse reference genome GRCm38 (mm10). The Seurat (v4.0.0) package was employed to analyze digital gene expression matrices.^[^ ^29^ ^]^ Cells were filtered by the number of UMIs (<1 00 000 UMIs), the number of genes (<6500 genes), and the percentage of mitochondrial genes (“percent.mt” lower than 10%) before downstream analysis. *SCTransform* was utilized to normalize the data, which included a regression of the percentage of mitochondrial genes.^[^ ^30^ ^]^ Utilizing the *SelectIntegrationFeatures* function, 3000 shared, highly variable genes were identified.^[^ ^31^ ^]^ Integration anchors were identified based on these genes using the *FindIntegrationAnchors* function with an SCT normalization method. The data were then integrated using the *IntegrateData* function. Principal component analysis (PCA) and uniform manifold approximation and projection (UMAP) dimension reduction with the top 30 principal components were performed. A nearest‐neighbor graph using the 30 dimensions of the PCA reduction was calculated using *FindNeighbors*, followed by clustering using *FindClusters* with a resolution of 0.8.

### Identification of Differentially Expressed Genes (DEGs) Among Clusters and Annotating Cell Clusters

The *FindAllMarkers* function was used to identify candidate marker genes for each cell cluster. The Wilcoxon rank‐sum test, as implemented in the *FindAllMarkers* function, was used to identify group‐specific differentially expressed genes (*min.pct = 0.1, logfc_threshold = 0.1, test.use = “wilcox”*) with the Seurat R package. Genes with an average expression difference of more than 0.5 natural log and a *p*‐value of less than 0.05 were selected as marker genes. To assign a cell type to each cell cluster, a combination of differentially expressed genes and known gene signatures was used.

### Pseudo‐Time Lineage Trajectory

Monocle2 R package was used to infer the cell lineage trajectory of cancer cells.^[^ ^32^ ^]^ Monocle ordered cells by learning an explicit principal graph from the single‐cell genomics data with reversed graph embedding, which accurately resolved biological processes.^[^ ^33^ ^]^ The differential *GeneTest* function was used to derive DEG from each cluster. After the cell trajectories were constructed, differentially expressed genes along the pseudo‐time trajectory were detected. All pseudo‐time‐dependent genes were visualized using the *plot_pseudotime_heatmap* function. Lineage trajectory plots and smooth expression curves based on *CellDataSet* were generated using *plot_cell_trajectory* and *plot_genes_in_pseudotime*, respectively.^[^ ^34^ ^]^

### Enrichment Analysis

The R package clusterProfiler (3.11.1) was employed to conduct KEGG pathway and GO annotation analyses, with parameters set to *PValueCutoff = 0.05, PAdjustMethod = BH, QValueCutoff = 0.05, MingsSize = 10, and MaxGSSize = 500*.

### Western Blot

Western blot analysis was performed as previously described.^[^ ^35^ ^]^

### Histology, Immunohistochemistry (IHC), and Immunofluorescence (IF)

Tumor tissues were subjected to H&E staining following standard protocols and analyzed by a pathologist under a light microscope (Olympus). The sections were initially subjected to deparaffinization, rehydration, and subsequent washing in 1% PBS‐Tween for all staining protocols. For IHC, the sections were treated with 2% hydrogen peroxide to inhibit endogenous peroxidases, blocked with 3% goat serum, and incubated with specific primary antibodies for 2 h at room temperature. Subsequently, the sections were incubated with streptavidin‐HRP for 40 min, stained using DAB substrate, and counter‐stained with hematoxylin. For IF analysis, the slides were labeled with fluorescent antibodies and subsequently counter‐stained with DAPI for a duration of 5 min. Finally, images were acquired using a confocal laser‐scanning microscope (Olympus FV1000).

### Target Discovery Via a Target‐Responsive Accessibility Profiling Approach (TRAP)

The screening of lobeline binding proteins was conducted using a TRAP approach as previously reported.^[^ ^24^ ^]^ This method was used to identify the binding proteins for lobeline in a cellular environment by monitoring changes in lysine accessibility on a proteome level induced by ligand engagement. Two dishes of cells were treated with either 30 µmol L^−1^ lobeline or PBS, and then permeabilized using M‐PER buffer (Thermo Scientific). The lysates were subsequently covalently labeled with formaldehyde and borane pyridine complex, and precipitated with organic solvent. The collected pellets were redissolved in 8 mol L^−1^ urea, reduced by dithiothreitol (DTT) at 56 °C for 30 min, followed by alkylation using iodoacetamide 261 (IAA) in the dark for 30 min. The proteome was then diluted with an ammonium bicarbonate solution until reaching a concentration of 1 mol L^−1^ urea, and the collected peptides were desalted using C18 HLB columns (Waters, Milford, MA, USA). An‐SYNAPT G2 Si Q‐TOF system (Waters, Milford, MA, USA) was utilized for quantitative profiling of lysine accessibility changes upon lobeline binding for target discovery. Data analysis was carried out using PEAKS Studio 8.5 (BSI Solutions, Waterloo, Canada). Cys alkylation and methionine oxidation were selected as fixed modifications while TRAP‐induced lysine dimethylation served as a variable modification during the analysis process. The abundance ratio of each TRAP‐labeled peptide was used to determine the degree of variation in accessibility related to ligand binding affinity. A student's *t*‐test was used to assess whether the detected changes in accessibility of labeled peptides were statistically significant. For screening target reactive peptides belonging to lobeline binding proteins, an inter‐group *p* value of *p* < 0.001 and an R‐value of (TRAP ratio>2 or <0.5) were set as cut‐off points.

### Flow Cytometry

The cells digested from tumors were incubated in PBS with the fixable viability dye (65‐0865‐14, eBioscience) before antibody staining. Fc gamma receptors were blocked with anti‐CD16/CD32 antibodies (2.4G2, BD). Then, cells were incubated with a cell surface marker for 30 min on ice. For intracellular staining, fixing and permeabilization were performed using an Intracellular Fixation and Permeabilization Buffer Set according to the manufacturer's instructions (eBioscience). Thereafter, the cells were incubated with CD206. Cells were analyzed with a Celesta (BD Bioscience) flow cytometer and analyzed by FlowJo (BD) software.

### Microscale Thermophoresis (MST)

The equilibrium dissociation constant (Kd) values were measured using the Monolith NT.115 instrument (NanoTemper Technologies). According to the manufacturer's protocol, mutant proteins of MAPK14 were fluorescently labeled. A range of lobeline concentrations, spanning from 5 nmol L^−1^ (1.9 ng in 1 mL) to 1 mmol L^−1^ (373.92 µg in 1 mL) were incubated with purified labeled MAPK14 protein at a concentration of 200 nmol L^−1^ (95.5 ng in 1 mL) for a duration of 10 min in assay buffer containing NaCl (100 mmol L^−1^; 5.85 mg in 1 mL deionized water) and Tris‐HCl (50 mmol L^−1^; 15.8 mg in 1 mL deionized water), pH = 7.5). Samples were loaded into the NanoTemper glass capillaries and microthermophoresis was carried out using 80% light‐emitting diode (LED) power and 80% MST. Using the mass action equation, the Kd value was calculated from repeated readings of three repeated experiments by NanoTemper software.

### Luciferase Assay

Two regions of the SLURP1 promoter (−1 to −0.5 kb and −0.5 to 0 kb) were inserted into the pGL3‐basic vector and confirmed by sequencing. Eight mutants of the SLURP1 promoter were constructed by Tsingke Biotech. Luciferase reporter assay was performed according to the manufacturer's instructions (Promega Corporation, Madison, WI, United States). 1 × 10^5^ HEK293T cells were seeded in the 24‐well plate overnight, and transfected with 0.05 µg of cytomegalovirus (CMV)‐Renilla together with 0.5 µg SLURP1 promoter constructs, with or without FLAG‐P53 by using Lipofectamine 2000 transfection reagent (Thermo Fisher Scientific, United States). After 48 h transfection, the cells were lysed. Firefly and Renilla luciferase activities were measured with the Dual‐Luciferase Reporter System (Promega).

### Statistics

Statistical analysis was performed using GraphPad Prism 8.0. Data are presented as mean ± SEM, and compared using one‐way ANOVA or Student׳s *t*‐test as appropriate. *N* = 6 mice in each group. Dunnett׳s test was used for comparing three or more groups. Statistical significance was set at ^*^ *p* < 0.05; ^**^ *p* < 0.01.

### Data availability

The raw sequence data reported in the present paper have been deposited in the GEO database under accession no. GSE247841. All data supporting the findings of this study are available within the paper or from the corresponding authors upon reasonable request.

## Conflict of Interest

The authors declare no conflict of interest.

## Author contributions

M.Z., L.Z. and Q.Z. contributed equally to this work. Y.S., H.C., Y.S., and D.S. conceived this project and designed the study. M.Z., Q.Z., M.W., Y.D., Y.W., R.P., and E.H. performed the experiments and analyzed the data. L.Z. analyzed the scRNA‐seq data. D.S., G.D., and Y.S. gave methodological support and conceptual advice. Y.S. and M.Z. wrote the manuscript.

## Supporting information



Supporting Information

## Data Availability

The data that support the findings of this study are openly available in gene expression omnibus database at https://www.https://www.ncbi.nlm.nih.gov/geo/, reference number 247841.
