# Supplementary material for: Targeting MAPK14 by Lobeline Upregulates Slurp1‐Mediated Inhibition of Alternative Activation of TAM and Retards Colorectal Cancer Growth
Source: Adv Sci (Weinh). 2025 Jan 22;12(10):2407900. doi: 10.1002/advs.202407900 (PMC11904982; doi:10.1002/advs.202407900)

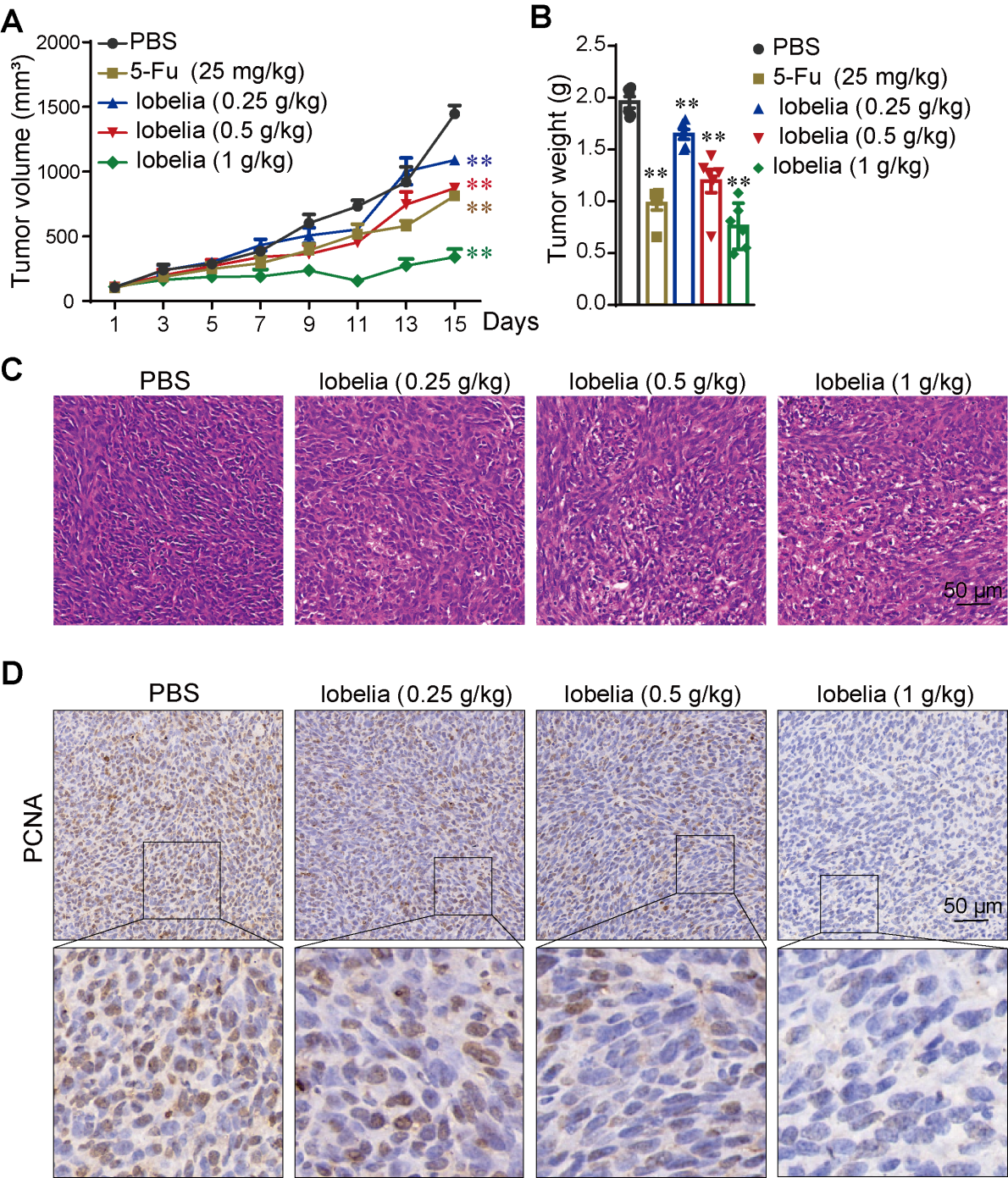


**Figure S1. lobelia inhibits tumor size in mice.**

MC38 cancer cells (1×10^6^) were inoculated subcutaneously into C57BL/6 mice (n = 5-6). When the tumors grew to 100-150 mm^3^, the mice were randomly divided into a PBS group (i.p., once a day), a 5-Fu group (25 mg kg^-1^, i.p., once every other day), or different doses of lobelia groups (0.25 g kg^-1^, 0.5 g kg^-1^ and 1 g kg^-1^, i.p. every day). (A) Tumor growth curve. (B) Tumor weight. N=6 mice in each group in (A-B). (C) HE staining of tumor sections. (D) IHC staining of PCNA. N=3 in (C-D). All data are expressed as mean ± SEM. *P*-values are determined by two-way ANOVA and Student׳s *t*-test, **P*<0.05, ***P*<0.01.


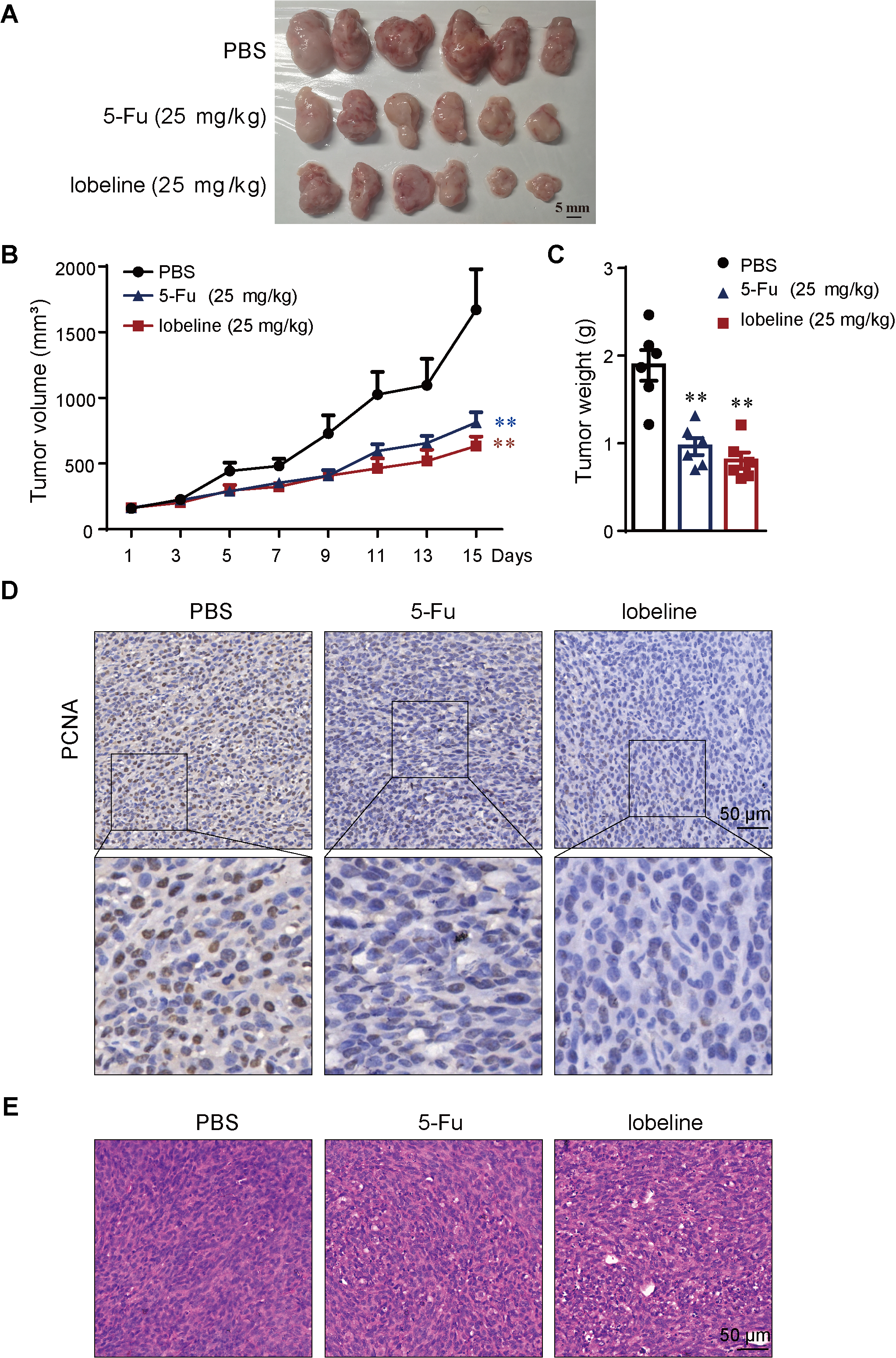


**Figure S2：Lobeline reduces tumor load in CT26 xenograft BALB/c mice.**

CT26 cancer cells (1×10^6^) were inoculated subcutaneously into BALB/c mice (n = 6). When the tumor grew to 100-150 mm^3^, the mice were randomly divided into a PBS group (i.p., once a day), a 5-Fu group (i.p., once every other day), or lobeline group (50 mg kg^-1^, i.p. every day). (A) Tumor photos. Scale bar = 5 cm. (B) Tumor growth curve. (C) Tumor weight. N=6 mice in each group in (A-C). (D) IHC staining of PCNA. (E) HE staining of tumor sections. N=3 in (D-E). All data are expressed as mean ± SEM. *P*-values are determined by two-way ANOVA and Student׳s *t*-test, **P*<0.05, ***P*<0.01.


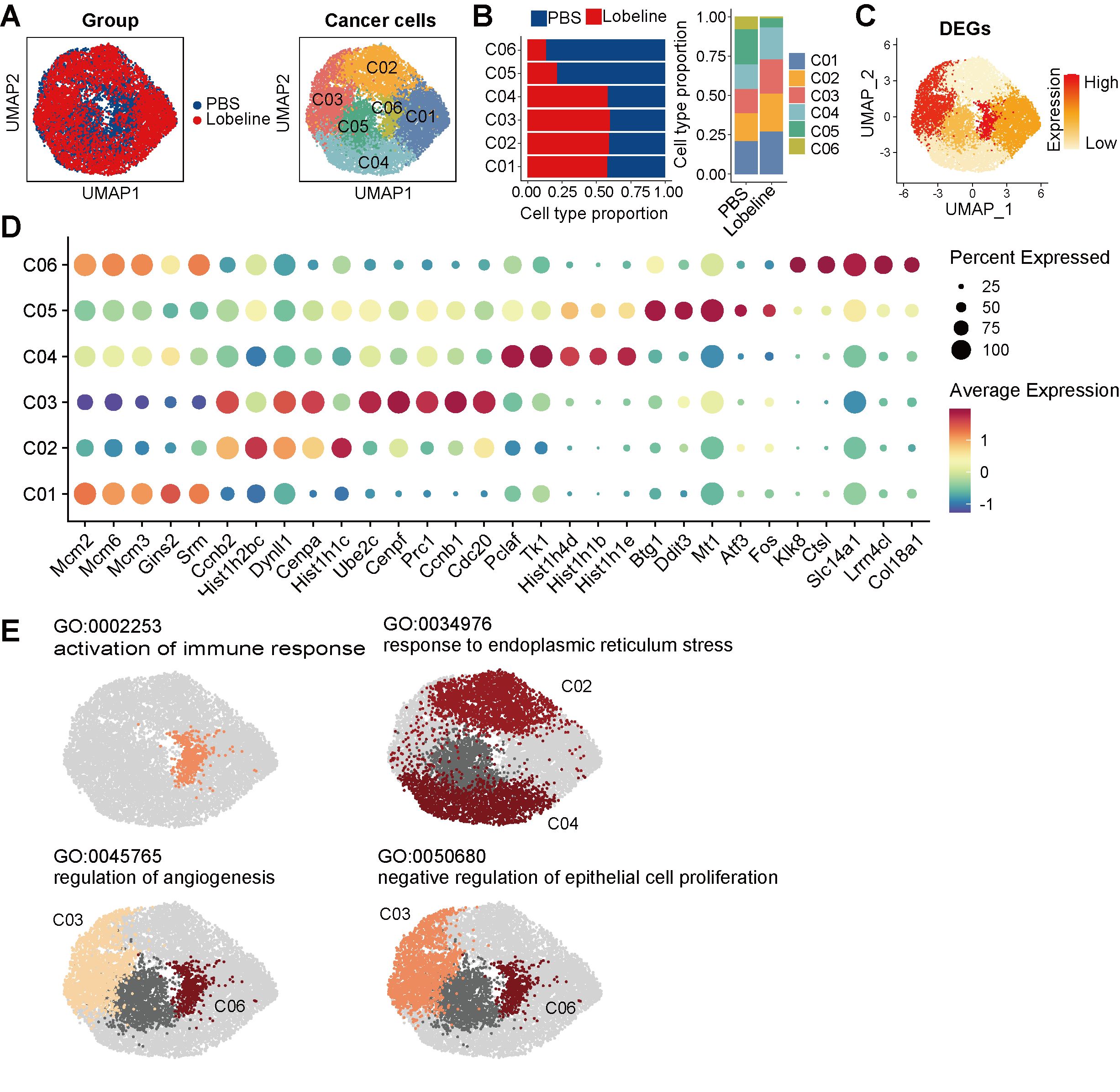


**Figure S3.** **Transcriptome analysis the lobeline’s effect on cancer cells.**

(A) UMAP plot of cancer cell in group and subclusters. (B) Proportion of cancer cell sub-populations between PBS and lobeline. (C) UMAP plots showing differentially expressed genes (DEGs) in cancer cell subclusters. (D) Dot plots showing representative top marker genes across the cancer cell subtypes.


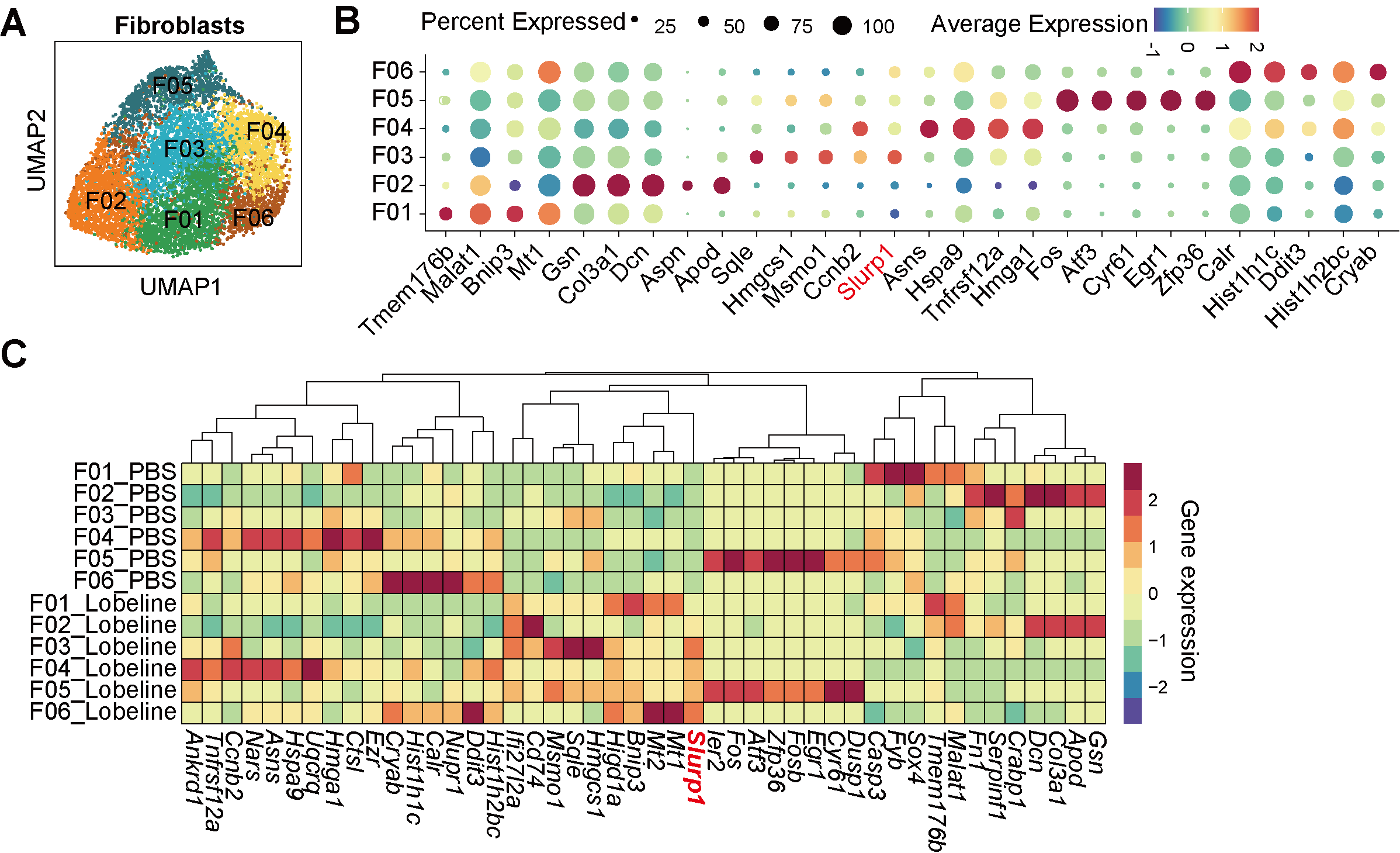


**Figure S4. Transcriptome analysis the lobeline’s effect on fibroblasts.**

1. UMAP plot of fibroblasts in subclusters. (B) Dot plots showing representative top marker genes across the fibroblast subtypes. (C) Heatmap showing the classification of cell subsets and selected marker genes. Red: high expression; blue: low expression.


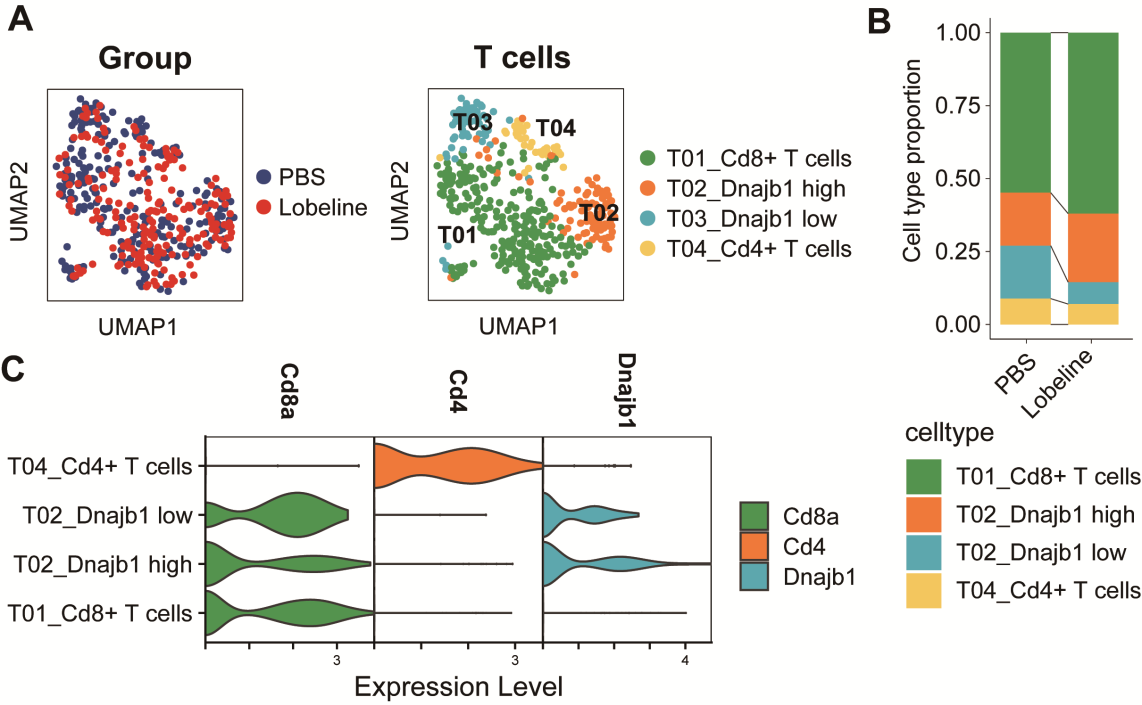


**Figure S5.** **Transcriptome analysis the lobeline’s effect on T cells.**

(A) UMAP plot of T cells in subclusters. (B) Proportion of T cell sub-populations between PBS and lobeline. (C) Violin plots show the mRNA of Cd8a, Cd4 and *Dnajb1* in minor clusters.


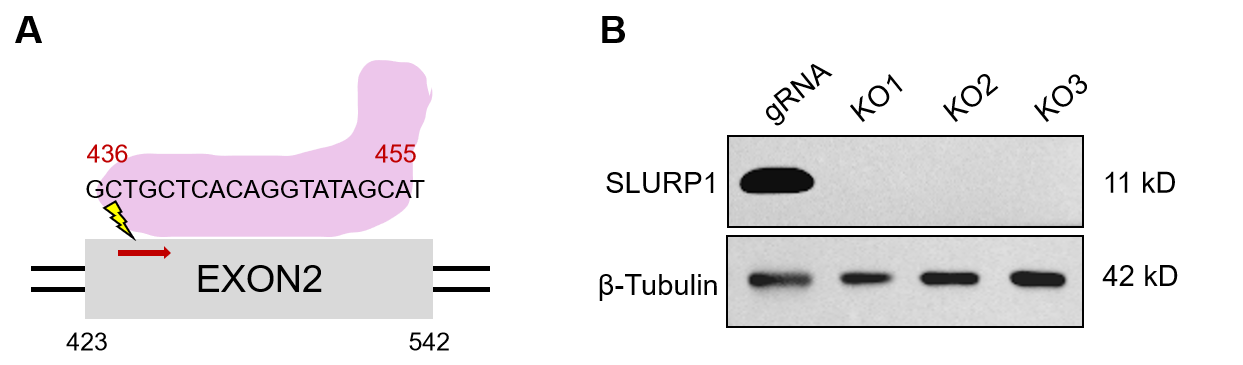


**Figure S6：Construction of *Slurp1* knockout MC38 cells.**

(A) Schematics for the strategy of generating *Slurp1* knockout in MC38 cells. The CRISPR targeting sequence on EXON 2 is shown here. Yellow markers indicate Cas9 cutting positions in the genome. (B) *Slurp1* deletion in MC38 cell clones was confirmed by Western Blot analysis.


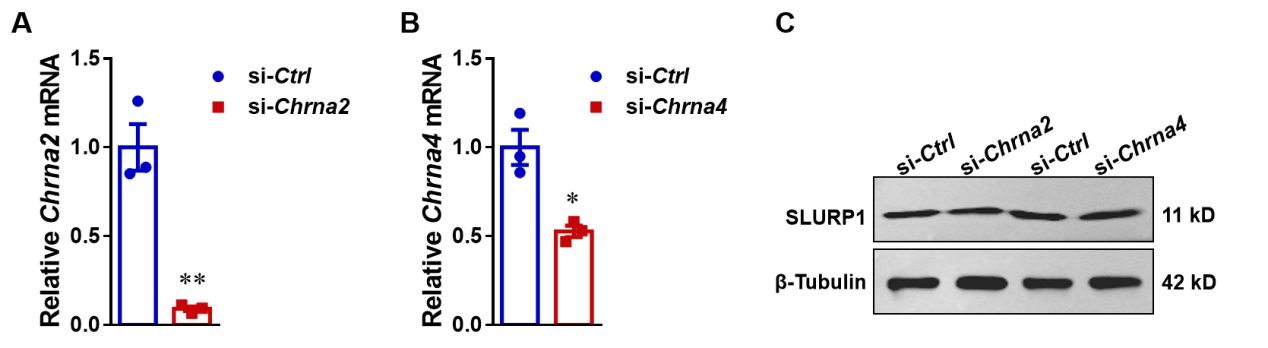


**Figure S7：*Chrna2* and *Chrna4* have no effects on the expression of SLURP1.**

MC38 cells was transfected with si-*Chrna2* or si-*Chrna4* for 48 h, then the cells were collected for the following experiments. (A-B) The si-*Chrna2* and si-*Chrna4* interference efficiency were detected by qPCR. N=3 in (A-B). (C) The SLURP1 protein levels were detected by Western blot. All data are expressed as mean ± SEM. *P*-values are determined by Student׳s *t*-test, **P*<0.05, ***P*<0.01.

**Table S1: Top100 markers of all sunbclusters**

| cluster | gene | p_val | avg_log2FC | pct.1 | pct.2 | p_val_adj |
| --- | --- | --- | --- | --- | --- | --- |
| C01 | Mcm2 | 0 | 1.135609317 | 0.973 | 0.587 | 0 |
| C01 | Mcm6 | 0 | 1.012251013 | 0.982 | 0.67 | 0 |
| C01 | Mcm3 | 0 | 0.996539454 | 0.927 | 0.472 | 0 |
| C01 | Gins2 | 0 | 0.939307423 | 0.848 | 0.383 | 0 |
| C01 | Mcm5 | 0 | 0.910574836 | 0.839 | 0.382 | 0 |
| C01 | Lig1 | 0 | 0.884280566 | 0.894 | 0.478 | 0 |
| C01 | Pcna | 0 | 0.793307723 | 0.903 | 0.545 | 0 |
| C01 | Hells | 0 | 0.790897829 | 0.814 | 0.409 | 0 |
| C01 | Mcm4 | 0 | 0.74428675 | 0.771 | 0.355 | 0 |
| C01 | Mcm7 | 0 | 0.705514218 | 0.802 | 0.418 | 0 |
| C01 | Srm | 0 | 0.690076298 | 0.881 | 0.581 | 0 |
| C01 | Cdca7 | 0 | 0.667569433 | 0.707 | 0.286 | 0 |
| C01 | Ranbp1 | 0 | 0.654453131 | 0.988 | 0.824 | 0 |
| C01 | Dctpp1 | 0 | 0.642747479 | 0.799 | 0.444 | 0 |
| C01 | Nme1 | 0 | 0.633728151 | 0.969 | 0.752 | 0 |
| C01 | Ncl | 0 | 0.612526431 | 1 | 0.971 | 0 |
| C01 | Eif5a | 0 | 0.579830981 | 0.998 | 0.962 | 0 |
| C01 | Cdt1 | 0 | 0.541909239 | 0.612 | 0.253 | 0 |
| C01 | Lgals1 | 0 | 0.524232891 | 1 | 0.997 | 0 |
| C01 | Slc25a5 | 1.21E-305 | 0.611202515 | 0.997 | 0.948 | 2.72E-301 |
| C01 | Rpa2 | 5.10E-298 | 0.561872404 | 0.637 | 0.301 | 1.15E-293 |
| C01 | Slfn9 | 2.41E-293 | 0.556888108 | 0.532 | 0.218 | 5.43E-289 |
| C01 | Dut | 8.63E-278 | 0.57585355 | 0.926 | 0.642 | 1.95E-273 |
| C01 | Rfc2 | 1.71E-277 | 0.534963647 | 0.728 | 0.389 | 3.86E-273 |
| C01 | Tipin | 8.42E-274 | 0.582822601 | 0.819 | 0.499 | 1.90E-269 |
| C01 | Dnmt1 | 1.59E-272 | 0.545506099 | 0.803 | 0.46 | 3.59E-268 |
| C01 | Nasp | 2.04E-270 | 0.58260442 | 0.8 | 0.47 | 4.61E-266 |
| C01 | Ccnd1 | 9.99E-270 | 0.575544585 | 0.987 | 0.836 | 2.25E-265 |
| C01 | Orc6 | 8.83E-267 | 0.56023724 | 0.838 | 0.51 | 1.99E-262 |
| C01 | Siva1 | 1.42E-243 | 0.536069843 | 0.859 | 0.555 | 3.19E-239 |
| C01 | Paics | 1.78E-240 | 0.508680058 | 0.964 | 0.791 | 4.01E-236 |
| C01 | Nhp2 | 2.30E-207 | 0.523333157 | 0.891 | 0.643 | 5.18E-203 |
| C01 | Hmga1 | 6.85E-190 | 0.511871866 | 0.946 | 0.71 | 1.54E-185 |
| C01 | Fkbp11 | 2.55E-185 | 0.509626104 | 0.772 | 0.509 | 5.76E-181 |
| C01 | Il1rl1 | 1.03E-91 | 0.518116443 | 0.48 | 0.294 | 2.33E-87 |
| C02 | Ccnb2 | 0 | 1.597505797 | 0.995 | 0.587 | 0 |
| C02 | Cenpa | 0 | 1.333229217 | 0.955 | 0.413 | 0 |
| C02 | Cdc20 | 0 | 1.179364633 | 0.881 | 0.369 | 0 |
| C02 | Hmgb3 | 0 | 0.942447155 | 0.914 | 0.614 | 0 |
| C02 | Tpx2 | 0 | 0.936395514 | 0.839 | 0.392 | 0 |
| C02 | Racgap1 | 0 | 0.922766598 | 0.918 | 0.505 | 0 |
| C02 | Hmgb2 | 0 | 0.873320434 | 0.988 | 0.824 | 0 |
| C02 | Cenpe | 0 | 0.860742342 | 0.772 | 0.331 | 0 |
| C02 | Dynll1 | 0 | 0.854612445 | 0.979 | 0.842 | 0 |
| C02 | Kif20a | 0 | 0.837717033 | 0.759 | 0.286 | 0 |
| C02 | Cdkn3 | 0 | 0.829374538 | 0.714 | 0.266 | 0 |
| C02 | Mki67 | 0 | 0.803338653 | 0.867 | 0.428 | 0 |
| C02 | Hmmr | 0 | 0.72024543 | 0.674 | 0.275 | 0 |
| C02 | Ptms | 0 | 0.700901038 | 0.987 | 0.911 | 0 |
| C02 | Hmgb1 | 0 | 0.665291633 | 0.999 | 0.937 | 0 |
| C02 | Cenpf | 0 | 0.603549411 | 0.819 | 0.387 | 0 |
| C02 | Hnrnpa2b1 | 0 | 0.599860034 | 1 | 0.976 | 0 |
| C02 | Lmna | 0 | 0.592351823 | 0.998 | 0.964 | 0 |
| C02 | Cdca3 | 8.05E-308 | 0.668243316 | 0.685 | 0.309 | 1.81E-303 |
| C02 | Cdca8 | 4.50E-307 | 0.727424646 | 0.793 | 0.39 | 1.01E-302 |
| C02 | Cep89 | 1.83E-302 | 0.56267009 | 0.497 | 0.179 | 4.13E-298 |
| C02 | Ddx39 | 2.05E-302 | 0.733195775 | 0.931 | 0.696 | 4.62E-298 |
| C02 | Selenoh | 8.66E-299 | 0.7311144 | 0.928 | 0.664 | 1.95E-294 |
| C02 | Hdgf | 2.54E-293 | 0.600690826 | 0.978 | 0.856 | 5.72E-289 |
| C02 | Rad21 | 1.66E-283 | 0.709398896 | 0.918 | 0.668 | 3.74E-279 |
| C02 | Ckap2 | 3.27E-275 | 0.642859302 | 0.665 | 0.319 | 7.38E-271 |
| C02 | Kif23 | 1.23E-258 | 0.607242129 | 0.599 | 0.267 | 2.78E-254 |
| C02 | Bub1b | 6.95E-256 | 0.561680352 | 0.566 | 0.253 | 1.57E-251 |
| C02 | C330027C09Rik | 1.81E-254 | 0.539846641 | 0.488 | 0.198 | 4.07E-250 |
| C02 | Sfpq | 1.56E-253 | 0.65689669 | 0.855 | 0.621 | 3.52E-249 |
| C02 | Nucks1 | 1.81E-242 | 0.611938139 | 0.956 | 0.777 | 4.07E-238 |
| C02 | Knstrn | 3.35E-240 | 0.588305194 | 0.606 | 0.287 | 7.56E-236 |
| C02 | Hsp90b1 | 3.73E-225 | 0.512481259 | 0.994 | 0.928 | 8.41E-221 |
| C02 | Diaph3 | 4.91E-221 | 0.567948458 | 0.64 | 0.334 | 1.11E-216 |
| C02 | Anp32e | 8.25E-218 | 0.593749426 | 0.911 | 0.69 | 1.86E-213 |
| C02 | Ran | 7.49E-217 | 0.553870388 | 0.972 | 0.848 | 1.69E-212 |
| C02 | Tacc3 | 2.20E-216 | 0.524008649 | 0.682 | 0.35 | 4.96E-212 |
| C02 | Hmgn2 | 7.83E-204 | 0.602528644 | 0.786 | 0.512 | 1.76E-199 |
| C02 | H2afz | 1.07E-196 | 0.518779576 | 0.965 | 0.789 | 2.42E-192 |
| C02 | H1fx | 5.29E-192 | 0.528949925 | 0.893 | 0.604 | 1.19E-187 |
| C02 | Cks2 | 9.66E-191 | 0.61577963 | 0.749 | 0.462 | 2.18E-186 |
| C02 | Hist1h2bc | 3.48E-189 | 0.709895952 | 0.967 | 0.835 | 7.85E-185 |
| C02 | Ckap2l | 6.79E-187 | 0.511095858 | 0.646 | 0.329 | 1.53E-182 |
| C02 | Hp1bp3 | 2.39E-180 | 0.531444186 | 0.92 | 0.743 | 5.39E-176 |
| C02 | Ckap5 | 9.64E-174 | 0.507754208 | 0.681 | 0.419 | 2.17E-169 |
| C02 | Cenpw | 5.42E-166 | 0.508733378 | 0.728 | 0.457 | 1.22E-161 |
| C02 | Klra4 | 1.21E-164 | 0.603196324 | 0.853 | 0.638 | 2.73E-160 |
| C02 | H2afv | 1.62E-164 | 0.501096202 | 0.926 | 0.736 | 3.64E-160 |
| C02 | Hmgn3 | 1.35E-160 | 0.507543937 | 0.749 | 0.528 | 3.05E-156 |
| C02 | Nup37 | 4.19E-152 | 0.528638767 | 0.691 | 0.461 | 9.44E-148 |
| C02 | Hist1h1c | 1.81E-104 | 0.533938877 | 0.875 | 0.735 | 4.08E-100 |
| C03 | Ube2c | 0 | 2.637168071 | 0.974 | 0.43 | 0 |
| C03 | Prc1 | 0 | 2.241733618 | 0.968 | 0.367 | 0 |
| C03 | Cenpf | 0 | 2.239196326 | 0.982 | 0.375 | 0 |
| C03 | Cdc20 | 0 | 2.139652686 | 0.987 | 0.364 | 0 |
| C03 | Ccnb1 | 0 | 2.088690919 | 0.962 | 0.303 | 0 |
| C03 | Ccnb2 | 0 | 2.024247637 | 1 | 0.592 | 0 |
| C03 | Cenpa | 0 | 1.949716253 | 0.993 | 0.416 | 0 |
| C03 | Nusap1 | 0 | 1.868908108 | 0.925 | 0.274 | 0 |
| C03 | Tpx2 | 0 | 1.7244697 | 0.979 | 0.383 | 0 |
| C03 | Ckap2l | 0 | 1.690062586 | 0.955 | 0.3 | 0 |
| C03 | Birc5 | 0 | 1.688936056 | 0.983 | 0.396 | 0 |
| C03 | Pimreg | 0 | 1.654954607 | 0.943 | 0.302 | 0 |
| C03 | Cks2 | 0 | 1.645990474 | 0.974 | 0.442 | 0 |
| C03 | Cenpe | 0 | 1.611631266 | 0.95 | 0.318 | 0 |
| C03 | Cdk1 | 0 | 1.603615561 | 0.888 | 0.431 | 0 |
| C03 | Tubb4b | 0 | 1.601489107 | 0.968 | 0.554 | 0 |
| C03 | Cdca8 | 0 | 1.594719094 | 0.975 | 0.376 | 0 |
| C03 | Ccna2 | 0 | 1.594354522 | 0.967 | 0.34 | 0 |
| C03 | Hmmr | 0 | 1.590948698 | 0.945 | 0.251 | 0 |
| C03 | Top2a | 0 | 1.588014776 | 0.942 | 0.457 | 0 |
| C03 | Plk1 | 0 | 1.587975864 | 0.902 | 0.209 | 0 |
| C03 | Hmgb2 | 0 | 1.573626235 | 1 | 0.825 | 0 |
| C03 | Racgap1 | 0 | 1.548251829 | 0.988 | 0.503 | 0 |
| C03 | Ckap2 | 0 | 1.545586493 | 0.942 | 0.293 | 0 |
| C03 | Cdkn3 | 0 | 1.543024969 | 0.912 | 0.251 | 0 |
| C03 | Anln | 0 | 1.520521958 | 0.969 | 0.435 | 0 |
| C03 | Kif20a | 0 | 1.507144201 | 0.933 | 0.274 | 0 |
| C03 | Tuba1c | 0 | 1.50517157 | 0.955 | 0.729 | 0 |
| C03 | Aurka | 0 | 1.492341627 | 0.873 | 0.177 | 0 |
| C03 | Kpna2 | 0 | 1.451792964 | 0.75 | 0.23 | 0 |
| C03 | Tacc3 | 0 | 1.423551707 | 0.937 | 0.327 | 0 |
| C03 | Mki67 | 0 | 1.42191711 | 0.965 | 0.424 | 0 |
| C03 | Smc4 | 0 | 1.39317089 | 0.992 | 0.663 | 0 |
| C03 | H2afx | 0 | 1.310180949 | 0.873 | 0.472 | 0 |
| C03 | Hn1 | 0 | 1.297786369 | 0.969 | 0.776 | 0 |
| C03 | Kif2c | 0 | 1.285706104 | 0.863 | 0.196 | 0 |
| C03 | Kif23 | 0 | 1.279503393 | 0.881 | 0.241 | 0 |
| C03 | Aspm | 0 | 1.274099484 | 0.814 | 0.152 | 0 |
| C03 | Lockd | 0 | 1.269530749 | 0.922 | 0.384 | 0 |
| C03 | Nek2 | 0 | 1.263058085 | 0.868 | 0.208 | 0 |
| C03 | Tubb6 | 0 | 1.240221868 | 0.949 | 0.506 | 0 |
| C03 | Cks1b | 0 | 1.218648955 | 0.984 | 0.602 | 0 |
| C03 | Smc2 | 0 | 1.215473222 | 0.958 | 0.462 | 0 |
| C03 | Hjurp | 0 | 1.210334647 | 0.906 | 0.4 | 0 |
| C03 | Sgol2a | 0 | 1.207424053 | 0.845 | 0.202 | 0 |
| C03 | Knstrn | 0 | 1.201221927 | 0.882 | 0.261 | 0 |
| C03 | Cdca3 | 0 | 1.180985982 | 0.872 | 0.294 | 0 |
| C03 | Kif20b | 0 | 1.173684303 | 0.826 | 0.204 | 0 |
| C03 | Bub3 | 0 | 1.152283519 | 0.905 | 0.646 | 0 |
| C03 | H1fx | 0 | 1.140642083 | 0.965 | 0.6 | 0 |
| C03 | Trim59 | 0 | 1.136666091 | 0.889 | 0.352 | 0 |
| C03 | Kif22 | 0 | 1.121865458 | 0.835 | 0.227 | 0 |
| C03 | Kif11 | 0 | 1.102911977 | 0.824 | 0.214 | 0 |
| C03 | Dbf4 | 0 | 1.097590985 | 0.862 | 0.293 | 0 |
| C03 | Calm2 | 0 | 1.090416469 | 0.999 | 0.944 | 0 |
| C03 | Kifc1 | 0 | 1.085397629 | 0.809 | 0.171 | 0 |
| C03 | Dynll1 | 0 | 1.043838429 | 0.989 | 0.843 | 0 |
| C03 | Ect2 | 0 | 1.040912098 | 0.827 | 0.211 | 0 |
| C03 | Ndc80 | 0 | 1.028771107 | 0.814 | 0.232 | 0 |
| C03 | Hmgb3 | 0 | 1.017935569 | 0.961 | 0.613 | 0 |
| C03 | Cep55 | 0 | 0.999658009 | 0.747 | 0.149 | 0 |
| C03 | Dlgap5 | 0 | 0.997045069 | 0.799 | 0.212 | 0 |
| C03 | Rad21 | 0 | 0.994676573 | 0.977 | 0.665 | 0 |
| C03 | Sgo1 | 0 | 0.977507325 | 0.786 | 0.207 | 0 |
| C03 | Aurkb | 0 | 0.975282949 | 0.752 | 0.236 | 0 |
| C03 | Cenpq | 0 | 0.952907791 | 0.868 | 0.332 | 0 |
| C03 | Troap | 0 | 0.943051687 | 0.723 | 0.126 | 0 |
| C03 | Arhgap11a | 0 | 0.937075734 | 0.757 | 0.19 | 0 |
| C03 | Miip | 0 | 0.928649616 | 0.718 | 0.169 | 0 |
| C03 | Reep4 | 0 | 0.92033734 | 0.802 | 0.268 | 0 |
| C03 | Tubb5 | 0 | 0.919078601 | 0.994 | 0.913 | 0 |
| C03 | Nucks1 | 0 | 0.917429401 | 0.992 | 0.776 | 0 |
| C03 | Sapcd2 | 0 | 0.91694171 | 0.678 | 0.105 | 0 |
| C03 | Ckap5 | 0 | 0.905607321 | 0.865 | 0.403 | 0 |
| C03 | Tmpo | 0 | 0.900781647 | 0.939 | 0.581 | 0 |
| C03 | Mad2l1 | 0 | 0.889586196 | 0.863 | 0.358 | 0 |
| C03 | H2afv | 0 | 0.888416264 | 0.971 | 0.733 | 0 |
| C03 | Terf1 | 0 | 0.874120879 | 0.811 | 0.313 | 0 |
| C03 | Lmnb1 | 0 | 0.873024324 | 0.799 | 0.326 | 0 |
| C03 | Tuba1a | 0 | 0.872315648 | 0.992 | 0.905 | 0 |
| C03 | Tuba1b | 0 | 0.86702109 | 0.991 | 0.91 | 0 |
| C03 | Shcbp1 | 0 | 0.866698629 | 0.806 | 0.288 | 0 |
| C03 | Bub1b | 0 | 0.860592021 | 0.767 | 0.235 | 0 |
| C03 | Klra4 | 0 | 0.849823254 | 0.935 | 0.632 | 0 |
| C03 | Nup37 | 0 | 0.848630915 | 0.862 | 0.446 | 0 |
| C03 | Knl1 | 0 | 0.848023779 | 0.718 | 0.19 | 0 |
| C03 | Incenp | 0 | 0.840200887 | 0.748 | 0.224 | 0 |
| C03 | Mis18bp1 | 0 | 0.833748328 | 0.659 | 0.147 | 0 |
| C03 | Spc25 | 0 | 0.833294711 | 0.709 | 0.204 | 0 |
| C03 | Prr11 | 0 | 0.824949644 | 0.685 | 0.135 | 0 |
| C03 | Selenoh | 0 | 0.82398016 | 0.965 | 0.664 | 0 |
| C03 | Cdc25c | 0 | 0.808490668 | 0.658 | 0.116 | 0 |
| C03 | Nde1 | 0 | 0.806117978 | 0.803 | 0.338 | 0 |
| C03 | Ptms | 0 | 0.802547158 | 0.997 | 0.911 | 0 |
| C03 | Cenpw | 0 | 0.80205325 | 0.888 | 0.443 | 0 |
| C03 | H2afz | 0 | 0.800921499 | 0.988 | 0.789 | 0 |
| C03 | Fam83d | 0 | 0.793571164 | 0.574 | 0.092 | 0 |
| C03 | Ubald2 | 0 | 0.792502893 | 0.836 | 0.384 | 0 |
| C03 | Ifi27l2a | 2.66E-230 | 1.05235535 | 0.825 | 0.523 | 6.00E-226 |
| C03 | Tubb3 | 1.42E-211 | 0.833446694 | 0.945 | 0.76 | 3.20E-207 |
| C04 | Pclaf | 0 | 1.831433056 | 0.99 | 0.428 | 0 |
| C04 | Tk1 | 0 | 1.666519727 | 0.984 | 0.409 | 0 |
| C04 | Top2a | 0 | 1.545975988 | 0.995 | 0.453 | 0 |
| C04 | Pbk | 0 | 1.425461069 | 0.965 | 0.306 | 0 |
| C04 | Tuba1b | 0 | 1.410220773 | 1 | 0.91 | 0 |
| C04 | Rrm2 | 0 | 1.355378746 | 0.916 | 0.304 | 0 |
| C04 | Cenph | 0 | 1.329410725 | 0.977 | 0.448 | 0 |
| C04 | Stmn1 | 0 | 1.328439224 | 0.996 | 0.639 | 0 |
| C04 | Tubb5 | 0 | 1.299512975 | 1 | 0.913 | 0 |
| C04 | Hist1h4d | 0 | 1.217488536 | 0.836 | 0.219 | 0 |
| C04 | Hist1h1b | 0 | 1.200903442 | 0.746 | 0.134 | 0 |
| C04 | Hist1h2ae | 0 | 1.157599947 | 0.846 | 0.27 | 0 |
| C04 | Hist1h1e | 0 | 1.149489057 | 0.801 | 0.259 | 0 |
| C04 | Asf1b | 0 | 1.134209079 | 0.938 | 0.349 | 0 |
| C04 | Birc5 | 0 | 1.130071865 | 0.968 | 0.4 | 0 |
| C04 | Cdk1 | 0 | 1.105494166 | 0.971 | 0.425 | 0 |
| C04 | Aurkb | 0 | 1.095330345 | 0.878 | 0.225 | 0 |
| C04 | Smc2 | 0 | 1.090490924 | 0.975 | 0.462 | 0 |
| C04 | Lig1 | 0 | 1.078049233 | 0.929 | 0.489 | 0 |
| C04 | Spc24 | 0 | 1.064511233 | 0.906 | 0.326 | 0 |
| C04 | Tubb3 | 0 | 1.037154845 | 0.988 | 0.756 | 0 |
| C04 | Tyms | 0 | 1.032420957 | 0.864 | 0.311 | 0 |
| C04 | Atad2 | 0 | 1.005405398 | 0.899 | 0.393 | 0 |
| C04 | Rrm1 | 0 | 0.995509858 | 0.947 | 0.493 | 0 |
| C04 | Nusap1 | 0 | 0.968273528 | 0.878 | 0.282 | 0 |
| C04 | Spc25 | 0 | 0.92645365 | 0.785 | 0.198 | 0 |
| C04 | Esco2 | 0 | 0.914286691 | 0.762 | 0.14 | 0 |
| C04 | H2afz | 0 | 0.895200998 | 0.997 | 0.789 | 0 |
| C04 | Mki67 | 0 | 0.890785347 | 0.932 | 0.429 | 0 |
| C04 | Hmgb2 | 0 | 0.889484144 | 0.999 | 0.826 | 0 |
| C04 | Pcna | 0 | 0.888586686 | 0.914 | 0.556 | 0 |
| C04 | Clspn | 0 | 0.861868709 | 0.772 | 0.233 | 0 |
| C04 | Tuba1c | 0 | 0.855881422 | 0.979 | 0.727 | 0 |
| C04 | Ccna2 | 0 | 0.855780707 | 0.917 | 0.347 | 0 |
| C04 | Prc1 | 0 | 0.854352587 | 0.913 | 0.375 | 0 |
| C04 | Smc4 | 0 | 0.839959321 | 0.991 | 0.664 | 0 |
| C04 | Ube2c | 0 | 0.836673422 | 0.936 | 0.437 | 0 |
| C04 | Cks1b | 0 | 0.816264626 | 0.985 | 0.604 | 0 |
| C04 | Ndc80 | 0 | 0.813538466 | 0.784 | 0.238 | 0 |
| C04 | Shcbp1 | 0 | 0.803307696 | 0.838 | 0.286 | 0 |
| C04 | Tuba1a | 0 | 0.801840589 | 0.999 | 0.905 | 0 |
| C04 | Cenpm | 0 | 0.769859052 | 0.798 | 0.272 | 0 |
| C04 | H2afx | 0 | 0.76740549 | 0.901 | 0.471 | 0 |
| C04 | Hist1h2ab | 0 | 0.765759836 | 0.611 | 0.085 | 0 |
| C04 | Mxd3 | 0 | 0.764028645 | 0.683 | 0.143 | 0 |
| C04 | Tubb6 | 0 | 0.760194306 | 0.938 | 0.509 | 0 |
| C04 | Cenpq | 0 | 0.759920247 | 0.871 | 0.334 | 0 |
| C04 | Melk | 0 | 0.757025372 | 0.763 | 0.207 | 0 |
| C04 | Pola1 | 0 | 0.750552758 | 0.808 | 0.313 | 0 |
| C04 | Rfc4 | 0 | 0.745137651 | 0.857 | 0.378 | 0 |
| C04 | Dek | 0 | 0.738687635 | 0.985 | 0.77 | 0 |
| C04 | Racgap1 | 0 | 0.736533674 | 0.958 | 0.508 | 0 |
| C04 | Dnmt1 | 0 | 0.732357057 | 0.884 | 0.464 | 0 |
| C04 | Tcf19 | 0 | 0.73221632 | 0.656 | 0.176 | 0 |
| C04 | Gmnn | 0 | 0.720569306 | 0.833 | 0.354 | 0 |
| C04 | Fbxo5 | 0 | 0.714902246 | 0.702 | 0.165 | 0 |
| C04 | Mad2l1 | 0 | 0.711931944 | 0.868 | 0.36 | 0 |
| C04 | Ezh2 | 0 | 0.711529187 | 0.929 | 0.537 | 0 |
| C04 | Nup85 | 0 | 0.703328146 | 0.883 | 0.434 | 0 |
| C04 | Orc6 | 0 | 0.692776192 | 0.908 | 0.514 | 0 |
| C04 | Ncaph | 0 | 0.672319638 | 0.702 | 0.197 | 0 |
| C04 | Incenp | 0 | 0.670561754 | 0.738 | 0.228 | 0 |
| C04 | Kif11 | 0 | 0.669401621 | 0.73 | 0.226 | 0 |
| C04 | Cdca8 | 0 | 0.657275117 | 0.895 | 0.387 | 0 |
| C04 | Mcm5 | 0 | 0.654235226 | 0.826 | 0.399 | 0 |
| C04 | Cdkn2d | 0 | 0.648601013 | 0.702 | 0.24 | 0 |
| C04 | Pimreg | 0 | 0.641976817 | 0.838 | 0.315 | 0 |
| C04 | Lockd | 0 | 0.640184554 | 0.89 | 0.389 | 0 |
| C04 | Ckap2l | 0 | 0.631488974 | 0.83 | 0.316 | 0 |
| C04 | Dynlt1f | 0 | 0.629353047 | 0.839 | 0.392 | 0 |
| C04 | Slfn9 | 0 | 0.627595424 | 0.611 | 0.221 | 0 |
| C04 | Anln | 0 | 0.616537717 | 0.913 | 0.442 | 0 |
| C04 | Ncapd3 | 0 | 0.61204342 | 0.728 | 0.264 | 0 |
| C04 | Hat1 | 0 | 0.6119748 | 0.795 | 0.367 | 0 |
| C04 | Rad51ap1 | 0 | 0.6062816 | 0.622 | 0.148 | 0 |
| C04 | Prim1 | 0 | 0.605879899 | 0.737 | 0.297 | 0 |
| C04 | Rpa1 | 0 | 0.598686148 | 0.792 | 0.37 | 0 |
| C04 | Cenpk | 0 | 0.597470869 | 0.722 | 0.267 | 0 |
| C04 | Dnajc9 | 0 | 0.594316334 | 0.813 | 0.384 | 0 |
| C04 | Tubg1 | 0 | 0.577559524 | 0.78 | 0.345 | 0 |
| C04 | Nrm | 0 | 0.577055178 | 0.726 | 0.269 | 0 |
| C04 | Diaph3 | 0 | 0.565843914 | 0.786 | 0.324 | 0 |
| C04 | Tmpo | 5.91E-305 | 0.657206055 | 0.951 | 0.581 | 1.33E-300 |
| C04 | Usp1 | 6.01E-305 | 0.662218999 | 0.916 | 0.535 | 1.36E-300 |
| C04 | Rpa3 | 6.90E-302 | 0.654892018 | 0.917 | 0.551 | 1.56E-297 |
| C04 | Hmgb1 | 7.18E-300 | 0.620985691 | 0.998 | 0.938 | 1.62E-295 |
| C04 | Lsm2 | 1.51E-288 | 0.627250631 | 0.911 | 0.529 | 3.40E-284 |
| C04 | Dut | 4.16E-285 | 0.678311816 | 0.959 | 0.649 | 9.39E-281 |
| C04 | Tubb4b | 9.65E-278 | 0.624467557 | 0.939 | 0.558 | 2.18E-273 |
| C04 | Cbx5 | 9.21E-275 | 0.684406292 | 0.945 | 0.651 | 2.08E-270 |
| C04 | Cenpw | 1.33E-267 | 0.574798491 | 0.869 | 0.447 | 3.01E-263 |
| C04 | Tipin | 7.30E-263 | 0.585816406 | 0.883 | 0.504 | 1.65E-258 |
| C04 | Prdx4 | 8.63E-260 | 0.597247704 | 0.981 | 0.768 | 1.95E-255 |
| C04 | Alyref | 8.62E-257 | 0.594631198 | 0.97 | 0.711 | 1.94E-252 |
| C04 | Gins2 | 5.17E-253 | 0.565536558 | 0.799 | 0.405 | 1.17E-248 |
| C04 | Dctpp1 | 7.03E-247 | 0.592192904 | 0.827 | 0.454 | 1.58E-242 |
| C04 | Anp32e | 2.21E-230 | 0.572838857 | 0.965 | 0.689 | 4.99E-226 |
| C04 | Ranbp1 | 7.59E-205 | 0.581414152 | 0.983 | 0.83 | 1.71E-200 |
| C04 | H1fx | 2.31E-199 | 0.644991613 | 0.922 | 0.606 | 5.20E-195 |
| C04 | Tnnt2 | 3.21E-133 | 0.573168112 | 0.873 | 0.602 | 7.23E-129 |
| C05 | Top2a | 0 | 1.366237761 | 0.99 | 0.466 | 0 |
| C05 | Ube2c | 0 | 1.221520335 | 0.967 | 0.446 | 0 |
| C05 | Prc1 | 0 | 1.118787222 | 0.955 | 0.384 | 0 |
| C05 | Pbk | 0 | 1.111157449 | 0.925 | 0.324 | 0 |
| C05 | Cdk1 | 0 | 1.100989742 | 0.975 | 0.437 | 0 |
| C05 | Nusap1 | 0 | 1.097994131 | 0.924 | 0.292 | 0 |
| C05 | H2afx | 0 | 1.074684055 | 0.938 | 0.478 | 0 |
| C05 | Hmgb2 | 0 | 1.055111788 | 0.999 | 0.83 | 0 |
| C05 | Smc2 | 0 | 0.985182928 | 0.975 | 0.473 | 0 |
| C05 | Hist1h2ae | 0 | 0.963337996 | 0.841 | 0.284 | 0 |
| C05 | Birc5 | 0 | 0.944818348 | 0.974 | 0.413 | 0 |
| C05 | Cks1b | 0 | 0.906057916 | 0.988 | 0.612 | 0 |
| C05 | Smc4 | 0 | 0.89491259 | 0.993 | 0.671 | 0 |
| C05 | Pclaf | 0 | 0.892845014 | 0.937 | 0.445 | 0 |
| C05 | Tuba1b | 0 | 0.885943128 | 0.997 | 0.912 | 0 |
| C05 | Ccna2 | 0 | 0.867288532 | 0.939 | 0.359 | 0 |
| C05 | Anln | 0 | 0.866506856 | 0.941 | 0.451 | 0 |
| C05 | Aurkb | 0 | 0.866381635 | 0.848 | 0.243 | 0 |
| C05 | Cdca8 | 0 | 0.796044357 | 0.941 | 0.395 | 0 |
| C05 | Hist1h4d | 0 | 0.790184093 | 0.748 | 0.24 | 0 |
| C05 | Lockd | 0 | 0.740798144 | 0.902 | 0.4 | 0 |
| C05 | Spc24 | 0 | 0.721580473 | 0.854 | 0.344 | 0 |
| C05 | Asf1b | 0 | 0.711532112 | 0.891 | 0.367 | 0 |
| C05 | Spc25 | 0 | 0.707870103 | 0.777 | 0.213 | 0 |
| C05 | Hist1h1b | 0 | 0.692469374 | 0.632 | 0.157 | 0 |
| C05 | Ckap2l | 0 | 0.691005932 | 0.874 | 0.324 | 0 |
| C05 | Esco2 | 0 | 0.690113244 | 0.679 | 0.161 | 0 |
| C05 | Kif11 | 0 | 0.68577541 | 0.784 | 0.234 | 0 |
| C05 | Incenp | 0 | 0.672382158 | 0.767 | 0.237 | 0 |
| C05 | Kpna2 | 0 | 0.667307137 | 0.721 | 0.246 | 0 |
| C05 | H3f3b | 0 | 0.626612755 | 1 | 0.995 | 0 |
| C05 | Shcbp1 | 0 | 0.599966359 | 0.814 | 0.301 | 0 |
| C05 | Sgo1 | 0 | 0.597794611 | 0.733 | 0.226 | 0 |
| C05 | Fbxo5 | 0 | 0.590573939 | 0.653 | 0.181 | 0 |
| C05 | Hist1h2ab | 0 | 0.577609143 | 0.543 | 0.102 | 0 |
| C05 | Plk4 | 0 | 0.565264674 | 0.74 | 0.254 | 0 |
| C05 | Ndc80 | 0 | 0.552454046 | 0.743 | 0.254 | 0 |
| C05 | Ube2t | 0 | 0.549416265 | 0.709 | 0.233 | 0 |
| C05 | Ska1 | 0 | 0.539802984 | 0.65 | 0.194 | 0 |
| C05 | 2700099C18Rik | 0 | 0.535731969 | 0.715 | 0.237 | 0 |
| C05 | Melk | 6.30E-306 | 0.518850663 | 0.676 | 0.227 | 1.42E-301 |
| C05 | Cenph | 2.09E-301 | 0.817652891 | 0.928 | 0.464 | 4.71E-297 |
| C05 | Cenpq | 1.39E-296 | 0.594583157 | 0.851 | 0.348 | 3.14E-292 |
| C05 | Ccnb1 | 3.36E-296 | 0.648184639 | 0.824 | 0.331 | 7.58E-292 |
| C05 | Mki67 | 1.64E-289 | 0.742931777 | 0.929 | 0.441 | 3.71E-285 |
| C05 | Cdkn2d | 6.20E-289 | 0.53981582 | 0.705 | 0.25 | 1.40E-284 |
| C05 | Stmn1 | 1.89E-288 | 0.871074161 | 0.982 | 0.648 | 4.27E-284 |
| C05 | Mad2l1 | 1.10E-287 | 0.600684263 | 0.861 | 0.372 | 2.47E-283 |
| C05 | Tubb5 | 7.25E-287 | 0.803979907 | 0.997 | 0.915 | 1.63E-282 |
| C05 | Racgap1 | 1.13E-285 | 0.700891717 | 0.977 | 0.517 | 2.54E-281 |
| C05 | Tpx2 | 1.58E-283 | 0.601930538 | 0.924 | 0.403 | 3.55E-279 |
| C05 | Hmmr | 2.73E-282 | 0.576786477 | 0.773 | 0.283 | 6.15E-278 |
| C05 | Pimreg | 2.52E-280 | 0.535239336 | 0.832 | 0.328 | 5.69E-276 |
| C05 | Cks2 | 2.94E-280 | 0.666434041 | 0.929 | 0.46 | 6.62E-276 |
| C05 | Atad2 | 5.10E-277 | 0.728593938 | 0.855 | 0.408 | 1.15E-272 |
| C05 | Calm2 | 5.62E-270 | 0.588124558 | 0.998 | 0.945 | 1.27E-265 |
| C05 | Dek | 5.03E-259 | 0.700525752 | 0.984 | 0.775 | 1.14E-254 |
| C05 | Hist1h1e | 2.43E-255 | 0.576011562 | 0.71 | 0.279 | 5.49E-251 |
| C05 | Tacc3 | 4.95E-253 | 0.523404189 | 0.837 | 0.351 | 1.12E-248 |
| C05 | Hist1h4i | 7.07E-243 | 0.603987931 | 0.694 | 0.277 | 1.59E-238 |
| C05 | Tsc22d1 | 1.08E-233 | 0.830177841 | 0.978 | 0.782 | 2.43E-229 |
| C05 | Rrm1 | 2.04E-222 | 0.619046191 | 0.909 | 0.507 | 4.60E-218 |
| C05 | Ezh2 | 2.49E-218 | 0.643754126 | 0.924 | 0.546 | 5.62E-214 |
| C05 | Bub3 | 4.05E-210 | 0.603246578 | 0.948 | 0.649 | 9.12E-206 |
| C05 | Tuba1c | 5.17E-210 | 0.733725552 | 0.963 | 0.734 | 1.17E-205 |
| C05 | Ddit3 | 3.59E-201 | 0.69736932 | 0.78 | 0.383 | 8.10E-197 |
| C05 | Rrm2 | 7.57E-199 | 0.533214702 | 0.745 | 0.331 | 1.71E-194 |
| C05 | Psat1 | 9.22E-197 | 0.621062101 | 0.853 | 0.484 | 2.08E-192 |
| C05 | Ezr | 3.85E-194 | 0.634570175 | 0.953 | 0.716 | 8.67E-190 |
| C05 | Sap30 | 8.42E-192 | 0.545308342 | 0.861 | 0.475 | 1.90E-187 |
| C05 | Tk1 | 4.89E-187 | 0.625353449 | 0.839 | 0.434 | 1.10E-182 |
| C05 | Tmpo | 1.75E-186 | 0.525450302 | 0.938 | 0.591 | 3.94E-182 |
| C05 | Hsp90aa1 | 1.23E-183 | 0.569916082 | 0.994 | 0.872 | 2.77E-179 |
| C05 | Anp32e | 1.88E-183 | 0.581470085 | 0.956 | 0.696 | 4.24E-179 |
| C05 | Hmgb1 | 1.91E-182 | 0.504697399 | 0.998 | 0.939 | 4.32E-178 |
| C05 | Hspa9 | 9.98E-180 | 0.633065929 | 0.983 | 0.813 | 2.25E-175 |
| C05 | Snhg5 | 7.49E-179 | 0.564155754 | 0.92 | 0.604 | 1.69E-174 |
| C05 | Nucks1 | 2.28E-176 | 0.541315609 | 0.99 | 0.781 | 5.13E-172 |
| C05 | H1fx | 4.08E-176 | 0.66427132 | 0.941 | 0.612 | 9.20E-172 |
| C05 | Rad21 | 4.21E-167 | 0.514406797 | 0.962 | 0.674 | 9.48E-163 |
| C05 | H2afz | 2.95E-161 | 0.506256338 | 0.989 | 0.794 | 6.64E-157 |
| C05 | Alyref | 9.31E-158 | 0.509950798 | 0.964 | 0.717 | 2.10E-153 |
| C05 | Hmga1 | 1.15E-151 | 0.626276425 | 0.955 | 0.723 | 2.59E-147 |
| C05 | Asns | 4.92E-149 | 0.50325786 | 0.85 | 0.503 | 1.11E-144 |
| C05 | 2410006H16Rik | 5.43E-144 | 0.566299105 | 0.997 | 0.948 | 1.22E-139 |
| C05 | H1f0 | 1.87E-138 | 0.639582794 | 1 | 0.962 | 4.21E-134 |
| C05 | Tubb3 | 7.26E-106 | 0.531228321 | 0.962 | 0.763 | 1.64E-101 |
| C05 | Tnnt2 | 5.66E-102 | 0.555120216 | 0.866 | 0.608 | 1.28E-97 |
| C05 | Hspa5 | 1.89E-97 | 0.530731444 | 0.999 | 0.942 | 4.25E-93 |
| C05 | Cryab | 1.75E-49 | 0.506873467 | 0.679 | 0.462 | 3.95E-45 |
| C06 | Ngfr | 0 | 0.74345841 | 0.622 | 0.094 | 0 |
| C06 | Klk8 | 5.94E-246 | 0.822902657 | 0.685 | 0.146 | 1.34E-241 |
| C06 | Slc14a1 | 1.37E-238 | 1.005434033 | 0.755 | 0.187 | 3.09E-234 |
| C06 | Ggct | 1.83E-197 | 0.735664315 | 0.604 | 0.137 | 4.12E-193 |
| C06 | Col18a1 | 2.55E-151 | 0.845276423 | 0.803 | 0.3 | 5.76E-147 |
| C06 | Ctsl | 6.17E-150 | 0.893119162 | 0.998 | 0.935 | 1.39E-145 |
| C06 | Hmga1 | 2.69E-138 | 0.961496081 | 1 | 0.733 | 6.06E-134 |
| C06 | Hells | 6.28E-136 | 0.932944078 | 0.924 | 0.449 | 1.41E-131 |
| C06 | Eps8 | 2.00E-123 | 0.87613294 | 0.982 | 0.689 | 4.51E-119 |
| C06 | Ccnd1 | 1.07E-122 | 0.8482572 | 0.998 | 0.852 | 2.40E-118 |
| C06 | Mcm6 | 2.89E-122 | 0.901052896 | 0.99 | 0.702 | 6.51E-118 |
| C06 | Mcm3 | 7.56E-121 | 0.878764927 | 0.944 | 0.519 | 1.70E-116 |
| C06 | Mcm2 | 1.38E-120 | 0.897814584 | 0.98 | 0.627 | 3.11E-116 |
| C06 | Esx1 | 9.45E-115 | 0.510328523 | 0.616 | 0.195 | 2.13E-110 |
| C06 | Ncl | 8.26E-112 | 0.664460484 | 1 | 0.974 | 1.86E-107 |
| C06 | Psat1 | 1.60E-110 | 0.75611305 | 0.944 | 0.5 | 3.60E-106 |
| C06 | Igfbp4 | 3.26E-110 | 0.796887196 | 0.996 | 0.86 | 7.35E-106 |
| C06 | Casp3 | 7.71E-109 | 1.030491112 | 0.98 | 0.725 | 1.74E-104 |
| C06 | Inhba | 8.49E-106 | 0.780867123 | 0.825 | 0.382 | 1.91E-101 |
| C06 | Dusp9 | 2.38E-102 | 0.602043697 | 0.657 | 0.25 | 5.38E-98 |
| C06 | Galnt1 | 1.46E-101 | 0.929099748 | 0.96 | 0.645 | 3.29E-97 |
| C06 | Tnfrsf12a | 6.56E-101 | 0.823310529 | 0.994 | 0.696 | 1.48E-96 |
| C06 | Slc25a5 | 7.86E-99 | 0.673086694 | 1 | 0.953 | 1.77E-94 |
| C06 | Ranbp1 | 1.19E-93 | 0.653728263 | 1 | 0.841 | 2.68E-89 |
| C06 | Fkbp4 | 1.45E-93 | 0.683010659 | 0.964 | 0.673 | 3.26E-89 |
| C06 | Upp1 | 5.24E-93 | 0.642388337 | 0.831 | 0.396 | 1.18E-88 |
| C06 | Tpm1 | 1.23E-92 | 0.700527568 | 1 | 0.879 | 2.78E-88 |
| C06 | Mcm5 | 2.42E-90 | 0.649457742 | 0.849 | 0.429 | 5.45E-86 |
| C06 | Fosl1 | 3.02E-90 | 0.775400343 | 0.861 | 0.461 | 6.82E-86 |
| C06 | Lgals3 | 3.51E-90 | 0.582512451 | 1 | 0.974 | 7.90E-86 |
| C06 | Hspd1 | 8.00E-88 | 0.641943305 | 1 | 0.841 | 1.80E-83 |
| C06 | Mcm4 | 1.14E-86 | 0.620837761 | 0.815 | 0.397 | 2.57E-82 |
| C06 | Mcm7 | 4.23E-86 | 0.639222977 | 0.863 | 0.456 | 9.53E-82 |
| C06 | Hmga2 | 5.48E-86 | 0.687619847 | 0.902 | 0.482 | 1.24E-81 |
| C06 | Lamc2 | 1.05E-83 | 0.6287151 | 0.876 | 0.491 | 2.38E-79 |
| C06 | Hspa9 | 2.69E-83 | 0.656270336 | 0.998 | 0.822 | 6.06E-79 |
| C06 | Eno1 | 4.10E-83 | 0.516750075 | 1 | 0.97 | 9.25E-79 |
| C06 | Cisd3 | 7.95E-83 | 0.629508161 | 0.849 | 0.467 | 1.79E-78 |
| C06 | Ezr | 3.76E-81 | 0.683918699 | 0.97 | 0.728 | 8.47E-77 |
| C06 | Pla2g7 | 6.13E-80 | 0.664101768 | 0.982 | 0.764 | 1.38E-75 |
| C06 | Htra1 | 6.38E-78 | 0.621061828 | 0.998 | 0.828 | 1.44E-73 |
| C06 | Eif5a | 4.85E-77 | 0.521486584 | 1 | 0.966 | 1.09E-72 |
| C06 | Set | 6.41E-76 | 0.544157917 | 1 | 0.884 | 1.44E-71 |
| C06 | Ybx3 | 1.63E-75 | 0.558496592 | 0.994 | 0.861 | 3.68E-71 |
| C06 | Banf1 | 9.24E-75 | 0.552474352 | 1 | 0.925 | 2.08E-70 |
| C06 | Nolc1 | 8.60E-74 | 0.616165268 | 0.886 | 0.521 | 1.94E-69 |
| C06 | Siva1 | 1.44E-73 | 0.596802806 | 0.922 | 0.585 | 3.26E-69 |
| C06 | Zfp706 | 7.19E-72 | 0.516369729 | 1 | 0.856 | 1.62E-67 |
| C06 | Tinagl1 | 2.08E-70 | 0.628124381 | 0.926 | 0.571 | 4.69E-66 |
| C06 | Nup85 | 6.91E-69 | 0.523824462 | 0.847 | 0.468 | 1.56E-64 |
| C06 | Dnmt1 | 1.30E-68 | 0.551335824 | 0.869 | 0.494 | 2.94E-64 |
| C06 | Gsr | 1.42E-67 | 0.548038452 | 0.886 | 0.548 | 3.19E-63 |
| C06 | Srm | 1.64E-67 | 0.597943398 | 0.942 | 0.611 | 3.69E-63 |
| C06 | Sox9 | 1.04E-66 | 0.571002765 | 0.96 | 0.69 | 2.33E-62 |
| C06 | Map4k1 | 1.19E-66 | 0.549305707 | 0.759 | 0.376 | 2.69E-62 |
| C06 | Asns | 1.42E-66 | 0.558780545 | 0.9 | 0.519 | 3.20E-62 |
| C06 | Kcnn4 | 1.40E-65 | 0.504927569 | 0.888 | 0.521 | 3.16E-61 |
| C06 | Hnrnpab | 8.15E-65 | 0.527283085 | 0.994 | 0.849 | 1.84E-60 |
| C06 | Klra4 | 6.99E-64 | 0.561765708 | 0.98 | 0.654 | 1.58E-59 |
| C06 | Pcna | 1.29E-63 | 0.563616924 | 0.918 | 0.582 | 2.90E-59 |
| C06 | Ank | 1.71E-63 | 0.617489127 | 0.962 | 0.729 | 3.85E-59 |
| C06 | Strap | 2.29E-63 | 0.521854515 | 0.966 | 0.704 | 5.17E-59 |
| C06 | Ecm1 | 3.71E-62 | 0.596068649 | 0.996 | 0.919 | 8.36E-58 |
| C06 | Tnnt2 | 9.62E-62 | 0.602636039 | 0.942 | 0.619 | 2.17E-57 |
| C06 | Dkc1 | 8.65E-61 | 0.516168902 | 0.869 | 0.517 | 1.95E-56 |
| C06 | Oaf | 8.25E-59 | 0.571328959 | 0.964 | 0.697 | 1.86E-54 |
| C06 | Cenph | 1.32E-57 | 0.575367937 | 0.867 | 0.489 | 2.98E-53 |
| C06 | Ran | 3.32E-57 | 0.515462805 | 0.99 | 0.858 | 7.50E-53 |
| C06 | Tsc22d1 | 2.87E-56 | 0.591520725 | 0.996 | 0.791 | 6.47E-52 |
| C06 | Nars | 2.48E-55 | 0.540134016 | 0.992 | 0.806 | 5.58E-51 |
| C06 | Ankrd1 | 1.83E-43 | 0.660630447 | 0.831 | 0.549 | 4.12E-39 |
| C06 | Lrrn4cl | 4.60E-43 | 0.724932297 | 0.659 | 0.394 | 1.04E-38 |
| C06 | Dqx1 | 3.03E-28 | 0.609546125 | 0.315 | 0.141 | 6.82E-24 |
| F01 | Mt1 | 3.77E-193 | 1.00830111 | 0.987 | 0.984 | 8.50E-189 |
| F01 | Tmem176b | 4.58E-158 | 0.872036274 | 0.547 | 0.302 | 1.03E-153 |
| F01 | Aldoa | 1.99E-151 | 0.720708857 | 0.941 | 0.951 | 4.50E-147 |
| F01 | Col3a1 | 3.40E-148 | 0.642767287 | 0.941 | 0.889 | 7.66E-144 |
| F01 | Mt2 | 9.15E-145 | 0.813208488 | 0.943 | 0.904 | 2.06E-140 |
| F01 | Dcn | 3.20E-139 | 0.741483809 | 0.837 | 0.713 | 7.22E-135 |
| F01 | Mmp2 | 2.29E-138 | 0.651314214 | 0.925 | 0.887 | 5.16E-134 |
| F01 | Bgn | 1.36E-125 | 0.550457852 | 0.93 | 0.891 | 3.07E-121 |
| F01 | Bnip3 | 8.04E-89 | 0.869001267 | 0.71 | 0.62 | 1.81E-84 |
| F01 | Stra6 | 1.79E-68 | 0.632600702 | 0.435 | 0.29 | 4.03E-64 |
| F01 | Sox4 | 1.01E-61 | 0.71020121 | 0.631 | 0.577 | 2.27E-57 |
| F01 | Wls | 3.98E-57 | 0.505179278 | 0.736 | 0.732 | 8.97E-53 |
| F01 | Gpnmb | 4.98E-54 | 0.570599803 | 0.561 | 0.479 | 1.12E-49 |
| F01 | Vcan | 1.64E-35 | 0.53301324 | 0.628 | 0.629 | 3.71E-31 |
| F01 | Aldh3a1 | 1.55E-34 | 0.554507835 | 0.527 | 0.464 | 3.50E-30 |
| F01 | Mmp14 | 1.16E-30 | 0.503076354 | 0.623 | 0.644 | 2.60E-26 |
| F02 | Gsn | 0 | 2.91873387 | 0.999 | 0.898 | 0 |
| F02 | Col3a1 | 0 | 2.897250442 | 1 | 0.885 | 0 |
| F02 | Dcn | 0 | 2.760602778 | 0.988 | 0.703 | 0 |
| F02 | Aspn | 0 | 2.1781504 | 0.401 | 0.021 | 0 |
| F02 | Apod | 0 | 2.086522743 | 0.729 | 0.271 | 0 |
| F02 | Ramp2 | 0 | 1.740005064 | 0.605 | 0.134 | 0 |
| F02 | Ogn | 0 | 1.721410608 | 0.396 | 0.026 | 0 |
| F02 | Ly6c1 | 0 | 1.665050455 | 0.975 | 0.82 | 0 |
| F02 | Mmp2 | 0 | 1.503387546 | 0.992 | 0.882 | 0 |
| F02 | Col6a1 | 0 | 1.490279669 | 0.969 | 0.785 | 0 |
| F02 | Islr | 0 | 1.487138531 | 0.626 | 0.148 | 0 |
| F02 | Prelp | 0 | 1.484471986 | 0.805 | 0.391 | 0 |
| F02 | Col6a2 | 0 | 1.479957692 | 0.854 | 0.595 | 0 |
| F02 | Mxra8 | 0 | 1.414710968 | 0.848 | 0.532 | 0 |
| F02 | Fbn1 | 0 | 1.40808077 | 0.725 | 0.398 | 0 |
| F02 | Sparc | 0 | 1.31362047 | 0.998 | 0.917 | 0 |
| F02 | Lbp | 0 | 1.259241617 | 0.305 | 0.043 | 0 |
| F02 | Wls | 0 | 1.096875539 | 0.894 | 0.72 | 0 |
| F02 | Lrp1 | 0 | 1.035437686 | 0.984 | 0.931 | 0 |
| F02 | Bgn | 0 | 1.003184296 | 0.982 | 0.888 | 0 |
| F02 | Laptm4a | 0 | 0.906642745 | 0.967 | 0.915 | 0 |
| F02 | Itm2b | 0 | 0.889064678 | 0.992 | 0.969 | 0 |
| F02 | Fstl1 | 4.12E-298 | 1.142550856 | 0.962 | 0.848 | 9.28E-294 |
| F02 | Ly6a | 8.25E-287 | 1.013612125 | 0.986 | 0.94 | 1.86E-282 |
| F02 | Itgb5 | 3.04E-281 | 1.178697322 | 0.752 | 0.475 | 6.85E-277 |
| F02 | Cdkn2c | 4.76E-281 | 1.165913008 | 0.698 | 0.379 | 1.07E-276 |
| F02 | Fbln2 | 3.58E-280 | 0.933552244 | 0.945 | 0.827 | 8.07E-276 |
| F02 | Cd34 | 2.89E-258 | 0.919334744 | 0.959 | 0.864 | 6.52E-254 |
| F02 | Sema3c | 3.43E-248 | 1.079102714 | 0.414 | 0.13 | 7.73E-244 |
| F02 | Col1a2 | 2.75E-236 | 1.169659794 | 0.803 | 0.611 | 6.21E-232 |
| F02 | Cavin3 | 1.07E-221 | 0.694713008 | 0.972 | 0.89 | 2.42E-217 |
| F02 | C1s1 | 3.43E-220 | 0.839596156 | 0.34 | 0.095 | 7.74E-216 |
| F02 | Pcolce | 1.43E-216 | 0.740416609 | 0.96 | 0.877 | 3.23E-212 |
| F02 | Cd248 | 9.51E-214 | 1.01952783 | 0.514 | 0.233 | 2.14E-209 |
| F02 | Matn2 | 2.39E-203 | 0.966448616 | 0.431 | 0.169 | 5.39E-199 |
| F02 | Aldh3a1 | 1.73E-193 | 1.354366471 | 0.68 | 0.452 | 3.90E-189 |
| F02 | Cd63 | 5.28E-188 | 0.565070666 | 0.988 | 0.932 | 1.19E-183 |
| F02 | Metrnl | 7.81E-184 | 0.908218838 | 0.761 | 0.583 | 1.76E-179 |
| F02 | Igfbp6 | 5.03E-180 | 0.922900209 | 0.941 | 0.856 | 1.13E-175 |
| F02 | Ddah2 | 4.68E-169 | 0.920823506 | 0.728 | 0.557 | 1.06E-164 |
| F02 | Adamts1 | 7.67E-168 | 1.153511004 | 0.537 | 0.28 | 1.73E-163 |
| F02 | Cpq | 2.31E-166 | 0.945770353 | 0.664 | 0.481 | 5.20E-162 |
| F02 | Scara5 | 1.33E-159 | 0.758477422 | 0.378 | 0.149 | 3.00E-155 |
| F02 | Mmp14 | 2.42E-155 | 0.861538577 | 0.761 | 0.633 | 5.45E-151 |
| F02 | Rnase4 | 4.45E-155 | 0.875731625 | 0.748 | 0.618 | 1.00E-150 |
| F02 | Anpep | 5.28E-152 | 0.706523095 | 0.41 | 0.173 | 1.19E-147 |
| F02 | Sdc2 | 5.24E-147 | 0.883219943 | 0.752 | 0.641 | 1.18E-142 |
| F02 | Col5a2 | 1.04E-146 | 0.820315693 | 0.415 | 0.191 | 2.34E-142 |
| F02 | Selenom | 1.54E-144 | 0.779238583 | 0.767 | 0.647 | 3.46E-140 |
| F02 | Col5a1 | 2.11E-144 | 0.834408425 | 0.489 | 0.261 | 4.76E-140 |
| F02 | Gabarap | 3.35E-143 | 0.641062445 | 0.886 | 0.845 | 7.56E-139 |
| F02 | Gstm1 | 2.57E-137 | 0.847150211 | 0.591 | 0.389 | 5.80E-133 |
| F02 | Olfml3 | 5.56E-137 | 0.761086783 | 0.386 | 0.171 | 1.25E-132 |
| F02 | App | 8.88E-137 | 0.759312007 | 0.754 | 0.632 | 2.00E-132 |
| F02 | Txnip | 2.33E-135 | 0.890230537 | 0.543 | 0.315 | 5.25E-131 |
| F02 | Nfix | 3.93E-134 | 0.757540229 | 0.743 | 0.617 | 8.86E-130 |
| F02 | Htra3 | 2.09E-133 | 1.077653272 | 0.561 | 0.357 | 4.72E-129 |
| F02 | Serinc3 | 1.26E-129 | 0.721070529 | 0.805 | 0.725 | 2.84E-125 |
| F02 | Ypel3 | 3.98E-128 | 0.763963696 | 0.548 | 0.34 | 8.96E-124 |
| F02 | Tcf4 | 6.83E-128 | 0.749723291 | 0.733 | 0.609 | 1.54E-123 |
| F02 | Fcgrt | 1.52E-126 | 0.801880288 | 0.49 | 0.281 | 3.42E-122 |
| F02 | Rabac1 | 4.34E-124 | 0.642478016 | 0.832 | 0.786 | 9.79E-120 |
| F02 | Dpysl3 | 2.01E-123 | 0.710508578 | 0.82 | 0.751 | 4.53E-119 |
| F02 | Tmem59 | 1.88E-120 | 0.574706147 | 0.879 | 0.837 | 4.24E-116 |
| F02 | Arap1 | 6.07E-119 | 0.760194421 | 0.513 | 0.311 | 1.37E-114 |
| F02 | Serpinf1 | 4.20E-116 | 1.119477162 | 0.414 | 0.213 | 9.47E-112 |
| F02 | Crip2 | 9.22E-116 | 0.57350804 | 0.939 | 0.865 | 2.08E-111 |
| F02 | Ltbp4 | 6.11E-115 | 0.678442533 | 0.856 | 0.77 | 1.38E-110 |
| F02 | Igf2r | 1.33E-113 | 0.718281412 | 0.366 | 0.174 | 2.99E-109 |
| F02 | Bmp1 | 1.84E-108 | 0.742615528 | 0.468 | 0.283 | 4.15E-104 |
| F02 | Fzd2 | 7.46E-105 | 0.726860898 | 0.586 | 0.427 | 1.68E-100 |
| F02 | Pdlim2 | 5.22E-102 | 0.711110434 | 0.689 | 0.579 | 1.18E-97 |
| F02 | Pink1 | 6.62E-100 | 0.636301452 | 0.439 | 0.257 | 1.49E-95 |
| F02 | Ddit4 | 4.87E-97 | 0.807891423 | 0.369 | 0.184 | 1.10E-92 |
| F02 | Lamp2 | 1.25E-94 | 0.609798129 | 0.786 | 0.738 | 2.82E-90 |
| F02 | Mxd4 | 1.25E-94 | 0.659780854 | 0.463 | 0.282 | 2.83E-90 |
| F02 | S100a16 | 4.49E-89 | 0.600574296 | 0.378 | 0.201 | 1.01E-84 |
| F02 | Cavin2 | 6.98E-82 | 0.809107748 | 0.648 | 0.545 | 1.57E-77 |
| F02 | Hspg2 | 8.58E-82 | 0.729704296 | 0.514 | 0.374 | 1.93E-77 |
| F02 | Sptbn1 | 4.41E-81 | 0.646776445 | 0.695 | 0.631 | 9.95E-77 |
| F02 | Mbnl1 | 1.13E-80 | 0.66798301 | 0.734 | 0.702 | 2.54E-76 |
| F02 | H2-T23 | 1.33E-80 | 0.665472246 | 0.595 | 0.451 | 3.00E-76 |
| F02 | Ebf1 | 1.63E-78 | 0.687839268 | 0.448 | 0.302 | 3.68E-74 |
| F02 | Tns2 | 3.10E-77 | 0.564033512 | 0.366 | 0.207 | 6.99E-73 |
| F02 | Smg6 | 2.32E-74 | 0.608013852 | 0.378 | 0.227 | 5.23E-70 |
| F02 | Fn1 | 8.55E-73 | 0.805012807 | 0.672 | 0.587 | 1.93E-68 |
| F02 | Cd82 | 1.87E-71 | 0.611477048 | 0.568 | 0.461 | 4.22E-67 |
| F02 | Loxl1 | 1.40E-65 | 0.60110921 | 0.393 | 0.254 | 3.16E-61 |
| F02 | Col16a1 | 4.40E-65 | 0.641961912 | 0.552 | 0.453 | 9.91E-61 |
| F02 | Col1a1 | 7.93E-65 | 1.128008628 | 0.254 | 0.122 | 1.79E-60 |
| F02 | Eva1b | 1.00E-64 | 0.612316003 | 0.572 | 0.473 | 2.26E-60 |
| F02 | Zfp36l1 | 1.22E-57 | 0.603914898 | 0.512 | 0.397 | 2.74E-53 |
| F02 | Casp12 | 8.10E-57 | 0.56525768 | 0.495 | 0.389 | 1.83E-52 |
| F02 | Entpd2 | 5.47E-55 | 0.6194633 | 0.428 | 0.317 | 1.23E-50 |
| F02 | Clec2d | 4.55E-54 | 0.562020159 | 0.445 | 0.32 | 1.03E-49 |
| F02 | Fhl2 | 1.92E-53 | 0.614998557 | 0.548 | 0.478 | 4.32E-49 |
| F02 | Col5a3 | 2.17E-51 | 0.607966622 | 0.439 | 0.331 | 4.90E-47 |
| F02 | Snhg18 | 7.02E-48 | 0.602157458 | 0.511 | 0.424 | 1.58E-43 |
| F02 | Nnmt | 1.76E-47 | 0.572050488 | 0.455 | 0.356 | 3.96E-43 |
| F02 | Hes1 | 6.90E-16 | 0.574403817 | 0.519 | 0.515 | 1.56E-11 |
| F03 | Sparc | 5.19E-204 | 0.603099186 | 0.997 | 0.917 | 1.17E-199 |
| F03 | Crabp2 | 2.15E-157 | 0.742977906 | 0.903 | 0.712 | 4.86E-153 |
| F03 | Igfbp6 | 7.02E-146 | 0.585052302 | 0.968 | 0.854 | 1.58E-141 |
| F03 | Pcolce | 2.40E-143 | 0.543159764 | 0.981 | 0.876 | 5.41E-139 |
| F03 | Mgst3 | 2.82E-141 | 0.610258605 | 0.943 | 0.81 | 6.36E-137 |
| F03 | Fstl1 | 3.94E-137 | 0.642794582 | 0.974 | 0.848 | 8.88E-133 |
| F03 | Ifitm1 | 8.66E-103 | 0.581641713 | 0.954 | 0.846 | 1.95E-98 |
| F03 | Phlda3 | 3.02E-94 | 0.556404184 | 0.847 | 0.703 | 6.81E-90 |
| F03 | Ly6c1 | 3.14E-93 | 0.508429317 | 0.947 | 0.823 | 7.08E-89 |
| F03 | Timp3 | 1.13E-72 | 0.643506173 | 0.7 | 0.538 | 2.54E-68 |
| F03 | Slurp1 | 2.80E-59 | 0.947565486 | 0.586 | 0.421 | 6.30E-55 |
| F04 | Hspa9 | 1.89E-178 | 0.775636078 | 0.975 | 0.817 | 4.27E-174 |
| F04 | Gas5 | 2.18E-141 | 0.525216947 | 1 | 0.984 | 4.92E-137 |
| F04 | Nars | 7.93E-132 | 0.707538661 | 0.953 | 0.802 | 1.79E-127 |
| F04 | Ghitm | 1.06E-121 | 0.607702708 | 0.953 | 0.813 | 2.38E-117 |
| F04 | 2410006H16Rik | 4.92E-114 | 0.573459381 | 0.993 | 0.949 | 1.11E-109 |
| F04 | Asns | 3.93E-112 | 0.62117811 | 0.784 | 0.513 | 8.85E-108 |
| F04 | Sqstm1 | 1.65E-106 | 0.59419157 | 0.989 | 0.912 | 3.72E-102 |
| F04 | Srm | 3.45E-105 | 0.610722759 | 0.846 | 0.605 | 7.78E-101 |
| F04 | Hspd1 | 1.37E-104 | 0.527267313 | 0.981 | 0.837 | 3.10E-100 |
| F04 | Gnl3 | 1.53E-99 | 0.547566172 | 0.835 | 0.593 | 3.44E-95 |
| F04 | Atf5 | 9.43E-96 | 0.568602016 | 0.789 | 0.538 | 2.13E-91 |
| F04 | Trib3 | 1.01E-92 | 0.565175512 | 0.401 | 0.182 | 2.28E-88 |
| F04 | Eif4ebp1 | 2.59E-89 | 0.518811341 | 0.982 | 0.902 | 5.84E-85 |
| F04 | Tars | 2.98E-89 | 0.573896365 | 0.781 | 0.566 | 6.72E-85 |
| F04 | Sars | 4.62E-82 | 0.500738711 | 0.855 | 0.677 | 1.04E-77 |
| F04 | Cited2 | 1.09E-80 | 0.556114163 | 0.868 | 0.71 | 2.45E-76 |
| F04 | Hist1h2bc | 5.73E-76 | 1.099455702 | 0.931 | 0.845 | 1.29E-71 |
| F04 | Eprs | 2.10E-75 | 0.500369221 | 0.92 | 0.766 | 4.72E-71 |
| F04 | Aars | 3.20E-75 | 0.516904142 | 0.851 | 0.664 | 7.22E-71 |
| F04 | Snhg3 | 4.71E-73 | 0.58497803 | 0.816 | 0.625 | 1.06E-68 |
| F04 | Mir5136 | 6.98E-72 | 0.569442834 | 0.58 | 0.367 | 1.57E-67 |
| F04 | Hist1h1c | 1.96E-69 | 1.149656912 | 0.858 | 0.744 | 4.42E-65 |
| F04 | Ddit3 | 5.61E-66 | 0.52162511 | 0.629 | 0.399 | 1.26E-61 |
| F04 | Timp3 | 1.55E-64 | 0.619171834 | 0.734 | 0.538 | 3.50E-60 |
| F04 | Hspa5 | 1.35E-63 | 0.685150328 | 0.991 | 0.944 | 3.04E-59 |
| F04 | Tinagl1 | 1.34E-54 | 0.527677422 | 0.743 | 0.57 | 3.02E-50 |
| F04 | Lrrn4cl | 1.25E-46 | 0.547598194 | 0.566 | 0.391 | 2.81E-42 |
| F04 | Ankrd1 | 3.49E-36 | 0.529221888 | 0.701 | 0.547 | 7.86E-32 |
| F04 | Cryab | 2.55E-17 | 0.618980399 | 0.563 | 0.472 | 5.76E-13 |
| F05 | Fos | 0 | 2.746380838 | 0.98 | 0.418 | 0 |
| F05 | Cyr61 | 0 | 2.487642757 | 0.888 | 0.482 | 0 |
| F05 | Egr1 | 0 | 2.461326222 | 0.913 | 0.296 | 0 |
| F05 | Ier2 | 0 | 2.26078289 | 0.966 | 0.615 | 0 |
| F05 | Fosb | 0 | 2.07888304 | 0.873 | 0.211 | 0 |
| F05 | Atf3 | 0 | 2.056241259 | 0.892 | 0.316 | 0 |
| F05 | Dnajb1 | 0 | 2.029730296 | 0.899 | 0.543 | 0 |
| F05 | Jun | 0 | 1.934866565 | 0.999 | 0.927 | 0 |
| F05 | Klf2 | 0 | 1.886755809 | 0.996 | 0.922 | 0 |
| F05 | Dusp1 | 0 | 1.778837222 | 0.965 | 0.541 | 0 |
| F05 | Socs3 | 0 | 1.701918985 | 0.882 | 0.41 | 0 |
| F05 | Junb | 0 | 1.677826652 | 0.94 | 0.6 | 0 |
| F05 | Zfp36 | 0 | 1.62229529 | 0.895 | 0.382 | 0 |
| F05 | Jund | 0 | 1.482575671 | 0.998 | 0.92 | 0 |
| F05 | Ubc | 0 | 1.381666998 | 0.997 | 0.931 | 0 |
| F05 | Klf6 | 4.03E-293 | 1.401681153 | 0.978 | 0.839 | 9.09E-289 |
| F05 | Nr4a1 | 9.41E-236 | 1.138166706 | 0.455 | 0.118 | 2.12E-231 |
| F05 | Btg2 | 5.43E-228 | 0.574293702 | 0.407 | 0.089 | 1.22E-223 |
| F05 | Klf4 | 1.57E-203 | 1.010963125 | 0.949 | 0.753 | 3.55E-199 |
| F05 | Tcim | 7.24E-202 | 1.135705577 | 0.762 | 0.4 | 1.63E-197 |
| F05 | Cxcl1 | 1.96E-196 | 1.69215017 | 0.353 | 0.085 | 4.42E-192 |
| F05 | Rhob | 3.48E-186 | 0.937405304 | 0.888 | 0.626 | 7.84E-182 |
| F05 | Sgk1 | 3.26E-178 | 1.330169419 | 0.695 | 0.361 | 7.34E-174 |
| F05 | Ubb | 1.11E-175 | 0.530789826 | 1 | 0.997 | 2.51E-171 |
| F05 | Ppp1r15a | 2.84E-155 | 0.735402104 | 0.704 | 0.36 | 6.40E-151 |
| F05 | Ier3 | 1.84E-152 | 0.946871943 | 0.703 | 0.362 | 4.14E-148 |
| F05 | Has1 | 3.62E-152 | 1.094619928 | 0.784 | 0.503 | 8.15E-148 |
| F05 | Trib1 | 1.02E-128 | 0.545612075 | 0.443 | 0.167 | 2.31E-124 |
| F05 | Serpine1 | 6.53E-127 | 0.506241911 | 0.266 | 0.069 | 1.47E-122 |
| F05 | Hspa1b | 8.95E-122 | 0.773480509 | 0.659 | 0.358 | 2.02E-117 |
| F05 | Clk1 | 1.05E-118 | 0.65753391 | 0.783 | 0.521 | 2.36E-114 |
| F05 | Myc | 1.49E-118 | 0.879844457 | 0.794 | 0.558 | 3.35E-114 |
| F05 | Phlda1 | 1.11E-115 | 1.021906968 | 0.684 | 0.416 | 2.51E-111 |
| F05 | Errfi1 | 7.57E-102 | 0.870345103 | 0.559 | 0.306 | 1.71E-97 |
| F05 | Adamts1 | 2.49E-101 | 0.947294558 | 0.544 | 0.286 | 5.61E-97 |
| F05 | Nfkbia | 2.34E-100 | 1.18338823 | 0.864 | 0.718 | 5.28E-96 |
| F05 | Hes1 | 9.78E-97 | 1.243582351 | 0.727 | 0.505 | 2.20E-92 |
| F05 | Dusp6 | 2.88E-92 | 0.633730368 | 0.551 | 0.299 | 6.50E-88 |
| F05 | Nupr1 | 2.93E-81 | 0.676182461 | 0.989 | 0.914 | 6.60E-77 |
| F05 | Hist1h2bc | 1.35E-71 | 0.63961865 | 0.943 | 0.845 | 3.04E-67 |
| F05 | Hsp90aa1 | 1.53E-68 | 0.566038423 | 0.968 | 0.876 | 3.46E-64 |
| F05 | Tsc22d1 | 3.73E-56 | 0.533432897 | 0.923 | 0.79 | 8.40E-52 |
| F05 | Sox9 | 6.21E-54 | 0.521724993 | 0.846 | 0.689 | 1.40E-49 |
| F05 | Cited2 | 2.58E-52 | 0.669807162 | 0.836 | 0.713 | 5.82E-48 |
| F05 | Ackr3 | 8.53E-40 | 0.587647928 | 0.699 | 0.57 | 1.92E-35 |
| F05 | Plk2 | 1.76E-38 | 0.604418353 | 0.608 | 0.467 | 3.96E-34 |
| F05 | Nppb | 1.62E-08 | 0.659946004 | 0.309 | 0.255 | 0.000365116 |
| F06 | Hist1h1c | 1.45E-203 | 1.556564481 | 0.931 | 0.741 | 3.27E-199 |
| F06 | Nupr1 | 4.13E-166 | 1.134770424 | 0.989 | 0.914 | 9.30E-162 |
| F06 | Calr | 5.64E-160 | 0.970808996 | 0.988 | 0.944 | 1.27E-155 |
| F06 | Hist1h2bc | 1.47E-154 | 1.117848299 | 0.953 | 0.845 | 3.31E-150 |
| F06 | Gas5 | 7.74E-141 | 0.604000657 | 0.999 | 0.984 | 1.75E-136 |
| F06 | 2410006H16Rik | 6.23E-129 | 0.728810052 | 0.993 | 0.949 | 1.41E-124 |
| F06 | Sqstm1 | 3.40E-128 | 0.73771519 | 0.988 | 0.913 | 7.67E-124 |
| F06 | Zfas1 | 1.20E-126 | 0.837688995 | 0.941 | 0.838 | 2.69E-122 |
| F06 | Mt2 | 1.59E-124 | 0.944978798 | 0.983 | 0.903 | 3.58E-120 |
| F06 | Mt1 | 3.31E-121 | 0.965561325 | 0.999 | 0.984 | 7.46E-117 |
| F06 | Eef1b2 | 7.10E-120 | 0.544800093 | 0.99 | 0.963 | 1.60E-115 |
| F06 | Cryab | 2.53E-115 | 1.385107432 | 0.708 | 0.467 | 5.71E-111 |
| F06 | Ddit3 | 2.60E-114 | 0.98308414 | 0.675 | 0.399 | 5.86E-110 |
| F06 | 1110038B12Rik | 5.92E-112 | 0.750617742 | 0.929 | 0.808 | 1.33E-107 |
| F06 | Timp3 | 1.16E-102 | 0.921351205 | 0.77 | 0.539 | 2.61E-98 |
| F06 | Gpnmb | 2.40E-96 | 0.735669639 | 0.716 | 0.475 | 5.40E-92 |
| F06 | Snhg6 | 8.74E-78 | 0.58017551 | 0.943 | 0.856 | 1.97E-73 |
| F06 | Igfbp6 | 3.12E-76 | 0.6678299 | 0.938 | 0.859 | 7.04E-72 |
| F06 | Eif4a2 | 3.29E-69 | 0.569162329 | 0.795 | 0.671 | 7.42E-65 |
| F06 | Ackr3 | 4.37E-69 | 0.731104719 | 0.719 | 0.57 | 9.86E-65 |
| F06 | Atf5 | 2.76E-68 | 0.608364256 | 0.729 | 0.544 | 6.22E-64 |
| F06 | H1f0 | 1.65E-67 | 0.524366029 | 0.995 | 0.964 | 3.72E-63 |
| F06 | Jund | 2.55E-67 | 0.569579425 | 0.965 | 0.921 | 5.76E-63 |
| F06 | Hspa5 | 1.06E-63 | 0.680463041 | 0.989 | 0.944 | 2.40E-59 |
| F06 | Klf4 | 1.58E-59 | 0.673097105 | 0.845 | 0.758 | 3.57E-55 |
| F06 | Hspe1 | 1.47E-55 | 0.565334101 | 0.968 | 0.91 | 3.31E-51 |
| F06 | Klf3 | 1.22E-45 | 0.548658109 | 0.699 | 0.597 | 2.75E-41 |
| F06 | Hsph1 | 7.64E-27 | 0.592939261 | 0.405 | 0.293 | 1.72E-22 |
| F06 | Bst2 | 1.67E-20 | 0.509624969 | 0.801 | 0.773 | 3.77E-16 |
| F06 | Slurp1 | 2.48E-07 | 0.612327915 | 0.479 | 0.43 | 0.005597195 |
| Cd14+ Monocytes | Cxcl2 | 0 | 4.582225631 | 0.759 | 0.067 | 0 |
| Cd14+ Monocytes | Rgs1 | 0 | 4.42422853 | 0.91 | 0.139 | 0 |
| Cd14+ Monocytes | Cd74 | 0 | 4.309234079 | 0.987 | 0.582 | 0 |
| Cd14+ Monocytes | H2-Aa | 0 | 3.96656534 | 0.96 | 0.343 | 0 |
| Cd14+ Monocytes | Cd14 | 0 | 3.925171256 | 0.95 | 0.177 | 0 |
| Cd14+ Monocytes | Lyz2 | 0 | 3.881920482 | 0.994 | 0.32 | 0 |
| Cd14+ Monocytes | H2-Eb1 | 0 | 3.852048582 | 0.946 | 0.316 | 0 |
| Cd14+ Monocytes | Zfp36 | 0 | 3.83918312 | 0.987 | 0.392 | 0 |
| Cd14+ Monocytes | H2-Ab1 | 0 | 3.822673171 | 0.956 | 0.312 | 0 |
| Cd14+ Monocytes | Hspa1a | 0 | 3.718571425 | 0.852 | 0.065 | 0 |
| Cd14+ Monocytes | Ccl4 | 0 | 3.490516179 | 0.519 | 0.072 | 0 |
| Cd14+ Monocytes | Atf3 | 0 | 3.482848509 | 0.956 | 0.329 | 0 |
| Cd14+ Monocytes | Ctss | 0 | 3.459520386 | 0.99 | 0.136 | 0 |
| Cd14+ Monocytes | C1qb | 0 | 3.439735075 | 0.877 | 0.108 | 0 |
| Cd14+ Monocytes | Fcer1g | 0 | 3.413984385 | 0.983 | 0.107 | 0 |
| Cd14+ Monocytes | Tyrobp | 0 | 3.385379415 | 0.983 | 0.11 | 0 |
| Cd14+ Monocytes | Apoe | 0 | 3.3222748 | 0.826 | 0.19 | 0 |
| Cd14+ Monocytes | Dusp1 | 0 | 3.312370369 | 0.989 | 0.551 | 0 |
| Cd14+ Monocytes | Ctsc | 0 | 3.171688377 | 0.956 | 0.077 | 0 |
| Cd14+ Monocytes | C1qc | 0 | 3.146126875 | 0.828 | 0.088 | 0 |
| Cd14+ Monocytes | Ccl6 | 0 | 3.020941292 | 0.833 | 0.076 | 0 |
| Cd14+ Monocytes | Ccl3 | 0 | 3.009119911 | 0.611 | 0.041 | 0 |
| Cd14+ Monocytes | H2-DMa | 0 | 2.985720652 | 0.92 | 0.211 | 0 |
| Cd14+ Monocytes | Fcgr3 | 0 | 2.943909437 | 0.958 | 0.077 | 0 |
| Cd14+ Monocytes | Arg1 | 0 | 2.933199907 | 0.693 | 0.069 | 0 |
| Cd14+ Monocytes | C1qa | 0 | 2.907005319 | 0.807 | 0.072 | 0 |
| Cd14+ Monocytes | Srgn | 0 | 2.893720901 | 0.916 | 0.093 | 0 |
| Cd14+ Monocytes | Fcgr2b | 0 | 2.868138684 | 0.929 | 0.087 | 0 |
| Cd14+ Monocytes | Cd83 | 0 | 2.854108532 | 0.697 | 0.025 | 0 |
| Cd14+ Monocytes | Alox5ap | 0 | 2.850703932 | 0.923 | 0.074 | 0 |
| Cd14+ Monocytes | Cd52 | 0 | 2.753303471 | 0.969 | 0.113 | 0 |
| Cd14+ Monocytes | Ccrl2 | 0 | 2.724367703 | 0.661 | 0.028 | 0 |
| Cd14+ Monocytes | Rgs2 | 0 | 2.699330789 | 0.761 | 0.108 | 0 |
| Cd14+ Monocytes | Id2 | 0 | 2.672144427 | 0.753 | 0.118 | 0 |
| Cd14+ Monocytes | Ucp2 | 0 | 2.671527848 | 0.895 | 0.103 | 0 |
| Cd14+ Monocytes | Mafb | 0 | 2.671509227 | 0.751 | 0.04 | 0 |
| Cd14+ Monocytes | Clec4n | 0 | 2.649234223 | 0.816 | 0.05 | 0 |
| Cd14+ Monocytes | Mmp12 | 0 | 2.585359583 | 0.579 | 0.049 | 0 |
| Cd14+ Monocytes | Lilr4b | 0 | 2.537160501 | 0.849 | 0.063 | 0 |
| Cd14+ Monocytes | Laptm5 | 0 | 2.497695875 | 0.914 | 0.075 | 0 |
| Cd14+ Monocytes | Il1b | 0 | 2.434471808 | 0.412 | 0.016 | 0 |
| Cd14+ Monocytes | Coro1a | 0 | 2.432084531 | 0.916 | 0.091 | 0 |
| Cd14+ Monocytes | Cfp | 0 | 2.415391876 | 0.841 | 0.059 | 0 |
| Cd14+ Monocytes | Cd68 | 0 | 2.400725128 | 0.879 | 0.08 | 0 |
| Cd14+ Monocytes | Btg2 | 0 | 2.378983395 | 0.774 | 0.088 | 0 |
| Cd14+ Monocytes | Osm | 0 | 2.371063381 | 0.492 | 0.016 | 0 |
| Cd14+ Monocytes | Pld4 | 0 | 2.340592448 | 0.847 | 0.048 | 0 |
| Cd14+ Monocytes | Sirpb1c | 0 | 2.338475571 | 0.828 | 0.041 | 0 |
| Cd14+ Monocytes | Cxcl16 | 0 | 2.305740411 | 0.789 | 0.133 | 0 |
| Cd14+ Monocytes | Ly86 | 0 | 2.304170388 | 0.81 | 0.049 | 0 |
| Cd14+ Monocytes | Ms4a6c | 0 | 2.276024685 | 0.828 | 0.045 | 0 |
| Cd14+ Monocytes | Lcp1 | 0 | 2.264612926 | 0.837 | 0.07 | 0 |
| Cd14+ Monocytes | C3ar1 | 0 | 2.260346875 | 0.791 | 0.066 | 0 |
| Cd14+ Monocytes | Ccr2 | 0 | 2.241187131 | 0.77 | 0.036 | 0 |
| Cd14+ Monocytes | Csf2ra | 0 | 2.225219667 | 0.799 | 0.046 | 0 |
| Cd14+ Monocytes | Emb | 0 | 2.152463064 | 0.818 | 0.102 | 0 |
| Cd14+ Monocytes | Slfn2 | 0 | 2.14318226 | 0.789 | 0.069 | 0 |
| Cd14+ Monocytes | Spi1 | 0 | 2.081739544 | 0.812 | 0.046 | 0 |
| Cd14+ Monocytes | Trem2 | 0 | 2.078838857 | 0.789 | 0.047 | 0 |
| Cd14+ Monocytes | Ccr1 | 0 | 2.049834388 | 0.762 | 0.042 | 0 |
| Cd14+ Monocytes | Hcar2 | 0 | 2.031231086 | 0.511 | 0.018 | 0 |
| Cd14+ Monocytes | Clec4a2 | 0 | 1.995778499 | 0.713 | 0.035 | 0 |
| Cd14+ Monocytes | Gm2a | 0 | 1.990921695 | 0.766 | 0.108 | 0 |
| Cd14+ Monocytes | Msr1 | 0 | 1.964854859 | 0.688 | 0.042 | 0 |
| Cd14+ Monocytes | AW112010 | 0 | 1.962883372 | 0.69 | 0.113 | 0 |
| Cd14+ Monocytes | Ccl9 | 0 | 1.937360304 | 0.678 | 0.081 | 0 |
| Cd14+ Monocytes | Cd300c2 | 0 | 1.920566583 | 0.709 | 0.04 | 0 |
| Cd14+ Monocytes | Clec7a | 0 | 1.88686193 | 0.592 | 0.026 | 0 |
| Cd14+ Monocytes | Itgb2 | 0 | 1.848627416 | 0.736 | 0.063 | 0 |
| Cd14+ Monocytes | Pycard | 0 | 1.836820304 | 0.697 | 0.043 | 0 |
| Cd14+ Monocytes | Pf4 | 0 | 1.81309061 | 0.414 | 0.043 | 0 |
| Cd14+ Monocytes | Aif1 | 0 | 1.811780063 | 0.626 | 0.034 | 0 |
| Cd14+ Monocytes | Plbd1 | 0 | 1.801942876 | 0.665 | 0.031 | 0 |
| Cd14+ Monocytes | Ms4a6d | 0 | 1.79510385 | 0.676 | 0.039 | 0 |
| Cd14+ Monocytes | Cd53 | 0 | 1.763590871 | 0.72 | 0.057 | 0 |
| Cd14+ Monocytes | Plek | 0 | 1.759823488 | 0.628 | 0.035 | 0 |
| Cd14+ Monocytes | Unc93b1 | 0 | 1.754575425 | 0.738 | 0.115 | 0 |
| Cd14+ Monocytes | Csf1r | 0 | 1.753390551 | 0.663 | 0.04 | 0 |
| Cd14+ Monocytes | Fos | 1.51E-300 | 2.866162489 | 0.977 | 0.432 | 3.41E-296 |
| Cd14+ Monocytes | Tmsb4x | 3.08E-284 | 2.563501198 | 1 | 0.983 | 6.94E-280 |
| Cd14+ Monocytes | Fth1 | 1.73E-272 | 2.024050584 | 1 | 0.992 | 3.90E-268 |
| Cd14+ Monocytes | Cebpb | 3.41E-255 | 2.821756525 | 0.96 | 0.702 | 7.69E-251 |
| Cd14+ Monocytes | Cyba | 6.07E-251 | 2.102696544 | 0.967 | 0.82 | 1.37E-246 |
| Cd14+ Monocytes | Fxyd5 | 2.19E-244 | 1.884522355 | 0.981 | 0.898 | 4.94E-240 |
| Cd14+ Monocytes | Junb | 3.39E-233 | 2.625119616 | 0.944 | 0.609 | 7.63E-229 |
| Cd14+ Monocytes | Tgfbi | 1.06E-229 | 2.122333657 | 0.96 | 0.757 | 2.40E-225 |
| Cd14+ Monocytes | Lsp1 | 4.19E-201 | 1.762416539 | 0.851 | 0.425 | 9.45E-197 |
| Cd14+ Monocytes | Ctsh | 4.21E-190 | 1.759051419 | 0.824 | 0.428 | 9.48E-186 |
| Cd14+ Monocytes | H2-DMb1 | 3.99E-184 | 1.829201391 | 0.818 | 0.431 | 9.00E-180 |
| Cd14+ Monocytes | Prdx5 | 1.92E-176 | 1.862013061 | 0.872 | 0.621 | 4.32E-172 |
| Cd14+ Monocytes | AA467197 | 4.65E-153 | 1.788709524 | 0.738 | 0.365 | 1.05E-148 |
| Cd14+ Monocytes | Cdkn1a | 3.66E-132 | 1.837649197 | 0.793 | 0.488 | 8.24E-128 |
| Cd14+ Monocytes | Ccl2 | 2.37E-113 | 3.102305255 | 0.784 | 0.576 | 5.34E-109 |
| Cd14+ Monocytes | Ier3 | 2.24E-106 | 2.306165756 | 0.68 | 0.371 | 5.06E-102 |
| Cd14+ Monocytes | Plaur | 7.70E-106 | 1.758661633 | 0.762 | 0.543 | 1.74E-101 |
| Cd14+ Monocytes | Socs3 | 1.18E-103 | 1.929824153 | 0.711 | 0.426 | 2.65E-99 |
| Cd14+ Monocytes | Sgk1 | 1.56E-102 | 1.886514318 | 0.665 | 0.37 | 3.52E-98 |
| Cd14+ Monocytes | Spp1 | 3.98E-68 | 2.141049287 | 0.762 | 0.637 | 8.96E-64 |
| Cd14+ Monocytes | Plk2 | 1.57E-62 | 1.856861753 | 0.648 | 0.47 | 3.55E-58 |
| Cd14+ Monocytes | Ccl7 | 1.57E-07 | 2.870091006 | 0.284 | 0.236 | 0.00354143 |
| Macrophages(M1 like) | Cd74 | 0 | 4.093889908 | 0.965 | 0.578 | 0 |
| Macrophages(M1 like) | H2-Ab1 | 0 | 4.068118524 | 0.869 | 0.309 | 0 |
| Macrophages(M1 like) | Apoe | 0 | 4.031981738 | 0.716 | 0.187 | 0 |
| Macrophages(M1 like) | H2-Eb1 | 0 | 3.911113429 | 0.856 | 0.313 | 0 |
| Macrophages(M1 like) | Lyz2 | 0 | 3.845960918 | 0.882 | 0.317 | 0 |
| Macrophages(M1 like) | H2-Aa | 0 | 3.8050607 | 0.857 | 0.34 | 0 |
| Macrophages(M1 like) | C1qb | 0 | 3.703384606 | 0.751 | 0.105 | 0 |
| Macrophages(M1 like) | Ctss | 0 | 3.636584945 | 0.887 | 0.131 | 0 |
| Macrophages(M1 like) | C1qc | 0 | 3.539466139 | 0.682 | 0.086 | 0 |
| Macrophages(M1 like) | Fcer1g | 0 | 3.48502299 | 0.841 | 0.102 | 0 |
| Macrophages(M1 like) | Tyrobp | 0 | 3.454376 | 0.835 | 0.106 | 0 |
| Macrophages(M1 like) | C1qa | 0 | 3.292444743 | 0.631 | 0.071 | 0 |
| Macrophages(M1 like) | Cd52 | 0 | 3.027566702 | 0.837 | 0.109 | 0 |
| Macrophages(M1 like) | Fcgr3 | 0 | 2.95202464 | 0.699 | 0.077 | 0 |
| Macrophages(M1 like) | Ctsc | 0 | 2.77591719 | 0.688 | 0.078 | 0 |
| Macrophages(M1 like) | Fcgr2b | 0 | 2.774301338 | 0.69 | 0.087 | 0 |
| Macrophages(M1 like) | Alox5ap | 0 | 2.683537247 | 0.691 | 0.073 | 0 |
| Macrophages(M1 like) | Tmsb4x | 0 | 2.601145107 | 0.978 | 0.984 | 0 |
| Macrophages(M1 like) | Ly86 | 0 | 2.50200363 | 0.593 | 0.049 | 0 |
| Macrophages(M1 like) | Ccl6 | 0 | 2.42280967 | 0.569 | 0.078 | 0 |
| Macrophages(M1 like) | Cd68 | 0 | 2.386201485 | 0.619 | 0.081 | 0 |
| Macrophages(M1 like) | Arg1 | 0 | 2.373121571 | 0.476 | 0.071 | 0 |
| Macrophages(M1 like) | Laptm5 | 0 | 2.355518475 | 0.656 | 0.075 | 0 |
| Macrophages(M1 like) | Ms4a6c | 0 | 2.351203679 | 0.569 | 0.046 | 0 |
| Macrophages(M1 like) | Ucp2 | 0 | 2.335882646 | 0.634 | 0.104 | 0 |
| Macrophages(M1 like) | Cfp | 0 | 2.316319184 | 0.571 | 0.061 | 0 |
| Macrophages(M1 like) | Pld4 | 0 | 2.281131749 | 0.57 | 0.05 | 0 |
| Macrophages(M1 like) | Coro1a | 0 | 2.236577148 | 0.623 | 0.093 | 0 |
| Macrophages(M1 like) | Srgn | 0 | 2.179645159 | 0.656 | 0.093 | 0 |
| Macrophages(M1 like) | Ccr2 | 0 | 2.151392743 | 0.456 | 0.039 | 0 |
| Macrophages(M1 like) | Spi1 | 0 | 2.135576001 | 0.554 | 0.047 | 0 |
| Macrophages(M1 like) | Aif1 | 0 | 2.12544952 | 0.487 | 0.033 | 0 |
| Macrophages(M1 like) | Clec4n | 0 | 2.066906887 | 0.49 | 0.053 | 0 |
| Macrophages(M1 like) | Trem2 | 0 | 1.981214475 | 0.495 | 0.05 | 0 |
| Macrophages(M1 like) | Csf2ra | 0 | 1.943424715 | 0.503 | 0.049 | 0 |
| Macrophages(M1 like) | Lcp1 | 0 | 1.917519302 | 0.551 | 0.073 | 0 |
| Macrophages(M1 like) | Lilr4b | 0 | 1.908624699 | 0.502 | 0.068 | 0 |
| Macrophages(M1 like) | Csf1r | 0 | 1.849210794 | 0.46 | 0.041 | 0 |
| Macrophages(M1 like) | Itgb2 | 0 | 1.835629351 | 0.493 | 0.065 | 0 |
| Macrophages(M1 like) | Cd300c2 | 0 | 1.828210942 | 0.476 | 0.041 | 0 |
| Macrophages(M1 like) | Ccr1 | 0 | 1.820776682 | 0.457 | 0.045 | 0 |
| Macrophages(M1 like) | Plbd1 | 0 | 1.806277716 | 0.423 | 0.033 | 0 |
| Macrophages(M1 like) | Msr1 | 0 | 1.802070393 | 0.434 | 0.044 | 0 |
| Macrophages(M1 like) | Sirpb1c | 0 | 1.789271694 | 0.487 | 0.045 | 0 |
| Macrophages(M1 like) | Ms4a6d | 0 | 1.757454051 | 0.454 | 0.041 | 0 |
| Macrophages(M1 like) | Pycard | 0 | 1.732371852 | 0.45 | 0.045 | 0 |
| Macrophages(M1 like) | Slfn2 | 0 | 1.707312518 | 0.465 | 0.073 | 0 |
| Macrophages(M1 like) | Mgl2 | 0 | 1.632340685 | 0.267 | 0.02 | 0 |
| Macrophages(M1 like) | Napsa | 0 | 1.626902769 | 0.367 | 0.03 | 0 |
| Macrophages(M1 like) | Cd53 | 0 | 1.592746168 | 0.45 | 0.06 | 0 |
| Macrophages(M1 like) | Clec4a2 | 0 | 1.581867713 | 0.405 | 0.039 | 0 |
| Macrophages(M1 like) | Mpeg1 | 0 | 1.54936969 | 0.376 | 0.038 | 0 |
| Macrophages(M1 like) | Fermt3 | 0 | 1.535584345 | 0.411 | 0.044 | 0 |
| Macrophages(M1 like) | Ptprc | 0 | 1.52225068 | 0.463 | 0.065 | 0 |
| Macrophages(M1 like) | Selplg | 0 | 1.521856368 | 0.401 | 0.046 | 0 |
| Macrophages(M1 like) | Fcgr1 | 0 | 1.511235973 | 0.362 | 0.034 | 0 |
| Macrophages(M1 like) | Bcl2a1b | 0 | 1.465278162 | 0.399 | 0.041 | 0 |
| Macrophages(M1 like) | Lat2 | 0 | 1.44872301 | 0.378 | 0.046 | 0 |
| Macrophages(M1 like) | Cyth4 | 0 | 1.441661123 | 0.382 | 0.042 | 0 |
| Macrophages(M1 like) | Gmfg | 0 | 1.437458087 | 0.392 | 0.043 | 0 |
| Macrophages(M1 like) | Cybb | 0 | 1.412558453 | 0.366 | 0.039 | 0 |
| Macrophages(M1 like) | Pilra | 0 | 1.386004235 | 0.351 | 0.034 | 0 |
| Macrophages(M1 like) | Bcl2a1d | 0 | 1.350790262 | 0.332 | 0.031 | 0 |
| Macrophages(M1 like) | Clec5a | 0 | 1.342710554 | 0.306 | 0.03 | 0 |
| Macrophages(M1 like) | Cxcl16 | 3.61E-306 | 2.197444755 | 0.577 | 0.134 | 8.14E-302 |
| Macrophages(M1 like) | H2-DMa | 5.80E-294 | 2.784064921 | 0.678 | 0.212 | 1.31E-289 |
| Macrophages(M1 like) | C3ar1 | 8.26E-290 | 1.717098113 | 0.435 | 0.071 | 1.86E-285 |
| Macrophages(M1 like) | Wfdc17 | 1.85E-282 | 1.545825771 | 0.318 | 0.037 | 4.17E-278 |
| Macrophages(M1 like) | Pf4 | 5.42E-276 | 2.545771636 | 0.332 | 0.042 | 1.22E-271 |
| Macrophages(M1 like) | Mafb | 2.34E-250 | 1.630740319 | 0.337 | 0.047 | 5.28E-246 |
| Macrophages(M1 like) | Ccl9 | 2.43E-244 | 1.711530774 | 0.431 | 0.083 | 5.48E-240 |
| Macrophages(M1 like) | Fth1 | 2.39E-238 | 1.712688928 | 0.973 | 0.993 | 5.38E-234 |
| Macrophages(M1 like) | AW112010 | 5.16E-236 | 2.014853819 | 0.497 | 0.114 | 1.16E-231 |
| Macrophages(M1 like) | Emb | 1.39E-223 | 1.682448259 | 0.465 | 0.107 | 3.14E-219 |
| Macrophages(M1 like) | Unc93b1 | 7.49E-212 | 1.70837125 | 0.469 | 0.118 | 1.69E-207 |
| Macrophages(M1 like) | Selenop | 3.10E-211 | 1.437574447 | 0.306 | 0.047 | 6.98E-207 |
| Macrophages(M1 like) | Hspa1a | 1.23E-210 | 2.301349078 | 0.385 | 0.073 | 2.76E-206 |
| Macrophages(M1 like) | Cst3 | 6.84E-203 | 2.433061762 | 0.859 | 0.926 | 1.54E-198 |
| Macrophages(M1 like) | Gm2a | 9.12E-200 | 1.61347382 | 0.45 | 0.112 | 2.06E-195 |
| Macrophages(M1 like) | Fxyd5 | 6.28E-190 | 1.738926651 | 0.86 | 0.902 | 1.42E-185 |
| Macrophages(M1 like) | Malat1 | 2.61E-166 | 2.667778629 | 0.937 | 0.918 | 5.89E-162 |
| Macrophages(M1 like) | Ifi30 | 7.92E-155 | 1.920676811 | 0.566 | 0.245 | 1.78E-150 |
| Macrophages(M1 like) | Cyba | 2.02E-142 | 2.058836149 | 0.778 | 0.825 | 4.55E-138 |
| Macrophages(M1 like) | Rgs1 | 1.05E-133 | 1.658870684 | 0.454 | 0.148 | 2.36E-129 |
| Macrophages(M1 like) | Cd14 | 2.01E-130 | 1.624358497 | 0.491 | 0.186 | 4.53E-126 |
| Macrophages(M1 like) | Rgs10 | 1.75E-125 | 1.371441817 | 0.382 | 0.12 | 3.96E-121 |
| Macrophages(M1 like) | Mmp12 | 1.81E-125 | 1.469827754 | 0.264 | 0.054 | 4.08E-121 |
| Macrophages(M1 like) | Id2 | 2.50E-123 | 1.447448327 | 0.405 | 0.123 | 5.63E-119 |
| Macrophages(M1 like) | Tgfbi | 4.52E-87 | 1.836891116 | 0.72 | 0.763 | 1.02E-82 |
| Macrophages(M1 like) | H2-DMb1 | 1.17E-74 | 1.850723022 | 0.573 | 0.436 | 2.63E-70 |
| Macrophages(M1 like) | Lsp1 | 1.12E-72 | 1.666054938 | 0.581 | 0.43 | 2.54E-68 |
| Macrophages(M1 like) | Lgmn | 5.04E-71 | 2.018499405 | 0.622 | 0.57 | 1.14E-66 |
| Macrophages(M1 like) | Prdx5 | 4.02E-68 | 1.606565308 | 0.648 | 0.627 | 9.06E-64 |
| Macrophages(M1 like) | Ctsh | 5.16E-56 | 1.458794336 | 0.541 | 0.434 | 1.16E-51 |
| Macrophages(M1 like) | Ptpn18 | 2.23E-55 | 1.35767591 | 0.45 | 0.284 | 5.02E-51 |
| Macrophages(M1 like) | AA467197 | 3.38E-43 | 1.530761794 | 0.479 | 0.37 | 7.61E-39 |
| Macrophages(M1 like) | Cebpb | 8.20E-36 | 1.414321025 | 0.629 | 0.711 | 1.85E-31 |
| Macrophages(M1 like) | Man2b1 | 7.46E-34 | 1.428026694 | 0.448 | 0.364 | 1.68E-29 |
| Macrophages(M1 like) | Fn1 | 2.44E-21 | 1.499929212 | 0.552 | 0.594 | 5.50E-17 |
| Macrophages(M1 like) | Spp1 | 3.44E-13 | 1.545520658 | 0.563 | 0.643 | 7.76E-09 |
| Macrophages(M2 like) | Arg1 | 0 | 4.06079965 | 0.909 | 0.071 | 0 |
| Macrophages(M2 like) | Ccl6 | 0 | 4.032501323 | 0.89 | 0.081 | 0 |
| Macrophages(M2 like) | Mmp12 | 0 | 3.85192278 | 0.742 | 0.05 | 0 |
| Macrophages(M2 like) | Rgs1 | 0 | 3.775510842 | 0.87 | 0.146 | 0 |
| Macrophages(M2 like) | Ctss | 0 | 3.756625747 | 0.989 | 0.143 | 0 |
| Macrophages(M2 like) | Pf4 | 0 | 3.68657133 | 0.649 | 0.042 | 0 |
| Macrophages(M2 like) | Cd68 | 0 | 3.431845051 | 0.966 | 0.085 | 0 |
| Macrophages(M2 like) | Srgn | 0 | 3.268744466 | 0.955 | 0.099 | 0 |
| Macrophages(M2 like) | Cd14 | 0 | 3.266413653 | 0.909 | 0.184 | 0 |
| Macrophages(M2 like) | Fcer1g | 0 | 3.153581272 | 0.983 | 0.114 | 0 |
| Macrophages(M2 like) | Tyrobp | 0 | 3.140008444 | 0.994 | 0.117 | 0 |
| Macrophages(M2 like) | Clec4d | 0 | 3.096872785 | 0.867 | 0.033 | 0 |
| Macrophages(M2 like) | Wfdc17 | 0 | 3.05844299 | 0.674 | 0.036 | 0 |
| Macrophages(M2 like) | C1qc | 0 | 3.047035521 | 0.788 | 0.095 | 0 |
| Macrophages(M2 like) | C1qb | 0 | 3.010238069 | 0.824 | 0.115 | 0 |
| Macrophages(M2 like) | Fcgr3 | 0 | 2.948351666 | 0.955 | 0.084 | 0 |
| Macrophages(M2 like) | C1qa | 0 | 2.753362308 | 0.756 | 0.079 | 0 |
| Macrophages(M2 like) | C3ar1 | 0 | 2.748743281 | 0.85 | 0.071 | 0 |
| Macrophages(M2 like) | Ccl9 | 0 | 2.676461255 | 0.844 | 0.083 | 0 |
| Macrophages(M2 like) | Fcgr2b | 0 | 2.669504759 | 0.926 | 0.094 | 0 |
| Macrophages(M2 like) | Id2 | 0 | 2.667997389 | 0.827 | 0.121 | 0 |
| Macrophages(M2 like) | Alox5ap | 0 | 2.612276378 | 0.895 | 0.081 | 0 |
| Macrophages(M2 like) | Lilr4b | 0 | 2.51144263 | 0.858 | 0.07 | 0 |
| Macrophages(M2 like) | Trem2 | 0 | 2.381740102 | 0.844 | 0.052 | 0 |
| Macrophages(M2 like) | Clec4n | 0 | 2.378091645 | 0.751 | 0.057 | 0 |
| Macrophages(M2 like) | Cd53 | 0 | 2.352433762 | 0.895 | 0.059 | 0 |
| Macrophages(M2 like) | Ctsc | 0 | 2.328655245 | 0.85 | 0.086 | 0 |
| Macrophages(M2 like) | Hmox1 | 0 | 2.325660253 | 0.686 | 0.044 | 0 |
| Macrophages(M2 like) | Lcp1 | 0 | 2.3003223 | 0.861 | 0.076 | 0 |
| Macrophages(M2 like) | Mafb | 0 | 2.291289322 | 0.694 | 0.046 | 0 |
| Macrophages(M2 like) | Laptm5 | 0 | 2.290645049 | 0.929 | 0.081 | 0 |
| Macrophages(M2 like) | Msr1 | 0 | 2.2403455 | 0.773 | 0.046 | 0 |
| Macrophages(M2 like) | Slfn2 | 0 | 2.203916106 | 0.807 | 0.075 | 0 |
| Macrophages(M2 like) | Ucp2 | 0 | 2.135455391 | 0.841 | 0.11 | 0 |
| Macrophages(M2 like) | Sirpb1c | 0 | 2.098519531 | 0.776 | 0.048 | 0 |
| Macrophages(M2 like) | Cfp | 0 | 2.089327954 | 0.802 | 0.066 | 0 |
| Macrophages(M2 like) | Ms4a6d | 0 | 2.066490899 | 0.674 | 0.044 | 0 |
| Macrophages(M2 like) | Adam8 | 0 | 2.002590694 | 0.683 | 0.046 | 0 |
| Macrophages(M2 like) | Cd52 | 0 | 1.951590127 | 0.935 | 0.12 | 0 |
| Macrophages(M2 like) | Mpeg1 | 0 | 1.936432556 | 0.711 | 0.038 | 0 |
| Macrophages(M2 like) | Cybb | 0 | 1.915484337 | 0.68 | 0.039 | 0 |
| Macrophages(M2 like) | Itgb2 | 0 | 1.849489632 | 0.728 | 0.068 | 0 |
| Macrophages(M2 like) | Cd300c2 | 0 | 1.843958844 | 0.72 | 0.045 | 0 |
| Macrophages(M2 like) | Clec4a2 | 0 | 1.832617487 | 0.674 | 0.041 | 0 |
| Macrophages(M2 like) | Ms4a6c | 0 | 1.814463498 | 0.671 | 0.054 | 0 |
| Macrophages(M2 like) | Clec4e | 0 | 1.807843604 | 0.552 | 0.019 | 0 |
| Macrophages(M2 like) | Ccr1 | 0 | 1.800229035 | 0.691 | 0.049 | 0 |
| Macrophages(M2 like) | Bcl2a1b | 0 | 1.781405019 | 0.72 | 0.042 | 0 |
| Macrophages(M2 like) | Selenop | 0 | 1.765538315 | 0.541 | 0.048 | 0 |
| Macrophages(M2 like) | Spi1 | 0 | 1.696801468 | 0.734 | 0.053 | 0 |
| Macrophages(M2 like) | Pycard | 0 | 1.690055922 | 0.674 | 0.049 | 0 |
| Macrophages(M2 like) | Ly86 | 0 | 1.687255588 | 0.711 | 0.057 | 0 |
| Macrophages(M2 like) | Ccrl2 | 0 | 1.656350708 | 0.45 | 0.037 | 0 |
| Macrophages(M2 like) | Csf2ra | 0 | 1.652562107 | 0.68 | 0.054 | 0 |
| Macrophages(M2 like) | Smpdl3a | 0 | 1.642526041 | 0.598 | 0.024 | 0 |
| Macrophages(M2 like) | Hspa1a | 2.24E-304 | 2.464859203 | 0.62 | 0.075 | 5.06E-300 |
| Macrophages(M2 like) | Emb | 1.78E-297 | 1.771969358 | 0.717 | 0.109 | 4.01E-293 |
| Macrophages(M2 like) | Rgs2 | 1.11E-296 | 2.465636238 | 0.72 | 0.114 | 2.50E-292 |
| Macrophages(M2 like) | Cxcl2 | 1.95E-288 | 3.279898819 | 0.592 | 0.076 | 4.41E-284 |
| Macrophages(M2 like) | Lyz2 | 6.77E-286 | 4.347588091 | 0.989 | 0.325 | 1.53E-281 |
| Macrophages(M2 like) | Apoe | 2.41E-252 | 4.053234679 | 0.819 | 0.195 | 5.43E-248 |
| Macrophages(M2 like) | Fth1 | 1.49E-222 | 3.355234982 | 1 | 0.992 | 3.35E-218 |
| Macrophages(M2 like) | Cxcl16 | 4.62E-214 | 1.734801516 | 0.688 | 0.14 | 1.04E-209 |
| Macrophages(M2 like) | Lgals3 | 4.56E-198 | 2.077936512 | 1 | 0.974 | 1.03E-193 |
| Macrophages(M2 like) | Ccl3 | 1.84E-196 | 1.946133916 | 0.411 | 0.049 | 4.14E-192 |
| Macrophages(M2 like) | Fxyd5 | 6.60E-182 | 2.115146736 | 0.992 | 0.899 | 1.49E-177 |
| Macrophages(M2 like) | Npc2 | 3.26E-181 | 1.968062122 | 0.983 | 0.941 | 7.35E-177 |
| Macrophages(M2 like) | Ftl1 | 3.56E-181 | 2.431280447 | 0.989 | 0.959 | 8.03E-177 |
| Macrophages(M2 like) | Ctsb | 3.01E-179 | 2.414915194 | 0.989 | 0.896 | 6.79E-175 |
| Macrophages(M2 like) | Cyba | 5.99E-175 | 1.946407934 | 0.986 | 0.821 | 1.35E-170 |
| Macrophages(M2 like) | Sat1 | 1.05E-174 | 2.375785738 | 0.972 | 0.718 | 2.36E-170 |
| Macrophages(M2 like) | Tmsb4x | 1.05E-173 | 2.084440395 | 1 | 0.983 | 2.36E-169 |
| Macrophages(M2 like) | Ctsd | 4.71E-173 | 2.731693646 | 0.955 | 0.75 | 1.06E-168 |
| Macrophages(M2 like) | Tmem189 | 2.92E-166 | 1.674177349 | 0.773 | 0.27 | 6.57E-162 |
| Macrophages(M2 like) | Psap | 1.90E-159 | 1.935983823 | 0.963 | 0.87 | 4.27E-155 |
| Macrophages(M2 like) | H2-Ab1 | 8.88E-159 | 1.765400915 | 0.861 | 0.319 | 2.00E-154 |
| Macrophages(M2 like) | H2-Aa | 3.37E-158 | 2.344811107 | 0.864 | 0.349 | 7.59E-154 |
| Macrophages(M2 like) | Cd74 | 1.72E-157 | 2.569834616 | 0.955 | 0.585 | 3.89E-153 |
| Macrophages(M2 like) | Plin2 | 6.86E-157 | 2.171392515 | 0.946 | 0.76 | 1.55E-152 |
| Macrophages(M2 like) | Lgmn | 2.31E-151 | 2.308174105 | 0.907 | 0.567 | 5.21E-147 |
| Macrophages(M2 like) | Mcl1 | 7.56E-151 | 2.130438511 | 0.909 | 0.586 | 1.71E-146 |
| Macrophages(M2 like) | Hilpda | 2.45E-149 | 2.849793404 | 0.748 | 0.287 | 5.52E-145 |
| Macrophages(M2 like) | Prdx5 | 1.01E-148 | 1.99373227 | 0.921 | 0.622 | 2.27E-144 |
| Macrophages(M2 like) | Cebpb | 6.43E-142 | 2.292679659 | 0.935 | 0.705 | 1.45E-137 |
| Macrophages(M2 like) | Metrnl | 1.37E-135 | 1.990583285 | 0.904 | 0.591 | 3.09E-131 |
| Macrophages(M2 like) | Basp1 | 1.34E-132 | 1.675813844 | 0.756 | 0.311 | 3.02E-128 |
| Macrophages(M2 like) | Spp1 | 1.11E-131 | 3.303994802 | 0.909 | 0.636 | 2.50E-127 |
| Macrophages(M2 like) | Atf3 | 2.67E-120 | 2.056977583 | 0.796 | 0.337 | 6.02E-116 |
| Macrophages(M2 like) | Malat1 | 3.48E-117 | 1.918993638 | 0.994 | 0.918 | 7.86E-113 |
| Macrophages(M2 like) | Dusp1 | 4.09E-109 | 2.458985249 | 0.87 | 0.557 | 9.22E-105 |
| Macrophages(M2 like) | Zfp36 | 4.81E-106 | 1.960824285 | 0.807 | 0.4 | 1.08E-101 |
| Macrophages(M2 like) | H2-Eb1 | 1.35E-104 | 1.851783094 | 0.742 | 0.324 | 3.05E-100 |
| Macrophages(M2 like) | Btg1 | 7.57E-103 | 1.764413389 | 0.856 | 0.574 | 1.71E-98 |
| Macrophages(M2 like) | Rgcc | 2.84E-101 | 2.268390881 | 0.722 | 0.321 | 6.39E-97 |
| Macrophages(M2 like) | Fos | 9.07E-99 | 1.820087584 | 0.853 | 0.439 | 2.04E-94 |
| Macrophages(M2 like) | Fabp5 | 2.02E-93 | 1.907633918 | 0.592 | 0.232 | 4.55E-89 |
| Macrophages(M2 like) | Cdkn1a | 4.78E-88 | 1.915561553 | 0.802 | 0.49 | 1.08E-83 |
| Macrophages(M2 like) | Plk2 | 1.17E-55 | 1.963437653 | 0.691 | 0.471 | 2.64E-51 |
| Macrophages(M2 like) | Ccl2 | 7.31E-31 | 2.286660801 | 0.669 | 0.58 | 1.65E-26 |
| Macrophages(M2 like) | Ccl7 | 1.68E-18 | 2.204319065 | 0.377 | 0.234 | 3.78E-14 |
| DCs | Cxcl9 | 0 | 3.958018665 | 0.723 | 0.027 | 0 |
| DCs | Ms4a4c | 0 | 2.251103176 | 0.564 | 0.016 | 0 |
| DCs | Acod1 | 0 | 1.798681191 | 0.351 | 0.004 | 0 |
| DCs | Fcgr4 | 2.42E-307 | 2.161969691 | 0.606 | 0.021 | 5.46E-303 |
| DCs | Ifi207 | 5.89E-258 | 2.062293199 | 0.628 | 0.028 | 1.33E-253 |
| DCs | Ifi209 | 1.02E-252 | 2.038614453 | 0.543 | 0.021 | 2.30E-248 |
| DCs | Fgl2 | 2.08E-251 | 2.584141542 | 0.649 | 0.03 | 4.69E-247 |
| DCs | Ms4a6c | 4.97E-233 | 2.782362907 | 0.872 | 0.061 | 1.12E-228 |
| DCs | Fcgr1 | 1.28E-232 | 2.232553814 | 0.734 | 0.042 | 2.90E-228 |
| DCs | Cybb | 5.52E-216 | 2.223133244 | 0.745 | 0.047 | 1.25E-211 |
| DCs | Bcl2a1b | 4.05E-195 | 2.060242051 | 0.734 | 0.05 | 9.12E-191 |
| DCs | Mgl2 | 3.64E-187 | 2.276202857 | 0.521 | 0.026 | 8.21E-183 |
| DCs | Csf2ra | 2.94E-185 | 2.068863048 | 0.787 | 0.061 | 6.63E-181 |
| DCs | Ly86 | 1.11E-184 | 2.655009355 | 0.798 | 0.065 | 2.50E-180 |
| DCs | Ms4a6d | 1.77E-183 | 2.229808832 | 0.723 | 0.052 | 3.99E-179 |
| DCs | Slfn2 | 2.14E-183 | 2.649408567 | 0.894 | 0.083 | 4.83E-179 |
| DCs | Fcgr3 | 2.14E-180 | 3.003170973 | 0.947 | 0.095 | 4.82E-176 |
| DCs | Ctsc | 7.88E-178 | 3.029611285 | 0.936 | 0.095 | 1.78E-173 |
| DCs | Ch25h | 1.55E-175 | 1.805315266 | 0.287 | 0.008 | 3.49E-171 |
| DCs | Clec4n | 8.43E-175 | 2.598014076 | 0.787 | 0.065 | 1.90E-170 |
| DCs | Spi1 | 1.41E-169 | 2.188784434 | 0.755 | 0.062 | 3.18E-165 |
| DCs | Plbd1 | 1.03E-162 | 1.949364256 | 0.628 | 0.044 | 2.32E-158 |
| DCs | Fcer1g | 1.44E-160 | 3.497551595 | 0.989 | 0.124 | 3.24E-156 |
| DCs | Aif1 | 1.68E-155 | 2.316475466 | 0.628 | 0.046 | 3.79E-151 |
| DCs | Sirpb1c | 1.06E-153 | 2.078224623 | 0.702 | 0.058 | 2.39E-149 |
| DCs | C1qa | 3.97E-151 | 3.250242491 | 0.83 | 0.087 | 8.96E-147 |
| DCs | Srgn | 1.29E-150 | 2.651682809 | 0.947 | 0.109 | 2.91E-146 |
| DCs | Laptm5 | 6.45E-149 | 2.247120133 | 0.872 | 0.092 | 1.45E-144 |
| DCs | Pld4 | 9.68E-148 | 1.997038238 | 0.734 | 0.065 | 2.18E-143 |
| DCs | C1qc | 3.11E-144 | 3.434330442 | 0.872 | 0.103 | 7.00E-140 |
| DCs | Fcgr2b | 5.67E-144 | 2.561772356 | 0.894 | 0.105 | 1.28E-139 |
| DCs | Cd52 | 1.91E-142 | 3.120472451 | 0.968 | 0.13 | 4.30E-138 |
| DCs | AW112010 | 4.32E-142 | 3.797568192 | 0.926 | 0.124 | 9.73E-138 |
| DCs | Tyrobp | 8.79E-142 | 3.028784573 | 0.968 | 0.128 | 1.98E-137 |
| DCs | Alox5ap | 6.49E-139 | 2.268437008 | 0.84 | 0.091 | 1.46E-134 |
| DCs | Ptprc | 1.57E-138 | 1.997924551 | 0.766 | 0.076 | 3.54E-134 |
| DCs | C1qb | 6.60E-137 | 3.602915654 | 0.915 | 0.123 | 1.49E-132 |
| DCs | Lilr4b | 8.46E-133 | 2.011585649 | 0.766 | 0.08 | 1.91E-128 |
| DCs | Coro1a | 1.25E-131 | 2.1157085 | 0.883 | 0.108 | 2.81E-127 |
| DCs | Ctss | 2.73E-131 | 3.357846626 | 0.989 | 0.153 | 6.16E-127 |
| DCs | Msr1 | 1.60E-130 | 1.907241746 | 0.638 | 0.055 | 3.61E-126 |
| DCs | Cd83 | 1.07E-128 | 2.26653135 | 0.532 | 0.039 | 2.41E-124 |
| DCs | Il1rn | 2.24E-125 | 1.83980533 | 0.383 | 0.021 | 5.04E-121 |
| DCs | Ucp2 | 7.69E-125 | 2.387626468 | 0.883 | 0.119 | 1.73E-120 |
| DCs | Cfp | 7.68E-122 | 2.220385038 | 0.713 | 0.076 | 1.73E-117 |
| DCs | Ccr1 | 1.95E-121 | 1.850353315 | 0.628 | 0.057 | 4.40E-117 |
| DCs | Ccr2 | 4.22E-118 | 1.904427565 | 0.585 | 0.051 | 9.50E-114 |
| DCs | Trem2 | 1.03E-117 | 1.789006517 | 0.649 | 0.063 | 2.32E-113 |
| DCs | Pycard | 2.42E-115 | 1.761665038 | 0.606 | 0.057 | 5.47E-111 |
| DCs | Lcp1 | 3.31E-113 | 2.030387098 | 0.745 | 0.086 | 7.46E-109 |
| DCs | Cd68 | 5.22E-111 | 2.010941639 | 0.777 | 0.097 | 1.18E-106 |
| DCs | Ccl12 | 2.47E-103 | 3.428589723 | 0.33 | 0.019 | 5.56E-99 |
| DCs | Cxcl16 | 1.48E-98 | 2.48666873 | 0.84 | 0.146 | 3.34E-94 |
| DCs | Ccl6 | 3.94E-97 | 2.026578038 | 0.713 | 0.092 | 8.87E-93 |
| DCs | Hspa1a | 9.92E-93 | 2.643154716 | 0.649 | 0.082 | 2.24E-88 |
| DCs | H2-DMa | 1.42E-89 | 3.21396323 | 0.936 | 0.225 | 3.20E-85 |
| DCs | C3ar1 | 2.88E-89 | 1.949617864 | 0.638 | 0.081 | 6.50E-85 |
| DCs | Emb | 7.71E-89 | 1.875821137 | 0.745 | 0.117 | 1.74E-84 |
| DCs | Wfdc17 | 6.91E-86 | 2.198971326 | 0.468 | 0.045 | 1.56E-81 |
| DCs | Ccrl2 | 1.47E-79 | 1.982179207 | 0.436 | 0.042 | 3.30E-75 |
| DCs | Ccl9 | 4.34E-78 | 1.788699339 | 0.638 | 0.093 | 9.79E-74 |
| DCs | Unc93b1 | 1.88E-76 | 1.889781875 | 0.713 | 0.127 | 4.23E-72 |
| DCs | H2-Eb1 | 2.84E-74 | 3.912455603 | 0.968 | 0.329 | 6.41E-70 |
| DCs | H2-Ab1 | 1.31E-70 | 3.798803158 | 0.947 | 0.325 | 2.94E-66 |
| DCs | H2-Aa | 1.39E-68 | 3.842439739 | 0.957 | 0.355 | 3.13E-64 |
| DCs | Arg1 | 6.96E-66 | 2.40191509 | 0.564 | 0.083 | 1.57E-61 |
| DCs | Apoe | 1.49E-65 | 2.818685779 | 0.819 | 0.203 | 3.35E-61 |
| DCs | Iigp1 | 8.62E-64 | 2.130301088 | 0.766 | 0.182 | 1.94E-59 |
| DCs | Lyz2 | 5.77E-63 | 2.965794317 | 0.947 | 0.334 | 1.30E-58 |
| DCs | Cd74 | 1.37E-60 | 4.072345977 | 1 | 0.59 | 3.09E-56 |
| DCs | Ifit3 | 4.22E-57 | 1.953988649 | 0.266 | 0.022 | 9.51E-53 |
| DCs | Tmsb4x | 1.82E-51 | 2.411289131 | 1 | 0.983 | 4.10E-47 |
| DCs | Gbp2 | 4.28E-50 | 2.723159522 | 0.787 | 0.267 | 9.65E-46 |
| DCs | Cyba | 1.42E-49 | 2.143416053 | 0.979 | 0.823 | 3.21E-45 |
| DCs | Cxcl10 | 5.18E-49 | 3.246040776 | 0.468 | 0.082 | 1.17E-44 |
| DCs | AA467197 | 5.50E-47 | 2.514865303 | 0.84 | 0.372 | 1.24E-42 |
| DCs | Ifi204 | 1.01E-45 | 1.929605418 | 0.596 | 0.142 | 2.28E-41 |
| DCs | Fxyd5 | 3.99E-44 | 1.839384024 | 0.979 | 0.9 | 9.00E-40 |
| DCs | Fth1 | 7.99E-44 | 1.90900905 | 0.989 | 0.992 | 1.80E-39 |
| DCs | H2-DMb1 | 2.93E-43 | 2.083989236 | 0.862 | 0.439 | 6.59E-39 |
| DCs | Samhd1 | 6.79E-42 | 2.223192484 | 0.851 | 0.437 | 1.53E-37 |
| DCs | Ifi47 | 3.33E-40 | 1.794441976 | 0.649 | 0.197 | 7.51E-36 |
| DCs | Prdx5 | 1.81E-39 | 1.898436138 | 0.926 | 0.626 | 4.08E-35 |
| DCs | Cst3 | 2.36E-38 | 2.070149051 | 0.968 | 0.924 | 5.31E-34 |
| DCs | Id2 | 9.29E-37 | 1.81295086 | 0.553 | 0.131 | 2.09E-32 |
| DCs | Isg15 | 1.92E-36 | 2.486702883 | 0.553 | 0.149 | 4.32E-32 |
| DCs | Ccl3 | 1.30E-35 | 2.178729408 | 0.34 | 0.054 | 2.94E-31 |
| DCs | Tgfbi | 1.61E-35 | 2.090564493 | 0.926 | 0.761 | 3.62E-31 |
| DCs | Ccl4 | 3.37E-35 | 3.247790418 | 0.415 | 0.082 | 7.60E-31 |
| DCs | Rgs1 | 1.36E-31 | 2.507933204 | 0.553 | 0.156 | 3.06E-27 |
| DCs | Cxcl2 | 1.37E-27 | 2.257818103 | 0.383 | 0.083 | 3.08E-23 |
| DCs | Naaa | 8.99E-25 | 1.783157451 | 0.66 | 0.331 | 2.03E-20 |
| DCs | Ifi203 | 5.05E-22 | 1.761478797 | 0.457 | 0.151 | 1.14E-17 |
| DCs | Zfp36 | 2.64E-19 | 2.389024211 | 0.681 | 0.406 | 5.96E-15 |
| DCs | Ccl5 | 3.59E-19 | 1.82622986 | 0.266 | 0.056 | 8.10E-15 |
| DCs | Ifit2 | 1.11E-18 | 1.984331358 | 0.404 | 0.139 | 2.50E-14 |
| DCs | Atf3 | 1.06E-15 | 2.227496229 | 0.617 | 0.343 | 2.38E-11 |
| DCs | Dusp1 | 2.65E-13 | 1.882095009 | 0.723 | 0.561 | 5.98E-09 |
| DCs | Socs3 | 1.96E-07 | 1.777293106 | 0.543 | 0.433 | 0.004422312 |
| DCs | Ccl2 | 2.92E-05 | 1.958336881 | 0.596 | 0.581 | 0.65765663 |
| T01_Cd8+ T cells | Ccl5 | 0 | 4.980220723 | 0.634 | 0.05 | 0 |
| T01_Cd8+ T cells | Nkg7 | 0 | 4.738698373 | 0.839 | 0.019 | 0 |
| T01_Cd8+ T cells | Ms4a4b | 0 | 4.404607278 | 0.862 | 0.013 | 0 |
| T01_Cd8+ T cells | Trbc2 | 0 | 3.994305031 | 0.744 | 0.013 | 0 |
| T01_Cd8+ T cells | Cd52 | 0 | 3.948870445 | 0.929 | 0.124 | 0 |
| T01_Cd8+ T cells | AW112010 | 0 | 3.891397038 | 0.835 | 0.119 | 0 |
| T01_Cd8+ T cells | Cd3g | 0 | 3.544669896 | 0.709 | 0.011 | 0 |
| T01_Cd8+ T cells | Thy1 | 0 | 3.53530002 | 0.717 | 0.012 | 0 |
| T01_Cd8+ T cells | Rac2 | 0 | 3.468040515 | 0.803 | 0.063 | 0 |
| T01_Cd8+ T cells | Cd3d | 0 | 3.381133727 | 0.665 | 0.011 | 0 |
| T01_Cd8+ T cells | Trac | 0 | 3.266569746 | 0.661 | 0.016 | 0 |
| T01_Cd8+ T cells | Ptprcap | 0 | 3.159212169 | 0.634 | 0.01 | 0 |
| T01_Cd8+ T cells | Trbc1 | 0 | 3.078669225 | 0.433 | 0.007 | 0 |
| T01_Cd8+ T cells | Hcst | 0 | 3.066009218 | 0.594 | 0.032 | 0 |
| T01_Cd8+ T cells | Cd3e | 0 | 2.989878505 | 0.638 | 0.009 | 0 |
| T01_Cd8+ T cells | Ptprc | 0 | 2.934904606 | 0.701 | 0.071 | 0 |
| T01_Cd8+ T cells | Ctsw | 0 | 2.927497913 | 0.598 | 0.013 | 0 |
| T01_Cd8+ T cells | Klrd1 | 0 | 2.917464957 | 0.496 | 0.009 | 0 |
| T01_Cd8+ T cells | Cd8b1 | 0 | 2.898433111 | 0.539 | 0.008 | 0 |
| T01_Cd8+ T cells | Cxcr6 | 0 | 2.793906986 | 0.524 | 0.009 | 0 |
| T01_Cd8+ T cells | Cd8a | 0 | 2.787060181 | 0.528 | 0.007 | 0 |
| T01_Cd8+ T cells | Ltb | 0 | 2.741316557 | 0.461 | 0.008 | 0 |
| T01_Cd8+ T cells | 1-Sep | 0 | 2.634032569 | 0.512 | 0.036 | 0 |
| T01_Cd8+ T cells | Cd2 | 0 | 2.614461033 | 0.516 | 0.008 | 0 |
| T01_Cd8+ T cells | Lck | 0 | 2.59228716 | 0.5 | 0.019 | 0 |
| T01_Cd8+ T cells | Gimap4 | 0 | 2.588493177 | 0.524 | 0.007 | 0 |
| T01_Cd8+ T cells | Klrc1 | 0 | 2.426339261 | 0.465 | 0.008 | 0 |
| T01_Cd8+ T cells | Lat | 0 | 2.420389956 | 0.449 | 0.008 | 0 |
| T01_Cd8+ T cells | Gimap6 | 0 | 2.238895567 | 0.406 | 0.01 | 0 |
| T01_Cd8+ T cells | Klrk1 | 0 | 2.233600679 | 0.421 | 0.013 | 0 |
| T01_Cd8+ T cells | Il2rb | 0 | 2.212584239 | 0.437 | 0.008 | 0 |
| T01_Cd8+ T cells | Nrgn | 0 | 2.200251882 | 0.283 | 0.005 | 0 |
| T01_Cd8+ T cells | Gimap3 | 0 | 2.180312555 | 0.386 | 0.006 | 0 |
| T01_Cd8+ T cells | Tnfrsf18 | 0 | 2.160214279 | 0.346 | 0.006 | 0 |
| T01_Cd8+ T cells | Sh2d2a | 0 | 2.128187637 | 0.402 | 0.008 | 0 |
| T01_Cd8+ T cells | Skap1 | 0 | 2.070793741 | 0.39 | 0.009 | 0 |
| T01_Cd8+ T cells | Gzmb | 0 | 2.034653595 | 0.252 | 0.005 | 0 |
| T01_Cd8+ T cells | Ifng | 0 | 1.932997773 | 0.252 | 0.007 | 0 |
| T01_Cd8+ T cells | Klre1 | 0 | 1.802709295 | 0.256 | 0.003 | 0 |
| T01_Cd8+ T cells | Cst7 | 0 | 1.800725348 | 0.303 | 0.005 | 0 |
| T01_Cd8+ T cells | Cd247 | 0 | 1.761648945 | 0.303 | 0.004 | 0 |
| T01_Cd8+ T cells | Gm8369 | 0 | 1.725154256 | 0.303 | 0.006 | 0 |
| T01_Cd8+ T cells | Gimap5 | 0 | 1.710016814 | 0.28 | 0.005 | 0 |
| T01_Cd8+ T cells | Gimap1 | 0 | 1.646507974 | 0.276 | 0.003 | 0 |
| T01_Cd8+ T cells | Id2 | 1.26E-305 | 3.636501117 | 0.85 | 0.124 | 2.84E-301 |
| T01_Cd8+ T cells | H2-Q7 | 4.27E-301 | 2.58138522 | 0.587 | 0.054 | 9.62E-297 |
| T01_Cd8+ T cells | Ly6c2 | 5.67E-292 | 3.753547058 | 0.39 | 0.023 | 1.28E-287 |
| T01_Cd8+ T cells | Coro1a | 5.87E-273 | 3.002154195 | 0.752 | 0.103 | 1.32E-268 |
| T01_Cd8+ T cells | Rinl | 5.34E-216 | 1.754057054 | 0.339 | 0.023 | 1.20E-211 |
| T01_Cd8+ T cells | Ms4a6b | 2.05E-215 | 2.181504288 | 0.425 | 0.037 | 4.61E-211 |
| T01_Cd8+ T cells | Gmfg | 1.16E-209 | 2.245681826 | 0.48 | 0.05 | 2.62E-205 |
| T01_Cd8+ T cells | Selplg | 9.67E-204 | 2.177221814 | 0.488 | 0.053 | 2.18E-199 |
| T01_Cd8+ T cells | Srgn | 3.33E-201 | 2.31608946 | 0.693 | 0.106 | 7.51E-197 |
| T01_Cd8+ T cells | Lcp1 | 1.60E-200 | 2.40968338 | 0.602 | 0.083 | 3.61E-196 |
| T01_Cd8+ T cells | Tbc1d10c | 5.37E-192 | 1.832079513 | 0.315 | 0.023 | 1.21E-187 |
| T01_Cd8+ T cells | Laptm5 | 5.09E-188 | 2.147850729 | 0.614 | 0.089 | 1.15E-183 |
| T01_Cd8+ T cells | Mir142hg | 2.64E-168 | 1.549823991 | 0.264 | 0.018 | 5.96E-164 |
| T01_Cd8+ T cells | Cd48 | 1.45E-162 | 1.897322712 | 0.378 | 0.039 | 3.27E-158 |
| T01_Cd8+ T cells | Samsn1 | 4.73E-151 | 1.733161225 | 0.291 | 0.025 | 1.07E-146 |
| T01_Cd8+ T cells | Arl6ip1 | 1.40E-139 | 1.926089112 | 0.413 | 0.055 | 3.16E-135 |
| T01_Cd8+ T cells | Cd69 | 6.48E-138 | 1.938912653 | 0.26 | 0.021 | 1.46E-133 |
| T01_Cd8+ T cells | Tmsb4x | 1.80E-136 | 2.761482506 | 0.996 | 0.983 | 4.06E-132 |
| T01_Cd8+ T cells | Snord13 | 2.26E-134 | 2.398515708 | 0.343 | 0.039 | 5.10E-130 |
| T01_Cd8+ T cells | Cd37 | 1.50E-133 | 1.416698566 | 0.28 | 0.026 | 3.38E-129 |
| T01_Cd8+ T cells | Bin2 | 8.02E-130 | 1.547977148 | 0.268 | 0.024 | 1.81E-125 |
| T01_Cd8+ T cells | Epsti1 | 2.31E-123 | 1.508828851 | 0.256 | 0.023 | 5.21E-119 |
| T01_Cd8+ T cells | Tmsb10 | 3.99E-121 | 1.788529777 | 0.98 | 0.979 | 8.99E-117 |
| T01_Cd8+ T cells | Cd53 | 4.55E-100 | 1.848807231 | 0.406 | 0.069 | 1.03E-95 |
| T01_Cd8+ T cells | H2-Q6 | 2.72E-76 | 1.543155699 | 0.252 | 0.035 | 6.12E-72 |
| T01_Cd8+ T cells | Ccl4 | 2.61E-70 | 2.871478152 | 0.37 | 0.08 | 5.88E-66 |
| T01_Cd8+ T cells | Slfn2 | 1.76E-69 | 1.605744914 | 0.382 | 0.083 | 3.97E-65 |
| T01_Cd8+ T cells | Tnfrsf9 | 6.99E-68 | 1.641290207 | 0.264 | 0.043 | 1.58E-63 |
| T01_Cd8+ T cells | Itgb2 | 7.79E-66 | 1.603120979 | 0.354 | 0.076 | 1.76E-61 |
| T01_Cd8+ T cells | Il2rg | 2.06E-62 | 2.338015965 | 0.551 | 0.221 | 4.64E-58 |
| T01_Cd8+ T cells | Fermt3 | 2.75E-60 | 1.32005188 | 0.283 | 0.053 | 6.19E-56 |
| T01_Cd8+ T cells | Ucp2 | 5.34E-58 | 1.433986062 | 0.433 | 0.119 | 1.20E-53 |
| T01_Cd8+ T cells | Gm42418 | 7.97E-58 | 1.698502619 | 0.961 | 0.984 | 1.80E-53 |
| T01_Cd8+ T cells | Serpina3g | 1.76E-54 | 1.592286621 | 0.299 | 0.067 | 3.98E-50 |
| T01_Cd8+ T cells | Ptpn18 | 3.98E-51 | 2.190617502 | 0.575 | 0.286 | 8.97E-47 |
| T01_Cd8+ T cells | Gm26917 | 3.83E-48 | 2.988894118 | 0.669 | 0.466 | 8.63E-44 |
| T01_Cd8+ T cells | Malat1 | 2.78E-47 | 1.68336976 | 0.925 | 0.919 | 6.27E-43 |
| T01_Cd8+ T cells | Ptpn6 | 7.46E-45 | 1.368018262 | 0.256 | 0.057 | 1.68E-40 |
| T01_Cd8+ T cells | Fxyd5 | 8.67E-43 | 1.427752713 | 0.807 | 0.902 | 1.95E-38 |
| T01_Cd8+ T cells | Bcl2 | 6.29E-42 | 1.677715406 | 0.26 | 0.063 | 1.42E-37 |
| T01_Cd8+ T cells | Ctla2a | 1.55E-38 | 1.892440423 | 0.39 | 0.148 | 3.50E-34 |
| T01_Cd8+ T cells | Cotl1 | 7.99E-35 | 1.259264798 | 0.291 | 0.087 | 1.80E-30 |
| T01_Cd8+ T cells | Lsp1 | 6.60E-34 | 1.809885981 | 0.606 | 0.433 | 1.49E-29 |
| T01_Cd8+ T cells | Arhgdib | 3.67E-33 | 1.824156445 | 0.693 | 0.703 | 8.27E-29 |
| T01_Cd8+ T cells | Ptpn22 | 3.61E-32 | 1.511185522 | 0.252 | 0.073 | 8.14E-28 |
| T01_Cd8+ T cells | Shisa5 | 1.20E-31 | 1.97956162 | 0.642 | 0.588 | 2.69E-27 |
| T01_Cd8+ T cells | Sub1 | 1.84E-27 | 1.365936007 | 0.717 | 0.87 | 4.14E-23 |
| T01_Cd8+ T cells | Psmb8 | 6.38E-26 | 1.348860442 | 0.728 | 0.844 | 1.44E-21 |
| T01_Cd8+ T cells | B4galnt1 | 4.19E-24 | 1.43774546 | 0.287 | 0.113 | 9.44E-20 |
| T01_Cd8+ T cells | Sp100 | 6.64E-15 | 1.40436886 | 0.335 | 0.199 | 1.50E-10 |
| T01_Cd8+ T cells | Limd2 | 4.42E-12 | 1.609289265 | 0.425 | 0.353 | 9.96E-08 |
| T01_Cd8+ T cells | Pld3 | 1.57E-07 | 1.394451236 | 0.343 | 0.281 | 0.003536286 |
| T01_Cd8+ T cells | Ifngr1 | 3.98E-06 | 1.689048659 | 0.425 | 0.452 | 0.08983378 |
| T01_Cd8+ T cells | Anxa6 | 4.57E-06 | 1.416061439 | 0.398 | 0.4 | 0.103108246 |
| T01_Cd8+ T cells | Ets1 | 1.35E-05 | 1.54240292 | 0.327 | 0.292 | 0.304048821 |
| T01_Cd8+ T cells | Clec2d | 1.49E-05 | 1.365527184 | 0.354 | 0.328 | 0.334850553 |
| T02_Dnajb1 high | Cd69 | 0 | 4.187871725 | 0.767 | 0.021 | 0 |
| T02_Dnajb1 high | Nkg7 | 0 | 4.167253388 | 0.756 | 0.025 | 0 |
| T02_Dnajb1 high | Trac | 0 | 3.841521494 | 0.778 | 0.02 | 0 |
| T02_Dnajb1 high | Trbc2 | 0 | 3.621975417 | 0.744 | 0.019 | 0 |
| T02_Dnajb1 high | Ms4a4b | 0 | 3.550857325 | 0.678 | 0.021 | 0 |
| T02_Dnajb1 high | Thy1 | 0 | 3.440832386 | 0.7 | 0.018 | 0 |
| T02_Dnajb1 high | Cd3g | 0 | 3.162025061 | 0.733 | 0.017 | 0 |
| T02_Dnajb1 high | Cd3d | 0 | 3.120771373 | 0.7 | 0.016 | 0 |
| T02_Dnajb1 high | Cxcr6 | 0 | 2.983296184 | 0.556 | 0.013 | 0 |
| T02_Dnajb1 high | Gimap6 | 0 | 2.843662448 | 0.567 | 0.012 | 0 |
| T02_Dnajb1 high | Klrc1 | 0 | 2.751005416 | 0.478 | 0.011 | 0 |
| T02_Dnajb1 high | Lat | 0 | 2.726477062 | 0.511 | 0.011 | 0 |
| T02_Dnajb1 high | Cd28 | 0 | 2.611914649 | 0.456 | 0.005 | 0 |
| T02_Dnajb1 high | Sh2d2a | 0 | 2.522267335 | 0.533 | 0.011 | 0 |
| T02_Dnajb1 high | Icos | 0 | 2.367324009 | 0.378 | 0.005 | 0 |
| T02_Dnajb1 high | Gimap3 | 0 | 2.223988933 | 0.444 | 0.008 | 0 |
| T02_Dnajb1 high | Cxcr3 | 0 | 1.889765982 | 0.3 | 0.003 | 0 |
| T02_Dnajb1 high | Gpr183 | 1.75E-289 | 3.214203241 | 0.6 | 0.022 | 3.95E-285 |
| T02_Dnajb1 high | Cd3e | 3.67E-286 | 2.500646283 | 0.5 | 0.014 | 8.27E-282 |
| T02_Dnajb1 high | Gimap4 | 2.61E-284 | 2.165483621 | 0.444 | 0.011 | 5.89E-280 |
| T02_Dnajb1 high | Ifng | 6.37E-283 | 3.140400303 | 0.389 | 0.009 | 1.44E-278 |
| T02_Dnajb1 high | Tespa1 | 2.02E-282 | 1.679180994 | 0.3 | 0.005 | 4.54E-278 |
| T02_Dnajb1 high | Gzmb | 9.59E-259 | 2.767242979 | 0.322 | 0.006 | 2.16E-254 |
| T02_Dnajb1 high | Cd8b1 | 6.08E-253 | 2.540085891 | 0.433 | 0.012 | 1.37E-248 |
| T02_Dnajb1 high | Ptprcap | 4.65E-250 | 2.466841408 | 0.478 | 0.015 | 1.05E-245 |
| T02_Dnajb1 high | Trbc1 | 4.63E-249 | 2.937619115 | 0.4 | 0.011 | 1.04E-244 |
| T02_Dnajb1 high | Ctsw | 8.02E-249 | 2.889386776 | 0.511 | 0.018 | 1.81E-244 |
| T02_Dnajb1 high | Cd2 | 2.66E-247 | 2.529143845 | 0.433 | 0.013 | 5.99E-243 |
| T02_Dnajb1 high | Gm8369 | 1.36E-241 | 2.169121533 | 0.344 | 0.008 | 3.07E-237 |
| T02_Dnajb1 high | Cd6 | 4.53E-231 | 1.660556843 | 0.267 | 0.005 | 1.02E-226 |
| T02_Dnajb1 high | Il2rb | 2.18E-212 | 2.023613695 | 0.378 | 0.011 | 4.92E-208 |
| T02_Dnajb1 high | Ltb | 2.13E-208 | 2.383362052 | 0.378 | 0.011 | 4.80E-204 |
| T02_Dnajb1 high | Dusp2 | 4.98E-195 | 3.350521489 | 0.489 | 0.021 | 1.12E-190 |
| T02_Dnajb1 high | Pdcd1 | 5.97E-194 | 2.068270584 | 0.3 | 0.008 | 1.35E-189 |
| T02_Dnajb1 high | Cd8a | 7.97E-194 | 2.369299392 | 0.367 | 0.012 | 1.80E-189 |
| T02_Dnajb1 high | Gimap1 | 3.86E-193 | 1.763816831 | 0.256 | 0.005 | 8.70E-189 |
| T02_Dnajb1 high | Tnfaip3 | 1.26E-182 | 2.779225623 | 0.567 | 0.032 | 2.84E-178 |
| T02_Dnajb1 high | Klrk1 | 4.48E-182 | 2.105659253 | 0.411 | 0.016 | 1.01E-177 |
| T02_Dnajb1 high | Lck | 3.48E-181 | 2.572354323 | 0.489 | 0.023 | 7.85E-177 |
| T02_Dnajb1 high | Rac2 | 2.09E-169 | 3.260246071 | 0.789 | 0.069 | 4.70E-165 |
| T02_Dnajb1 high | Tagap | 3.82E-160 | 1.843784945 | 0.256 | 0.007 | 8.61E-156 |
| T02_Dnajb1 high | Hcst | 2.05E-151 | 2.838849836 | 0.556 | 0.036 | 4.63E-147 |
| T02_Dnajb1 high | Gimap5 | 7.74E-150 | 1.677503419 | 0.256 | 0.007 | 1.74E-145 |
| T02_Dnajb1 high | Ptprc | 1.05E-128 | 3.044884895 | 0.722 | 0.076 | 2.36E-124 |
| T02_Dnajb1 high | Ccl5 | 8.46E-115 | 4.715467294 | 0.589 | 0.055 | 1.91E-110 |
| T02_Dnajb1 high | Id2 | 1.67E-114 | 3.681517606 | 0.867 | 0.13 | 3.75E-110 |
| T02_Dnajb1 high | Rinl | 1.03E-110 | 2.180109489 | 0.4 | 0.025 | 2.33E-106 |
| T02_Dnajb1 high | H2-Q7 | 5.41E-109 | 2.625200151 | 0.589 | 0.058 | 1.22E-104 |
| T02_Dnajb1 high | Srgn | 2.00E-107 | 3.195616193 | 0.8 | 0.11 | 4.51E-103 |
| T02_Dnajb1 high | 1-Sep | 3.61E-98 | 2.547103599 | 0.467 | 0.04 | 8.14E-94 |
| T02_Dnajb1 high | Ly6c2 | 4.13E-97 | 3.14381736 | 0.378 | 0.026 | 9.32E-93 |
| T02_Dnajb1 high | Klrd1 | 3.36E-94 | 1.863736943 | 0.278 | 0.014 | 7.57E-90 |
| T02_Dnajb1 high | Arl6ip1 | 4.53E-93 | 2.215607526 | 0.544 | 0.057 | 1.02E-88 |
| T02_Dnajb1 high | Cd52 | 1.36E-91 | 3.158676829 | 0.811 | 0.131 | 3.08E-87 |
| T02_Dnajb1 high | Ms4a6b | 3.83E-85 | 2.143872793 | 0.444 | 0.04 | 8.64E-81 |
| T02_Dnajb1 high | Coro1a | 4.86E-82 | 2.791610239 | 0.7 | 0.109 | 1.10E-77 |
| T02_Dnajb1 high | Laptm5 | 2.76E-80 | 2.300228495 | 0.656 | 0.093 | 6.21E-76 |
| T02_Dnajb1 high | Selplg | 8.52E-78 | 2.353593537 | 0.5 | 0.057 | 1.92E-73 |
| T02_Dnajb1 high | Cd37 | 8.45E-76 | 1.707844482 | 0.344 | 0.027 | 1.90E-71 |
| T02_Dnajb1 high | Cd48 | 1.40E-74 | 2.111241758 | 0.422 | 0.042 | 3.16E-70 |
| T02_Dnajb1 high | Btg2 | 4.47E-72 | 3.137433753 | 0.633 | 0.103 | 1.01E-67 |
| T02_Dnajb1 high | AW112010 | 7.08E-69 | 2.801866189 | 0.689 | 0.125 | 1.60E-64 |
| T02_Dnajb1 high | Samsn1 | 2.31E-67 | 1.843736696 | 0.322 | 0.026 | 5.20E-63 |
| T02_Dnajb1 high | Rgs1 | 3.46E-66 | 2.865414982 | 0.756 | 0.156 | 7.80E-62 |
| T02_Dnajb1 high | Lcp1 | 2.71E-65 | 2.297002001 | 0.578 | 0.087 | 6.12E-61 |
| T02_Dnajb1 high | Ccl4 | 3.10E-65 | 4.627431359 | 0.544 | 0.081 | 7.00E-61 |
| T02_Dnajb1 high | Tnfrsf4 | 2.31E-63 | 2.010584671 | 0.256 | 0.018 | 5.21E-59 |
| T02_Dnajb1 high | Dusp5 | 6.33E-63 | 2.587263574 | 0.433 | 0.053 | 1.43E-58 |
| T02_Dnajb1 high | Cish | 2.74E-61 | 1.905546559 | 0.3 | 0.026 | 6.18E-57 |
| T02_Dnajb1 high | Sla | 2.71E-51 | 1.736087934 | 0.256 | 0.022 | 6.10E-47 |
| T02_Dnajb1 high | Tmsb4x | 4.41E-47 | 2.400258177 | 0.989 | 0.983 | 9.94E-43 |
| T02_Dnajb1 high | H2-Q6 | 9.94E-45 | 1.96432217 | 0.311 | 0.037 | 2.24E-40 |
| T02_Dnajb1 high | Nr4a1 | 1.25E-41 | 3.241461809 | 0.556 | 0.133 | 2.81E-37 |
| T02_Dnajb1 high | Cd53 | 1.57E-40 | 1.975130921 | 0.422 | 0.072 | 3.54E-36 |
| T02_Dnajb1 high | Ccl3 | 1.05E-33 | 3.140970689 | 0.333 | 0.054 | 2.36E-29 |
| T02_Dnajb1 high | Snord13 | 2.45E-33 | 1.894559197 | 0.289 | 0.042 | 5.53E-29 |
| T02_Dnajb1 high | Malat1 | 4.10E-32 | 1.901103401 | 0.978 | 0.919 | 9.25E-28 |
| T02_Dnajb1 high | Slfn2 | 2.06E-31 | 2.005835545 | 0.411 | 0.086 | 4.64E-27 |
| T02_Dnajb1 high | Btg1 | 3.21E-30 | 2.629396264 | 0.811 | 0.578 | 7.23E-26 |
| T02_Dnajb1 high | Junb | 3.37E-27 | 2.710935999 | 0.822 | 0.616 | 7.59E-23 |
| T02_Dnajb1 high | Il2rg | 9.97E-24 | 2.167413086 | 0.556 | 0.224 | 2.25E-19 |
| T02_Dnajb1 high | Ucp2 | 2.21E-22 | 1.703161362 | 0.433 | 0.121 | 4.99E-18 |
| T02_Dnajb1 high | Ctla2a | 4.21E-21 | 2.431749295 | 0.444 | 0.15 | 9.48E-17 |
| T02_Dnajb1 high | Stk17b | 4.51E-18 | 2.122348542 | 0.467 | 0.189 | 1.02E-13 |
| T02_Dnajb1 high | Klf6 | 1.14E-15 | 1.977146542 | 0.789 | 0.846 | 2.56E-11 |
| T02_Dnajb1 high | Ifngr1 | 2.27E-15 | 2.521428822 | 0.622 | 0.451 | 5.12E-11 |
| T02_Dnajb1 high | Bhlhe40 | 2.55E-14 | 2.503183825 | 0.522 | 0.291 | 5.75E-10 |
| T02_Dnajb1 high | Hspa1a | 7.14E-14 | 2.267824493 | 0.289 | 0.083 | 1.61E-09 |
| T02_Dnajb1 high | Fos | 1.56E-13 | 1.676469334 | 0.678 | 0.445 | 3.53E-09 |
| T02_Dnajb1 high | Rgs2 | 5.17E-13 | 2.243511777 | 0.344 | 0.124 | 1.17E-08 |
| T02_Dnajb1 high | Ptpn18 | 1.42E-12 | 2.142362568 | 0.5 | 0.288 | 3.20E-08 |
| T02_Dnajb1 high | Dusp1 | 3.15E-11 | 1.783482658 | 0.678 | 0.562 | 7.10E-07 |
| T02_Dnajb1 high | Clec2d | 1.79E-10 | 2.044198942 | 0.5 | 0.328 | 4.04E-06 |
| T02_Dnajb1 high | Zfp36l2 | 2.20E-10 | 1.903561683 | 0.589 | 0.517 | 4.96E-06 |
| T02_Dnajb1 high | Arhgdib | 4.42E-10 | 1.666903609 | 0.656 | 0.703 | 9.95E-06 |
| T02_Dnajb1 high | Pnrc1 | 1.97E-09 | 2.094492726 | 0.511 | 0.379 | 4.45E-05 |
| T02_Dnajb1 high | Zfp36 | 1.46E-06 | 1.948224244 | 0.511 | 0.407 | 0.032907156 |
| T02_Dnajb1 high | Vps37b | 0.000231712 | 1.754758372 | 0.333 | 0.244 | 1 |
| T02_Dnajb1 high | Nfkbia | 0.000313855 | 2.067063606 | 0.578 | 0.726 | 1 |
| T02_Dnajb1 high | Gm26917 | 0.003705549 | 2.453630181 | 0.444 | 0.469 | 1 |
| T03_Dnajb1 low | Nkg7 | 0 | 4.569812856 | 1 | 0.026 | 0 |
| T03_Dnajb1 low | Xcl1 | 0 | 4.166404018 | 0.448 | 0.006 | 0 |
| T03_Dnajb1 low | Ifng | 0 | 3.825106294 | 0.741 | 0.008 | 0 |
| T03_Dnajb1 low | Trbc2 | 0 | 3.714589419 | 0.948 | 0.019 | 0 |
| T03_Dnajb1 low | Ms4a4b | 0 | 3.504010255 | 0.897 | 0.021 | 0 |
| T03_Dnajb1 low | Pdcd1 | 0 | 3.412436276 | 0.724 | 0.007 | 0 |
| T03_Dnajb1 low | Cd3g | 0 | 3.366346661 | 0.862 | 0.017 | 0 |
| T03_Dnajb1 low | Sh2d2a | 0 | 3.244567447 | 0.776 | 0.011 | 0 |
| T03_Dnajb1 low | Klrd1 | 0 | 3.23765759 | 0.793 | 0.013 | 0 |
| T03_Dnajb1 low | Thy1 | 0 | 3.204232969 | 0.862 | 0.018 | 0 |
| T03_Dnajb1 low | Dusp2 | 0 | 3.183203465 | 0.897 | 0.021 | 0 |
| T03_Dnajb1 low | Klrc1 | 0 | 3.164150403 | 0.828 | 0.011 | 0 |
| T03_Dnajb1 low | Cd3d | 0 | 3.162795197 | 0.879 | 0.017 | 0 |
| T03_Dnajb1 low | Trac | 0 | 3.156523334 | 0.879 | 0.021 | 0 |
| T03_Dnajb1 low | Cd3e | 0 | 2.857075889 | 0.81 | 0.014 | 0 |
| T03_Dnajb1 low | Cd8a | 0 | 2.746318365 | 0.759 | 0.011 | 0 |
| T03_Dnajb1 low | Cxcr6 | 0 | 2.702574839 | 0.741 | 0.013 | 0 |
| T03_Dnajb1 low | Klrk1 | 0 | 2.68741545 | 0.862 | 0.015 | 0 |
| T03_Dnajb1 low | Cd8b1 | 0 | 2.68307467 | 0.759 | 0.012 | 0 |
| T03_Dnajb1 low | Il2rb | 0 | 2.647905957 | 0.707 | 0.011 | 0 |
| T03_Dnajb1 low | Ptprcap | 0 | 2.643779275 | 0.776 | 0.015 | 0 |
| T03_Dnajb1 low | Ctsw | 0 | 2.633876328 | 0.741 | 0.018 | 0 |
| T03_Dnajb1 low | Cst7 | 0 | 2.311431233 | 0.569 | 0.007 | 0 |
| T03_Dnajb1 low | Nrgn | 0 | 2.294431138 | 0.552 | 0.007 | 0 |
| T03_Dnajb1 low | Gimap6 | 0 | 2.283093127 | 0.638 | 0.013 | 0 |
| T03_Dnajb1 low | Gzmb | 0 | 2.274559815 | 0.483 | 0.006 | 0 |
| T03_Dnajb1 low | Gm8369 | 0 | 2.230189617 | 0.569 | 0.008 | 0 |
| T03_Dnajb1 low | Lat | 0 | 2.032159098 | 0.603 | 0.012 | 0 |
| T03_Dnajb1 low | Prf1 | 0 | 2.01079681 | 0.448 | 0.004 | 0 |
| T03_Dnajb1 low | Gimap3 | 0 | 1.960124005 | 0.517 | 0.009 | 0 |
| T03_Dnajb1 low | Tnfrsf18 | 0 | 1.934505129 | 0.517 | 0.009 | 0 |
| T03_Dnajb1 low | Ctla4 | 0 | 1.880784231 | 0.397 | 0.003 | 0 |
| T03_Dnajb1 low | Klre1 | 0 | 1.878377173 | 0.552 | 0.005 | 0 |
| T03_Dnajb1 low | Cd28 | 0 | 1.866491518 | 0.569 | 0.005 | 0 |
| T03_Dnajb1 low | Lag3 | 0 | 1.825119506 | 0.483 | 0.007 | 0 |
| T03_Dnajb1 low | Gimap5 | 0 | 1.734045776 | 0.517 | 0.007 | 0 |
| T03_Dnajb1 low | Gimap4 | 5.70E-301 | 1.983069749 | 0.569 | 0.012 | 1.28E-296 |
| T03_Dnajb1 low | Cd2 | 1.37E-274 | 1.921912499 | 0.569 | 0.013 | 3.09E-270 |
| T03_Dnajb1 low | Lck | 2.98E-273 | 2.347708783 | 0.741 | 0.023 | 6.71E-269 |
| T03_Dnajb1 low | Ltb | 4.67E-254 | 2.439994412 | 0.517 | 0.012 | 1.05E-249 |
| T03_Dnajb1 low | Cd69 | 4.82E-246 | 3.260579744 | 0.69 | 0.022 | 1.09E-241 |
| T03_Dnajb1 low | Pglyrp1 | 1.75E-224 | 1.816805716 | 0.517 | 0.013 | 3.96E-220 |
| T03_Dnajb1 low | Tnfaip3 | 5.98E-216 | 2.913600918 | 0.759 | 0.032 | 1.35E-211 |
| T03_Dnajb1 low | Tnfrsf4 | 6.61E-201 | 2.41652928 | 0.552 | 0.017 | 1.49E-196 |
| T03_Dnajb1 low | 1-Sep | 9.96E-174 | 2.112905899 | 0.759 | 0.039 | 2.25E-169 |
| T03_Dnajb1 low | Samsn1 | 4.38E-172 | 2.582181946 | 0.621 | 0.026 | 9.87E-168 |
| T03_Dnajb1 low | Trbc1 | 1.53E-171 | 2.444970391 | 0.414 | 0.011 | 3.44E-167 |
| T03_Dnajb1 low | Rac2 | 3.93E-167 | 3.25105142 | 0.966 | 0.07 | 8.85E-163 |
| T03_Dnajb1 low | Skap1 | 4.95E-163 | 1.657570279 | 0.431 | 0.013 | 1.12E-158 |
| T03_Dnajb1 low | Rgs16 | 4.04E-148 | 3.219315951 | 0.69 | 0.039 | 9.11E-144 |
| T03_Dnajb1 low | Hcst | 1.69E-128 | 2.269378431 | 0.638 | 0.037 | 3.82E-124 |
| T03_Dnajb1 low | Ms4a6b | 1.64E-122 | 2.172979404 | 0.655 | 0.04 | 3.71E-118 |
| T03_Dnajb1 low | Ptprc | 2.00E-122 | 2.735533949 | 0.879 | 0.077 | 4.52E-118 |
| T03_Dnajb1 low | H2-Q7 | 9.29E-119 | 2.369373183 | 0.759 | 0.058 | 2.09E-114 |
| T03_Dnajb1 low | Ccl4 | 1.83E-116 | 5.508433747 | 0.862 | 0.081 | 4.14E-112 |
| T03_Dnajb1 low | Ccl5 | 1.72E-107 | 3.501456076 | 0.707 | 0.055 | 3.88E-103 |
| T03_Dnajb1 low | Sla | 3.21E-106 | 1.633500157 | 0.448 | 0.022 | 7.24E-102 |
| T03_Dnajb1 low | Srgn | 2.61E-101 | 2.81136979 | 0.966 | 0.111 | 5.89E-97 |
| T03_Dnajb1 low | AW112010 | 8.34E-101 | 3.434342914 | 0.983 | 0.125 | 1.88E-96 |
| T03_Dnajb1 low | Gpr183 | 4.19E-100 | 2.222739248 | 0.448 | 0.023 | 9.46E-96 |
| T03_Dnajb1 low | Coro1a | 1.02E-93 | 2.719327566 | 0.914 | 0.109 | 2.30E-89 |
| T03_Dnajb1 low | Cd52 | 4.37E-93 | 3.172680473 | 1 | 0.132 | 9.85E-89 |
| T03_Dnajb1 low | Selplg | 6.26E-92 | 2.067904981 | 0.672 | 0.057 | 1.41E-87 |
| T03_Dnajb1 low | Lcp1 | 1.52E-87 | 2.504543904 | 0.81 | 0.087 | 3.42E-83 |
| T03_Dnajb1 low | Ccl3 | 7.97E-86 | 3.924939422 | 0.621 | 0.053 | 1.80E-81 |
| T03_Dnajb1 low | Gmfg | 2.96E-81 | 1.771359897 | 0.621 | 0.054 | 6.68E-77 |
| T03_Dnajb1 low | Lpxn | 3.58E-80 | 1.63608531 | 0.483 | 0.034 | 8.08E-76 |
| T03_Dnajb1 low | Tnfrsf9 | 8.42E-76 | 2.065293389 | 0.534 | 0.045 | 1.90E-71 |
| T03_Dnajb1 low | Id2 | 1.87E-73 | 2.940866945 | 0.879 | 0.131 | 4.22E-69 |
| T03_Dnajb1 low | Rgs1 | 1.38E-72 | 3.768016933 | 0.931 | 0.156 | 3.10E-68 |
| T03_Dnajb1 low | Cd48 | 9.44E-68 | 1.850818941 | 0.5 | 0.042 | 2.13E-63 |
| T03_Dnajb1 low | Btg2 | 1.84E-64 | 2.717632034 | 0.741 | 0.103 | 4.16E-60 |
| T03_Dnajb1 low | Serpina3g | 2.14E-64 | 2.125042474 | 0.603 | 0.069 | 4.82E-60 |
| T03_Dnajb1 low | Ly6c2 | 9.13E-63 | 2.202274328 | 0.379 | 0.026 | 2.06E-58 |
| T03_Dnajb1 low | Laptm5 | 4.02E-61 | 2.01430745 | 0.724 | 0.093 | 9.07E-57 |
| T03_Dnajb1 low | Cd53 | 3.75E-59 | 1.807186007 | 0.621 | 0.072 | 8.45E-55 |
| T03_Dnajb1 low | Arl6ip1 | 3.19E-56 | 1.774255202 | 0.534 | 0.058 | 7.19E-52 |
| T03_Dnajb1 low | Ptpn22 | 8.59E-53 | 1.703845224 | 0.569 | 0.073 | 1.94E-48 |
| T03_Dnajb1 low | Nr4a1 | 8.24E-50 | 2.880541889 | 0.724 | 0.133 | 1.86E-45 |
| T03_Dnajb1 low | Rgs2 | 6.10E-41 | 2.649688349 | 0.655 | 0.123 | 1.38E-36 |
| T03_Dnajb1 low | Dusp5 | 3.79E-40 | 1.706328111 | 0.431 | 0.054 | 8.53E-36 |
| T03_Dnajb1 low | Ctla2a | 5.60E-38 | 2.55265667 | 0.672 | 0.149 | 1.26E-33 |
| T03_Dnajb1 low | Nr4a2 | 1.30E-37 | 2.078237498 | 0.448 | 0.063 | 2.93E-33 |
| T03_Dnajb1 low | Irf8 | 2.21E-37 | 1.735753962 | 0.448 | 0.062 | 4.99E-33 |
| T03_Dnajb1 low | Tmsb4x | 3.64E-33 | 2.521409242 | 1 | 0.983 | 8.21E-29 |
| T03_Dnajb1 low | Bhlhe40 | 2.31E-31 | 2.863657792 | 0.793 | 0.291 | 5.20E-27 |
| T03_Dnajb1 low | Il2rg | 5.19E-29 | 2.076680324 | 0.707 | 0.224 | 1.17E-24 |
| T03_Dnajb1 low | Ptpn18 | 9.70E-27 | 2.045393914 | 0.759 | 0.288 | 2.19E-22 |
| T03_Dnajb1 low | D16Ertd472e | 6.42E-25 | 1.914057319 | 0.569 | 0.155 | 1.45E-20 |
| T03_Dnajb1 low | Stk17b | 8.03E-23 | 1.701521614 | 0.603 | 0.189 | 1.81E-18 |
| T03_Dnajb1 low | Arhgdib | 1.13E-22 | 1.792931402 | 0.897 | 0.703 | 2.54E-18 |
| T03_Dnajb1 low | Serpinb6b | 1.63E-22 | 1.797714575 | 0.569 | 0.171 | 3.68E-18 |
| T03_Dnajb1 low | Nfil3 | 3.04E-21 | 1.627992164 | 0.569 | 0.175 | 6.86E-17 |
| T03_Dnajb1 low | Junb | 5.77E-21 | 2.098242201 | 0.897 | 0.616 | 1.30E-16 |
| T03_Dnajb1 low | Vps37b | 1.28E-19 | 2.329606204 | 0.621 | 0.243 | 2.89E-15 |
| T03_Dnajb1 low | Btg1 | 3.36E-18 | 2.060767748 | 0.81 | 0.578 | 7.58E-14 |
| T03_Dnajb1 low | Pnrc1 | 5.42E-17 | 1.955161425 | 0.707 | 0.378 | 1.22E-12 |
| T03_Dnajb1 low | Ifngr1 | 1.45E-14 | 1.709319608 | 0.707 | 0.451 | 3.28E-10 |
| T03_Dnajb1 low | Hilpda | 3.96E-08 | 1.760062964 | 0.5 | 0.294 | 0.000893366 |
| T03_Dnajb1 low | Nfkbia | 3.87E-06 | 1.773512554 | 0.69 | 0.725 | 0.08723291 |
| T04_Cd4+ T cells | Penk | 0 | 3.665773267 | 0.371 | 0.001 | 0 |
| T04_Cd4+ T cells | Tnfrsf18 | 0 | 3.476972671 | 0.771 | 0.009 | 0 |
| T04_Cd4+ T cells | Icos | 0 | 3.171835869 | 0.686 | 0.005 | 0 |
| T04_Cd4+ T cells | Cd2 | 0 | 3.077946257 | 0.857 | 0.013 | 0 |
| T04_Cd4+ T cells | Klrg1 | 0 | 3.010808742 | 0.343 | 0.001 | 0 |
| T04_Cd4+ T cells | Ctla4 | 0 | 2.728843231 | 0.571 | 0.003 | 0 |
| T04_Cd4+ T cells | Cd4 | 0 | 2.537655553 | 0.543 | 0.002 | 0 |
| T04_Cd4+ T cells | Cd5 | 0 | 1.980193649 | 0.543 | 0.004 | 0 |
| T04_Cd4+ T cells | Cd3g | 5.46E-302 | 3.553121679 | 0.886 | 0.018 | 1.23E-297 |
| T04_Cd4+ T cells | Foxp3 | 3.08E-253 | 1.748436333 | 0.286 | 0.002 | 6.94E-249 |
| T04_Cd4+ T cells | Cd3e | 7.22E-251 | 3.068349684 | 0.743 | 0.015 | 1.63E-246 |
| T04_Cd4+ T cells | Tnfrsf4 | 1.73E-243 | 4.337382299 | 0.771 | 0.018 | 3.91E-239 |
| T04_Cd4+ T cells | Cd3d | 8.32E-235 | 3.36343599 | 0.771 | 0.018 | 1.88E-230 |
| T04_Cd4+ T cells | Trac | 8.35E-221 | 3.562122382 | 0.829 | 0.022 | 1.88E-216 |
| T04_Cd4+ T cells | Trbc2 | 3.73E-218 | 3.3465228 | 0.8 | 0.021 | 8.40E-214 |
| T04_Cd4+ T cells | Gimap5 | 4.52E-215 | 2.018935279 | 0.486 | 0.007 | 1.02E-210 |
| T04_Cd4+ T cells | Cd6 | 5.55E-205 | 2.037965248 | 0.4 | 0.005 | 1.25E-200 |
| T04_Cd4+ T cells | Ccl1 | 5.18E-193 | 4.103447254 | 0.286 | 0.003 | 1.17E-188 |
| T04_Cd4+ T cells | Trbc1 | 6.89E-163 | 3.443129751 | 0.514 | 0.011 | 1.55E-158 |
| T04_Cd4+ T cells | Thy1 | 2.24E-157 | 2.867385888 | 0.657 | 0.019 | 5.04E-153 |
| T04_Cd4+ T cells | Pdcd1 | 2.99E-156 | 2.113036531 | 0.429 | 0.008 | 6.75E-152 |
| T04_Cd4+ T cells | Gimap6 | 1.60E-153 | 2.332037425 | 0.543 | 0.013 | 3.60E-149 |
| T04_Cd4+ T cells | Lat | 4.12E-150 | 2.209507639 | 0.514 | 0.012 | 9.28E-146 |
| T04_Cd4+ T cells | Lag3 | 8.38E-126 | 1.744634762 | 0.371 | 0.008 | 1.89E-121 |
| T04_Cd4+ T cells | Cst7 | 4.33E-123 | 1.798893133 | 0.371 | 0.008 | 9.75E-119 |
| T04_Cd4+ T cells | Sh2d2a | 2.28E-121 | 2.411366411 | 0.457 | 0.012 | 5.15E-117 |
| T04_Cd4+ T cells | Gimap3 | 1.18E-116 | 1.899174425 | 0.4 | 0.01 | 2.66E-112 |
| T04_Cd4+ T cells | Ptprcap | 6.37E-114 | 2.492827886 | 0.514 | 0.016 | 1.44E-109 |
| T04_Cd4+ T cells | Samsn1 | 7.10E-108 | 2.873864934 | 0.629 | 0.027 | 1.60E-103 |
| T04_Cd4+ T cells | Ltb | 9.86E-106 | 2.493057908 | 0.429 | 0.012 | 2.22E-101 |
| T04_Cd4+ T cells | Cxcr6 | 1.48E-102 | 2.656517644 | 0.457 | 0.014 | 3.34E-98 |
| T04_Cd4+ T cells | Tnfsf8 | 4.84E-102 | 1.819691783 | 0.257 | 0.004 | 1.09E-97 |
| T04_Cd4+ T cells | Ifng | 7.11E-101 | 2.79518461 | 0.371 | 0.01 | 1.60E-96 |
| T04_Cd4+ T cells | Il2rb | 1.63E-93 | 2.160515635 | 0.4 | 0.012 | 3.68E-89 |
| T04_Cd4+ T cells | Gimap4 | 2.32E-90 | 2.25276999 | 0.4 | 0.013 | 5.22E-86 |
| T04_Cd4+ T cells | Rac2 | 2.21E-85 | 3.341811385 | 0.886 | 0.071 | 4.99E-81 |
| T04_Cd4+ T cells | Nkg7 | 1.45E-84 | 3.312848969 | 0.571 | 0.028 | 3.27E-80 |
| T04_Cd4+ T cells | Gzmb | 1.96E-79 | 1.826790574 | 0.286 | 0.007 | 4.42E-75 |
| T04_Cd4+ T cells | Lck | 1.47E-78 | 2.220324086 | 0.514 | 0.024 | 3.32E-74 |
| T04_Cd4+ T cells | Il1r2 | 1.48E-77 | 2.524323937 | 0.371 | 0.013 | 3.33E-73 |
| T04_Cd4+ T cells | Skap1 | 9.23E-74 | 1.880403829 | 0.371 | 0.013 | 2.08E-69 |
| T04_Cd4+ T cells | Nrgn | 1.11E-73 | 2.277163715 | 0.286 | 0.008 | 2.50E-69 |
| T04_Cd4+ T cells | Cd53 | 2.43E-71 | 2.805464323 | 0.829 | 0.072 | 5.47E-67 |
| T04_Cd4+ T cells | Cytip | 4.19E-68 | 1.84607247 | 0.429 | 0.019 | 9.44E-64 |
| T04_Cd4+ T cells | H2-Q7 | 2.93E-65 | 2.822157433 | 0.714 | 0.059 | 6.60E-61 |
| T04_Cd4+ T cells | Srgn | 5.14E-63 | 3.633647036 | 0.943 | 0.112 | 1.16E-58 |
| T04_Cd4+ T cells | Cd37 | 4.94E-61 | 1.841657767 | 0.486 | 0.028 | 1.11E-56 |
| T04_Cd4+ T cells | 1-Sep | 2.83E-59 | 2.282876808 | 0.571 | 0.04 | 6.37E-55 |
| T04_Cd4+ T cells | Hcst | 3.22E-56 | 2.217829712 | 0.543 | 0.038 | 7.26E-52 |
| T04_Cd4+ T cells | Coro1a | 5.67E-54 | 2.79092996 | 0.886 | 0.11 | 1.28E-49 |
| T04_Cd4+ T cells | Ptprc | 2.35E-52 | 2.810283474 | 0.743 | 0.078 | 5.29E-48 |
| T04_Cd4+ T cells | Arl6ip1 | 2.26E-50 | 2.606424219 | 0.629 | 0.058 | 5.09E-46 |
| T04_Cd4+ T cells | Ms4a4b | 3.09E-49 | 2.604059829 | 0.4 | 0.023 | 6.96E-45 |
| T04_Cd4+ T cells | Gmfg | 2.05E-48 | 2.451411748 | 0.6 | 0.054 | 4.62E-44 |
| T04_Cd4+ T cells | Cd52 | 1.12E-47 | 3.288356778 | 0.914 | 0.133 | 2.53E-43 |
| T04_Cd4+ T cells | AW112010 | 1.50E-46 | 3.734687826 | 0.857 | 0.126 | 3.39E-42 |
| T04_Cd4+ T cells | H2-Q6 | 6.72E-46 | 1.958000215 | 0.486 | 0.037 | 1.52E-41 |
| T04_Cd4+ T cells | Lcp1 | 1.92E-45 | 2.421513056 | 0.743 | 0.088 | 4.34E-41 |
| T04_Cd4+ T cells | Ptpn7 | 4.38E-45 | 1.75278451 | 0.371 | 0.022 | 9.88E-41 |
| T04_Cd4+ T cells | Tnfrsf9 | 2.41E-43 | 2.576342574 | 0.514 | 0.045 | 5.43E-39 |
| T04_Cd4+ T cells | Id2 | 4.27E-40 | 2.910337196 | 0.829 | 0.132 | 9.64E-36 |
| T04_Cd4+ T cells | Cd48 | 7.16E-40 | 2.131835784 | 0.486 | 0.043 | 1.61E-35 |
| T04_Cd4+ T cells | Gpr65 | 7.81E-38 | 1.81620221 | 0.286 | 0.016 | 1.76E-33 |
| T04_Cd4+ T cells | Klrc1 | 9.23E-37 | 1.735350088 | 0.257 | 0.013 | 2.08E-32 |
| T04_Cd4+ T cells | Tnfaip3 | 9.63E-35 | 1.858219146 | 0.4 | 0.033 | 2.17E-30 |
| T04_Cd4+ T cells | Laptm5 | 1.87E-34 | 2.305526989 | 0.686 | 0.094 | 4.22E-30 |
| T04_Cd4+ T cells | Dusp5 | 3.65E-32 | 2.085030696 | 0.486 | 0.054 | 8.23E-28 |
| T04_Cd4+ T cells | Rgs1 | 4.73E-29 | 3.744231925 | 0.771 | 0.157 | 1.07E-24 |
| T04_Cd4+ T cells | Cd69 | 1.31E-24 | 1.962686743 | 0.286 | 0.024 | 2.94E-20 |
| T04_Cd4+ T cells | Bcl2a1d | 3.09E-24 | 1.94561842 | 0.371 | 0.04 | 6.97E-20 |
| T04_Cd4+ T cells | Snord13 | 1.50E-23 | 2.641432968 | 0.371 | 0.042 | 3.37E-19 |
| T04_Cd4+ T cells | Il2rg | 2.51E-23 | 2.915882236 | 0.771 | 0.224 | 5.67E-19 |
| T04_Cd4+ T cells | Tmsb4x | 3.67E-21 | 2.654021279 | 1 | 0.983 | 8.28E-17 |
| T04_Cd4+ T cells | Rgs16 | 6.22E-21 | 2.789877942 | 0.343 | 0.04 | 1.40E-16 |
| T04_Cd4+ T cells | Ucp2 | 3.21E-20 | 1.97431009 | 0.6 | 0.122 | 7.23E-16 |
| T04_Cd4+ T cells | Sdf4 | 2.06E-14 | 3.369697898 | 0.829 | 0.591 | 4.64E-10 |
| T04_Cd4+ T cells | Crem | 4.74E-13 | 1.816988258 | 0.4 | 0.087 | 1.07E-08 |
| T04_Cd4+ T cells | Maf | 5.98E-13 | 2.471446219 | 0.543 | 0.169 | 1.35E-08 |
| T04_Cd4+ T cells | Rgs2 | 3.56E-11 | 2.212093292 | 0.457 | 0.124 | 8.02E-07 |
| T04_Cd4+ T cells | Lsp1 | 5.63E-11 | 1.871747026 | 0.743 | 0.435 | 1.27E-06 |
| T04_Cd4+ T cells | Shisa5 | 1.09E-09 | 1.773460314 | 0.771 | 0.588 | 2.47E-05 |
| T04_Cd4+ T cells | H2-T23 | 9.54E-09 | 1.952627357 | 0.714 | 0.461 | 0.000215002 |
| T04_Cd4+ T cells | Ifi27l2a | 1.71E-08 | 2.315177806 | 0.743 | 0.552 | 0.000386367 |
| T04_Cd4+ T cells | Ccl3 | 4.57E-08 | 2.240562809 | 0.257 | 0.055 | 0.001030777 |
| T04_Cd4+ T cells | Junb | 8.32E-08 | 2.868742016 | 0.743 | 0.617 | 0.001875559 |
| T04_Cd4+ T cells | Arhgdib | 8.84E-08 | 1.935408197 | 0.743 | 0.703 | 0.001993703 |
| T04_Cd4+ T cells | Dusp1 | 1.26E-07 | 2.512235509 | 0.743 | 0.562 | 0.002834715 |
| T04_Cd4+ T cells | Ccl5 | 1.48E-07 | 3.282430849 | 0.257 | 0.057 | 0.003346752 |
| T04_Cd4+ T cells | Glrx | 9.35E-07 | 1.964489876 | 0.371 | 0.131 | 0.021086862 |
| T04_Cd4+ T cells | Pnrc1 | 1.01E-06 | 1.865806583 | 0.6 | 0.379 | 0.022734415 |
| T04_Cd4+ T cells | Nr4a1 | 1.68E-06 | 2.520138836 | 0.371 | 0.134 | 0.037768899 |
| T04_Cd4+ T cells | Btg1 | 4.45E-06 | 1.761548409 | 0.686 | 0.579 | 0.100433694 |
| T04_Cd4+ T cells | Bhlhe40 | 4.99E-06 | 2.30513069 | 0.514 | 0.292 | 0.112522052 |
| T04_Cd4+ T cells | Socs1 | 1.22E-05 | 1.973345525 | 0.429 | 0.208 | 0.274702356 |
| T04_Cd4+ T cells | Igfbp7 | 5.29E-05 | 1.913710755 | 0.286 | 0.103 | 1 |
| T04_Cd4+ T cells | Nfkbia | 0.000125758 | 2.558950828 | 0.686 | 0.725 | 1 |
| T04_Cd4+ T cells | Ccnd2 | 0.000152148 | 2.003167501 | 0.514 | 0.363 | 1 |
| T04_Cd4+ T cells | Gadd45b | 0.000186686 | 1.825003402 | 0.286 | 0.115 | 1 |
| T04_Cd4+ T cells | Odc1 | 0.000204607 | 1.742453503 | 0.429 | 0.258 | 1 |
| T04_Cd4+ T cells | Klf6 | 0.002114827 | 1.778983455 | 0.714 | 0.846 | 1 |

**Table S2: GO Enrichment analysis.**

| cluster | ID | Description | GeneRatio | BgRatio | P  value | p.  adjust | qvalue | geneID | Count |
| --- | --- | --- | --- | --- | --- | --- | --- | --- | --- |
| C01 | GO:0006260 | DNA replication | 15/34 | 253/29008 | 1.35E-22 | 9.48E-20 | 4.96E-20 | Mcm2/Mcm6/Mcm3/Gins2/Mcm5/Lig1/Pcna/Mcm4/Mcm7/Cdt1/Rpa2/Rfc2/Tipin/Nasp/Orc6 | 15 |
| C01 | GO:0006261 | DNA-dependent DNA replication | 12/34 | 142/29008 | 5.89E-20 | 2.06E-17 | 1.08E-17 | Mcm2/Mcm6/Mcm3/Mcm5/Lig1/Pcna/Mcm4/Mcm7/Cdt1/Rfc2/Tipin/Orc6 | 12 |
| C01 | GO:0000727 | double-strand break repair via break-induced replication | 7/34 | 12/29008 | 1.24E-18 | 2.89E-16 | 1.51E-16 | Mcm2/Mcm6/Mcm3/Gins2/Mcm5/Mcm4/Mcm7 | 7 |
| C01 | GO:0006270 | DNA replication initiation | 8/34 | 31/29008 | 1.13E-17 | 1.98E-15 | 1.04E-15 | Mcm2/Mcm6/Mcm3/Mcm5/Mcm4/Mcm7/Cdt1/Orc6 | 8 |
| C01 | GO:0033260 | nuclear DNA replication | 7/34 | 33/29008 | 6.57E-15 | 9.19E-13 | 4.81E-13 | Mcm2/Mcm6/Mcm3/Lig1/Pcna/Mcm4/Cdt1 | 7 |
| C01 | GO:0044786 | cell cycle DNA replication | 7/34 | 38/29008 | 1.93E-14 | 2.25E-12 | 1.18E-12 | Mcm2/Mcm6/Mcm3/Lig1/Pcna/Mcm4/Cdt1 | 7 |
| C01 | GO:0006281 | DNA repair | 12/34 | 495/29008 | 2.08E-13 | 2.08E-11 | 1.09E-11 | Mcm2/Mcm6/Mcm3/Gins2/Mcm5/Lig1/Pcna/Mcm4/Mcm7/Rpa2/Rfc2/Hmga1 | 12 |
| C01 | GO:0006271 | DNA strand elongation involved in DNA replication | 5/34 | 13/29008 | 2.08E-12 | 1.82E-10 | 9.52E-11 | Mcm3/Lig1/Pcna/Mcm4/Mcm7 | 5 |
| C01 | GO:0000724 | double-strand break repair via homologous recombination | 8/34 | 135/29008 | 2.93E-12 | 2.10E-10 | 1.10E-10 | Mcm2/Mcm6/Mcm3/Gins2/Mcm5/Mcm4/Mcm7/Rpa2 | 8 |
| C01 | GO:1902969 | mitotic DNA replication | 5/34 | 14/29008 | 3.23E-12 | 2.10E-10 | 1.10E-10 | Mcm2/Mcm6/Mcm3/Lig1/Mcm4 | 5 |
| C01 | GO:0000725 | recombinational repair | 8/34 | 137/29008 | 3.30E-12 | 2.10E-10 | 1.10E-10 | Mcm2/Mcm6/Mcm3/Gins2/Mcm5/Mcm4/Mcm7/Rpa2 | 8 |
| C01 | GO:0032508 | DNA duplex unwinding | 6/34 | 37/29008 | 3.68E-12 | 2.15E-10 | 1.12E-10 | Mcm2/Mcm6/Mcm3/Mcm5/Mcm4/Mcm7 | 6 |
| C01 | GO:0006302 | double-strand break repair | 9/34 | 244/29008 | 7.94E-12 | 4.28E-10 | 2.24E-10 | Mcm2/Mcm6/Mcm3/Gins2/Mcm5/Lig1/Mcm4/Mcm7/Rpa2 | 9 |
| C01 | GO:0032392 | DNA geometric change | 6/34 | 44/29008 | 1.11E-11 | 5.56E-10 | 2.91E-10 | Mcm2/Mcm6/Mcm3/Mcm5/Mcm4/Mcm7 | 6 |
| C01 | GO:0022616 | DNA strand elongation | 5/34 | 21/29008 | 3.27E-11 | 1.52E-09 | 7.97E-10 | Mcm3/Lig1/Pcna/Mcm4/Mcm7 | 5 |
| C01 | GO:0006310 | DNA recombination | 9/34 | 301/29008 | 5.17E-11 | 2.26E-09 | 1.18E-09 | Mcm2/Mcm6/Mcm3/Gins2/Mcm5/Lig1/Mcm4/Mcm7/Rpa2 | 9 |
| C01 | GO:0071103 | DNA conformation change | 8/34 | 203/29008 | 7.78E-11 | 3.20E-09 | 1.68E-09 | Mcm2/Mcm6/Mcm3/Mcm5/Hells/Mcm4/Mcm7/Nasp | 8 |
| C01 | GO:0006268 | DNA unwinding involved in DNA replication | 4/34 | 14/29008 | 1.56E-09 | 6.07E-08 | 3.18E-08 | Mcm2/Mcm6/Mcm4/Mcm7 | 4 |
| C01 | GO:0006284 | base-excision repair | 4/34 | 40/29008 | 1.39E-07 | 5.14E-06 | 2.69E-06 | Lig1/Pcna/Rpa2/Hmga1 | 4 |
| C01 | GO:0090329 | regulation of DNA-dependent DNA replication | 4/34 | 46/29008 | 2.48E-07 | 8.67E-06 | 4.54E-06 | Pcna/Cdt1/Rfc2/Tipin | 4 |
| C01 | GO:0009147 | pyrimidine nucleoside triphosphate metabolic process | 3/34 | 19/29008 | 1.41E-06 | 4.69E-05 | 2.45E-05 | Dctpp1/Nme1/Dut | 3 |
| C01 | GO:0009219 | pyrimidine deoxyribonucleotide metabolic process | 3/34 | 22/29008 | 2.23E-06 | 7.10E-05 | 3.72E-05 | Dctpp1/Nme1/Dut | 3 |
| C01 | GO:0006275 | regulation of DNA replication | 4/34 | 110/29008 | 8.31E-06 | 0.000253054 | 0.000132425 | Pcna/Cdt1/Rfc2/Tipin | 4 |
| C01 | GO:0031570 | DNA integrity checkpoint signaling | 4/34 | 117/29008 | 1.06E-05 | 0.000309624 | 0.000162029 | Cdt1/Rpa2/Tipin/Ccnd1 | 4 |
| C01 | GO:0006220 | pyrimidine nucleotide metabolic process | 3/34 | 41/29008 | 1.52E-05 | 0.000425914 | 0.000222884 | Dctpp1/Nme1/Dut | 3 |
| C01 | GO:0009124 | nucleoside monophosphate biosynthetic process | 3/34 | 43/29008 | 1.76E-05 | 0.000463879 | 0.000242752 | Nme1/Dut/Paics | 3 |
| C01 | GO:0009394 | 2'-deoxyribonucleotide metabolic process | 3/34 | 44/29008 | 1.89E-05 | 0.000463879 | 0.000242752 | Dctpp1/Nme1/Dut | 3 |
| C01 | GO:0019692 | deoxyribose phosphate metabolic process | 3/34 | 44/29008 | 1.89E-05 | 0.000463879 | 0.000242752 | Dctpp1/Nme1/Dut | 3 |
| C01 | GO:0007093 | mitotic cell cycle checkpoint signaling | 4/34 | 136/29008 | 1.92E-05 | 0.000463879 | 0.000242752 | Cdt1/Rpa2/Tipin/Ccnd1 | 4 |
| C01 | GO:0065004 | protein-DNA complex assembly | 4/34 | 145/29008 | 2.47E-05 | 0.000576732 | 0.000301809 | Mcm2/Hells/Cdt1/Nasp | 4 |
| C01 | GO:0009262 | deoxyribonucleotide metabolic process | 3/34 | 49/29008 | 2.61E-05 | 0.000589853 | 0.000308675 | Dctpp1/Nme1/Dut | 3 |
| C01 | GO:0072527 | pyrimidine-containing compound metabolic process | 3/34 | 57/29008 | 4.12E-05 | 0.000901703 | 0.000471869 | Dctpp1/Nme1/Dut | 3 |
| C01 | GO:0071897 | DNA biosynthetic process | 4/34 | 170/29008 | 4.60E-05 | 0.000976358 | 0.000510936 | Lig1/Pcna/Rfc2/Nhp2 | 4 |
| C01 | GO:0000075 | cell cycle checkpoint signaling | 4/34 | 175/29008 | 5.15E-05 | 0.001060838 | 0.000555145 | Cdt1/Rpa2/Tipin/Ccnd1 | 4 |
| C01 | GO:0009157 | deoxyribonucleoside monophosphate biosynthetic process | 2/34 | 10/29008 | 5.97E-05 | 0.001098863 | 0.000575044 | Nme1/Dut | 2 |
| C01 | GO:0010216 | maintenance of DNA methylation | 2/34 | 10/29008 | 5.97E-05 | 0.001098863 | 0.000575044 | Hells/Dnmt1 | 2 |
| C01 | GO:1900262 | regulation of DNA-directed DNA polymerase activity | 2/34 | 10/29008 | 5.97E-05 | 0.001098863 | 0.000575044 | Pcna/Rfc2 | 2 |
| C01 | GO:1900264 | positive regulation of DNA-directed DNA polymerase activity | 2/34 | 10/29008 | 5.97E-05 | 0.001098863 | 0.000575044 | Pcna/Rfc2 | 2 |
| C01 | GO:0009123 | nucleoside monophosphate metabolic process | 3/34 | 67/29008 | 6.69E-05 | 0.001201651 | 0.000628834 | Nme1/Dut/Paics | 3 |
| C01 | GO:0009221 | pyrimidine deoxyribonucleotide biosynthetic process | 2/34 | 11/29008 | 7.29E-05 | 0.001230728 | 0.00064405 | Nme1/Dut | 2 |
| C01 | GO:0031055 | chromatin remodeling at centromere | 2/34 | 11/29008 | 7.29E-05 | 0.001230728 | 0.00064405 | Hells/Nasp | 2 |
| C01 | GO:0071824 | protein-DNA complex subunit organization | 4/34 | 192/29008 | 7.38E-05 | 0.001230728 | 0.00064405 | Mcm2/Hells/Cdt1/Nasp | 4 |
| C01 | GO:0051052 | regulation of DNA metabolic process | 5/34 | 388/29008 | 8.44E-05 | 0.001373314 | 0.000718667 | Pcna/Cdt1/Rpa2/Rfc2/Dnmt1 | 5 |
| C01 | GO:0009223 | pyrimidine deoxyribonucleotide catabolic process | 2/34 | 12/29008 | 8.74E-05 | 0.001389848 | 0.000727319 | Dctpp1/Dut | 2 |
| C01 | GO:0009143 | nucleoside triphosphate catabolic process | 2/34 | 13/29008 | 0.00010317 | 0.001604866 | 0.000839839 | Dctpp1/Dut | 2 |
| C01 | GO:0044773 | mitotic DNA damage checkpoint signaling | 3/34 | 79/29008 | 0.00010946 | 0.001665689 | 0.000871669 | Rpa2/Tipin/Ccnd1 | 3 |
| C01 | GO:0046073 | dTMP metabolic process | 2/34 | 14/29008 | 0.000120276 | 0.001791352 | 0.000937429 | Nme1/Dut | 2 |
| C01 | GO:0044774 | mitotic DNA integrity checkpoint signaling | 3/34 | 83/29008 | 0.000126773 | 0.001848778 | 0.000967481 | Rpa2/Tipin/Ccnd1 | 3 |
| C01 | GO:0006244 | pyrimidine nucleotide catabolic process | 2/34 | 15/29008 | 0.000138679 | 0.001866827 | 0.000976926 | Dctpp1/Dut | 2 |
| C01 | GO:0009130 | pyrimidine nucleoside monophosphate biosynthetic process | 2/34 | 15/29008 | 0.000138679 | 0.001866827 | 0.000976926 | Nme1/Dut | 2 |
| C02 | GO:0007059 | chromosome segregation | 15/42 | 338/29008 | 5.39E-19 | 5.48E-16 | 3.64E-16 | Cdc20/Racgap1/Cenpe/Mki67/Cenpf/Cdca8/Rad21/Kif23/Bub1b/Sfpq/Knstrn/Ran/Tacc3/Cenpw/Nup37 | 15 |
| C02 | GO:0000280 | nuclear division | 14/42 | 442/29008 | 1.06E-15 | 5.40E-13 | 3.58E-13 | Ccnb2/Cdc20/Tpx2/Racgap1/Cenpe/Mki67/Cdca8/Rad21/Kif23/Bub1b/Knstrn/Ran/Tacc3/Cks2 | 14 |
| C02 | GO:0000819 | sister chromatid segregation | 11/42 | 188/29008 | 2.27E-15 | 7.68E-13 | 5.10E-13 | Cdc20/Racgap1/Cenpe/Cdca8/Rad21/Kif23/Bub1b/Sfpq/Knstrn/Ran/Tacc3 | 11 |
| C02 | GO:0098813 | nuclear chromosome segregation | 12/42 | 273/29008 | 3.26E-15 | 8.24E-13 | 5.47E-13 | Cdc20/Racgap1/Cenpe/Cenpf/Cdca8/Rad21/Kif23/Bub1b/Sfpq/Knstrn/Ran/Tacc3 | 12 |
| C02 | GO:0140014 | mitotic nuclear division | 12/42 | 278/29008 | 4.05E-15 | 8.24E-13 | 5.47E-13 | Cdc20/Tpx2/Racgap1/Cenpe/Mki67/Cdca8/Rad21/Kif23/Bub1b/Knstrn/Ran/Tacc3 | 12 |
| C02 | GO:0048285 | organelle fission | 14/42 | 495/29008 | 5.05E-15 | 8.56E-13 | 5.68E-13 | Ccnb2/Cdc20/Tpx2/Racgap1/Cenpe/Mki67/Cdca8/Rad21/Kif23/Bub1b/Knstrn/Ran/Tacc3/Cks2 | 14 |
| C02 | GO:0000070 | mitotic sister chromatid segregation | 10/42 | 159/29008 | 2.33E-14 | 3.38E-12 | 2.24E-12 | Cdc20/Racgap1/Cenpe/Cdca8/Rad21/Kif23/Bub1b/Knstrn/Ran/Tacc3 | 10 |
| C02 | GO:0007051 | spindle organization | 9/42 | 183/29008 | 4.84E-12 | 6.15E-10 | 4.08E-10 | Ccnb2/Cdc20/Tpx2/Racgap1/Cenpe/Kif23/Knstrn/Tacc3/Ckap5 | 9 |
| C02 | GO:1902850 | microtubule cytoskeleton organization involved in mitosis | 8/42 | 146/29008 | 3.47E-11 | 3.92E-09 | 2.60E-09 | Cenpa/Cdc20/Tpx2/Racgap1/Cenpe/Kif23/Tacc3/Ckap5 | 8 |
| C02 | GO:0007052 | mitotic spindle organization | 7/42 | 119/29008 | 3.92E-10 | 3.98E-08 | 2.64E-08 | Cdc20/Tpx2/Racgap1/Cenpe/Kif23/Tacc3/Ckap5 | 7 |
| C02 | GO:0033044 | regulation of chromosome organization | 7/42 | 185/29008 | 8.56E-09 | 7.91E-07 | 5.25E-07 | Cenpe/Hnrnpa2b1/Lmna/Rad21/Bub1b/Sfpq/Tacc3 | 7 |
| C02 | GO:0051225 | spindle assembly | 6/42 | 115/29008 | 1.59E-08 | 1.35E-06 | 8.93E-07 | Ccnb2/Cdc20/Tpx2/Racgap1/Cenpe/Kif23 | 6 |
| C02 | GO:0000281 | mitotic cytokinesis | 5/42 | 67/29008 | 4.50E-08 | 3.52E-06 | 2.33E-06 | Cenpa/Racgap1/Kif20a/Ckap2/Kif23 | 5 |
| C02 | GO:0140694 | non-membrane-bounded organelle assembly | 8/42 | 373/29008 | 5.59E-08 | 4.06E-06 | 2.69E-06 | Ccnb2/Cenpa/Cdc20/Tpx2/Racgap1/Cenpe/Kif23/Cenpw | 8 |
| C02 | GO:0051983 | regulation of chromosome segregation | 5/42 | 86/29008 | 1.59E-07 | 1.08E-05 | 7.15E-06 | Cenpe/Mki67/Rad21/Bub1b/Tacc3 | 5 |
| C02 | GO:0061640 | cytoskeleton-dependent cytokinesis | 5/42 | 93/29008 | 2.35E-07 | 1.50E-05 | 9.92E-06 | Cenpa/Racgap1/Kif20a/Ckap2/Kif23 | 5 |
| C02 | GO:0071824 | protein-DNA complex subunit organization | 6/42 | 192/29008 | 3.35E-07 | 2.00E-05 | 1.33E-05 | Cenpa/Cenpe/Hmgb1/Anp32e/Hp1bp3/Cenpw | 6 |
| C02 | GO:0140013 | meiotic nuclear division | 6/42 | 200/29008 | 4.25E-07 | 2.40E-05 | 1.59E-05 | Ccnb2/Cdc20/Cenpe/Rad21/Bub1b/Cks2 | 6 |
| C02 | GO:0051321 | meiotic cell cycle | 7/42 | 333/29008 | 4.71E-07 | 2.52E-05 | 1.67E-05 | Ccnb2/Cdc20/Cenpe/Mki67/Rad21/Bub1b/Cks2 | 7 |
| C02 | GO:1903046 | meiotic cell cycle process | 6/42 | 220/29008 | 7.42E-07 | 3.77E-05 | 2.50E-05 | Ccnb2/Cdc20/Cenpe/Rad21/Bub1b/Cks2 | 6 |
| C02 | GO:0051988 | regulation of attachment of spindle microtubules to kinetochore | 3/42 | 14/29008 | 1.02E-06 | 4.92E-05 | 3.26E-05 | Racgap1/Cenpe/Knstrn | 3 |
| C02 | GO:0007062 | sister chromatid cohesion | 4/42 | 55/29008 | 1.23E-06 | 5.67E-05 | 3.76E-05 | Cdc20/Rad21/Bub1b/Sfpq | 4 |
| C02 | GO:0044772 | mitotic cell cycle phase transition | 7/42 | 406/29008 | 1.77E-06 | 7.51E-05 | 4.98E-05 | Ccnb2/Cenpe/Cenpf/Rad21/Bub1b/Tacc3/Cks2 | 7 |
| C02 | GO:0007091 | metaphase/anaphase transition of mitotic cell cycle | 4/42 | 61/29008 | 1.87E-06 | 7.51E-05 | 4.98E-05 | Cenpe/Rad21/Bub1b/Tacc3 | 4 |
| C02 | GO:0051382 | kinetochore assembly | 3/42 | 17/29008 | 1.89E-06 | 7.51E-05 | 4.98E-05 | Cenpa/Cenpe/Cenpw | 3 |
| C02 | GO:0010965 | regulation of mitotic sister chromatid separation | 4/42 | 62/29008 | 1.99E-06 | 7.51E-05 | 4.98E-05 | Cenpe/Rad21/Bub1b/Tacc3 | 4 |
| C02 | GO:0051783 | regulation of nuclear division | 5/42 | 143/29008 | 1.99E-06 | 7.51E-05 | 4.98E-05 | Cdc20/Cenpe/Mki67/Rad21/Bub1b | 5 |
| C02 | GO:0065004 | protein-DNA complex assembly | 5/42 | 145/29008 | 2.13E-06 | 7.75E-05 | 5.14E-05 | Cenpa/Cenpe/Hmgb1/Hp1bp3/Cenpw | 5 |
| C02 | GO:0044784 | metaphase/anaphase transition of cell cycle | 4/42 | 64/29008 | 2.26E-06 | 7.94E-05 | 5.27E-05 | Cenpe/Rad21/Bub1b/Tacc3 | 4 |
| C02 | GO:0051306 | mitotic sister chromatid separation | 4/42 | 65/29008 | 2.41E-06 | 8.17E-05 | 5.42E-05 | Cenpe/Rad21/Bub1b/Tacc3 | 4 |
| C02 | GO:0090307 | mitotic spindle assembly | 4/42 | 66/29008 | 2.56E-06 | 8.41E-05 | 5.58E-05 | Cdc20/Tpx2/Racgap1/Kif23 | 4 |
| C02 | GO:1905818 | regulation of chromosome separation | 4/42 | 68/29008 | 2.89E-06 | 9.18E-05 | 6.09E-05 | Cenpe/Rad21/Bub1b/Tacc3 | 4 |
| C02 | GO:0033045 | regulation of sister chromatid segregation | 4/42 | 69/29008 | 3.06E-06 | 9.44E-05 | 6.27E-05 | Cenpe/Rad21/Bub1b/Tacc3 | 4 |
| C02 | GO:0007346 | regulation of mitotic cell cycle | 7/42 | 462/29008 | 4.14E-06 | 0.000123822 | 8.22E-05 | Cenpe/Mki67/Hmgb1/Cenpf/Rad21/Bub1b/Cks2 | 7 |
| C02 | GO:0051383 | kinetochore organization | 3/42 | 22/29008 | 4.26E-06 | 0.00012389 | 8.22E-05 | Cenpa/Cenpe/Cenpw | 3 |
| C02 | GO:0006325 | chromatin organization | 7/42 | 477/29008 | 5.10E-06 | 0.000144149 | 9.56E-05 | Mki67/Hmgb1/Nucks1/Anp32e/Hmgn2/Hp1bp3/Hmgn3 | 7 |
| C02 | GO:0044770 | cell cycle phase transition | 7/42 | 492/29008 | 6.25E-06 | 0.000171674 | 0.000113898 | Ccnb2/Cenpe/Cenpf/Rad21/Bub1b/Tacc3/Cks2 | 7 |
| C02 | GO:0000910 | cytokinesis | 5/42 | 182/29008 | 6.48E-06 | 0.00017354 | 0.000115136 | Cenpa/Racgap1/Kif20a/Ckap2/Kif23 | 5 |
| C02 | GO:0050000 | chromosome localization | 4/42 | 84/29008 | 6.73E-06 | 0.000175576 | 0.000116487 | Cenpe/Cenpf/Lmna/Cdca8 | 4 |
| C02 | GO:0045787 | positive regulation of cell cycle | 6/42 | 336/29008 | 8.53E-06 | 0.000216761 | 0.000143812 | Racgap1/Cenpe/Hmgb1/Rad21/Kif23/Sfpq | 6 |
| C02 | GO:0034508 | centromere complex assembly | 3/42 | 30/29008 | 1.12E-05 | 0.000276581 | 0.0001835 | Cenpa/Cenpe/Cenpw | 3 |
| C02 | GO:0051304 | chromosome separation | 4/42 | 96/29008 | 1.14E-05 | 0.000277186 | 0.000183901 | Cenpe/Rad21/Bub1b/Tacc3 | 4 |
| C02 | GO:0010639 | negative regulation of organelle organization | 6/42 | 364/29008 | 1.34E-05 | 0.000317451 | 0.000210616 | Tpx2/Cenpe/Lmna/Rad21/Ckap2/Bub1b | 6 |
| C02 | GO:0008608 | attachment of spindle microtubules to kinetochore | 3/42 | 33/29008 | 1.49E-05 | 0.000345294 | 0.000229088 | Racgap1/Cenpe/Knstrn | 3 |
| C02 | GO:0007088 | regulation of mitotic nuclear division | 4/42 | 110/29008 | 1.96E-05 | 0.000443061 | 0.000293952 | Cenpe/Mki67/Rad21/Bub1b | 4 |
| C02 | GO:0051656 | establishment of organelle localization | 6/42 | 415/29008 | 2.81E-05 | 0.000620368 | 0.000411588 | Cenpa/Cenpe/Cenpf/Cdca8/Ran/Tacc3 | 6 |
| C02 | GO:0090068 | positive regulation of cell cycle process | 5/42 | 249/29008 | 2.94E-05 | 0.00063577 | 0.000421807 | Racgap1/Cenpe/Rad21/Kif23/Sfpq | 5 |
| C02 | GO:0045841 | negative regulation of mitotic metaphase/anaphase transition | 3/42 | 42/29008 | 3.11E-05 | 0.000659979 | 0.000437868 | Cenpe/Rad21/Bub1b | 3 |
| C02 | GO:0033046 | negative regulation of sister chromatid segregation | 3/42 | 43/29008 | 3.35E-05 | 0.000667071 | 0.000442574 | Cenpe/Rad21/Bub1b | 3 |
| C02 | GO:0033048 | negative regulation of mitotic sister chromatid segregation | 3/42 | 43/29008 | 3.35E-05 | 0.000667071 | 0.000442574 | Cenpe/Rad21/Bub1b | 3 |
| C03 | GO:0000280 | nuclear division | 26/45 | 442/29008 | 5.09E-36 | 3.37E-33 | 1.64E-33 | Ube2c/Prc1/Cdc20/Ccnb1/Ccnb2/Nusap1/Tpx2/Birc5/Cks2/Cenpe/Cdca8/Top2a/Plk1/Racgap1/Aurka/Tacc3/Mki67/Smc4/Kif2c/Kif23/Aspm/Nek2/Smc2/Knstrn/Kif20b/Bub3 | 26 |
| C03 | GO:0048285 | organelle fission | 26/45 | 495/29008 | 1.01E-34 | 3.35E-32 | 1.63E-32 | Ube2c/Prc1/Cdc20/Ccnb1/Ccnb2/Nusap1/Tpx2/Birc5/Cks2/Cenpe/Cdca8/Top2a/Plk1/Racgap1/Aurka/Tacc3/Mki67/Smc4/Kif2c/Kif23/Aspm/Nek2/Smc2/Knstrn/Kif20b/Bub3 | 26 |
| C03 | GO:0140014 | mitotic nuclear division | 22/45 | 278/29008 | 5.71E-33 | 1.26E-30 | 6.13E-31 | Ube2c/Prc1/Cdc20/Ccnb1/Nusap1/Tpx2/Birc5/Cenpe/Cdca8/Plk1/Racgap1/Aurka/Tacc3/Mki67/Smc4/Kif2c/Kif23/Nek2/Smc2/Knstrn/Kif20b/Bub3 | 22 |
| C03 | GO:0007059 | chromosome segregation | 22/45 | 338/29008 | 4.69E-31 | 7.74E-29 | 3.77E-29 | Ube2c/Prc1/Cenpf/Cdc20/Ccnb1/Nusap1/Birc5/Cenpe/Cdca8/Top2a/Plk1/Racgap1/Tacc3/Mki67/Smc4/Kif2c/Kif23/Nek2/Smc2/Hjurp/Knstrn/Bub3 | 22 |
| C03 | GO:0000819 | sister chromatid segregation | 19/45 | 188/29008 | 2.18E-30 | 2.89E-28 | 1.41E-28 | Ube2c/Prc1/Cdc20/Ccnb1/Nusap1/Birc5/Cenpe/Cdca8/Top2a/Plk1/Racgap1/Tacc3/Smc4/Kif2c/Kif23/Nek2/Smc2/Knstrn/Bub3 | 19 |
| C03 | GO:0000070 | mitotic sister chromatid segregation | 18/45 | 159/29008 | 1.12E-29 | 1.23E-27 | 6.00E-28 | Ube2c/Prc1/Cdc20/Ccnb1/Nusap1/Birc5/Cenpe/Cdca8/Plk1/Racgap1/Tacc3/Smc4/Kif2c/Kif23/Nek2/Smc2/Knstrn/Bub3 | 18 |
| C03 | GO:0098813 | nuclear chromosome segregation | 20/45 | 273/29008 | 3.77E-29 | 3.56E-27 | 1.74E-27 | Ube2c/Prc1/Cenpf/Cdc20/Ccnb1/Nusap1/Birc5/Cenpe/Cdca8/Top2a/Plk1/Racgap1/Tacc3/Smc4/Kif2c/Kif23/Nek2/Smc2/Knstrn/Bub3 | 20 |
| C03 | GO:0007051 | spindle organization | 14/45 | 183/29008 | 1.34E-20 | 1.11E-18 | 5.40E-19 | Prc1/Cdc20/Ccnb1/Ccnb2/Tpx2/Cenpe/Plk1/Racgap1/Aurka/Tacc3/Kif23/Aspm/Nek2/Knstrn | 14 |
| C03 | GO:1902850 | microtubule cytoskeleton organization involved in mitosis | 13/45 | 146/29008 | 4.90E-20 | 3.60E-18 | 1.75E-18 | Prc1/Cdc20/Ccnb1/Cenpa/Nusap1/Tpx2/Cenpe/Plk1/Racgap1/Aurka/Tacc3/Kif23/Nek2 | 13 |
| C03 | GO:0000281 | mitotic cytokinesis | 10/45 | 67/29008 | 6.40E-18 | 4.23E-16 | 2.06E-16 | Cenpa/Nusap1/Birc5/Plk1/Racgap1/Ckap2/Anln/Kif20a/Kif23/Kif20b | 10 |
| C03 | GO:0051225 | spindle assembly | 11/45 | 115/29008 | 2.11E-17 | 1.27E-15 | 6.18E-16 | Prc1/Cdc20/Ccnb2/Tpx2/Cenpe/Plk1/Racgap1/Aurka/Kif23/Aspm/Nek2 | 11 |
| C03 | GO:0007052 | mitotic spindle organization | 11/45 | 119/29008 | 3.11E-17 | 1.71E-15 | 8.36E-16 | Prc1/Cdc20/Ccnb1/Tpx2/Cenpe/Plk1/Racgap1/Aurka/Tacc3/Kif23/Nek2 | 11 |
| C03 | GO:0000910 | cytokinesis | 12/45 | 182/29008 | 6.19E-17 | 3.15E-15 | 1.53E-15 | Prc1/Cenpa/Nusap1/Birc5/Plk1/Racgap1/Ckap2/Anln/Kif20a/Aurka/Kif23/Kif20b | 12 |
| C03 | GO:0061640 | cytoskeleton-dependent cytokinesis | 10/45 | 93/29008 | 2.03E-16 | 9.57E-15 | 4.66E-15 | Cenpa/Nusap1/Birc5/Plk1/Racgap1/Ckap2/Anln/Kif20a/Kif23/Kif20b | 10 |
| C03 | GO:0051321 | meiotic cell cycle | 13/45 | 333/29008 | 2.50E-15 | 1.10E-13 | 5.36E-14 | Cdc20/Ccnb2/Cks2/Cenpe/Top2a/Plk1/Aurka/Mki67/Smc4/Aspm/Nek2/Smc2/Bub3 | 13 |
| C03 | GO:0051983 | regulation of chromosome segregation | 9/45 | 86/29008 | 9.34E-15 | 3.86E-13 | 1.88E-13 | Ube2c/Ccnb1/Birc5/Cenpe/Plk1/Tacc3/Mki67/Kif2c/Bub3 | 9 |
| C03 | GO:0044770 | cell cycle phase transition | 14/45 | 492/29008 | 1.40E-14 | 5.45E-13 | 2.65E-13 | Ube2c/Cenpf/Ccnb1/Ccnb2/Birc5/Cks2/Cenpe/Cdk1/Ccna2/Plk1/Aurka/Tacc3/Cks1b/Bub3 | 14 |
| C03 | GO:0044772 | mitotic cell cycle phase transition | 13/45 | 406/29008 | 3.18E-14 | 1.17E-12 | 5.69E-13 | Ube2c/Cenpf/Ccnb1/Ccnb2/Birc5/Cks2/Cenpe/Cdk1/Ccna2/Plk1/Tacc3/Cks1b/Bub3 | 13 |
| C03 | GO:0008608 | attachment of spindle microtubules to kinetochore | 7/45 | 33/29008 | 5.49E-14 | 1.91E-12 | 9.31E-13 | Ccnb1/Cenpe/Racgap1/Kif2c/Nek2/Knstrn/Bub3 | 7 |
| C03 | GO:0007346 | regulation of mitotic cell cycle | 13/45 | 462/29008 | 1.65E-13 | 5.45E-12 | 2.65E-12 | Ube2c/Cenpf/Ccnb1/Nusap1/Birc5/Cks2/Cenpe/Cdk1/Plk1/Mki67/Cks1b/Kif20b/Bub3 | 13 |
| C03 | GO:0140694 | non-membrane-bounded organelle assembly | 12/45 | 373/29008 | 3.36E-13 | 1.06E-11 | 5.16E-12 | Prc1/Cdc20/Ccnb2/Cenpa/Tpx2/Cenpe/Plk1/Racgap1/Aurka/Kif23/Aspm/Nek2 | 12 |
| C03 | GO:0140013 | meiotic nuclear division | 10/45 | 200/29008 | 5.01E-13 | 1.50E-11 | 7.33E-12 | Cdc20/Ccnb2/Cks2/Cenpe/Top2a/Plk1/Aurka/Smc4/Aspm/Smc2 | 10 |
| C03 | GO:0051783 | regulation of nuclear division | 9/45 | 143/29008 | 1.02E-12 | 2.92E-11 | 1.42E-11 | Cdc20/Ccnb1/Nusap1/Birc5/Cenpe/Plk1/Mki67/Kif20b/Bub3 | 9 |
| C03 | GO:1903046 | meiotic cell cycle process | 10/45 | 220/29008 | 1.30E-12 | 3.57E-11 | 1.74E-11 | Cdc20/Ccnb2/Cks2/Cenpe/Top2a/Plk1/Aurka/Smc4/Aspm/Smc2 | 10 |
| C03 | GO:0051304 | chromosome separation | 8/45 | 96/29008 | 2.08E-12 | 5.51E-11 | 2.68E-11 | Ube2c/Ccnb1/Birc5/Cenpe/Top2a/Plk1/Tacc3/Bub3 | 8 |
| C03 | GO:0090068 | positive regulation of cell cycle process | 10/45 | 249/29008 | 4.44E-12 | 1.13E-10 | 5.50E-11 | Ube2c/Ccnb1/Nusap1/Birc5/Cenpe/Cdk1/Racgap1/Aurka/Kif23/Kif20b | 10 |
| C03 | GO:0007091 | metaphase/anaphase transition of mitotic cell cycle | 7/45 | 61/29008 | 5.43E-12 | 1.33E-10 | 6.48E-11 | Ube2c/Ccnb1/Birc5/Cenpe/Plk1/Tacc3/Bub3 | 7 |
| C03 | GO:0010965 | regulation of mitotic sister chromatid separation | 7/45 | 62/29008 | 6.11E-12 | 1.44E-10 | 7.03E-11 | Ube2c/Ccnb1/Birc5/Cenpe/Plk1/Tacc3/Bub3 | 7 |
| C03 | GO:0007088 | regulation of mitotic nuclear division | 8/45 | 110/29008 | 6.33E-12 | 1.44E-10 | 7.03E-11 | Ccnb1/Nusap1/Birc5/Cenpe/Plk1/Mki67/Kif20b/Bub3 | 8 |
| C03 | GO:0044784 | metaphase/anaphase transition of cell cycle | 7/45 | 64/29008 | 7.71E-12 | 1.70E-10 | 8.27E-11 | Ube2c/Ccnb1/Birc5/Cenpe/Plk1/Tacc3/Bub3 | 7 |
| C03 | GO:0051306 | mitotic sister chromatid separation | 7/45 | 65/29008 | 8.63E-12 | 1.84E-10 | 8.96E-11 | Ube2c/Ccnb1/Birc5/Cenpe/Plk1/Tacc3/Bub3 | 7 |
| C03 | GO:0090307 | mitotic spindle assembly | 7/45 | 66/29008 | 9.64E-12 | 1.99E-10 | 9.70E-11 | Prc1/Cdc20/Tpx2/Plk1/Racgap1/Kif23/Nek2 | 7 |
| C03 | GO:0033044 | regulation of chromosome organization | 9/45 | 185/29008 | 1.04E-11 | 2.09E-10 | 1.02E-10 | Ube2c/Ccnb1/Birc5/Cenpe/Top2a/Plk1/Tacc3/Nek2/Bub3 | 9 |
| C03 | GO:1905818 | regulation of chromosome separation | 7/45 | 68/29008 | 1.20E-11 | 2.33E-10 | 1.13E-10 | Ube2c/Ccnb1/Birc5/Cenpe/Plk1/Tacc3/Bub3 | 7 |
| C03 | GO:0033045 | regulation of sister chromatid segregation | 7/45 | 69/29008 | 1.33E-11 | 2.51E-10 | 1.22E-10 | Ube2c/Ccnb1/Birc5/Cenpe/Plk1/Tacc3/Bub3 | 7 |
| C03 | GO:0051988 | regulation of attachment of spindle microtubules to kinetochore | 5/45 | 14/29008 | 1.41E-11 | 2.60E-10 | 1.27E-10 | Ccnb1/Cenpe/Racgap1/Nek2/Knstrn | 5 |
| C03 | GO:0030261 | chromosome condensation | 6/45 | 45/29008 | 7.67E-11 | 1.33E-09 | 6.50E-10 | Ccnb1/Nusap1/Cdk1/Top2a/Smc4/Smc2 | 6 |
| C03 | GO:0031577 | spindle checkpoint signaling | 6/45 | 45/29008 | 7.67E-11 | 1.33E-09 | 6.50E-10 | Ccnb1/Birc5/Cenpe/Plk1/Aurka/Bub3 | 6 |
| C03 | GO:0045787 | positive regulation of cell cycle | 10/45 | 336/29008 | 8.47E-11 | 1.44E-09 | 7.00E-10 | Ube2c/Ccnb1/Nusap1/Birc5/Cenpe/Cdk1/Racgap1/Aurka/Kif23/Kif20b | 10 |
| C03 | GO:0032465 | regulation of cytokinesis | 7/45 | 101/29008 | 2.04E-10 | 3.38E-09 | 1.65E-09 | Prc1/Plk1/Racgap1/Kif20a/Aurka/Kif23/Kif20b | 7 |
| C03 | GO:0030071 | regulation of mitotic metaphase/anaphase transition | 6/45 | 58/29008 | 3.75E-10 | 6.05E-09 | 2.95E-09 | Ube2c/Ccnb1/Birc5/Cenpe/Plk1/Bub3 | 6 |
| C03 | GO:0051302 | regulation of cell division | 8/45 | 186/29008 | 4.32E-10 | 6.80E-09 | 3.32E-09 | Prc1/Plk1/Racgap1/Kif20a/Aurka/Kif23/Aspm/Kif20b | 8 |
| C03 | GO:1902099 | regulation of metaphase/anaphase transition of cell cycle | 6/45 | 61/29008 | 5.13E-10 | 7.89E-09 | 3.84E-09 | Ube2c/Ccnb1/Birc5/Cenpe/Plk1/Bub3 | 6 |
| C03 | GO:0051656 | establishment of organelle localization | 10/45 | 415/29008 | 6.58E-10 | 9.89E-09 | 4.82E-09 | Cenpf/Ccnb1/Cenpa/Nusap1/Birc5/Cenpe/Cdca8/Plk1/Tacc3/Kif2c | 10 |
| C03 | GO:0090306 | meiotic spindle assembly | 4/45 | 10/29008 | 1.05E-09 | 1.55E-08 | 7.54E-09 | Ccnb2/Cenpe/Aurka/Aspm | 4 |
| C03 | GO:0044839 | cell cycle G2/M phase transition | 7/45 | 146/29008 | 2.74E-09 | 3.93E-08 | 1.92E-08 | Cenpf/Ccnb1/Ccnb2/Birc5/Cdk1/Ccna2/Plk1 | 7 |
| C03 | GO:0051303 | establishment of chromosome localization | 6/45 | 82/29008 | 3.16E-09 | 4.44E-08 | 2.16E-08 | Cenpf/Ccnb1/Birc5/Cenpe/Cdca8/Kif2c | 6 |
| C03 | GO:0050000 | chromosome localization | 6/45 | 84/29008 | 3.66E-09 | 5.04E-08 | 2.46E-08 | Cenpf/Ccnb1/Birc5/Cenpe/Cdca8/Kif2c | 6 |
| C03 | GO:0006323 | DNA packaging | 7/45 | 155/29008 | 4.15E-09 | 5.58E-08 | 2.72E-08 | Ccnb1/Nusap1/Cdk1/Top2a/Smc4/Smc2/Hjurp | 7 |
| C03 | GO:2001251 | negative regulation of chromosome organization | 6/45 | 86/29008 | 4.22E-09 | 5.58E-08 | 2.72E-08 | Ccnb1/Birc5/Cenpe/Top2a/Plk1/Bub3 | 6 |
| C04 | GO:0007059 | chromosome segregation | 15/43 | 338/29008 | 8.19E-19 | 5.13E-16 | 3.11E-16 | Top2a/Cenph/Birc5/Aurkb/Smc2/Spc24/Nusap1/Spc25/Esco2/Mki67/Prc1/Smc4/Ube2c/Ndc80/Cenpq | 15 |
| C04 | GO:0000819 | sister chromatid segregation | 10/43 | 188/29008 | 1.64E-13 | 3.85E-11 | 2.34E-11 | Top2a/Birc5/Aurkb/Smc2/Nusap1/Esco2/Prc1/Smc4/Ube2c/Ndc80 | 10 |
| C04 | GO:0098813 | nuclear chromosome segregation | 11/43 | 273/29008 | 1.85E-13 | 3.85E-11 | 2.34E-11 | Top2a/Birc5/Aurkb/Smc2/Nusap1/Esco2/Prc1/Smc4/Ube2c/Ndc80/Cenpq | 11 |
| C04 | GO:0006260 | DNA replication | 10/43 | 253/29008 | 3.17E-12 | 4.97E-10 | 3.02E-10 | Pclaf/Rrm2/Cdk1/Lig1/Rrm1/Esco2/Pcna/Ccna2/Pola1/Rfc4 | 10 |
| C04 | GO:0000070 | mitotic sister chromatid segregation | 8/43 | 159/29008 | 8.41E-11 | 1.05E-08 | 6.39E-09 | Birc5/Aurkb/Smc2/Nusap1/Prc1/Smc4/Ube2c/Ndc80 | 8 |
| C04 | GO:0140014 | mitotic nuclear division | 9/43 | 278/29008 | 2.54E-10 | 2.65E-08 | 1.61E-08 | Birc5/Aurkb/Smc2/Nusap1/Mki67/Prc1/Smc4/Ube2c/Ndc80 | 9 |
| C04 | GO:0000280 | nuclear division | 10/43 | 442/29008 | 7.46E-10 | 6.67E-08 | 4.05E-08 | Top2a/Birc5/Aurkb/Smc2/Nusap1/Mki67/Prc1/Smc4/Ube2c/Ndc80 | 10 |
| C04 | GO:1902850 | microtubule cytoskeleton organization involved in mitosis | 7/43 | 146/29008 | 1.96E-09 | 1.53E-07 | 9.31E-08 | Cenph/Stmn1/Aurkb/Nusap1/Spc25/Prc1/Ndc80 | 7 |
| C04 | GO:0048285 | organelle fission | 10/43 | 495/29008 | 2.21E-09 | 1.54E-07 | 9.35E-08 | Top2a/Birc5/Aurkb/Smc2/Nusap1/Mki67/Prc1/Smc4/Ube2c/Ndc80 | 10 |
| C04 | GO:0071897 | DNA biosynthetic process | 7/43 | 170/29008 | 5.66E-09 | 3.54E-07 | 2.15E-07 | Pclaf/Tk1/Aurkb/Lig1/Pcna/Pola1/Rfc4 | 7 |
| C04 | GO:0030261 | chromosome condensation | 5/43 | 45/29008 | 6.58E-09 | 3.74E-07 | 2.27E-07 | Top2a/Cdk1/Smc2/Nusap1/Smc4 | 5 |
| C04 | GO:0007051 | spindle organization | 7/43 | 183/29008 | 9.43E-09 | 4.92E-07 | 2.99E-07 | Cenph/Stmn1/Tubb5/Aurkb/Spc25/Prc1/Ndc80 | 7 |
| C04 | GO:0034501 | protein localization to kinetochore | 4/43 | 19/29008 | 1.60E-08 | 7.14E-07 | 4.33E-07 | Cdk1/Aurkb/Ndc80/Cenpq | 4 |
| C04 | GO:1903083 | protein localization to condensed chromosome | 4/43 | 19/29008 | 1.60E-08 | 7.14E-07 | 4.33E-07 | Cdk1/Aurkb/Ndc80/Cenpq | 4 |
| C04 | GO:0007346 | regulation of mitotic cell cycle | 9/43 | 462/29008 | 2.13E-08 | 8.84E-07 | 5.37E-07 | Birc5/Cdk1/Aurkb/Nusap1/Mki67/Clspn/Ube2c/Cks1b/Ndc80 | 9 |
| C04 | GO:0007052 | mitotic spindle organization | 6/43 | 119/29008 | 2.26E-08 | 8.84E-07 | 5.37E-07 | Cenph/Stmn1/Aurkb/Spc25/Prc1/Ndc80 | 6 |
| C04 | GO:0051383 | kinetochore organization | 4/43 | 22/29008 | 3.00E-08 | 1.11E-06 | 6.71E-07 | Cenph/Smc2/Smc4/Ndc80 | 4 |
| C04 | GO:0006281 | DNA repair | 9/43 | 495/29008 | 3.84E-08 | 1.34E-06 | 8.12E-07 | Pclaf/Smc2/Lig1/Esco2/Pcna/Clspn/Smc4/Pola1/Rfc4 | 9 |
| C04 | GO:0071459 | protein localization to chromosome | centromeric region | 4/43 | 25/29008 | 5.17E-08 | 1.70E-06 | 1.03E-06 | Cdk1/Aurkb/Ndc80/Cenpq |
| C04 | GO:0006323 | DNA packaging | 6/43 | 155/29008 | 1.09E-07 | 3.42E-06 | 2.08E-06 | Top2a/Asf1b/Cdk1/Smc2/Nusap1/Smc4 | 6 |
| C04 | GO:0044772 | mitotic cell cycle phase transition | 8/43 | 406/29008 | 1.30E-07 | 3.87E-06 | 2.35E-06 | Birc5/Cdk1/Aurkb/Clspn/Ccna2/Ube2c/Cks1b/Ndc80 | 8 |
| C04 | GO:0051983 | regulation of chromosome segregation | 5/43 | 86/29008 | 1.79E-07 | 5.10E-06 | 3.10E-06 | Birc5/Aurkb/Mki67/Ube2c/Ndc80 | 5 |
| C04 | GO:0034502 | protein localization to chromosome | 5/43 | 96/29008 | 3.11E-07 | 8.12E-06 | 4.93E-06 | Cdk1/Aurkb/Esco2/Ndc80/Cenpq | 5 |
| C04 | GO:0051304 | chromosome separation | 5/43 | 96/29008 | 3.11E-07 | 8.12E-06 | 4.93E-06 | Top2a/Birc5/Aurkb/Ube2c/Ndc80 | 5 |
| C04 | GO:0071103 | DNA conformation change | 6/43 | 203/29008 | 5.36E-07 | 1.34E-05 | 8.14E-06 | Top2a/Asf1b/Cdk1/Smc2/Nusap1/Smc4 | 6 |
| C04 | GO:0044770 | cell cycle phase transition | 8/43 | 492/29008 | 5.57E-07 | 1.34E-05 | 8.14E-06 | Birc5/Cdk1/Aurkb/Clspn/Ccna2/Ube2c/Cks1b/Ndc80 | 8 |
| C04 | GO:0006275 | regulation of DNA replication | 5/43 | 110/29008 | 6.14E-07 | 1.37E-05 | 8.33E-06 | Cdk1/Esco2/Pcna/Ccna2/Rfc4 | 5 |
| C04 | GO:0007088 | regulation of mitotic nuclear division | 5/43 | 110/29008 | 6.14E-07 | 1.37E-05 | 8.33E-06 | Birc5/Aurkb/Nusap1/Mki67/Ndc80 | 5 |
| C04 | GO:0006271 | DNA strand elongation involved in DNA replication | 3/43 | 13/29008 | 8.59E-07 | 1.79E-05 | 1.09E-05 | Lig1/Pcna/Pola1 | 3 |
| C04 | GO:0007076 | mitotic chromosome condensation | 3/43 | 13/29008 | 8.59E-07 | 1.79E-05 | 1.09E-05 | Smc2/Nusap1/Smc4 | 3 |
| C04 | GO:0030071 | regulation of mitotic metaphase/anaphase transition | 4/43 | 58/29008 | 1.67E-06 | 3.34E-05 | 2.03E-05 | Birc5/Aurkb/Ube2c/Ndc80 | 4 |
| C04 | GO:0007093 | mitotic cell cycle checkpoint signaling | 5/43 | 136/29008 | 1.75E-06 | 3.34E-05 | 2.03E-05 | Birc5/Cdk1/Aurkb/Clspn/Ndc80 | 5 |
| C04 | GO:0090068 | positive regulation of cell cycle process | 6/43 | 249/29008 | 1.76E-06 | 3.34E-05 | 2.03E-05 | Birc5/Cdk1/Aurkb/Nusap1/Ube2c/Ndc80 | 6 |
| C04 | GO:0007091 | metaphase/anaphase transition of mitotic cell cycle | 4/43 | 61/29008 | 2.05E-06 | 3.67E-05 | 2.23E-05 | Birc5/Aurkb/Ube2c/Ndc80 | 4 |
| C04 | GO:1902099 | regulation of metaphase/anaphase transition of cell cycle | 4/43 | 61/29008 | 2.05E-06 | 3.67E-05 | 2.23E-05 | Birc5/Aurkb/Ube2c/Ndc80 | 4 |
| C04 | GO:0010965 | regulation of mitotic sister chromatid separation | 4/43 | 62/29008 | 2.19E-06 | 3.80E-05 | 2.31E-05 | Birc5/Aurkb/Ube2c/Ndc80 | 4 |
| C04 | GO:0051783 | regulation of nuclear division | 5/43 | 143/29008 | 2.25E-06 | 3.80E-05 | 2.31E-05 | Birc5/Aurkb/Nusap1/Mki67/Ndc80 | 5 |
| C04 | GO:0009263 | deoxyribonucleotide biosynthetic process | 3/43 | 18/29008 | 2.44E-06 | 3.90E-05 | 2.37E-05 | Rrm2/Tyms/Rrm1 | 3 |
| C04 | GO:0044839 | cell cycle G2/M phase transition | 5/43 | 146/29008 | 2.49E-06 | 3.90E-05 | 2.37E-05 | Birc5/Cdk1/Clspn/Ccna2/Ndc80 | 5 |
| C04 | GO:0044784 | metaphase/anaphase transition of cell cycle | 4/43 | 64/29008 | 2.49E-06 | 3.90E-05 | 2.37E-05 | Birc5/Aurkb/Ube2c/Ndc80 | 4 |
| C04 | GO:0051306 | mitotic sister chromatid separation | 4/43 | 65/29008 | 2.65E-06 | 4.05E-05 | 2.46E-05 | Birc5/Aurkb/Ube2c/Ndc80 | 4 |
| C04 | GO:1905818 | regulation of chromosome separation | 4/43 | 68/29008 | 3.18E-06 | 4.74E-05 | 2.88E-05 | Birc5/Aurkb/Ube2c/Ndc80 | 4 |
| C04 | GO:0033045 | regulation of sister chromatid segregation | 4/43 | 69/29008 | 3.37E-06 | 4.91E-05 | 2.98E-05 | Birc5/Aurkb/Ube2c/Ndc80 | 4 |
| C04 | GO:0022616 | DNA strand elongation | 3/43 | 21/29008 | 3.96E-06 | 5.63E-05 | 3.42E-05 | Lig1/Pcna/Pola1 | 3 |
| C04 | GO:1901990 | regulation of mitotic cell cycle phase transition | 6/43 | 293/29008 | 4.49E-06 | 6.25E-05 | 3.79E-05 | Birc5/Cdk1/Aurkb/Clspn/Ube2c/Ndc80 | 6 |
| C04 | GO:1901991 | negative regulation of mitotic cell cycle phase transition | 5/43 | 169/29008 | 5.09E-06 | 6.93E-05 | 4.20E-05 | Birc5/Cdk1/Aurkb/Clspn/Ndc80 | 5 |
| C04 | GO:0000075 | cell cycle checkpoint signaling | 5/43 | 175/29008 | 6.03E-06 | 8.04E-05 | 4.88E-05 | Birc5/Cdk1/Aurkb/Clspn/Ndc80 | 5 |
| C04 | GO:0051303 | establishment of chromosome localization | 4/43 | 82/29008 | 6.73E-06 | 8.78E-05 | 5.33E-05 | Birc5/Aurkb/Ndc80/Cenpq | 4 |
| C04 | GO:0000910 | cytokinesis | 5/43 | 182/29008 | 7.30E-06 | 9.27E-05 | 5.63E-05 | Stmn1/Birc5/Aurkb/Nusap1/Prc1 | 5 |
| C04 | GO:0050000 | chromosome localization | 4/43 | 84/29008 | 7.41E-06 | 9.27E-05 | 5.63E-05 | Birc5/Aurkb/Ndc80/Cenpq | 4 |
| C05 | GO:0007059 | chromosome segregation | 17/45 | 338/29008 | 7.38E-22 | 6.82E-19 | 4.40E-19 | Top2a/Ube2c/Prc1/Nusap1/Smc2/Birc5/Smc4/Aurkb/Cdca8/Spc24/Spc25/Esco2/Incenp/Cenph/Ccnb1/Mki67/Racgap1 | 17 |
| C05 | GO:0000819 | sister chromatid segregation | 13/45 | 188/29008 | 1.42E-18 | 6.58E-16 | 4.24E-16 | Top2a/Ube2c/Prc1/Nusap1/Smc2/Birc5/Smc4/Aurkb/Cdca8/Esco2/Incenp/Ccnb1/Racgap1 | 13 |
| C05 | GO:0030261 | chromosome condensation | 9/45 | 45/29008 | 1.88E-17 | 5.80E-15 | 3.74E-15 | Top2a/Cdk1/Nusap1/Smc2/Smc4/Incenp/H3f3b/Ccnb1/H1f0 | 9 |
| C05 | GO:0000280 | nuclear division | 15/45 | 442/29008 | 9.94E-17 | 2.30E-14 | 1.48E-14 | Top2a/Ube2c/Prc1/Nusap1/Smc2/Birc5/Smc4/Aurkb/Cdca8/Kif11/Incenp/Ccnb1/Mki67/Racgap1/Cks2 | 15 |
| C05 | GO:0098813 | nuclear chromosome segregation | 13/45 | 273/29008 | 1.90E-16 | 3.52E-14 | 2.27E-14 | Top2a/Ube2c/Prc1/Nusap1/Smc2/Birc5/Smc4/Aurkb/Cdca8/Esco2/Incenp/Ccnb1/Racgap1 | 13 |
| C05 | GO:0140014 | mitotic nuclear division | 13/45 | 278/29008 | 2.41E-16 | 3.71E-14 | 2.39E-14 | Ube2c/Prc1/Nusap1/Smc2/Birc5/Smc4/Aurkb/Cdca8/Kif11/Incenp/Ccnb1/Mki67/Racgap1 | 13 |
| C05 | GO:0048285 | organelle fission | 15/45 | 495/29008 | 5.29E-16 | 6.99E-14 | 4.51E-14 | Top2a/Ube2c/Prc1/Nusap1/Smc2/Birc5/Smc4/Aurkb/Cdca8/Kif11/Incenp/Ccnb1/Mki67/Racgap1/Cks2 | 15 |
| C05 | GO:0000070 | mitotic sister chromatid segregation | 11/45 | 159/29008 | 8.17E-16 | 9.43E-14 | 6.08E-14 | Ube2c/Prc1/Nusap1/Smc2/Birc5/Smc4/Aurkb/Cdca8/Incenp/Ccnb1/Racgap1 | 11 |
| C05 | GO:0007051 | spindle organization | 11/45 | 183/29008 | 3.92E-15 | 4.02E-13 | 2.59E-13 | Prc1/Aurkb/Spc25/Kif11/Incenp/Cenph/Ccnb1/Stmn1/Tubb5/Racgap1/Ezr | 11 |
| C05 | GO:0006323 | DNA packaging | 10/45 | 155/29008 | 3.84E-14 | 3.55E-12 | 2.29E-12 | Top2a/Cdk1/Nusap1/Smc2/Smc4/Asf1b/Incenp/H3f3b/Ccnb1/H1f0 | 10 |
| C05 | GO:0071103 | DNA conformation change | 10/45 | 203/29008 | 5.81E-13 | 4.88E-11 | 3.15E-11 | Top2a/Cdk1/Nusap1/Smc2/Smc4/Asf1b/Incenp/H3f3b/Ccnb1/H1f0 | 10 |
| C05 | GO:1902850 | microtubule cytoskeleton organization involved in mitosis | 9/45 | 146/29008 | 1.23E-12 | 9.45E-11 | 6.09E-11 | Prc1/Nusap1/Aurkb/Spc25/Kif11/Cenph/Ccnb1/Stmn1/Racgap1 | 9 |
| C05 | GO:0007052 | mitotic spindle organization | 8/45 | 119/29008 | 1.20E-11 | 8.53E-10 | 5.50E-10 | Prc1/Aurkb/Spc25/Kif11/Cenph/Ccnb1/Stmn1/Racgap1 | 8 |
| C05 | GO:0061640 | cytoskeleton-dependent cytokinesis | 7/45 | 93/29008 | 1.14E-10 | 7.50E-09 | 4.84E-09 | Nusap1/Birc5/Anln/Aurkb/Incenp/Stmn1/Racgap1 | 7 |
| C05 | GO:0000910 | cytokinesis | 8/45 | 182/29008 | 3.64E-10 | 2.24E-08 | 1.44E-08 | Prc1/Nusap1/Birc5/Anln/Aurkb/Incenp/Stmn1/Racgap1 | 8 |
| C05 | GO:0000281 | mitotic cytokinesis | 6/45 | 67/29008 | 9.16E-10 | 5.29E-08 | 3.41E-08 | Nusap1/Birc5/Anln/Incenp/Stmn1/Racgap1 | 6 |
| C05 | GO:0007346 | regulation of mitotic cell cycle | 10/45 | 462/29008 | 1.85E-09 | 1.00E-07 | 6.47E-08 | Ube2c/Cdk1/Nusap1/Birc5/Cks1b/Aurkb/Ccnb1/Mki67/Cks2/Ezh2 | 10 |
| C05 | GO:0090068 | positive regulation of cell cycle process | 8/45 | 249/29008 | 4.32E-09 | 2.22E-07 | 1.43E-07 | Ube2c/Cdk1/Nusap1/Birc5/Aurkb/Ccnb1/Racgap1/Ezh2 | 8 |
| C05 | GO:0044772 | mitotic cell cycle phase transition | 9/45 | 406/29008 | 1.07E-08 | 5.21E-07 | 3.36E-07 | Ube2c/Cdk1/Birc5/Cks1b/Ccna2/Aurkb/Ccnb1/Cks2/Ezh2 | 9 |
| C05 | GO:0051225 | spindle assembly | 6/45 | 115/29008 | 2.44E-08 | 1.13E-06 | 7.28E-07 | Prc1/Aurkb/Kif11/Incenp/Tubb5/Racgap1 | 6 |
| C05 | GO:0045787 | positive regulation of cell cycle | 8/45 | 336/29008 | 4.43E-08 | 1.95E-06 | 1.26E-06 | Ube2c/Cdk1/Nusap1/Birc5/Aurkb/Ccnb1/Racgap1/Ezh2 | 8 |
| C05 | GO:0044770 | cell cycle phase transition | 9/45 | 492/29008 | 5.57E-08 | 2.34E-06 | 1.51E-06 | Ube2c/Cdk1/Birc5/Cks1b/Ccna2/Aurkb/Ccnb1/Cks2/Ezh2 | 9 |
| C05 | GO:0140694 | non-membrane-bounded organelle assembly | 8/45 | 373/29008 | 9.87E-08 | 3.96E-06 | 2.56E-06 | Prc1/Aurkb/Kif11/Incenp/Cenph/Tubb5/Racgap1/Ezr | 8 |
| C05 | GO:0051303 | establishment of chromosome localization | 5/45 | 82/29008 | 1.78E-07 | 6.87E-06 | 4.43E-06 | Birc5/Aurkb/Cdca8/Incenp/Ccnb1 | 5 |
| C05 | GO:0050000 | chromosome localization | 5/45 | 84/29008 | 2.01E-07 | 7.44E-06 | 4.80E-06 | Birc5/Aurkb/Cdca8/Incenp/Ccnb1 | 5 |
| C05 | GO:0051983 | regulation of chromosome segregation | 5/45 | 86/29008 | 2.27E-07 | 7.75E-06 | 5.00E-06 | Ube2c/Birc5/Aurkb/Ccnb1/Mki67 | 5 |
| C05 | GO:2001251 | negative regulation of chromosome organization | 5/45 | 86/29008 | 2.27E-07 | 7.75E-06 | 5.00E-06 | Top2a/Birc5/Aurkb/H3f3b/Ccnb1 | 5 |
| C05 | GO:0016572 | histone phosphorylation | 4/45 | 37/29008 | 3.21E-07 | 1.06E-05 | 6.84E-06 | Ccna2/Aurkb/Incenp/Ccnb1 | 4 |
| C05 | GO:0051304 | chromosome separation | 5/45 | 96/29008 | 3.93E-07 | 1.25E-05 | 8.08E-06 | Top2a/Ube2c/Birc5/Aurkb/Ccnb1 | 5 |
| C05 | GO:0033044 | regulation of chromosome organization | 6/45 | 185/29008 | 4.11E-07 | 1.27E-05 | 8.16E-06 | Top2a/Ube2c/Birc5/Aurkb/H3f3b/Ccnb1 | 6 |
| C05 | GO:0007088 | regulation of mitotic nuclear division | 5/45 | 110/29008 | 7.75E-07 | 2.31E-05 | 1.49E-05 | Nusap1/Birc5/Aurkb/Ccnb1/Mki67 | 5 |
| C05 | GO:1901989 | positive regulation of cell cycle phase transition | 5/45 | 111/29008 | 8.10E-07 | 2.34E-05 | 1.51E-05 | Ube2c/Cdk1/Birc5/Ccnb1/Ezh2 | 5 |
| C05 | GO:0007076 | mitotic chromosome condensation | 3/45 | 13/29008 | 9.87E-07 | 2.76E-05 | 1.78E-05 | Nusap1/Smc2/Smc4 | 3 |
| C05 | GO:0051988 | regulation of attachment of spindle microtubules to kinetochore | 3/45 | 14/29008 | 1.25E-06 | 3.41E-05 | 2.20E-05 | Aurkb/Ccnb1/Racgap1 | 3 |
| C05 | GO:0030071 | regulation of mitotic metaphase/anaphase transition | 4/45 | 58/29008 | 2.02E-06 | 5.32E-05 | 3.43E-05 | Ube2c/Birc5/Aurkb/Ccnb1 | 4 |
| C05 | GO:0007091 | metaphase/anaphase transition of mitotic cell cycle | 4/45 | 61/29008 | 2.47E-06 | 6.17E-05 | 3.98E-05 | Ube2c/Birc5/Aurkb/Ccnb1 | 4 |
| C05 | GO:1902099 | regulation of metaphase/anaphase transition of cell cycle | 4/45 | 61/29008 | 2.47E-06 | 6.17E-05 | 3.98E-05 | Ube2c/Birc5/Aurkb/Ccnb1 | 4 |
| C05 | GO:0010965 | regulation of mitotic sister chromatid separation | 4/45 | 62/29008 | 2.64E-06 | 6.42E-05 | 4.14E-05 | Ube2c/Birc5/Aurkb/Ccnb1 | 4 |
| C05 | GO:0051783 | regulation of nuclear division | 5/45 | 143/29008 | 2.83E-06 | 6.70E-05 | 4.32E-05 | Nusap1/Birc5/Aurkb/Ccnb1/Mki67 | 5 |
| C05 | GO:0044784 | metaphase/anaphase transition of cell cycle | 4/45 | 64/29008 | 3.00E-06 | 6.76E-05 | 4.36E-05 | Ube2c/Birc5/Aurkb/Ccnb1 | 4 |
| C05 | GO:0051310 | metaphase plate congression | 4/45 | 64/29008 | 3.00E-06 | 6.76E-05 | 4.36E-05 | Aurkb/Cdca8/Incenp/Ccnb1 | 4 |
| C05 | GO:0051306 | mitotic sister chromatid separation | 4/45 | 65/29008 | 3.19E-06 | 7.02E-05 | 4.53E-05 | Ube2c/Birc5/Aurkb/Ccnb1 | 4 |
| C05 | GO:0051656 | establishment of organelle localization | 7/45 | 415/29008 | 3.31E-06 | 7.12E-05 | 4.59E-05 | Nusap1/Birc5/Aurkb/Cdca8/Incenp/Ccnb1/Ezr | 7 |
| C05 | GO:0090307 | mitotic spindle assembly | 4/45 | 66/29008 | 3.39E-06 | 7.13E-05 | 4.60E-05 | Prc1/Aurkb/Kif11/Racgap1 | 4 |
| C05 | GO:1905818 | regulation of chromosome separation | 4/45 | 68/29008 | 3.83E-06 | 7.86E-05 | 5.07E-05 | Ube2c/Birc5/Aurkb/Ccnb1 | 4 |
| C05 | GO:0033045 | regulation of sister chromatid segregation | 4/45 | 69/29008 | 4.06E-06 | 8.15E-05 | 5.26E-05 | Ube2c/Birc5/Aurkb/Ccnb1 | 4 |
| C05 | GO:0051383 | kinetochore organization | 3/45 | 22/29008 | 5.26E-06 | 0.000103456 | 6.67E-05 | Smc2/Smc4/Cenph | 3 |
| C05 | GO:1901990 | regulation of mitotic cell cycle phase transition | 6/45 | 293/29008 | 5.90E-06 | 0.000113609 | 7.33E-05 | Ube2c/Cdk1/Birc5/Aurkb/Ccnb1/Ezh2 | 6 |
| C05 | GO:1901991 | negative regulation of mitotic cell cycle phase transition | 5/45 | 169/29008 | 6.40E-06 | 0.000120683 | 7.78E-05 | Cdk1/Birc5/Aurkb/Ccnb1/Ezh2 | 5 |
| C05 | GO:0006325 | chromatin organization | 7/45 | 477/29008 | 8.23E-06 | 0.000152011 | 9.80E-05 | Asf1b/H3f3b/Mki67/Atad2/Dek/Ezh2/H1f0 | 7 |
| C06 | GO:0000727 | double-strand break repair via break-induced replication | 6/49 | 12/29008 | 1.55E-14 | 2.29E-11 | 1.45E-11 | Mcm6/Mcm3/Mcm2/Mcm5/Mcm4/Mcm7 | 6 |
| C06 | GO:0006270 | DNA replication initiation | 6/49 | 31/29008 | 1.21E-11 | 8.92E-09 | 5.64E-09 | Mcm6/Mcm3/Mcm2/Mcm5/Mcm4/Mcm7 | 6 |
| C06 | GO:0032508 | DNA duplex unwinding | 6/49 | 37/29008 | 3.78E-11 | 1.86E-08 | 1.18E-08 | Mcm6/Mcm3/Mcm2/Mcm5/Mcm4/Mcm7 | 6 |
| C06 | GO:0032392 | DNA geometric change | 6/49 | 44/29008 | 1.14E-10 | 4.21E-08 | 2.66E-08 | Mcm6/Mcm3/Mcm2/Mcm5/Mcm4/Mcm7 | 6 |
| C06 | GO:0071103 | DNA conformation change | 8/49 | 203/29008 | 1.77E-09 | 5.23E-07 | 3.31E-07 | Hells/Mcm6/Mcm3/Mcm2/Mcm5/Mcm4/Mcm7/Hmga2 | 8 |
| C06 | GO:0006268 | DNA unwinding involved in DNA replication | 4/49 | 14/29008 | 7.10E-09 | 1.50E-06 | 9.49E-07 | Mcm6/Mcm2/Mcm4/Mcm7 | 4 |
| C06 | GO:1902969 | mitotic DNA replication | 4/49 | 14/29008 | 7.10E-09 | 1.50E-06 | 9.49E-07 | Mcm6/Mcm3/Mcm2/Mcm4 | 4 |
| C06 | GO:0000724 | double-strand break repair via homologous recombination | 6/49 | 135/29008 | 1.08E-07 | 1.93E-05 | 1.22E-05 | Mcm6/Mcm3/Mcm2/Mcm5/Mcm4/Mcm7 | 6 |
| C06 | GO:0000725 | recombinational repair | 6/49 | 137/29008 | 1.18E-07 | 1.93E-05 | 1.22E-05 | Mcm6/Mcm3/Mcm2/Mcm5/Mcm4/Mcm7 | 6 |
| C06 | GO:0006261 | DNA-dependent DNA replication | 6/49 | 142/29008 | 1.46E-07 | 2.15E-05 | 1.36E-05 | Mcm6/Mcm3/Mcm2/Mcm5/Mcm4/Mcm7 | 6 |
| C06 | GO:0006302 | double-strand break repair | 7/49 | 244/29008 | 1.74E-07 | 2.34E-05 | 1.48E-05 | Mcm6/Mcm3/Mcm2/Mcm5/Mcm4/Mcm7/Hmga2 | 7 |
| C06 | GO:0033260 | nuclear DNA replication | 4/49 | 33/29008 | 2.84E-07 | 3.50E-05 | 2.21E-05 | Mcm6/Mcm3/Mcm2/Mcm4 | 4 |
| C06 | GO:0044786 | cell cycle DNA replication | 4/49 | 38/29008 | 5.08E-07 | 5.79E-05 | 3.66E-05 | Mcm6/Mcm3/Mcm2/Mcm4 | 4 |
| C06 | GO:0006310 | DNA recombination | 7/49 | 301/29008 | 7.14E-07 | 7.55E-05 | 4.78E-05 | Mcm6/Mcm3/Mcm2/Mcm5/Hspd1/Mcm4/Mcm7 | 7 |
| C06 | GO:0050673 | epithelial cell proliferation | 8/49 | 471/29008 | 1.15E-06 | 0.000113006 | 7.15E-05 | Ngfr/Klk8/Ctsl/Ccnd1/Igfbp4/Hmga2/Htra1/Ecm1 | 8 |
| C06 | GO:0006271 | DNA strand elongation involved in DNA replication | 3/49 | 13/29008 | 1.28E-06 | 0.000118404 | 7.49E-05 | Mcm3/Mcm4/Mcm7 | 3 |
| C06 | GO:0006281 | DNA repair | 8/49 | 495/29008 | 1.66E-06 | 0.000144373 | 9.13E-05 | Hmga1/Mcm6/Mcm3/Mcm2/Mcm5/Mcm4/Mcm7/Hmga2 | 8 |
| C06 | GO:0006260 | DNA replication | 6/49 | 253/29008 | 4.24E-06 | 0.000348384 | 0.00022028 | Mcm6/Mcm3/Mcm2/Mcm5/Mcm4/Mcm7 | 6 |
| C06 | GO:0022616 | DNA strand elongation | 3/49 | 21/29008 | 5.90E-06 | 0.00045929 | 0.000290404 | Mcm3/Mcm4/Mcm7 | 3 |
| C06 | GO:0048608 | reproductive structure development | 7/49 | 480/29008 | 1.53E-05 | 0.001133596 | 0.000716762 | Ctsl/Ccnd1/Inhba/Fkbp4/Fosl1/Hmga2/Htra1 | 7 |
| C06 | GO:0061458 | reproductive system development | 7/49 | 484/29008 | 1.62E-05 | 0.001138791 | 0.000720047 | Ctsl/Ccnd1/Inhba/Fkbp4/Fosl1/Hmga2/Htra1 | 7 |
| C06 | GO:0008637 | apoptotic mitochondrial changes | 4/49 | 117/29008 | 4.63E-05 | 0.003113987 | 0.001968944 | Ggct/Slc25a5/Hspd1/Siva1 | 4 |
| C06 | GO:2001233 | regulation of apoptotic signaling pathway | 6/49 | 402/29008 | 5.77E-05 | 0.003606001 | 0.002280039 | Ngfr/Hells/Inhba/Tnfrsf12a/Slc25a5/Lgals3 | 6 |
| C06 | GO:0050678 | regulation of epithelial cell proliferation | 6/49 | 403/29008 | 5.85E-05 | 0.003606001 | 0.002280039 | Ngfr/Ctsl/Ccnd1/Hmga2/Htra1/Ecm1 | 6 |
| C06 | GO:0030218 | erythrocyte differentiation | 4/49 | 143/29008 | 0.000100977 | 0.005977826 | 0.003779721 | Casp3/Inhba/Slc25a5/Hspa9 | 4 |
| C06 | GO:0030049 | muscle filament sliding | 2/49 | 10/29008 | 0.000124703 | 0.007098456 | 0.004488284 | Tpm1/Tnnt2 | 2 |
| C06 | GO:0034101 | erythrocyte homeostasis | 4/49 | 155/29008 | 0.000137781 | 0.007552414 | 0.004775317 | Casp3/Inhba/Slc25a5/Hspa9 | 4 |
| C06 | GO:0045765 | regulation of angiogenesis | 5/49 | 302/29008 | 0.000155056 | 0.008195798 | 0.005182123 | Ngfr/Tnfrsf12a/Lgals3/Hmga2/Ecm1 | 5 |
| C06 | GO:1901342 | regulation of vasculature development | 5/49 | 306/29008 | 0.000164838 | 0.008412433 | 0.005319099 | Ngfr/Tnfrsf12a/Lgals3/Hmga2/Ecm1 | 5 |
| C06 | GO:1990830 | cellular response to leukemia inhibitory factor | 5/49 | 311/29008 | 0.000177723 | 0.008440745 | 0.005337 | Hells/Eps8/Ncl/Slc25a5/Srm | 5 |
| C06 | GO:1990823 | response to leukemia inhibitory factor | 5/49 | 312/29008 | 0.00018039 | 0.008440745 | 0.005337 | Hells/Eps8/Ncl/Slc25a5/Srm | 5 |
| C06 | GO:0033275 | actin-myosin filament sliding | 2/49 | 12/29008 | 0.000182503 | 0.008440745 | 0.005337 | Tpm1/Tnnt2 | 2 |
| C06 | GO:0046661 | male sex differentiation | 4/49 | 172/29008 | 0.000205365 | 0.009110548 | 0.00576051 | Ccnd1/Inhba/Fkbp4/Hmga2 | 4 |
| C06 | GO:0071863 | regulation of cell proliferation in bone marrow | 2/49 | 13/29008 | 0.000215452 | 0.009110548 | 0.00576051 | Lgals3/Hmga2 | 2 |
| C06 | GO:2000774 | positive regulation of cellular senescence | 2/49 | 13/29008 | 0.000215452 | 0.009110548 | 0.00576051 | Hmga1/Hmga2 | 2 |
| C06 | GO:0055008 | cardiac muscle tissue morphogenesis | 3/49 | 72/29008 | 0.000248865 | 0.009528529 | 0.006024796 | Tpm1/Tnnt2/Ankrd1 | 3 |
| C06 | GO:0071674 | mononuclear cell migration | 4/49 | 181/29008 | 0.000249492 | 0.009528529 | 0.006024796 | Eps8/Lgals3/Pla2g7/Ecm1 | 4 |
| C06 | GO:0051764 | actin crosslink formation | 2/49 | 14/29008 | 0.00025109 | 0.009528529 | 0.006024796 | Eps8/Tnnt2 | 2 |
| C06 | GO:0071838 | cell proliferation in bone marrow | 2/49 | 14/29008 | 0.00025109 | 0.009528529 | 0.006024796 | Lgals3/Hmga2 | 2 |
| C06 | GO:0090343 | positive regulation of cell aging | 2/49 | 15/29008 | 0.000289406 | 0.010708031 | 0.006770583 | Hmga1/Hmga2 | 2 |
| C06 | GO:0002262 | myeloid cell homeostasis | 4/49 | 196/29008 | 0.000337621 | 0.012187284 | 0.0077059 | Casp3/Inhba/Slc25a5/Hspa9 | 4 |
| C06 | GO:0048872 | homeostasis of number of cells | 5/49 | 363/29008 | 0.00036225 | 0.012625859 | 0.007983207 | Casp3/Inhba/Slc25a5/Hspa9/Siva1 | 5 |
| C06 | GO:0010639 | negative regulation of organelle organization | 5/49 | 364/29008 | 0.000366832 | 0.012625859 | 0.007983207 | Eps8/Mcm2/Slc25a5/Fkbp4/Tpm1 | 5 |
| C06 | GO:0032272 | negative regulation of protein polymerization | 3/49 | 84/29008 | 0.000391983 | 0.013184896 | 0.008336681 | Eps8/Fkbp4/Tpm1 | 3 |
| C06 | GO:0060415 | muscle tissue morphogenesis | 3/49 | 86/29008 | 0.000420014 | 0.013524085 | 0.008551146 | Tpm1/Tnnt2/Ankrd1 | 3 |
| C06 | GO:0042026 | protein refolding | 2/49 | 18/29008 | 0.000420343 | 0.013524085 | 0.008551146 | Hspd1/Hspa9 | 2 |
| C06 | GO:0034620 | cellular response to unfolded protein | 3/49 | 88/29008 | 0.000449302 | 0.014148234 | 0.008945789 | Ccnd1/Hspd1/Hspa9 | 3 |
| C06 | GO:0050679 | positive regulation of epithelial cell proliferation | 4/49 | 215/29008 | 0.000478796 | 0.014762874 | 0.00933442 | Ccnd1/Hmga2/Htra1/Ecm1 | 4 |
| C06 | GO:1901028 | regulation of mitochondrial outer membrane permeabilization involved in apoptotic signaling pathway | 2/49 | 20/29008 | 0.00052087 | 0.015732391 | 0.009947436 | Slc25a5/Siva1 | 2 |
| C06 | GO:0048644 | muscle organ morphogenesis | 3/49 | 94/29008 | 0.000544941 | 0.016130265 | 0.010199008 | Tpm1/Tnnt2/Ankrd1 | 3 |
| F01 | GO:0018149 | peptide cross-linking | 3月16日 | 41/29008 | 1.45E-06 | 0.00105625 | 0.000483476 | Col3a1/Dcn/Bgn | 3 |
| F01 | GO:0048844 | artery morphogenesis | 3月16日 | 80/29008 | 1.10E-05 | 0.003110938 | 0.001423966 | Col3a1/Stra6/Sox4 | 3 |
| F01 | GO:0097501 | stress response to metal ion | 2月16日 | 10/29008 | 1.28E-05 | 0.003110938 | 0.001423966 | Mt1/Mt2 | 2 |
| F01 | GO:0071294 | cellular response to zinc ion | 2月16日 | 14/29008 | 2.59E-05 | 0.004410127 | 0.002018642 | Mt1/Mt2 | 2 |
| F01 | GO:0060840 | artery development | 3月16日 | 112/29008 | 3.02E-05 | 0.004410127 | 0.002018642 | Col3a1/Stra6/Sox4 | 3 |
| F01 | GO:0071280 | cellular response to copper ion | 2月16日 | 18/29008 | 4.34E-05 | 0.004649419 | 0.002128173 | Mt1/Mt2 | 2 |
| F01 | GO:0001655 | urogenital system development | 4月16日 | 385/29008 | 4.90E-05 | 0.004649419 | 0.002128173 | Mmp2/Stra6/Sox4/Vcan | 4 |
| F01 | GO:0090141 | positive regulation of mitochondrial fission | 2月16日 | 20/29008 | 5.39E-05 | 0.004649419 | 0.002128173 | Dcn/Bnip3 | 2 |
| F01 | GO:0055123 | digestive system development | 3月16日 | 139/29008 | 5.76E-05 | 0.004649419 | 0.002128173 | Col3a1/Stra6/Wls | 3 |
| F01 | GO:0007263 | nitric oxide mediated signal transduction | 2月16日 | 25/29008 | 8.49E-05 | 0.004649419 | 0.002128173 | Mt1/Mt2 | 2 |
| F01 | GO:0071276 | cellular response to cadmium ion | 2月16日 | 26/29008 | 9.20E-05 | 0.004649419 | 0.002128173 | Mt1/Mt2 | 2 |
| F01 | GO:0030204 | chondroitin sulfate metabolic process | 2月16日 | 27/29008 | 9.93E-05 | 0.004649419 | 0.002128173 | Dcn/Bgn | 2 |
| F01 | GO:0071248 | cellular response to metal ion | 3月16日 | 167/29008 | 9.93E-05 | 0.004649419 | 0.002128173 | Mt1/Mt2/Bnip3 | 3 |
| F01 | GO:0006882 | cellular zinc ion homeostasis | 2月16日 | 28/29008 | 0.000106918 | 0.004649419 | 0.002128173 | Mt1/Mt2 | 2 |
| F01 | GO:0046688 | response to copper ion | 2月16日 | 28/29008 | 0.000106918 | 0.004649419 | 0.002128173 | Mt1/Mt2 | 2 |
| F01 | GO:0048745 | smooth muscle tissue development | 2月16日 | 29/29008 | 0.0001148 | 0.004649419 | 0.002128173 | Col3a1/Stra6 | 2 |
| F01 | GO:0055069 | zinc ion homeostasis | 2月16日 | 29/29008 | 0.0001148 | 0.004649419 | 0.002128173 | Mt1/Mt2 | 2 |
| F01 | GO:1903319 | positive regulation of protein maturation | 2月16日 | 29/29008 | 0.0001148 | 0.004649419 | 0.002128173 | Sox4/Mmp14 | 2 |
| F01 | GO:0050654 | chondroitin sulfate proteoglycan metabolic process | 2月16日 | 34/29008 | 0.000158373 | 0.005889591 | 0.002695835 | Dcn/Bgn | 2 |
| F01 | GO:0090140 | regulation of mitochondrial fission | 2月16日 | 35/29008 | 0.000167918 | 0.005889591 | 0.002695835 | Dcn/Bnip3 | 2 |
| F01 | GO:0048771 | tissue remodeling | 3月16日 | 202/29008 | 0.000174255 | 0.005889591 | 0.002695835 | Mmp2/Gpnmb/Mmp14 | 3 |
| F01 | GO:0010043 | response to zinc ion | 2月16日 | 36/29008 | 0.000177738 | 0.005889591 | 0.002695835 | Mt1/Mt2 | 2 |
| F01 | GO:0035909 | aorta morphogenesis | 2月16日 | 37/29008 | 0.000187834 | 0.005953522 | 0.002725098 | Col3a1/Sox4 | 2 |
| F01 | GO:0046686 | response to cadmium ion | 2月16日 | 38/29008 | 0.000198205 | 0.006020491 | 0.002755752 | Mt1/Mt2 | 2 |
| F01 | GO:0001666 | response to hypoxia | 3月16日 | 214/29008 | 0.000206531 | 0.00602243 | 0.002756639 | Mmp2/Bnip3/Aldh3a1 | 3 |
| F01 | GO:0060325 | face morphogenesis | 2月16日 | 40/29008 | 0.000219774 | 0.006162114 | 0.002820576 | Mmp2/Stra6 | 2 |
| F01 | GO:0030324 | lung development | 3月16日 | 233/29008 | 0.000265176 | 0.006515946 | 0.002982535 | Col3a1/Stra6/Mmp14 | 3 |
| F01 | GO:0030323 | respiratory tube development | 3月16日 | 236/29008 | 0.000275318 | 0.006515946 | 0.002982535 | Col3a1/Stra6/Mmp14 | 3 |
| F01 | GO:0071241 | cellular response to inorganic substance | 3月16日 | 237/29008 | 0.000278754 | 0.006515946 | 0.002982535 | Mt1/Mt2/Bnip3 | 3 |
| F01 | GO:0060323 | head morphogenesis | 2月16日 | 46/29008 | 0.00029106 | 0.006515946 | 0.002982535 | Mmp2/Stra6 | 2 |
| F01 | GO:0036293 | response to decreased oxygen levels | 3月16日 | 241/29008 | 0.000292775 | 0.006515946 | 0.002982535 | Mmp2/Bnip3/Aldh3a1 | 3 |
| F01 | GO:0060348 | bone development | 3月16日 | 243/29008 | 0.000299954 | 0.006515946 | 0.002982535 | Col3a1/Bgn/Mmp14 | 3 |
| F01 | GO:0000266 | mitochondrial fission | 2月16日 | 47/29008 | 0.000303899 | 0.006515946 | 0.002982535 | Dcn/Bnip3 | 2 |
| F01 | GO:0030574 | collagen catabolic process | 2月16日 | 47/29008 | 0.000303899 | 0.006515946 | 0.002982535 | Mmp2/Mmp14 | 2 |
| F01 | GO:0003179 | heart valve morphogenesis | 2月16日 | 48/29008 | 0.00031701 | 0.00660286 | 0.003022318 | Stra6/Sox4 | 2 |
| F01 | GO:0048705 | skeletal system morphogenesis | 3月16日 | 261/29008 | 0.000369742 | 0.007487269 | 0.003427138 | Col3a1/Mmp2/Mmp14 | 3 |
| F01 | GO:0060541 | respiratory system development | 3月16日 | 266/29008 | 0.000390828 | 0.007700361 | 0.003524676 | Col3a1/Stra6/Mmp14 | 3 |
| F01 | GO:0098754 | detoxification | 2月16日 | 58/29008 | 0.000463063 | 0.00888349 | 0.004066228 | Mt1/Mt2 | 2 |
| F01 | GO:0003170 | heart valve development | 2月16日 | 59/29008 | 0.000479156 | 0.008956537 | 0.004099664 | Stra6/Sox4 | 2 |
| F01 | GO:0060324 | face development | 2月16日 | 61/29008 | 0.000512152 | 0.009048735 | 0.004141865 | Mmp2/Stra6 | 2 |
| F01 | GO:0010038 | response to metal ion | 3月16日 | 292/29008 | 0.000513011 | 0.009048735 | 0.004141865 | Mt1/Mt2/Bnip3 | 3 |
| F01 | GO:0010171 | body morphogenesis | 2月16日 | 62/29008 | 0.000529054 | 0.009048735 | 0.004141865 | Mmp2/Stra6 | 2 |
| F01 | GO:0070482 | response to oxygen levels | 3月16日 | 296/29008 | 0.000533739 | 0.009048735 | 0.004141865 | Mmp2/Bnip3/Aldh3a1 | 3 |
| F01 | GO:0006081 | cellular aldehyde metabolic process | 2月16日 | 64/29008 | 0.000563663 | 0.009092462 | 0.00416188 | Aldoa/Aldh3a1 | 2 |
| F01 | GO:0016239 | positive regulation of macroautophagy | 2月16日 | 66/29008 | 0.000599345 | 0.009092462 | 0.00416188 | Dcn/Bnip3 | 2 |
| F01 | GO:0060350 | endochondral bone morphogenesis | 2月16日 | 66/29008 | 0.000599345 | 0.009092462 | 0.00416188 | Col3a1/Mmp14 | 2 |
| F01 | GO:0007162 | negative regulation of cell adhesion | 3月16日 | 310/29008 | 0.000610508 | 0.009092462 | 0.00416188 | Mmp2/Gpnmb/Mmp14 | 3 |
| F01 | GO:0030198 | extracellular matrix organization | 3月16日 | 311/29008 | 0.000616247 | 0.009092462 | 0.00416188 | Col3a1/Mmp2/Mmp14 | 3 |
| F01 | GO:0043062 | extracellular structure organization | 3月16日 | 312/29008 | 0.000622021 | 0.009092462 | 0.00416188 | Col3a1/Mmp2/Mmp14 | 3 |
| F01 | GO:0045229 | external encapsulating structure organization | 3月16日 | 313/29008 | 0.000627829 | 0.009092462 | 0.00416188 | Col3a1/Mmp2/Mmp14 | 3 |
| F02 | GO:0030198 | extracellular matrix organization | 10/48 | 311/29008 | 7.92E-11 | 3.19E-08 | 2.02E-08 | Col3a1/Ramp2/Mmp2/Fbln2/Col1a2/Adamts1/Mmp14/Col5a2/Col5a1/Col1a1 | 10 |
| F02 | GO:0043062 | extracellular structure organization | 10/48 | 312/29008 | 8.18E-11 | 3.19E-08 | 2.02E-08 | Col3a1/Ramp2/Mmp2/Fbln2/Col1a2/Adamts1/Mmp14/Col5a2/Col5a1/Col1a1 | 10 |
| F02 | GO:0045229 | external encapsulating structure organization | 10/48 | 313/29008 | 8.44E-11 | 3.19E-08 | 2.02E-08 | Col3a1/Ramp2/Mmp2/Fbln2/Col1a2/Adamts1/Mmp14/Col5a2/Col5a1/Col1a1 | 10 |
| F02 | GO:0007179 | transforming growth factor beta receptor signaling pathway | 7/48 | 186/29008 | 2.35E-08 | 6.65E-06 | 4.22E-06 | Col3a1/Aspn/Fbn1/Lrp1/Itgb5/Col1a2/Htra3 | 7 |
| F02 | GO:0030199 | collagen fibril organization | 5/48 | 59/29008 | 4.69E-08 | 1.06E-05 | 6.75E-06 | Col3a1/Col1a2/Col5a2/Col5a1/Col1a1 | 5 |
| F02 | GO:0071560 | cellular response to transforming growth factor beta stimulus | 7/48 | 226/29008 | 8.91E-08 | 1.63E-05 | 1.03E-05 | Col3a1/Aspn/Fbn1/Lrp1/Itgb5/Col1a2/Htra3 | 7 |
| F02 | GO:0071559 | response to transforming growth factor beta | 7/48 | 230/29008 | 1.00E-07 | 1.63E-05 | 1.03E-05 | Col3a1/Aspn/Fbn1/Lrp1/Itgb5/Col1a2/Htra3 | 7 |
| F02 | GO:0031589 | cell-substrate adhesion | 8/48 | 366/29008 | 1.45E-07 | 2.05E-05 | 1.30E-05 | Col3a1/Apod/Lrp1/Itgb5/Fbln2/Cd34/Mmp14/Col1a1 | 8 |
| F02 | GO:0007178 | transmembrane receptor protein serine/threonine kinase signaling pathway | 8/48 | 387/29008 | 2.21E-07 | 2.78E-05 | 1.77E-05 | Col3a1/Aspn/Fbn1/Lrp1/Fstl1/Itgb5/Col1a2/Htra3 | 8 |
| F02 | GO:0071230 | cellular response to amino acid stimulus | 6/48 | 173/29008 | 4.11E-07 | 4.66E-05 | 2.96E-05 | Col3a1/Mmp2/Col6a1/Col1a2/Col5a2/Col1a1 | 6 |
| F02 | GO:0043200 | response to amino acid | 6/48 | 187/29008 | 6.49E-07 | 6.13E-05 | 3.89E-05 | Col3a1/Mmp2/Col6a1/Col1a2/Col5a2/Col1a1 | 6 |
| F02 | GO:0071229 | cellular response to acid chemical | 6/48 | 187/29008 | 6.49E-07 | 6.13E-05 | 3.89E-05 | Col3a1/Mmp2/Col6a1/Col1a2/Col5a2/Col1a1 | 6 |
| F02 | GO:0001101 | response to acid chemical | 6/48 | 209/29008 | 1.24E-06 | 0.000108191 | 6.88E-05 | Col3a1/Mmp2/Col6a1/Col1a2/Col5a2/Col1a1 | 6 |
| F02 | GO:0032963 | collagen metabolic process | 5/48 | 119/29008 | 1.59E-06 | 0.000128478 | 8.16E-05 | Mmp2/Col1a2/Mmp14/Col5a1/Col1a1 | 5 |
| F02 | GO:0007160 | cell-matrix adhesion | 6/48 | 225/29008 | 1.90E-06 | 0.000143839 | 9.14E-05 | Col3a1/Apod/Lrp1/Itgb5/Cd34/Mmp14 | 6 |
| F02 | GO:0051895 | negative regulation of focal adhesion assembly | 3/48 | 17/29008 | 2.84E-06 | 0.000186939 | 0.000118796 | Apod/Lrp1/Mmp14 | 3 |
| F02 | GO:0150118 | negative regulation of cell-substrate junction organization | 3/48 | 17/29008 | 2.84E-06 | 0.000186939 | 0.000118796 | Apod/Lrp1/Mmp14 | 3 |
| F02 | GO:0060348 | bone development | 6/48 | 243/29008 | 2.97E-06 | 0.000186939 | 0.000118796 | Col3a1/Fbn1/Sparc/Bgn/Mmp14/Col1a1 | 6 |
| F02 | GO:0010812 | negative regulation of cell-substrate adhesion | 4/48 | 61/29008 | 3.21E-06 | 0.000191563 | 0.000121735 | Apod/Lrp1/Mmp14/Col1a1 | 4 |
| F02 | GO:0030512 | negative regulation of transforming growth factor beta receptor signaling pathway | 4/48 | 75/29008 | 7.36E-06 | 0.000416725 | 0.000264821 | Aspn/Fbn1/Lrp1/Htra3 | 4 |
| F02 | GO:0043588 | skin development | 6/48 | 294/29008 | 8.84E-06 | 0.000476851 | 0.00030303 | Col3a1/Col1a2/Col5a2/Col5a1/Txnip/Col1a1 | 6 |
| F02 | GO:0090287 | regulation of cellular response to growth factor stimulus | 6/48 | 299/29008 | 9.73E-06 | 0.000500954 | 0.000318347 | Dcn/Aspn/Fbn1/Lrp1/Fstl1/Htra3 | 6 |
| F02 | GO:0045765 | regulation of angiogenesis | 6/48 | 302/29008 | 1.03E-05 | 0.000507115 | 0.000322262 | Dcn/Ramp2/Sparc/Cd34/Adamts1/Serpinf1 | 6 |
| F02 | GO:1901342 | regulation of vasculature development | 6/48 | 306/29008 | 1.11E-05 | 0.000523644 | 0.000332766 | Dcn/Ramp2/Sparc/Cd34/Adamts1/Serpinf1 | 6 |
| F02 | GO:0060343 | trabecula formation | 3/48 | 29/29008 | 1.51E-05 | 0.000683138 | 0.000434121 | Mmp2/Adamts1/Col1a1 | 3 |
| F02 | GO:1901889 | negative regulation of cell junction assembly | 3/48 | 31/29008 | 1.85E-05 | 0.00080617 | 0.000512306 | Apod/Lrp1/Mmp14 | 3 |
| F02 | GO:0040013 | negative regulation of locomotion | 6/48 | 341/29008 | 2.04E-05 | 0.000857816 | 0.000545126 | Col3a1/Dcn/Apod/Lrp1/Sema3c/Serpinf1 | 6 |
| F02 | GO:0016525 | negative regulation of angiogenesis | 4/48 | 110/29008 | 3.35E-05 | 0.001313578 | 0.000834754 | Dcn/Sparc/Adamts1/Serpinf1 | 4 |
| F02 | GO:0001953 | negative regulation of cell-matrix adhesion | 3/48 | 38/29008 | 3.44E-05 | 0.001313578 | 0.000834754 | Apod/Lrp1/Mmp14 | 3 |
| F02 | GO:0010810 | regulation of cell-substrate adhesion | 5/48 | 226/29008 | 3.58E-05 | 0.001313578 | 0.000834754 | Apod/Lrp1/Fbln2/Mmp14/Col1a1 | 5 |
| F02 | GO:2000181 | negative regulation of blood vessel morphogenesis | 4/48 | 112/29008 | 3.59E-05 | 0.001313578 | 0.000834754 | Dcn/Sparc/Adamts1/Serpinf1 | 4 |
| F02 | GO:1901343 | negative regulation of vasculature development | 4/48 | 113/29008 | 3.72E-05 | 0.00131763 | 0.000837329 | Dcn/Sparc/Adamts1/Serpinf1 | 4 |
| F02 | GO:0018149 | peptide cross-linking | 3/48 | 41/29008 | 4.34E-05 | 0.0014889 | 0.000946167 | Col3a1/Dcn/Bgn | 3 |
| F02 | GO:0017015 | regulation of transforming growth factor beta receptor signaling pathway | 4/48 | 121/29008 | 4.86E-05 | 0.001620465 | 0.001029775 | Aspn/Fbn1/Lrp1/Htra3 | 4 |
| F02 | GO:0085029 | extracellular matrix assembly | 3/48 | 43/29008 | 5.01E-05 | 0.001621419 | 0.001030381 | Col3a1/Ramp2/Col1a2 | 3 |
| F02 | GO:1903844 | regulation of cellular response to transforming growth factor beta stimulus | 4/48 | 124/29008 | 5.35E-05 | 0.001683894 | 0.001070083 | Aspn/Fbn1/Lrp1/Htra3 | 4 |
| F02 | GO:0090101 | negative regulation of transmembrane receptor protein serine/threonine kinase signaling pathway | 4/48 | 128/29008 | 6.05E-05 | 0.001823402 | 0.001158737 | Aspn/Fbn1/Lrp1/Htra3 | 4 |
| F02 | GO:0090092 | regulation of transmembrane receptor protein serine/threonine kinase signaling pathway | 5/48 | 253/29008 | 6.12E-05 | 0.001823402 | 0.001158737 | Aspn/Fbn1/Lrp1/Fstl1/Htra3 | 5 |
| F02 | GO:0061383 | trabecula morphogenesis | 3/48 | 56/29008 | 0.00011082 | 0.003219464 | 0.002045908 | Mmp2/Adamts1/Col1a1 | 3 |
| F02 | GO:0030336 | negative regulation of cell migration | 5/48 | 293/29008 | 0.000121929 | 0.003453649 | 0.002194728 | Col3a1/Dcn/Apod/Lrp1/Serpinf1 | 5 |
| F02 | GO:0031102 | neuron projection regeneration | 3/48 | 61/29008 | 0.000143049 | 0.003933787 | 0.002499847 | Mmp2/Lrp1/Matn2 | 3 |
| F02 | GO:2001214 | positive regulation of vasculogenesis | 2/48 | 11/29008 | 0.000146066 | 0.003933787 | 0.002499847 | Ramp2/Cd34 | 2 |
| F02 | GO:2000146 | negative regulation of cell motility | 5/48 | 306/29008 | 0.000149296 | 0.003933787 | 0.002499847 | Col3a1/Dcn/Apod/Lrp1/Serpinf1 | 5 |
| F02 | GO:0007162 | negative regulation of cell adhesion | 5/48 | 310/29008 | 0.000158596 | 0.003978182 | 0.002528059 | Apod/Mmp2/Lrp1/Mmp14/Col1a1 | 5 |
| F02 | GO:0048008 | platelet-derived growth factor receptor signaling pathway | 3/48 | 64/29008 | 0.000165026 | 0.003978182 | 0.002528059 | Apod/Lrp1/Txnip | 3 |
| F02 | GO:0051893 | regulation of focal adhesion assembly | 3/48 | 64/29008 | 0.000165026 | 0.003978182 | 0.002528059 | Apod/Lrp1/Mmp14 | 3 |
| F02 | GO:0090109 | regulation of cell-substrate junction assembly | 3/48 | 64/29008 | 0.000165026 | 0.003978182 | 0.002528059 | Apod/Lrp1/Mmp14 | 3 |
| F02 | GO:0051271 | negative regulation of cellular component movement | 5/48 | 315/29008 | 0.000170838 | 0.004032483 | 0.002562566 | Col3a1/Dcn/Apod/Lrp1/Serpinf1 | 5 |
| F02 | GO:0060350 | endochondral bone morphogenesis | 3/48 | 66/29008 | 0.00018083 | 0.004181223 | 0.002657088 | Col3a1/Mmp14/Col1a1 | 3 |
| F02 | GO:0150116 | regulation of cell-substrate junction organization | 3/48 | 69/29008 | 0.000206326 | 0.004503902 | 0.002862144 | Apod/Lrp1/Mmp14 | 3 |
| F03 | GO:0050680 | negative regulation of epithelial cell proliferation | 2月11日 | 171/29008 | 0.001834863 | 0.069397462 | 0.037953331 | Sparc/Slurp1 | 2 |
| F03 | GO:0043542 | endothelial cell migration | 2月11日 | 221/29008 | 0.003037257 | 0.069397462 | 0.037953331 | Sparc/Fstl1 | 2 |
| F03 | GO:0036462 | TRAIL-activated apoptotic signaling pathway | 1月11日 | 11/29008 | 0.00416408 | 0.069397462 | 0.037953331 | Timp3 | 1 |
| F03 | GO:0019370 | leukotriene biosynthetic process | 1月11日 | 13/29008 | 0.004919489 | 0.069397462 | 0.037953331 | Mgst3 | 1 |
| F03 | GO:1905048 | regulation of metallopeptidase activity | 1月11日 | 13/29008 | 0.004919489 | 0.069397462 | 0.037953331 | Timp3 | 1 |
| F03 | GO:0030336 | negative regulation of cell migration | 2月11日 | 293/29008 | 0.005265612 | 0.069397462 | 0.037953331 | Ifitm1/Slurp1 | 2 |
| F03 | GO:0048672 | positive regulation of collateral sprouting | 1月11日 | 14/29008 | 0.005296999 | 0.069397462 | 0.037953331 | Crabp2 | 1 |
| F03 | GO:0010839 | negative regulation of keratinocyte proliferation | 1月11日 | 15/29008 | 0.005674378 | 0.069397462 | 0.037953331 | Slurp1 | 1 |
| F03 | GO:0010631 | epithelial cell migration | 2月11日 | 306/29008 | 0.005728643 | 0.069397462 | 0.037953331 | Sparc/Fstl1 | 2 |
| F03 | GO:2000146 | negative regulation of cell motility | 2月11日 | 306/29008 | 0.005728643 | 0.069397462 | 0.037953331 | Ifitm1/Slurp1 | 2 |
| F03 | GO:0090132 | epithelium migration | 2月11日 | 308/29008 | 0.005801495 | 0.069397462 | 0.037953331 | Sparc/Fstl1 | 2 |
| F03 | GO:0090130 | tissue migration | 2月11日 | 310/29008 | 0.005874777 | 0.069397462 | 0.037953331 | Sparc/Fstl1 | 2 |
| F03 | GO:1902043 | positive regulation of extrinsic apoptotic signaling pathway via death domain receptors | 1月11日 | 16/29008 | 0.006051627 | 0.069397462 | 0.037953331 | Timp3 | 1 |
| F03 | GO:0051271 | negative regulation of cellular component movement | 2月11日 | 315/29008 | 0.006059855 | 0.069397462 | 0.037953331 | Ifitm1/Slurp1 | 2 |
| F03 | GO:0040013 | negative regulation of locomotion | 2月11日 | 341/29008 | 0.007065123 | 0.069397462 | 0.037953331 | Ifitm1/Slurp1 | 2 |
| F03 | GO:0050884 | neuromuscular process controlling posture | 1月11日 | 19/29008 | 0.007182594 | 0.069397462 | 0.037953331 | Slurp1 | 1 |
| F03 | GO:0051043 | regulation of membrane protein ectodomain proteolysis | 1月11日 | 23/29008 | 0.008688729 | 0.069397462 | 0.037953331 | Timp3 | 1 |
| F03 | GO:0006691 | leukotriene metabolic process | 1月11日 | 24/29008 | 0.009064938 | 0.069397462 | 0.037953331 | Mgst3 | 1 |
| F03 | GO:0048670 | regulation of collateral sprouting | 1月11日 | 24/29008 | 0.009064938 | 0.069397462 | 0.037953331 | Crabp2 | 1 |
| F03 | GO:0043567 | regulation of insulin-like growth factor receptor signaling pathway | 1月11日 | 25/29008 | 0.009441018 | 0.069397462 | 0.037953331 | Igfbp6 | 1 |
| F03 | GO:0050678 | regulation of epithelial cell proliferation | 2月11日 | 403/29008 | 0.009746491 | 0.069397462 | 0.037953331 | Sparc/Slurp1 | 2 |
| F03 | GO:0061484 | hematopoietic stem cell homeostasis | 1月11日 | 26/29008 | 0.009816968 | 0.069397462 | 0.037953331 | Fstl1 | 1 |
| F03 | GO:0042573 | retinoic acid metabolic process | 1月11日 | 28/29008 | 0.010568478 | 0.069397462 | 0.037953331 | Crabp2 | 1 |
| F03 | GO:0046597 | negative regulation of viral entry into host cell | 1月11日 | 30/29008 | 0.011319469 | 0.069397462 | 0.037953331 | Ifitm1 | 1 |
| F03 | GO:0035455 | response to interferon-alpha | 1月11日 | 31/29008 | 0.011694771 | 0.069397462 | 0.037953331 | Ifitm1 | 1 |
| F03 | GO:0001667 | ameboidal-type cell migration | 2月11日 | 444/29008 | 0.011733328 | 0.069397462 | 0.037953331 | Sparc/Fstl1 | 2 |
| F03 | GO:0048668 | collateral sprouting | 1月11日 | 32/29008 | 0.012069943 | 0.069397462 | 0.037953331 | Crabp2 | 1 |
| F03 | GO:0095500 | acetylcholine receptor signaling pathway | 1月11日 | 33/29008 | 0.012444985 | 0.069397462 | 0.037953331 | Ly6c1 | 1 |
| F03 | GO:1903901 | negative regulation of viral life cycle | 1月11日 | 33/29008 | 0.012444985 | 0.069397462 | 0.037953331 | Ifitm1 | 1 |
| F03 | GO:0050673 | epithelial cell proliferation | 2月11日 | 471/29008 | 0.013131919 | 0.069397462 | 0.037953331 | Sparc/Slurp1 | 2 |
| F03 | GO:0010837 | regulation of keratinocyte proliferation | 1月11日 | 35/29008 | 0.013194682 | 0.069397462 | 0.037953331 | Slurp1 | 1 |
| F03 | GO:1905145 | cellular response to acetylcholine | 1月11日 | 35/29008 | 0.013194682 | 0.069397462 | 0.037953331 | Ly6c1 | 1 |
| F03 | GO:0035115 | embryonic forelimb morphogenesis | 1月11日 | 36/29008 | 0.013569336 | 0.069397462 | 0.037953331 | Crabp2 | 1 |
| F03 | GO:0048009 | insulin-like growth factor receptor signaling pathway | 1月11日 | 36/29008 | 0.013569336 | 0.069397462 | 0.037953331 | Igfbp6 | 1 |
| F03 | GO:1905144 | response to acetylcholine | 1月11日 | 36/29008 | 0.013569336 | 0.069397462 | 0.037953331 | Ly6c1 | 1 |
| F03 | GO:0006509 | membrane protein ectodomain proteolysis | 1月11日 | 45/29008 | 0.01693541 | 0.076060388 | 0.041597272 | Timp3 | 1 |
| F03 | GO:0035136 | forelimb morphogenesis | 1月11日 | 45/29008 | 0.01693541 | 0.076060388 | 0.041597272 | Crabp2 | 1 |
| F03 | GO:0042771 | intrinsic apoptotic signaling pathway in response to DNA damage by p53 class mediator | 1月11日 | 45/29008 | 0.01693541 | 0.076060388 | 0.041597272 | Phlda3 | 1 |
| F03 | GO:0051898 | negative regulation of protein kinase B signaling | 1月11日 | 46/29008 | 0.017308773 | 0.076060388 | 0.041597272 | Phlda3 | 1 |
| F03 | GO:0043616 | keratinocyte proliferation | 1月11日 | 47/29008 | 0.017682007 | 0.076060388 | 0.041597272 | Slurp1 | 1 |
| F03 | GO:0098926 | postsynaptic signal transduction | 1月11日 | 49/29008 | 0.018428089 | 0.076060388 | 0.041597272 | Ly6c1 | 1 |
| F03 | GO:0060337 | type I interferon signaling pathway | 1月11日 | 50/29008 | 0.018800936 | 0.076060388 | 0.041597272 | Ifitm1 | 1 |
| F03 | GO:0071357 | cellular response to type I interferon | 1月11日 | 50/29008 | 0.018800936 | 0.076060388 | 0.041597272 | Ifitm1 | 1 |
| F03 | GO:0046456 | icosanoid biosynthetic process | 1月11日 | 51/29008 | 0.019173655 | 0.076060388 | 0.041597272 | Mgst3 | 1 |
| F03 | GO:0046596 | regulation of viral entry into host cell | 1月11日 | 51/29008 | 0.019173655 | 0.076060388 | 0.041597272 | Ifitm1 | 1 |
| F03 | GO:1902041 | regulation of extrinsic apoptotic signaling pathway via death domain receptors | 1月11日 | 52/29008 | 0.019546245 | 0.076060388 | 0.041597272 | Timp3 | 1 |
| F03 | GO:0034340 | response to type I interferon | 1月11日 | 56/29008 | 0.021035319 | 0.077528812 | 0.04240035 | Ifitm1 | 1 |
| F03 | GO:0052372 | modulation by symbiont of entry into host | 1月11日 | 56/29008 | 0.021035319 | 0.077528812 | 0.04240035 | Ifitm1 | 1 |
| F03 | GO:0033619 | membrane protein proteolysis | 1月11日 | 58/29008 | 0.021779084 | 0.077528812 | 0.04240035 | Timp3 | 1 |
| F03 | GO:0045071 | negative regulation of viral genome replication | 1月11日 | 58/29008 | 0.021779084 | 0.077528812 | 0.04240035 | Ifitm1 | 1 |
| F04 | GO:0006418 | tRNA aminoacylation for protein translation | 5月25日 | 40/29008 | 2.00E-10 | 8.75E-08 | 5.35E-08 | Nars/Tars/Sars/Eprs/Aars | 5 |
| F04 | GO:0043039 | tRNA aminoacylation | 5月25日 | 43/29008 | 2.92E-10 | 8.75E-08 | 5.35E-08 | Nars/Tars/Sars/Eprs/Aars | 5 |
| F04 | GO:0043038 | amino acid activation | 5月25日 | 44/29008 | 3.30E-10 | 8.75E-08 | 5.35E-08 | Nars/Tars/Sars/Eprs/Aars | 5 |
| F04 | GO:0006520 | cellular amino acid metabolic process | 6月25日 | 256/29008 | 6.86E-08 | 1.36E-05 | 8.33E-06 | Nars/Asns/Tars/Sars/Eprs/Aars | 6 |
| F04 | GO:0006399 | tRNA metabolic process | 5月25日 | 168/29008 | 2.97E-07 | 4.73E-05 | 2.89E-05 | Nars/Tars/Sars/Eprs/Aars | 5 |
| F04 | GO:0042026 | protein refolding | 3月25日 | 18/29008 | 4.57E-07 | 6.07E-05 | 3.71E-05 | Hspa9/Hspd1/Hspa5 | 3 |
| F04 | GO:0034620 | cellular response to unfolded protein | 4月25日 | 88/29008 | 9.53E-07 | 0.000108319 | 6.62E-05 | Hspa9/Hspd1/Ddit3/Hspa5 | 4 |
| F04 | GO:0035967 | cellular response to topologically incorrect protein | 4月25日 | 108/29008 | 2.16E-06 | 0.000215308 | 0.000131542 | Hspa9/Hspd1/Ddit3/Hspa5 | 4 |
| F04 | GO:0006986 | response to unfolded protein | 4月25日 | 116/29008 | 2.88E-06 | 0.000254524 | 0.000155502 | Hspa9/Hspd1/Ddit3/Hspa5 | 4 |
| F04 | GO:0035966 | response to topologically incorrect protein | 4月25日 | 138/29008 | 5.74E-06 | 0.000456842 | 0.000279108 | Hspa9/Hspd1/Ddit3/Hspa5 | 4 |
| F04 | GO:0006457 | protein folding | 4月25日 | 163/29008 | 1.11E-05 | 0.00080215 | 0.000490073 | Hspa9/Hspd1/Hspa5/Cryab | 4 |
| F04 | GO:0042594 | response to starvation | 4月25日 | 184/29008 | 1.79E-05 | 0.001184539 | 0.000723693 | Asns/Eif4ebp1/Ddit3/Hspa5 | 4 |
| F04 | GO:0034660 | ncRNA metabolic process | 5月25日 | 453/29008 | 3.73E-05 | 0.002283419 | 0.001395054 | Nars/Tars/Sars/Eprs/Aars | 5 |
| F04 | GO:0006983 | ER overload response | 2月25日 | 13/29008 | 5.53E-05 | 0.003039149 | 0.001856767 | Ddit3/Hspa5 | 2 |
| F04 | GO:1903320 | regulation of protein modification by small protein conjugation or removal | 4月25日 | 248/29008 | 5.73E-05 | 0.003039149 | 0.001856767 | Sqstm1/Gnl3/Trib3/Hspa5 | 4 |
| F04 | GO:0031667 | response to nutrient levels | 4月25日 | 358/29008 | 0.000235071 | 0.011694785 | 0.007144923 | Asns/Eif4ebp1/Ddit3/Hspa5 | 4 |
| F04 | GO:1903322 | positive regulation of protein modification by small protein conjugation or removal | 3月25日 | 145/29008 | 0.00025955 | 0.012153036 | 0.007424891 | Gnl3/Trib3/Hspa5 | 3 |
| F04 | GO:0009991 | response to extracellular stimulus | 4月25日 | 391/29008 | 0.000328607 | 0.014531747 | 0.008878163 | Asns/Eif4ebp1/Ddit3/Hspa5 | 4 |
| F04 | GO:0051085 | chaperone cofactor-dependent protein refolding | 2月25日 | 32/29008 | 0.000348119 | 0.014584335 | 0.008910292 | Hspa9/Hspa5 | 2 |
| F04 | GO:0072594 | establishment of protein localization to organelle | 4月25日 | 418/29008 | 0.000422965 | 0.016641714 | 0.010167247 | Sqstm1/Hspd1/Ddit3/Hspa5 | 4 |
| F04 | GO:0006458 | 'de novo' protein folding | 2月25日 | 37/29008 | 0.000466201 | 0.016641714 | 0.010167247 | Hspa9/Hspa5 | 2 |
| F04 | GO:0051084 | 'de novo' posttranslational protein folding | 2月25日 | 37/29008 | 0.000466201 | 0.016641714 | 0.010167247 | Hspa9/Hspa5 | 2 |
| F04 | GO:0030902 | hindbrain development | 3月25日 | 179/29008 | 0.000480854 | 0.016641714 | 0.010167247 | Atf5/Aars/Hspa5 | 3 |
| F04 | GO:0021680 | cerebellar Purkinje cell layer development | 2月25日 | 39/29008 | 0.000518153 | 0.017185421 | 0.010499424 | Aars/Hspa5 | 2 |
| F04 | GO:0006984 | ER-nucleus signaling pathway | 2月25日 | 43/29008 | 0.000630102 | 0.020062444 | 0.01225714 | Ddit3/Hspa5 | 2 |
| F04 | GO:0032869 | cellular response to insulin stimulus | 3月25日 | 208/29008 | 0.000743902 | 0.022452774 | 0.013717511 | Trib3/Eif4ebp1/Eprs | 3 |
| F04 | GO:0001666 | response to hypoxia | 3月25日 | 214/29008 | 0.000807728 | 0.022452774 | 0.013717511 | Eif4ebp1/Cited2/Cryab | 3 |
| F04 | GO:0031396 | regulation of protein ubiquitination | 3月25日 | 214/29008 | 0.000807728 | 0.022452774 | 0.013717511 | Sqstm1/Trib3/Hspa5 | 3 |
| F04 | GO:0042149 | cellular response to glucose starvation | 2月25日 | 49/29008 | 0.000818003 | 0.022452774 | 0.013717511 | Asns/Hspa5 | 2 |
| F04 | GO:0043618 | regulation of transcription from RNA polymerase II promoter in response to stress | 2月25日 | 50/29008 | 0.000851637 | 0.022596767 | 0.013805483 | Cited2/Ddit3 | 2 |
| F04 | GO:0071560 | cellular response to transforming growth factor beta stimulus | 3月25日 | 226/29008 | 0.00094562 | 0.023356429 | 0.014269598 | Cited2/Hspa5/Ankrd1 | 3 |
| F04 | GO:0065002 | intracellular protein transmembrane transport | 2月25日 | 53/29008 | 0.000956489 | 0.023356429 | 0.014269598 | Hspd1/Hspa5 | 2 |
| F04 | GO:0071559 | response to transforming growth factor beta | 3月25日 | 230/29008 | 0.000994694 | 0.023356429 | 0.014269598 | Cited2/Hspa5/Ankrd1 | 3 |
| F04 | GO:0043620 | regulation of DNA-templated transcription in response to stress | 2月25日 | 55/29008 | 0.001029672 | 0.023356429 | 0.014269598 | Cited2/Ddit3 | 2 |
| F04 | GO:2000378 | negative regulation of reactive oxygen species metabolic process | 2月25日 | 55/29008 | 0.001029672 | 0.023356429 | 0.014269598 | Hspd1/Cryab | 2 |
| F04 | GO:0071806 | protein transmembrane transport | 2月25日 | 57/29008 | 0.00110547 | 0.023356429 | 0.014269598 | Hspd1/Hspa5 | 2 |
| F04 | GO:0036293 | response to decreased oxygen levels | 3月25日 | 241/29008 | 0.001137906 | 0.023356429 | 0.014269598 | Eif4ebp1/Cited2/Cryab | 3 |
| F04 | GO:0045599 | negative regulation of fat cell differentiation | 2月25日 | 58/29008 | 0.001144348 | 0.023356429 | 0.014269598 | Trib3/Ddit3 | 2 |
| F04 | GO:0061077 | chaperone-mediated protein folding | 2月25日 | 58/29008 | 0.001144348 | 0.023356429 | 0.014269598 | Hspa9/Hspa5 | 2 |
| F04 | GO:0032868 | response to insulin | 3月25日 | 244/29008 | 0.001179105 | 0.023464186 | 0.014335432 | Trib3/Eif4ebp1/Eprs | 3 |
| F04 | GO:0034976 | response to endoplasmic reticulum stress | 3月25日 | 254/29008 | 0.001323218 | 0.025689793 | 0.015695166 | Trib3/Ddit3/Hspa5 | 3 |
| F04 | GO:0070059 | intrinsic apoptotic signaling pathway in response to endoplasmic reticulum stress | 2月25日 | 64/29008 | 0.001391237 | 0.026190006 | 0.016000771 | Trib3/Ddit3 | 2 |
| F04 | GO:0045444 | fat cell differentiation | 3月25日 | 260/29008 | 0.001414787 | 0.026190006 | 0.016000771 | Atf5/Trib3/Ddit3 | 3 |
| F04 | GO:0071375 | cellular response to peptide hormone stimulus | 3月25日 | 264/29008 | 0.001477997 | 0.02673831 | 0.016335757 | Trib3/Eif4ebp1/Eprs | 3 |
| F04 | GO:0030968 | endoplasmic reticulum unfolded protein response | 2月25日 | 67/29008 | 0.001523394 | 0.026947142 | 0.016463342 | Ddit3/Hspa5 | 2 |
| F04 | GO:0021695 | cerebellar cortex development | 2月25日 | 69/29008 | 0.001614706 | 0.027941428 | 0.017070801 | Aars/Hspa5 | 2 |
| F04 | GO:0043523 | regulation of neuron apoptotic process | 3月25日 | 276/29008 | 0.001678219 | 0.028422595 | 0.01736477 | Hspd1/Aars/Ddit3 | 3 |
| F04 | GO:0071496 | cellular response to external stimulus | 3月25日 | 295/29008 | 0.002028678 | 0.033274219 | 0.020328867 | Asns/Hspa5/Ankrd1 | 3 |
| F04 | GO:0070482 | response to oxygen levels | 3月25日 | 296/29008 | 0.002048287 | 0.033274219 | 0.020328867 | Eif4ebp1/Cited2/Cryab | 3 |
| F04 | GO:1901653 | cellular response to peptide | 3月25日 | 308/29008 | 0.0022929 | 0.036117597 | 0.022066027 | Trib3/Eif4ebp1/Eprs | 3 |
| F05 | GO:0043620 | regulation of DNA-templated transcription in response to stress | 8/45 | 55/29008 | 2.00E-14 | 3.88E-11 | 1.78E-11 | Egr1/Atf3/Dnajb1/Jun/Klf2/Tcim/Ppp1r15a/Cited2 | 8 |
| F05 | GO:0035914 | skeletal muscle cell differentiation | 8/45 | 89/29008 | 1.12E-12 | 8.12E-10 | 3.72E-10 | Fos/Egr1/Atf3/Nr4a1/Btg2/Myc/Nupr1/Cited2 | 8 |
| F05 | GO:0043618 | regulation of transcription from RNA polymerase II promoter in response to stress | 7/45 | 50/29008 | 1.26E-12 | 8.12E-10 | 3.72E-10 | Egr1/Atf3/Dnajb1/Jun/Klf2/Ppp1r15a/Cited2 | 7 |
| F05 | GO:0007519 | skeletal muscle tissue development | 8/45 | 201/29008 | 8.00E-10 | 3.87E-07 | 1.77E-07 | Fos/Egr1/Atf3/Nr4a1/Btg2/Myc/Nupr1/Cited2 | 8 |
| F05 | GO:0060538 | skeletal muscle organ development | 8/45 | 212/29008 | 1.22E-09 | 4.71E-07 | 2.16E-07 | Fos/Egr1/Atf3/Nr4a1/Btg2/Myc/Nupr1/Cited2 | 8 |
| F05 | GO:0097201 | negative regulation of transcription from RNA polymerase II promoter in response to stress | 4/45 | 12/29008 | 2.48E-09 | 7.97E-07 | 3.66E-07 | Dnajb1/Jun/Ppp1r15a/Cited2 | 4 |
| F05 | GO:0070371 | ERK1 and ERK2 cascade | 9/45 | 349/29008 | 2.88E-09 | 7.97E-07 | 3.66E-07 | Atf3/Jun/Dusp1/Klf4/Myc/Errfi1/Dusp6/Sox9/Ackr3 | 9 |
| F05 | GO:0009896 | positive regulation of catabolic process | 10/45 | 494/29008 | 3.51E-09 | 8.48E-07 | 3.89E-07 | Zfp36/Btg2/Ier3/Trib1/Hspa1b/Myc/Nupr1/Hsp90aa1/Sox9/Plk2 | 10 |
| F05 | GO:0033002 | muscle cell proliferation | 8/45 | 255/29008 | 5.20E-09 | 1.12E-06 | 5.13E-07 | Egr1/Jun/Klf4/Trib1/Myc/Adamts1/Cited2/Nppb | 8 |
| F05 | GO:0042326 | negative regulation of phosphorylation | 9/45 | 396/29008 | 8.64E-09 | 1.67E-06 | 7.66E-07 | Jun/Dusp1/Socs3/Ppp1r15a/Ier3/Trib1/Errfi1/Dusp6/Nupr1 | 9 |
| F05 | GO:0050678 | regulation of epithelial cell proliferation | 9/45 | 403/29008 | 1.00E-08 | 1.75E-06 | 8.02E-07 | Jun/Zfp36/Nr4a1/Myc/Errfi1/Hes1/Nupr1/Sox9/Nppb | 9 |
| F05 | GO:0048660 | regulation of smooth muscle cell proliferation | 7/45 | 178/29008 | 1.08E-08 | 1.75E-06 | 8.02E-07 | Egr1/Jun/Klf4/Trib1/Myc/Adamts1/Nppb | 7 |
| F05 | GO:0048659 | smooth muscle cell proliferation | 7/45 | 184/29008 | 1.36E-08 | 2.03E-06 | 9.31E-07 | Egr1/Jun/Klf4/Trib1/Myc/Adamts1/Nppb | 7 |
| F05 | GO:0030099 | myeloid cell differentiation | 9/45 | 426/29008 | 1.62E-08 | 2.24E-06 | 1.03E-06 | Fos/Jun/Klf2/Junb/Zfp36/Trib1/Myc/Nfkbia/Cited2 | 9 |
| F05 | GO:0010563 | negative regulation of phosphorus metabolic process | 9/45 | 453/29008 | 2.75E-08 | 3.32E-06 | 1.52E-06 | Jun/Dusp1/Socs3/Ppp1r15a/Ier3/Trib1/Errfi1/Dusp6/Nupr1 | 9 |
| F05 | GO:0045936 | negative regulation of phosphate metabolic process | 9/45 | 453/29008 | 2.75E-08 | 3.32E-06 | 1.52E-06 | Jun/Dusp1/Socs3/Ppp1r15a/Ier3/Trib1/Errfi1/Dusp6/Nupr1 | 9 |
| F05 | GO:0050673 | epithelial cell proliferation | 9/45 | 471/29008 | 3.84E-08 | 4.37E-06 | 2.00E-06 | Jun/Zfp36/Nr4a1/Myc/Errfi1/Hes1/Nupr1/Sox9/Nppb | 9 |
| F05 | GO:0048608 | reproductive structure development | 9/45 | 480/29008 | 4.51E-08 | 4.62E-06 | 2.12E-06 | Socs3/Junb/Ubb/Serpine1/Adamts1/Hes1/Nupr1/Sox9/Cited2 | 9 |
| F05 | GO:0045732 | positive regulation of protein catabolic process | 7/45 | 221/29008 | 4.81E-08 | 4.62E-06 | 2.12E-06 | Ier3/Trib1/Hspa1b/Nupr1/Hsp90aa1/Sox9/Plk2 | 7 |
| F05 | GO:0061458 | reproductive system development | 9/45 | 484/29008 | 4.84E-08 | 4.62E-06 | 2.12E-06 | Socs3/Junb/Ubb/Serpine1/Adamts1/Hes1/Nupr1/Sox9/Cited2 | 9 |
| F05 | GO:0060537 | muscle tissue development | 9/45 | 486/29008 | 5.01E-08 | 4.62E-06 | 2.12E-06 | Fos/Egr1/Atf3/Nr4a1/Btg2/Myc/Nupr1/Sox9/Cited2 | 9 |
| F05 | GO:0007517 | muscle organ development | 8/45 | 360/29008 | 7.52E-08 | 6.61E-06 | 3.03E-06 | Fos/Egr1/Atf3/Nr4a1/Btg2/Myc/Nupr1/Cited2 | 8 |
| F05 | GO:0042176 | regulation of protein catabolic process | 8/45 | 389/29008 | 1.36E-07 | 1.12E-05 | 5.12E-06 | Ubb/Ier3/Trib1/Hspa1b/Nupr1/Hsp90aa1/Sox9/Plk2 | 8 |
| F05 | GO:0071277 | cellular response to calcium ion | 5/45 | 78/29008 | 1.39E-07 | 1.12E-05 | 5.12E-06 | Fos/Fosb/Jun/Junb/Jund | 5 |
| F05 | GO:1903706 | regulation of hemopoiesis | 8/45 | 398/29008 | 1.62E-07 | 1.25E-05 | 5.75E-06 | Fos/Jun/Zfp36/Tcim/Trib1/Myc/Nfkbia/Hsp90aa1 | 8 |
| F05 | GO:0070373 | negative regulation of ERK1 and ERK2 cascade | 5/45 | 83/29008 | 1.90E-07 | 1.36E-05 | 6.23E-06 | Atf3/Dusp1/Klf4/Errfi1/Dusp6 | 5 |
| F05 | GO:1902893 | regulation of pri-miRNA transcription by RNA polymerase II | 5/45 | 83/29008 | 1.90E-07 | 1.36E-05 | 6.23E-06 | Fos/Egr1/Jun/Klf4/Sox9 | 5 |
| F05 | GO:0061614 | pri-miRNA transcription by RNA polymerase II | 5/45 | 85/29008 | 2.14E-07 | 1.48E-05 | 6.77E-06 | Fos/Egr1/Jun/Klf4/Sox9 | 5 |
| F05 | GO:0031331 | positive regulation of cellular catabolic process | 8/45 | 425/29008 | 2.67E-07 | 1.78E-05 | 8.16E-06 | Zfp36/Btg2/Trib1/Hspa1b/Myc/Nupr1/Hsp90aa1/Plk2 | 8 |
| F05 | GO:0030336 | negative regulation of cell migration | 7/45 | 293/29008 | 3.26E-07 | 2.10E-05 | 9.65E-06 | Dusp1/Klf4/Rhob/Has1/Trib1/Serpine1/Cited2 | 7 |
| F05 | GO:0014910 | regulation of smooth muscle cell migration | 5/45 | 94/29008 | 3.54E-07 | 2.21E-05 | 1.01E-05 | Egr1/Trib1/Serpine1/Myc/Adamts1 | 5 |
| F05 | GO:0001667 | ameboidal-type cell migration | 8/45 | 444/29008 | 3.71E-07 | 2.25E-05 | 1.03E-05 | Jun/Nr4a1/Klf4/Rhob/Has1/Serpine1/Sox9/Plk2 | 8 |
| F05 | GO:0045765 | regulation of angiogenesis | 7/45 | 302/29008 | 4.00E-07 | 2.34E-05 | 1.08E-05 | Klf2/Klf4/Rhob/Ppp1r15a/Serpine1/Adamts1/Plk2 | 7 |
| F05 | GO:0010631 | epithelial cell migration | 7/45 | 306/29008 | 4.37E-07 | 2.35E-05 | 1.08E-05 | Jun/Nr4a1/Klf4/Rhob/Serpine1/Sox9/Plk2 | 7 |
| F05 | GO:1901342 | regulation of vasculature development | 7/45 | 306/29008 | 4.37E-07 | 2.35E-05 | 1.08E-05 | Klf2/Klf4/Rhob/Ppp1r15a/Serpine1/Adamts1/Plk2 | 7 |
| F05 | GO:2000146 | negative regulation of cell motility | 7/45 | 306/29008 | 4.37E-07 | 2.35E-05 | 1.08E-05 | Dusp1/Klf4/Rhob/Has1/Trib1/Serpine1/Cited2 | 7 |
| F05 | GO:0090132 | epithelium migration | 7/45 | 308/29008 | 4.56E-07 | 2.39E-05 | 1.09E-05 | Jun/Nr4a1/Klf4/Rhob/Serpine1/Sox9/Plk2 | 7 |
| F05 | GO:0090130 | tissue migration | 7/45 | 310/29008 | 4.77E-07 | 2.43E-05 | 1.11E-05 | Jun/Nr4a1/Klf4/Rhob/Serpine1/Sox9/Plk2 | 7 |
| F05 | GO:0014706 | striated muscle tissue development | 8/45 | 461/29008 | 4.93E-07 | 2.45E-05 | 1.12E-05 | Fos/Egr1/Atf3/Nr4a1/Btg2/Myc/Nupr1/Cited2 | 8 |
| F05 | GO:0051271 | negative regulation of cellular component movement | 7/45 | 315/29008 | 5.31E-07 | 2.57E-05 | 1.18E-05 | Dusp1/Klf4/Rhob/Has1/Trib1/Serpine1/Cited2 | 7 |
| F05 | GO:0014909 | smooth muscle cell migration | 5/45 | 103/29008 | 5.58E-07 | 2.64E-05 | 1.21E-05 | Egr1/Trib1/Serpine1/Myc/Adamts1 | 5 |
| F05 | GO:0070372 | regulation of ERK1 and ERK2 cascade | 7/45 | 326/29008 | 6.68E-07 | 3.08E-05 | 1.41E-05 | Atf3/Jun/Dusp1/Klf4/Errfi1/Dusp6/Ackr3 | 7 |
| F05 | GO:0016525 | negative regulation of angiogenesis | 5/45 | 110/29008 | 7.75E-07 | 3.49E-05 | 1.60E-05 | Klf2/Klf4/Serpine1/Adamts1/Plk2 | 5 |
| F05 | GO:2000181 | negative regulation of blood vessel morphogenesis | 5/45 | 112/29008 | 8.47E-07 | 3.73E-05 | 1.71E-05 | Klf2/Klf4/Serpine1/Adamts1/Plk2 | 5 |
| F05 | GO:1901343 | negative regulation of vasculature development | 5/45 | 113/29008 | 8.85E-07 | 3.79E-05 | 1.74E-05 | Klf2/Klf4/Serpine1/Adamts1/Plk2 | 5 |
| F05 | GO:0040013 | negative regulation of locomotion | 7/45 | 341/29008 | 9.02E-07 | 3.79E-05 | 1.74E-05 | Dusp1/Klf4/Rhob/Has1/Trib1/Serpine1/Cited2 | 7 |
| F05 | GO:0014812 | muscle cell migration | 5/45 | 117/29008 | 1.05E-06 | 4.24E-05 | 1.95E-05 | Egr1/Trib1/Serpine1/Myc/Adamts1 | 5 |
| F05 | GO:0001933 | negative regulation of protein phosphorylation | 7/45 | 349/29008 | 1.05E-06 | 4.24E-05 | 1.95E-05 | Jun/Dusp1/Socs3/Ppp1r15a/Trib1/Errfi1/Dusp6 | 7 |
| F05 | GO:0045637 | regulation of myeloid cell differentiation | 6/45 | 219/29008 | 1.10E-06 | 4.35E-05 | 1.99E-05 | Fos/Jun/Zfp36/Trib1/Myc/Nfkbia | 6 |
| F05 | GO:0061469 | regulation of type B pancreatic cell proliferation | 3/45 | 15/29008 | 1.57E-06 | 6.06E-05 | 2.78E-05 | Nr4a1/Errfi1/Nupr1 | 3 |
| F06 | GO:0006457 | protein folding | 5月28日 | 163/29008 | 4.66E-07 | 0.000390766 | 0.000241498 | Calr/Cryab/Hspa5/Hspe1/Hsph1 | 5 |
| F06 | GO:0051085 | chaperone cofactor-dependent protein refolding | 3月28日 | 32/29008 | 3.92E-06 | 0.001282466 | 0.00079258 | Hspa5/Hspe1/Hsph1 | 3 |
| F06 | GO:0006458 | 'de novo' protein folding | 3月28日 | 37/29008 | 6.12E-06 | 0.001282466 | 0.00079258 | Hspa5/Hspe1/Hsph1 | 3 |
| F06 | GO:0051084 | 'de novo' posttranslational protein folding | 3月28日 | 37/29008 | 6.12E-06 | 0.001282466 | 0.00079258 | Hspa5/Hspe1/Hsph1 | 3 |
| F06 | GO:2000677 | regulation of transcription regulatory region DNA binding | 3月28日 | 53/29008 | 1.83E-05 | 0.002870998 | 0.001774313 | Ddit3/H1f0/Klf4 | 3 |
| F06 | GO:2001242 | regulation of intrinsic apoptotic signaling pathway | 4月28日 | 176/29008 | 2.39E-05 | 0.002870998 | 0.001774313 | Nupr1/Ddit3/Ackr3/Hsph1 | 4 |
| F06 | GO:0061077 | chaperone-mediated protein folding | 3月28日 | 58/29008 | 2.40E-05 | 0.002870998 | 0.001774313 | Hspa5/Hspe1/Hsph1 | 3 |
| F06 | GO:1901214 | regulation of neuron death | 5月28日 | 384/29008 | 3.03E-05 | 0.003172941 | 0.001960918 | Nupr1/Mt1/Ddit3/Gpnmb/Hsph1 | 5 |
| F06 | GO:2001233 | regulation of apoptotic signaling pathway | 5月28日 | 402/29008 | 3.77E-05 | 0.003371951 | 0.002083909 | Nupr1/Ddit3/Timp3/Ackr3/Hsph1 | 5 |
| F06 | GO:0097501 | stress response to metal ion | 2月28日 | 10/29008 | 4.02E-05 | 0.003371951 | 0.002083909 | Mt2/Mt1 | 2 |
| F06 | GO:0070997 | neuron death | 5月28日 | 428/29008 | 5.07E-05 | 0.003865547 | 0.002388957 | Nupr1/Mt1/Ddit3/Gpnmb/Hsph1 | 5 |
| F06 | GO:0006983 | ER overload response | 2月28日 | 13/29008 | 6.96E-05 | 0.004861873 | 0.0030047 | Ddit3/Hspa5 | 2 |
| F06 | GO:0071294 | cellular response to zinc ion | 2月28日 | 14/29008 | 8.12E-05 | 0.005232736 | 0.003233898 | Mt2/Mt1 | 2 |
| F06 | GO:0034976 | response to endoplasmic reticulum stress | 4月28日 | 254/29008 | 9.96E-05 | 0.005963272 | 0.003685379 | Nupr1/Calr/Ddit3/Hspa5 | 4 |
| F06 | GO:0071280 | cellular response to copper ion | 2月28日 | 18/29008 | 0.000136157 | 0.007182766 | 0.004439042 | Mt2/Mt1 | 2 |
| F06 | GO:0043523 | regulation of neuron apoptotic process | 4月28日 | 276/29008 | 0.000137141 | 0.007182766 | 0.004439042 | Nupr1/Mt1/Ddit3/Hsph1 | 4 |
| F06 | GO:0001818 | negative regulation of cytokine production | 4月28日 | 292/29008 | 0.000170209 | 0.007939041 | 0.004906429 | Ddit3/Gpnmb/Klf4/Bst2 | 4 |
| F06 | GO:0030336 | negative regulation of cell migration | 4月28日 | 293/29008 | 0.000172451 | 0.007939041 | 0.004906429 | Calr/Klf4/Bst2/Slurp1 | 4 |
| F06 | GO:0006986 | response to unfolded protein | 3月28日 | 116/29008 | 0.000189754 | 0.007939041 | 0.004906429 | Ddit3/Hspa5/Hsph1 | 3 |
| F06 | GO:2000146 | negative regulation of cell motility | 4月28日 | 306/29008 | 0.000203574 | 0.007939041 | 0.004906429 | Calr/Klf4/Bst2/Slurp1 | 4 |
| F06 | GO:0097193 | intrinsic apoptotic signaling pathway | 4月28日 | 307/29008 | 0.000206125 | 0.007939041 | 0.004906429 | Nupr1/Ddit3/Ackr3/Hsph1 | 4 |
| F06 | GO:0051402 | neuron apoptotic process | 4月28日 | 309/29008 | 0.000211296 | 0.007939041 | 0.004906429 | Nupr1/Mt1/Ddit3/Hsph1 | 4 |
| F06 | GO:0009636 | response to toxic substance | 3月28日 | 123/29008 | 0.000225537 | 0.007939041 | 0.004906429 | Nupr1/Mt2/Mt1 | 3 |
| F06 | GO:0051271 | negative regulation of cellular component movement | 4月28日 | 315/29008 | 0.000227371 | 0.007939041 | 0.004906429 | Calr/Klf4/Bst2/Slurp1 | 4 |
| F06 | GO:0051101 | regulation of DNA binding | 3月28日 | 127/29008 | 0.000247817 | 0.007956843 | 0.004917431 | Ddit3/H1f0/Klf4 | 3 |
| F06 | GO:0070372 | regulation of ERK1 and ERK2 cascade | 4月28日 | 326/29008 | 0.000259108 | 0.007956843 | 0.004917431 | Timp3/Gpnmb/Ackr3/Klf4 | 4 |
| F06 | GO:0007263 | nitric oxide mediated signal transduction | 2月28日 | 25/29008 | 0.000265861 | 0.007956843 | 0.004917431 | Mt2/Mt1 | 2 |
| F06 | GO:2000679 | positive regulation of transcription regulatory region DNA binding | 2月28日 | 25/29008 | 0.000265861 | 0.007956843 | 0.004917431 | H1f0/Klf4 | 2 |
| F06 | GO:0071276 | cellular response to cadmium ion | 2月28日 | 26/29008 | 0.000287844 | 0.008271672 | 0.005111999 | Mt2/Mt1 | 2 |
| F06 | GO:0040013 | negative regulation of locomotion | 4月28日 | 341/29008 | 0.000307375 | 0.008271672 | 0.005111999 | Calr/Klf4/Bst2/Slurp1 | 4 |
| F06 | GO:0035966 | response to topologically incorrect protein | 3月28日 | 138/29008 | 0.000316302 | 0.008271672 | 0.005111999 | Ddit3/Hspa5/Hsph1 | 3 |
| F06 | GO:0006882 | cellular zinc ion homeostasis | 2月28日 | 28/29008 | 0.000334386 | 0.008271672 | 0.005111999 | Mt2/Mt1 | 2 |
| F06 | GO:0046688 | response to copper ion | 2月28日 | 28/29008 | 0.000334386 | 0.008271672 | 0.005111999 | Mt2/Mt1 | 2 |
| F06 | GO:0070371 | ERK1 and ERK2 cascade | 4月28日 | 349/29008 | 0.000335605 | 0.008271672 | 0.005111999 | Timp3/Gpnmb/Ackr3/Klf4 | 4 |
| F06 | GO:0055069 | zinc ion homeostasis | 2月28日 | 29/29008 | 0.00035894 | 0.008594061 | 0.00531124 | Mt2/Mt1 | 2 |
| F06 | GO:0045786 | negative regulation of cell cycle | 4月28日 | 367/29008 | 0.000405866 | 0.009447661 | 0.005838775 | Nupr1/Gpnmb/Atf5/Klf4 | 4 |
| F06 | GO:2001235 | positive regulation of apoptotic signaling pathway | 3月28日 | 157/29008 | 0.000461313 | 0.01044812 | 0.006457072 | Nupr1/Ddit3/Timp3 | 3 |
| F06 | GO:0043433 | negative regulation of DNA-binding transcription factor activity | 3月28日 | 167/29008 | 0.000552265 | 0.011620008 | 0.007181314 | Nupr1/Ddit3/Klf4 | 3 |
| F06 | GO:0071248 | cellular response to metal ion | 3月28日 | 167/29008 | 0.000552265 | 0.011620008 | 0.007181314 | Mt2/Mt1/Jund | 3 |
| F06 | GO:0010043 | response to zinc ion | 2月28日 | 36/29008 | 0.000554654 | 0.011620008 | 0.007181314 | Mt2/Mt1 | 2 |
| F06 | GO:0050680 | negative regulation of epithelial cell proliferation | 3月28日 | 171/29008 | 0.00059163 | 0.012092343 | 0.007473223 | Nupr1/Zfas1/Slurp1 | 3 |
| F06 | GO:0046686 | response to cadmium ion | 2月28日 | 38/29008 | 0.000618186 | 0.012334274 | 0.007622739 | Mt2/Mt1 | 2 |
| F06 | GO:0072594 | establishment of protein localization to organelle | 4月28日 | 418/29008 | 0.000661651 | 0.012894499 | 0.007968966 | Sqstm1/Ddit3/Hspa5/Hsph1 | 4 |
| F06 | GO:0010951 | negative regulation of endopeptidase activity | 3月28日 | 187/29008 | 0.000766945 | 0.014422545 | 0.008913318 | Cryab/Timp3/Klf4 | 3 |
| F06 | GO:0043409 | negative regulation of MAPK cascade | 3月28日 | 189/29008 | 0.000790933 | 0.014422545 | 0.008913318 | Timp3/Klf4/Hsph1 | 3 |
| F06 | GO:0006984 | ER-nucleus signaling pathway | 2月28日 | 43/29008 | 0.000791691 | 0.014422545 | 0.008913318 | Ddit3/Hspa5 | 2 |
| F06 | GO:0051898 | negative regulation of protein kinase B signaling | 2月28日 | 46/29008 | 0.000905797 | 0.016150177 | 0.009981016 | Ddit3/Klf4 | 2 |
| F06 | GO:0098754 | detoxification | 2月28日 | 58/29008 | 0.001436336 | 0.02507603 | 0.015497308 | Mt2/Mt1 | 2 |
| F06 | GO:0043388 | positive regulation of DNA binding | 2月28日 | 59/29008 | 0.001485848 | 0.025227638 | 0.015591003 | H1f0/Klf4 | 2 |
| F06 | GO:0071241 | cellular response to inorganic substance | 3月28日 | 237/29008 | 0.001516957 | 0.025227638 | 0.015591003 | Mt2/Mt1/Jund | 3 |
| Cd14+ Monocytes | GO:0002478 | antigen processing and presentation of exogenous peptide antigen | 9/49 | 33/29008 | 1.92E-18 | 3.73E-15 | 1.88E-15 | Cd74/H2-Aa/H2-Eb1/H2-Ab1/Ctss/Fcer1g/H2-DMa/Fcgr3/Fcgr2b | 9 |
| Cd14+ Monocytes | GO:0019886 | antigen processing and presentation of exogenous peptide antigen via MHC class II | 8/49 | 20/29008 | 4.50E-18 | 4.37E-15 | 2.21E-15 | Cd74/H2-Aa/H2-Eb1/H2-Ab1/Ctss/Fcer1g/H2-DMa/Fcgr2b | 8 |
| Cd14+ Monocytes | GO:0019884 | antigen processing and presentation of exogenous antigen | 9/49 | 40/29008 | 1.35E-17 | 8.74E-15 | 4.42E-15 | Cd74/H2-Aa/H2-Eb1/H2-Ab1/Ctss/Fcer1g/H2-DMa/Fcgr3/Fcgr2b | 9 |
| Cd14+ Monocytes | GO:0002683 | negative regulation of immune system process | 16/49 | 460/29008 | 2.56E-17 | 1.02E-14 | 5.13E-15 | Cd74/H2-Aa/H2-Ab1/Fcer1g/Tyrobp/Dusp1/C1qc/Arg1/Fcgr2b/Id2/Mafb/Mmp12/Laptm5/Cd68/Cebpb/Ccl2 | 16 |
| Cd14+ Monocytes | GO:0002495 | antigen processing and presentation of peptide antigen via MHC class II | 8/49 | 24/29008 | 2.62E-17 | 1.02E-14 | 5.13E-15 | Cd74/H2-Aa/H2-Eb1/H2-Ab1/Ctss/Fcer1g/H2-DMa/Fcgr2b | 8 |
| Cd14+ Monocytes | GO:0002504 | antigen processing and presentation of peptide or polysaccharide antigen via MHC class II | 8/49 | 26/29008 | 5.54E-17 | 1.79E-14 | 9.07E-15 | Cd74/H2-Aa/H2-Eb1/H2-Ab1/Ctss/Fcer1g/H2-DMa/Fcgr2b | 8 |
| Cd14+ Monocytes | GO:0032103 | positive regulation of response to external stimulus | 15/49 | 427/29008 | 2.58E-16 | 7.16E-14 | 3.62E-14 | Cd74/Ctss/Fcer1g/Tyrobp/Ctsc/Ccl3/Fcgr3/Arg1/Alox5ap/Clec4n/Mmp12/Il1b/Cebpb/Ccl2/Ccl7 | 15 |
| Cd14+ Monocytes | GO:0031349 | positive regulation of defense response | 13/49 | 273/29008 | 6.62E-16 | 1.61E-13 | 8.12E-14 | Cd74/Ctss/Fcer1g/Tyrobp/Ctsc/Ccl3/Fcgr3/Arg1/Alox5ap/Clec4n/Mmp12/Il1b/Cebpb | 13 |
| Cd14+ Monocytes | GO:0002449 | lymphocyte mediated immunity | 15/49 | 477/29008 | 1.32E-15 | 2.55E-13 | 1.29E-13 | Cd74/H2-Aa/H2-Eb1/H2-Ab1/C1qb/Fcer1g/Ctsc/C1qc/H2-DMa/Fcgr3/Arg1/C1qa/Fcgr2b/Il1b/Coro1a | 15 |
| Cd14+ Monocytes | GO:0030593 | neutrophil chemotaxis | 10/49 | 102/29008 | 1.35E-15 | 2.55E-13 | 1.29E-13 | Cxcl2/Cd74/Ccl4/Fcer1g/Ccl6/Ccl3/Fcgr3/Il1b/Ccl2/Ccl7 | 10 |
| Cd14+ Monocytes | GO:0002460 | adaptive immune response based on somatic recombination of immune receptors built from immunoglobulin superfamily domains | 15/49 | 480/29008 | 1.45E-15 | 2.55E-13 | 1.29E-13 | Cd74/H2-Aa/H2-Eb1/H2-Ab1/C1qb/Fcer1g/Ctsc/C1qc/H2-DMa/Fcgr3/Arg1/C1qa/Fcgr2b/Clec4n/Il1b | 15 |
| Cd14+ Monocytes | GO:0030595 | leukocyte chemotaxis | 12/49 | 226/29008 | 2.67E-15 | 4.32E-13 | 2.18E-13 | Cxcl2/Cd74/Ccl4/Fcer1g/Dusp1/Ccl6/Ccl3/Fcgr3/Il1b/Coro1a/Ccl2/Ccl7 | 12 |
| Cd14+ Monocytes | GO:0060326 | cell chemotaxis | 13/49 | 312/29008 | 3.73E-15 | 5.57E-13 | 2.81E-13 | Cxcl2/Cd74/Ccl4/Fcer1g/Dusp1/Ccl6/Ccl3/Fcgr3/Ccrl2/Il1b/Coro1a/Ccl2/Ccl7 | 13 |
| Cd14+ Monocytes | GO:0048002 | antigen processing and presentation of peptide antigen | 9/49 | 78/29008 | 8.59E-15 | 1.19E-12 | 6.02E-13 | Cd74/H2-Aa/H2-Eb1/H2-Ab1/Ctss/Fcer1g/H2-DMa/Fcgr3/Fcgr2b | 9 |
| Cd14+ Monocytes | GO:0019882 | antigen processing and presentation | 10/49 | 126/29008 | 1.18E-14 | 1.53E-12 | 7.74E-13 | Cd74/H2-Aa/H2-Eb1/H2-Ab1/Ctss/Fcer1g/H2-DMa/Fcgr3/Fcgr2b/Cd68 | 10 |
| Cd14+ Monocytes | GO:0071621 | granulocyte chemotaxis | 10/49 | 127/29008 | 1.28E-14 | 1.56E-12 | 7.87E-13 | Cxcl2/Cd74/Ccl4/Fcer1g/Ccl6/Ccl3/Fcgr3/Il1b/Ccl2/Ccl7 | 10 |
| Cd14+ Monocytes | GO:1990266 | neutrophil migration | 10/49 | 128/29008 | 1.39E-14 | 1.59E-12 | 8.02E-13 | Cxcl2/Cd74/Ccl4/Fcer1g/Ccl6/Ccl3/Fcgr3/Il1b/Ccl2/Ccl7 | 10 |
| Cd14+ Monocytes | GO:0034341 | response to interferon-gamma | 10/49 | 139/29008 | 3.22E-14 | 3.47E-12 | 1.75E-12 | Cd74/H2-Aa/H2-Eb1/H2-Ab1/Ccl4/Ccl6/Ccl3/Arg1/Ccl2/Ccl7 | 10 |
| Cd14+ Monocytes | GO:0001819 | positive regulation of cytokine production | 14/49 | 500/29008 | 6.64E-14 | 6.78E-12 | 3.43E-12 | Cd74/Cd14/Ccl4/Fcer1g/Tyrobp/Ccl3/Fcgr3/Cd83/Clec4n/Mmp12/Laptm5/Il1b/Cebpb/Ccl2 | 14 |
| Cd14+ Monocytes | GO:0097530 | granulocyte migration | 10/49 | 157/29008 | 1.11E-13 | 1.04E-11 | 5.28E-12 | Cxcl2/Cd74/Ccl4/Fcer1g/Ccl6/Ccl3/Fcgr3/Il1b/Ccl2/Ccl7 | 10 |
| Cd14+ Monocytes | GO:0097529 | myeloid leukocyte migration | 11/49 | 226/29008 | 1.13E-13 | 1.04E-11 | 5.28E-12 | Cxcl2/Cd74/Ccl4/Fcer1g/Dusp1/Ccl6/Ccl3/Fcgr3/Il1b/Ccl2/Ccl7 | 11 |
| Cd14+ Monocytes | GO:1903037 | regulation of leukocyte cell-cell adhesion | 12/49 | 333/29008 | 2.71E-13 | 2.39E-11 | 1.21E-11 | Cd74/H2-Aa/H2-Eb1/H2-Ab1/H2-DMa/Arg1/Cd83/Laptm5/Il1b/Coro1a/Cebpb/Ccl2 | 12 |
| Cd14+ Monocytes | GO:0050863 | regulation of T cell activation | 12/49 | 339/29008 | 3.35E-13 | 2.83E-11 | 1.43E-11 | Cd74/H2-Aa/H2-Eb1/H2-Ab1/H2-DMa/Arg1/Cd83/Laptm5/Il1b/Coro1a/Cebpb/Ccl2 | 12 |
| Cd14+ Monocytes | GO:0007159 | leukocyte cell-cell adhesion | 12/49 | 373/29008 | 1.03E-12 | 8.01E-11 | 4.04E-11 | Cd74/H2-Aa/H2-Eb1/H2-Ab1/H2-DMa/Arg1/Cd83/Laptm5/Il1b/Coro1a/Cebpb/Ccl2 | 12 |
| Cd14+ Monocytes | GO:0050900 | leukocyte migration | 12/49 | 373/29008 | 1.03E-12 | 8.01E-11 | 4.04E-11 | Cxcl2/Cd74/Ccl4/Fcer1g/Dusp1/Ccl6/Ccl3/Fcgr3/Il1b/Coro1a/Ccl2/Ccl7 | 12 |
| Cd14+ Monocytes | GO:0016064 | immunoglobulin mediated immune response | 11/49 | 284/29008 | 1.37E-12 | 1.02E-10 | 5.16E-11 | Cd74/H2-Aa/H2-Eb1/H2-Ab1/C1qb/Fcer1g/C1qc/H2-DMa/Fcgr3/C1qa/Fcgr2b | 11 |
| Cd14+ Monocytes | GO:0019724 | B cell mediated immunity | 11/49 | 287/29008 | 1.53E-12 | 1.10E-10 | 5.57E-11 | Cd74/H2-Aa/H2-Eb1/H2-Ab1/C1qb/Fcer1g/C1qc/H2-DMa/Fcgr3/C1qa/Fcgr2b | 11 |
| Cd14+ Monocytes | GO:0019221 | cytokine-mediated signaling pathway | 12/49 | 397/29008 | 2.14E-12 | 1.48E-10 | 7.46E-11 | Cxcl2/Cd74/Ccl4/Fcer1g/Ccl6/Ccl3/Arg1/Mmp12/Laptm5/Il1b/Ccl2/Ccl7 | 12 |
| Cd14+ Monocytes | GO:1903706 | regulation of hemopoiesis | 12/49 | 398/29008 | 2.20E-12 | 1.48E-10 | 7.46E-11 | Cd74/H2-Aa/Zfp36/Tyrobp/C1qc/Ccl3/H2-DMa/Cd83/Id2/Mafb/Fos/Cebpb | 12 |
| Cd14+ Monocytes | GO:0050866 | negative regulation of cell activation | 10/49 | 223/29008 | 3.72E-12 | 2.41E-10 | 1.22E-10 | Cd74/H2-Aa/H2-Ab1/Tyrobp/Apoe/Arg1/Fcgr2b/Id2/Laptm5/Cebpb | 10 |
| Cd14+ Monocytes | GO:1902105 | regulation of leukocyte differentiation | 11/49 | 320/29008 | 4.98E-12 | 3.12E-10 | 1.57E-10 | Cd74/H2-Aa/Tyrobp/C1qc/Ccl3/H2-DMa/Cd83/Id2/Mafb/Fos/Cebpb | 11 |
| Cd14+ Monocytes | GO:0002573 | myeloid leukocyte differentiation | 10/49 | 238/29008 | 7.10E-12 | 4.31E-10 | 2.18E-10 | Cd74/Fcer1g/Tyrobp/C1qc/Ccl3/Id2/Mafb/Fos/Cebpb/Junb | 10 |
| Cd14+ Monocytes | GO:0050670 | regulation of lymphocyte proliferation | 10/49 | 241/29008 | 8.04E-12 | 4.73E-10 | 2.39E-10 | Cd74/H2-Aa/H2-Ab1/Tyrobp/Arg1/Fcgr2b/Laptm5/Il1b/Coro1a/Cebpb | 10 |
| Cd14+ Monocytes | GO:0032944 | regulation of mononuclear cell proliferation | 10/49 | 245/29008 | 9.46E-12 | 5.40E-10 | 2.73E-10 | Cd74/H2-Aa/H2-Ab1/Tyrobp/Arg1/Fcgr2b/Laptm5/Il1b/Coro1a/Cebpb | 10 |
| Cd14+ Monocytes | GO:0051250 | negative regulation of lymphocyte activation | 9/49 | 168/29008 | 9.95E-12 | 5.49E-10 | 2.77E-10 | Cd74/H2-Aa/H2-Ab1/Tyrobp/Arg1/Fcgr2b/Id2/Laptm5/Cebpb | 9 |
| Cd14+ Monocytes | GO:0050727 | regulation of inflammatory response | 11/49 | 342/29008 | 1.02E-11 | 5.49E-10 | 2.77E-10 | Zfp36/Ctss/Fcer1g/Apoe/Ctsc/Ccl3/Fcgr3/Fcgr2b/Alox5ap/Il1b/Cebpb | 11 |
| Cd14+ Monocytes | GO:0022407 | regulation of cell-cell adhesion | 12/49 | 457/29008 | 1.10E-11 | 5.79E-10 | 2.92E-10 | Cd74/H2-Aa/H2-Eb1/H2-Ab1/H2-DMa/Arg1/Cd83/Laptm5/Il1b/Coro1a/Cebpb/Ccl2 | 12 |
| Cd14+ Monocytes | GO:0070663 | regulation of leukocyte proliferation | 10/49 | 261/29008 | 1.77E-11 | 9.03E-10 | 4.56E-10 | Cd74/H2-Aa/H2-Ab1/Tyrobp/Arg1/Fcgr2b/Laptm5/Il1b/Coro1a/Cebpb | 10 |
| Cd14+ Monocytes | GO:1990868 | response to chemokine | 7/49 | 71/29008 | 3.07E-11 | 1.48E-09 | 7.47E-10 | Cxcl2/Ccl4/Dusp1/Ccl6/Ccl3/Ccl2/Ccl7 | 7 |
| Cd14+ Monocytes | GO:1990869 | cellular response to chemokine | 7/49 | 71/29008 | 3.07E-11 | 1.48E-09 | 7.47E-10 | Cxcl2/Ccl4/Dusp1/Ccl6/Ccl3/Ccl2/Ccl7 | 7 |
| Cd14+ Monocytes | GO:0002696 | positive regulation of leukocyte activation | 12/49 | 500/29008 | 3.12E-11 | 1.48E-09 | 7.47E-10 | Cd74/H2-Aa/H2-Eb1/H2-Ab1/Fcer1g/Tyrobp/Ctsc/H2-DMa/Cd83/Il1b/Coro1a/Ccl2 | 12 |
| Cd14+ Monocytes | GO:0002695 | negative regulation of leukocyte activation | 9/49 | 200/29008 | 4.76E-11 | 2.20E-09 | 1.11E-09 | Cd74/H2-Aa/H2-Ab1/Tyrobp/Arg1/Fcgr2b/Id2/Laptm5/Cebpb | 9 |
| Cd14+ Monocytes | GO:0050729 | positive regulation of inflammatory response | 8/49 | 134/29008 | 6.46E-11 | 2.92E-09 | 1.47E-09 | Ctss/Fcer1g/Ctsc/Ccl3/Fcgr3/Alox5ap/Il1b/Cebpb | 8 |
| Cd14+ Monocytes | GO:0071347 | cellular response to interleukin-1 | 7/49 | 80/29008 | 7.26E-11 | 3.20E-09 | 1.62E-09 | Ccl4/Ccl6/Ccl3/Il1b/Cebpb/Ccl2/Ccl7 | 7 |
| Cd14+ Monocytes | GO:0002761 | regulation of myeloid leukocyte differentiation | 8/49 | 137/29008 | 7.71E-11 | 3.33E-09 | 1.68E-09 | Cd74/Tyrobp/C1qc/Ccl3/Id2/Mafb/Fos/Cebpb | 8 |
| Cd14+ Monocytes | GO:0050870 | positive regulation of T cell activation | 9/49 | 216/29008 | 9.45E-11 | 3.99E-09 | 2.02E-09 | Cd74/H2-Aa/H2-Eb1/H2-Ab1/H2-DMa/Cd83/Il1b/Coro1a/Ccl2 | 9 |
| Cd14+ Monocytes | GO:0030099 | myeloid cell differentiation | 11/49 | 426/29008 | 1.06E-10 | 4.32E-09 | 2.18E-09 | Cd74/Zfp36/Fcer1g/Tyrobp/C1qc/Ccl3/Id2/Mafb/Fos/Cebpb/Junb | 11 |
| Cd14+ Monocytes | GO:0045637 | regulation of myeloid cell differentiation | 9/49 | 219/29008 | 1.07E-10 | 4.32E-09 | 2.18E-09 | Cd74/Zfp36/Tyrobp/C1qc/Ccl3/Id2/Mafb/Fos/Cebpb | 9 |
| Cd14+ Monocytes | GO:0070997 | neuron death | 11/49 | 428/29008 | 1.12E-10 | 4.43E-09 | 2.24E-09 | Cxcl2/Tyrobp/Apoe/Ccl3/C1qa/Fcgr2b/Il1b/Coro1a/Fos/Cebpb/Ccl2 | 11 |
| Cd14+ Monocytes | GO:0070372 | regulation of ERK1 and ERK2 cascade | 10/49 | 326/29008 | 1.56E-10 | 6.06E-09 | 3.06E-09 | Cd74/Ccl4/Atf3/Apoe/Dusp1/Ccl6/Ccl3/Il1b/Ccl2/Ccl7 | 10 |
| Macrophages(M1 like) | GO:0002495 | antigen processing and presentation of peptide antigen via MHC class II | 11/48 | 24/29008 | 1.82E-25 | 3.12E-22 | 1.48E-22 | Cd74/H2-Ab1/H2-Eb1/H2-Aa/Ctss/Fcer1g/Fcgr2b/Trem2/H2-DMa/Ifi30/H2-DMb1 | 11 |
| Macrophages(M1 like) | GO:0002504 | antigen processing and presentation of peptide or polysaccharide antigen via MHC class II | 11/48 | 26/29008 | 5.61E-25 | 4.82E-22 | 2.29E-22 | Cd74/H2-Ab1/H2-Eb1/H2-Aa/Ctss/Fcer1g/Fcgr2b/Trem2/H2-DMa/Ifi30/H2-DMb1 | 11 |
| Macrophages(M1 like) | GO:0019886 | antigen processing and presentation of exogenous peptide antigen via MHC class II | 10/48 | 20/29008 | 1.03E-23 | 5.90E-21 | 2.80E-21 | Cd74/H2-Ab1/H2-Eb1/H2-Aa/Ctss/Fcer1g/Fcgr2b/H2-DMa/Ifi30/H2-DMb1 | 10 |
| Macrophages(M1 like) | GO:0002478 | antigen processing and presentation of exogenous peptide antigen | 11/48 | 33/29008 | 1.39E-23 | 5.99E-21 | 2.84E-21 | Cd74/H2-Ab1/H2-Eb1/H2-Aa/Ctss/Fcer1g/Fcgr3/Fcgr2b/H2-DMa/Ifi30/H2-DMb1 | 11 |
| Macrophages(M1 like) | GO:0019884 | antigen processing and presentation of exogenous antigen | 11/48 | 40/29008 | 1.65E-22 | 5.68E-20 | 2.69E-20 | Cd74/H2-Ab1/H2-Eb1/H2-Aa/Ctss/Fcer1g/Fcgr3/Fcgr2b/H2-DMa/Ifi30/H2-DMb1 | 11 |
| Macrophages(M1 like) | GO:0048002 | antigen processing and presentation of peptide antigen | 12/48 | 78/29008 | 3.79E-21 | 1.09E-18 | 5.16E-19 | Cd74/H2-Ab1/H2-Eb1/H2-Aa/Ctss/Fcer1g/Fcgr3/Fcgr2b/Trem2/H2-DMa/Ifi30/H2-DMb1 | 12 |
| Macrophages(M1 like) | GO:0019882 | antigen processing and presentation | 13/48 | 126/29008 | 1.76E-20 | 4.33E-18 | 2.05E-18 | Cd74/H2-Ab1/H2-Eb1/H2-Aa/Ctss/Fcer1g/Fcgr3/Fcgr2b/Cd68/Trem2/H2-DMa/Ifi30/H2-DMb1 | 13 |
| Macrophages(M1 like) | GO:0032103 | positive regulation of response to external stimulus | 17/48 | 427/29008 | 1.46E-19 | 3.13E-17 | 1.48E-17 | Cd74/Ctss/Fcer1g/Tyrobp/Fcgr3/Ctsc/Alox5ap/Ly86/Arg1/Ccr2/Spi1/Aif1/Clec4n/Trem2/Csf1r/Cyba/Lgmn | 17 |
| Macrophages(M1 like) | GO:0002449 | lymphocyte mediated immunity | 17/48 | 477/29008 | 9.40E-19 | 1.80E-16 | 8.52E-17 | Cd74/H2-Ab1/H2-Eb1/H2-Aa/C1qb/C1qc/Fcer1g/C1qa/Fcgr3/Ctsc/Fcgr2b/Arg1/Coro1a/Ccr2/Trem2/H2-DMa/H2-DMb1 | 17 |
| Macrophages(M1 like) | GO:0002460 | adaptive immune response based on somatic recombination of immune receptors built from immunoglobulin superfamily domains | 17/48 | 480/29008 | 1.04E-18 | 1.80E-16 | 8.52E-17 | Cd74/H2-Ab1/H2-Eb1/H2-Aa/C1qb/C1qc/Fcer1g/C1qa/Fcgr3/Ctsc/Fcgr2b/Arg1/Ccr2/Clec4n/Trem2/H2-DMa/H2-DMb1 | 17 |
| Macrophages(M1 like) | GO:0031349 | positive regulation of defense response | 13/48 | 273/29008 | 4.91E-16 | 7.68E-14 | 3.64E-14 | Cd74/Ctss/Fcer1g/Tyrobp/Fcgr3/Ctsc/Alox5ap/Arg1/Ccr2/Spi1/Clec4n/Trem2/Cyba | 13 |
| Macrophages(M1 like) | GO:0016064 | immunoglobulin mediated immune response | 13/48 | 284/29008 | 8.19E-16 | 1.17E-13 | 5.57E-14 | Cd74/H2-Ab1/H2-Eb1/H2-Aa/C1qb/C1qc/Fcer1g/C1qa/Fcgr3/Fcgr2b/Trem2/H2-DMa/H2-DMb1 | 13 |
| Macrophages(M1 like) | GO:0019724 | B cell mediated immunity | 13/48 | 287/29008 | 9.39E-16 | 1.24E-13 | 5.89E-14 | Cd74/H2-Ab1/H2-Eb1/H2-Aa/C1qb/C1qc/Fcer1g/C1qa/Fcgr3/Fcgr2b/Trem2/H2-DMa/H2-DMb1 | 13 |
| Macrophages(M1 like) | GO:0030595 | leukocyte chemotaxis | 12/48 | 226/29008 | 2.03E-15 | 2.49E-13 | 1.18E-13 | Cd74/Fcer1g/Fcgr3/Ccl6/Coro1a/Ccr2/Spi1/Aif1/Csf1r/Cxcl16/Pf4/Lgmn | 12 |
| Macrophages(M1 like) | GO:0032944 | regulation of mononuclear cell proliferation | 12/48 | 245/29008 | 5.35E-15 | 6.14E-13 | 2.91E-13 | Cd74/H2-Ab1/H2-Aa/Tyrobp/Fcgr2b/Arg1/Laptm5/Coro1a/Ccr2/Aif1/Csf1r/H2-DMb1 | 12 |
| Macrophages(M1 like) | GO:0070663 | regulation of leukocyte proliferation | 12/48 | 261/29008 | 1.14E-14 | 1.23E-12 | 5.82E-13 | Cd74/H2-Ab1/H2-Aa/Tyrobp/Fcgr2b/Arg1/Laptm5/Coro1a/Ccr2/Aif1/Csf1r/H2-DMb1 | 12 |
| Macrophages(M1 like) | GO:0070661 | leukocyte proliferation | 13/48 | 358/29008 | 1.62E-14 | 1.64E-12 | 7.78E-13 | Cd74/H2-Ab1/H2-Aa/Tyrobp/Fcgr2b/Arg1/Laptm5/Coro1a/Ccr2/Aif1/Trem2/Csf1r/H2-DMb1 | 13 |
| Macrophages(M1 like) | GO:0050900 | leukocyte migration | 13/48 | 373/29008 | 2.74E-14 | 2.62E-12 | 1.24E-12 | Cd74/Fcer1g/Fcgr3/Ccl6/Coro1a/Ccr2/Spi1/Aif1/Trem2/Csf1r/Cxcl16/Pf4/Lgmn | 13 |
| Macrophages(M1 like) | GO:0050866 | negative regulation of cell activation | 11/48 | 223/29008 | 7.61E-14 | 6.90E-12 | 3.27E-12 | Cd74/H2-Ab1/Apoe/H2-Aa/Tyrobp/Fcgr2b/Arg1/Laptm5/Ccr2/Spi1/Trem2 | 11 |
| Macrophages(M1 like) | GO:0097529 | myeloid leukocyte migration | 11/48 | 226/29008 | 8.82E-14 | 7.59E-12 | 3.60E-12 | Cd74/Fcer1g/Fcgr3/Ccl6/Ccr2/Spi1/Aif1/Trem2/Csf1r/Pf4/Lgmn | 11 |
| Macrophages(M1 like) | GO:0060326 | cell chemotaxis | 12/48 | 312/29008 | 9.56E-14 | 7.84E-12 | 3.72E-12 | Cd74/Fcer1g/Fcgr3/Ccl6/Coro1a/Ccr2/Spi1/Aif1/Csf1r/Cxcl16/Pf4/Lgmn | 12 |
| Macrophages(M1 like) | GO:0050670 | regulation of lymphocyte proliferation | 11/48 | 241/29008 | 1.78E-13 | 1.40E-11 | 6.62E-12 | Cd74/H2-Ab1/H2-Aa/Tyrobp/Fcgr2b/Arg1/Laptm5/Coro1a/Ccr2/Aif1/H2-DMb1 | 11 |
| Macrophages(M1 like) | GO:0032943 | mononuclear cell proliferation | 12/48 | 334/29008 | 2.14E-13 | 1.60E-11 | 7.60E-12 | Cd74/H2-Ab1/H2-Aa/Tyrobp/Fcgr2b/Arg1/Laptm5/Coro1a/Ccr2/Aif1/Csf1r/H2-DMb1 | 12 |
| Macrophages(M1 like) | GO:0002683 | negative regulation of immune system process | 13/48 | 460/29008 | 3.95E-13 | 2.83E-11 | 1.34E-11 | Cd74/H2-Ab1/H2-Aa/C1qc/Fcer1g/Tyrobp/Fcgr2b/Cd68/Arg1/Laptm5/Ccr2/Spi1/Trem2 | 13 |
| Macrophages(M1 like) | GO:0002696 | positive regulation of leukocyte activation | 13/48 | 500/29008 | 1.13E-12 | 7.78E-11 | 3.69E-11 | Cd74/H2-Ab1/H2-Eb1/H2-Aa/Fcer1g/Tyrobp/Ctsc/Coro1a/Ccr2/Aif1/Trem2/H2-DMa/H2-DMb1 | 13 |
| Macrophages(M1 like) | GO:0002399 | MHC class II protein complex assembly | 5/48 | 11/29008 | 4.59E-12 | 2.93E-10 | 1.39E-10 | H2-Ab1/H2-Eb1/H2-Aa/H2-DMa/H2-DMb1 | 5 |
| Macrophages(M1 like) | GO:0002503 | peptide antigen assembly with MHC class II protein complex | 5/48 | 11/29008 | 4.59E-12 | 2.93E-10 | 1.39E-10 | H2-Ab1/H2-Eb1/H2-Aa/H2-DMa/H2-DMb1 | 5 |
| Macrophages(M1 like) | GO:0002819 | regulation of adaptive immune response | 10/48 | 236/29008 | 5.24E-12 | 3.22E-10 | 1.53E-10 | Cd74/H2-Ab1/Fcer1g/Fcgr3/Fcgr2b/Arg1/Ccr2/Clec4n/Trem2/H2-DMa | 10 |
| Macrophages(M1 like) | GO:0046651 | lymphocyte proliferation | 11/48 | 330/29008 | 5.43E-12 | 3.22E-10 | 1.53E-10 | Cd74/H2-Ab1/H2-Aa/Tyrobp/Fcgr2b/Arg1/Laptm5/Coro1a/Ccr2/Aif1/H2-DMb1 | 11 |
| Macrophages(M1 like) | GO:1903037 | regulation of leukocyte cell-cell adhesion | 11/48 | 333/29008 | 5.99E-12 | 3.43E-10 | 1.63E-10 | Cd74/H2-Ab1/H2-Eb1/H2-Aa/Arg1/Laptm5/Coro1a/Ccr2/Aif1/H2-DMa/H2-DMb1 | 11 |
| Macrophages(M1 like) | GO:0050863 | regulation of T cell activation | 11/48 | 339/29008 | 7.26E-12 | 4.03E-10 | 1.91E-10 | Cd74/H2-Ab1/H2-Eb1/H2-Aa/Arg1/Laptm5/Coro1a/Ccr2/Aif1/H2-DMa/H2-DMb1 | 11 |
| Macrophages(M1 like) | GO:0022407 | regulation of cell-cell adhesion | 12/48 | 457/29008 | 8.45E-12 | 4.54E-10 | 2.15E-10 | Cd74/H2-Ab1/H2-Eb1/H2-Aa/Arg1/Laptm5/Coro1a/Ccr2/Spi1/Aif1/H2-DMa/H2-DMb1 | 12 |
| Macrophages(M1 like) | GO:0002501 | peptide antigen assembly with MHC protein complex | 5/48 | 13/29008 | 1.28E-11 | 6.65E-10 | 3.15E-10 | H2-Ab1/H2-Eb1/H2-Aa/H2-DMa/H2-DMb1 | 5 |
| Macrophages(M1 like) | GO:0002396 | MHC protein complex assembly | 5/48 | 14/29008 | 1.98E-11 | 9.96E-10 | 4.72E-10 | H2-Ab1/H2-Eb1/H2-Aa/H2-DMa/H2-DMb1 | 5 |
| Macrophages(M1 like) | GO:0007159 | leukocyte cell-cell adhesion | 11/48 | 373/29008 | 2.03E-11 | 9.96E-10 | 4.72E-10 | Cd74/H2-Ab1/H2-Eb1/H2-Aa/Arg1/Laptm5/Coro1a/Ccr2/Aif1/H2-DMa/H2-DMb1 | 11 |
| Macrophages(M1 like) | GO:0002440 | production of molecular mediator of immune response | 11/48 | 380/29008 | 2.47E-11 | 1.18E-09 | 5.60E-10 | Cd74/H2-Ab1/H2-Eb1/H2-Aa/Fcer1g/Fcgr2b/Arg1/Laptm5/Ccr2/H2-DMa/H2-DMb1 | 11 |
| Macrophages(M1 like) | GO:0002695 | negative regulation of leukocyte activation | 9/48 | 200/29008 | 3.91E-11 | 1.79E-09 | 8.47E-10 | Cd74/H2-Ab1/H2-Aa/Tyrobp/Fcgr2b/Arg1/Laptm5/Ccr2/Spi1 | 9 |
| Macrophages(M1 like) | GO:0019221 | cytokine-mediated signaling pathway | 11/48 | 397/29008 | 3.95E-11 | 1.79E-09 | 8.47E-10 | Cd74/Fcer1g/Ccl6/Arg1/Laptm5/Ccr2/Spi1/Trem2/Csf2ra/Csf1r/Pf4 | 11 |
| Macrophages(M1 like) | GO:0002699 | positive regulation of immune effector process | 10/48 | 296/29008 | 4.88E-11 | 2.16E-09 | 1.02E-09 | Cd74/Fcer1g/Tyrobp/Fcgr3/Arg1/Laptm5/Ccr2/Spi1/Trem2/H2-DMb1 | 10 |
| Macrophages(M1 like) | GO:0050870 | positive regulation of T cell activation | 9/48 | 216/29008 | 7.76E-11 | 3.34E-09 | 1.58E-09 | Cd74/H2-Ab1/H2-Eb1/H2-Aa/Coro1a/Ccr2/Aif1/H2-DMa/H2-DMb1 | 9 |
| Macrophages(M1 like) | GO:0002697 | regulation of immune effector process | 11/48 | 424/29008 | 7.95E-11 | 3.34E-09 | 1.58E-09 | Cd74/Fcer1g/Tyrobp/Fcgr3/Fcgr2b/Arg1/Laptm5/Ccr2/Spi1/Trem2/H2-DMb1 | 11 |
| Macrophages(M1 like) | GO:0002822 | regulation of adaptive immune response based on somatic recombination of immune receptors built from immunoglobulin superfamily domains | 9/48 | 220/29008 | 9.14E-11 | 3.75E-09 | 1.78E-09 | H2-Ab1/Fcer1g/Fcgr3/Fcgr2b/Arg1/Ccr2/Clec4n/Trem2/H2-DMa | 9 |
| Macrophages(M1 like) | GO:1903039 | positive regulation of leukocyte cell-cell adhesion | 9/48 | 240/29008 | 1.98E-10 | 7.92E-09 | 3.76E-09 | Cd74/H2-Ab1/H2-Eb1/H2-Aa/Coro1a/Ccr2/Aif1/H2-DMa/H2-DMb1 | 9 |
| Macrophages(M1 like) | GO:0002821 | positive regulation of adaptive immune response | 8/48 | 159/29008 | 2.14E-10 | 8.36E-09 | 3.96E-09 | Cd74/H2-Ab1/Fcer1g/Fcgr3/Ccr2/Clec4n/Trem2/H2-DMa | 8 |
| Macrophages(M1 like) | GO:0002274 | myeloid leukocyte activation | 9/48 | 261/29008 | 4.16E-10 | 1.59E-08 | 7.54E-09 | Fcer1g/Tyrobp/C1qa/Fcgr3/Ctsc/Ccr2/Spi1/Aif1/Trem2 | 9 |
| Macrophages(M1 like) | GO:0001819 | positive regulation of cytokine production | 11/48 | 500/29008 | 4.55E-10 | 1.70E-08 | 8.06E-09 | Cd74/Fcer1g/Tyrobp/Fcgr3/Laptm5/Ccr2/Aif1/Clec4n/Trem2/Csf1r/Cyba | 11 |
| Macrophages(M1 like) | GO:0006909 | phagocytosis | 10/48 | 383/29008 | 5.99E-10 | 2.20E-08 | 1.04E-08 | Fcer1g/Tyrobp/Fcgr3/Fcgr2b/Pld4/Coro1a/Ccr2/Aif1/Trem2/Cyba | 10 |
| Macrophages(M1 like) | GO:0042129 | regulation of T cell proliferation | 8/48 | 183/29008 | 6.54E-10 | 2.35E-08 | 1.11E-08 | H2-Ab1/H2-Aa/Arg1/Laptm5/Coro1a/Ccr2/Aif1/H2-DMb1 | 8 |
| Macrophages(M1 like) | GO:0022409 | positive regulation of cell-cell adhesion | 9/48 | 288/29008 | 9.89E-10 | 3.47E-08 | 1.65E-08 | Cd74/H2-Ab1/H2-Eb1/H2-Aa/Coro1a/Ccr2/Aif1/H2-DMa/H2-DMb1 | 9 |
| Macrophages(M1 like) | GO:0002888 | positive regulation of myeloid leukocyte mediated immunity | 5/48 | 29/29008 | 1.15E-09 | 3.97E-08 | 1.88E-08 | Fcer1g/Tyrobp/Fcgr3/Arg1/Spi1 | 5 |
| Macrophages(M2 like) | GO:0002683 | negative regulation of immune system process | 17/49 | 460/29008 | 7.71E-19 | 1.49E-15 | 6.83E-16 | Arg1/Mmp12/Cd68/Fcer1g/Tyrobp/C1qc/Fcgr2b/Id2/Trem2/Hmox1/Mafb/Laptm5/H2-Aa/Cd74/Cebpb/Dusp1/Ccl2 | 17 |
| Macrophages(M2 like) | GO:0097529 | myeloid leukocyte migration | 13/49 | 226/29008 | 5.64E-17 | 5.45E-14 | 2.50E-14 | Ccl6/Pf4/Fcer1g/Fcgr3/C3ar1/Ccl9/Trem2/Cxcl2/Cd74/Lgmn/Spp1/Dusp1/Ccl2 | 13 |
| Macrophages(M2 like) | GO:0032103 | positive regulation of response to external stimulus | 15/49 | 427/29008 | 2.58E-16 | 1.66E-13 | 7.62E-14 | Arg1/Mmp12/Ctss/Fcer1g/Tyrobp/Fcgr3/C3ar1/Alox5ap/Trem2/Clec4n/Ctsc/Cd74/Lgmn/Cebpb/Ccl2 | 15 |
| Macrophages(M2 like) | GO:0030593 | neutrophil chemotaxis | 10/49 | 102/29008 | 1.35E-15 | 6.51E-13 | 2.99E-13 | Ccl6/Pf4/Fcer1g/Fcgr3/C3ar1/Ccl9/Cxcl2/Cd74/Spp1/Ccl2 | 10 |
| Macrophages(M2 like) | GO:0030595 | leukocyte chemotaxis | 12/49 | 226/29008 | 2.67E-15 | 1.03E-12 | 4.73E-13 | Ccl6/Pf4/Fcer1g/Fcgr3/C3ar1/Ccl9/Cxcl2/Cd74/Lgmn/Spp1/Dusp1/Ccl2 | 12 |
| Macrophages(M2 like) | GO:0071621 | granulocyte chemotaxis | 10/49 | 127/29008 | 1.28E-14 | 3.84E-12 | 1.76E-12 | Ccl6/Pf4/Fcer1g/Fcgr3/C3ar1/Ccl9/Cxcl2/Cd74/Spp1/Ccl2 | 10 |
| Macrophages(M2 like) | GO:1990266 | neutrophil migration | 10/49 | 128/29008 | 1.39E-14 | 3.84E-12 | 1.76E-12 | Ccl6/Pf4/Fcer1g/Fcgr3/C3ar1/Ccl9/Cxcl2/Cd74/Spp1/Ccl2 | 10 |
| Macrophages(M2 like) | GO:0031349 | positive regulation of defense response | 12/49 | 273/29008 | 2.57E-14 | 6.20E-12 | 2.84E-12 | Arg1/Mmp12/Ctss/Fcer1g/Tyrobp/Fcgr3/Alox5ap/Trem2/Clec4n/Ctsc/Cd74/Cebpb | 12 |
| Macrophages(M2 like) | GO:0050900 | leukocyte migration | 13/49 | 373/29008 | 3.69E-14 | 7.92E-12 | 3.63E-12 | Ccl6/Pf4/Fcer1g/Fcgr3/C3ar1/Ccl9/Trem2/Cxcl2/Cd74/Lgmn/Spp1/Dusp1/Ccl2 | 13 |
| Macrophages(M2 like) | GO:0001819 | positive regulation of cytokine production | 14/49 | 500/29008 | 6.64E-14 | 1.28E-11 | 5.88E-12 | Mmp12/Cd14/Fcer1g/Tyrobp/Fcgr3/C3ar1/Trem2/Clec4n/Laptm5/Cd74/Hilpda/Cebpb/Rgcc/Ccl2 | 14 |
| Macrophages(M2 like) | GO:0050866 | negative regulation of cell activation | 11/49 | 223/29008 | 9.75E-14 | 1.71E-11 | 7.86E-12 | Arg1/Tyrobp/Fcgr2b/Id2/Trem2/Hmox1/Laptm5/Apoe/H2-Aa/Cd74/Cebpb | 11 |
| Macrophages(M2 like) | GO:0097530 | granulocyte migration | 10/49 | 157/29008 | 1.11E-13 | 1.78E-11 | 8.17E-12 | Ccl6/Pf4/Fcer1g/Fcgr3/C3ar1/Ccl9/Cxcl2/Cd74/Spp1/Ccl2 | 10 |
| Macrophages(M2 like) | GO:0060326 | cell chemotaxis | 12/49 | 312/29008 | 1.25E-13 | 1.86E-11 | 8.55E-12 | Ccl6/Pf4/Fcer1g/Fcgr3/C3ar1/Ccl9/Cxcl2/Cd74/Lgmn/Spp1/Dusp1/Ccl2 | 12 |
| Macrophages(M2 like) | GO:0002460 | adaptive immune response based on somatic recombination of immune receptors built from immunoglobulin superfamily domains | 13/49 | 480/29008 | 9.06E-13 | 1.25E-10 | 5.74E-11 | Arg1/Fcer1g/C1qc/C1qb/Fcgr3/C1qa/C3ar1/Fcgr2b/Trem2/Clec4n/Ctsc/H2-Aa/Cd74 | 13 |
| Macrophages(M2 like) | GO:0002495 | antigen processing and presentation of peptide antigen via MHC class II | 6/49 | 24/29008 | 2.22E-12 | 2.86E-10 | 1.31E-10 | Ctss/Fcer1g/Fcgr2b/Trem2/H2-Aa/Cd74 | 6 |
| Macrophages(M2 like) | GO:0002504 | antigen processing and presentation of peptide or polysaccharide antigen via MHC class II | 6/49 | 26/29008 | 3.79E-12 | 4.58E-10 | 2.10E-10 | Ctss/Fcer1g/Fcgr2b/Trem2/H2-Aa/Cd74 | 6 |
| Macrophages(M2 like) | GO:0002478 | antigen processing and presentation of exogenous peptide antigen | 6/49 | 33/29008 | 1.81E-11 | 2.06E-09 | 9.43E-10 | Ctss/Fcer1g/Fcgr3/Fcgr2b/H2-Aa/Cd74 | 6 |
| Macrophages(M2 like) | GO:1990868 | response to chemokine | 7/49 | 71/29008 | 3.07E-11 | 3.13E-09 | 1.43E-09 | Ccl6/Pf4/Ccl9/Trem2/Cxcl2/Dusp1/Ccl2 | 7 |
| Macrophages(M2 like) | GO:1990869 | cellular response to chemokine | 7/49 | 71/29008 | 3.07E-11 | 3.13E-09 | 1.43E-09 | Ccl6/Pf4/Ccl9/Trem2/Cxcl2/Dusp1/Ccl2 | 7 |
| Macrophages(M2 like) | GO:0019882 | antigen processing and presentation | 8/49 | 126/29008 | 3.93E-11 | 3.80E-09 | 1.74E-09 | Ctss/Cd68/Fcer1g/Fcgr3/Fcgr2b/Trem2/H2-Aa/Cd74 | 8 |
| Macrophages(M2 like) | GO:0002695 | negative regulation of leukocyte activation | 9/49 | 200/29008 | 4.76E-11 | 4.38E-09 | 2.01E-09 | Arg1/Tyrobp/Fcgr2b/Id2/Hmox1/Laptm5/H2-Aa/Cd74/Cebpb | 9 |
| Macrophages(M2 like) | GO:0019221 | cytokine-mediated signaling pathway | 11/49 | 397/29008 | 5.03E-11 | 4.41E-09 | 2.02E-09 | Arg1/Ccl6/Mmp12/Pf4/Fcer1g/Ccl9/Trem2/Laptm5/Cxcl2/Cd74/Ccl2 | 11 |
| Macrophages(M2 like) | GO:0048002 | antigen processing and presentation of peptide antigen | 7/49 | 78/29008 | 6.05E-11 | 5.00E-09 | 2.30E-09 | Ctss/Fcer1g/Fcgr3/Fcgr2b/Trem2/H2-Aa/Cd74 | 7 |
| Macrophages(M2 like) | GO:0019884 | antigen processing and presentation of exogenous antigen | 6/49 | 40/29008 | 6.22E-11 | 5.00E-09 | 2.30E-09 | Ctss/Fcer1g/Fcgr3/Fcgr2b/H2-Aa/Cd74 | 6 |
| Macrophages(M2 like) | GO:0019886 | antigen processing and presentation of exogenous peptide antigen via MHC class II | 5/49 | 20/29008 | 1.70E-10 | 1.31E-08 | 6.01E-09 | Ctss/Fcer1g/Fcgr2b/H2-Aa/Cd74 | 5 |
| Macrophages(M2 like) | GO:0002449 | lymphocyte mediated immunity | 11/49 | 477/29008 | 3.52E-10 | 2.61E-08 | 1.20E-08 | Arg1/Fcer1g/C1qc/C1qb/Fcgr3/C1qa/Fcgr2b/Trem2/Ctsc/H2-Aa/Cd74 | 11 |
| Macrophages(M2 like) | GO:0051250 | negative regulation of lymphocyte activation | 8/49 | 168/29008 | 3.94E-10 | 2.82E-08 | 1.29E-08 | Arg1/Tyrobp/Fcgr2b/Id2/Laptm5/H2-Aa/Cd74/Cebpb | 8 |
| Macrophages(M2 like) | GO:0070098 | chemokine-mediated signaling pathway | 6/49 | 61/29008 | 8.75E-10 | 6.04E-08 | 2.77E-08 | Ccl6/Pf4/Ccl9/Trem2/Cxcl2/Ccl2 | 6 |
| Macrophages(M2 like) | GO:0016064 | immunoglobulin mediated immune response | 9/49 | 284/29008 | 1.06E-09 | 7.07E-08 | 3.25E-08 | Fcer1g/C1qc/C1qb/Fcgr3/C1qa/Fcgr2b/Trem2/H2-Aa/Cd74 | 9 |
| Macrophages(M2 like) | GO:0019724 | B cell mediated immunity | 9/49 | 287/29008 | 1.16E-09 | 7.50E-08 | 3.44E-08 | Fcer1g/C1qc/C1qb/Fcgr3/C1qa/Fcgr2b/Trem2/H2-Aa/Cd74 | 9 |
| Macrophages(M2 like) | GO:0098883 | synapse pruning | 4/49 | 10/29008 | 1.50E-09 | 9.21E-08 | 4.23E-08 | C1qc/C1qb/C1qa/Trem2 | 4 |
| Macrophages(M2 like) | GO:0002699 | positive regulation of immune effector process | 9/49 | 296/29008 | 1.53E-09 | 9.21E-08 | 4.23E-08 | Arg1/Fcer1g/Tyrobp/Fcgr3/Trem2/Hmox1/Laptm5/Cd74/Ccl2 | 9 |
| Macrophages(M2 like) | GO:0002697 | regulation of immune effector process | 10/49 | 424/29008 | 1.98E-09 | 1.16E-07 | 5.32E-08 | Arg1/Fcer1g/Tyrobp/Fcgr3/Fcgr2b/Trem2/Hmox1/Laptm5/Cd74/Ccl2 | 10 |
| Macrophages(M2 like) | GO:0070997 | neuron death | 10/49 | 428/29008 | 2.17E-09 | 1.23E-07 | 5.65E-08 | Tyrobp/C1qa/Fcgr2b/Trem2/Hmox1/Cxcl2/Apoe/Lgmn/Cebpb/Ccl2 | 10 |
| Macrophages(M2 like) | GO:0002253 | activation of immune response | 10/49 | 439/29008 | 2.76E-09 | 1.50E-07 | 6.90E-08 | Fcer1g/Tyrobp/C1qc/C1qb/C1qa/C3ar1/Trem2/Clec4n/Laptm5/Rgcc | 10 |
| Macrophages(M2 like) | GO:0050729 | positive regulation of inflammatory response | 7/49 | 134/29008 | 2.80E-09 | 1.50E-07 | 6.90E-08 | Ctss/Fcer1g/Fcgr3/Alox5ap/Trem2/Ctsc/Cebpb | 7 |
| Macrophages(M2 like) | GO:0002761 | regulation of myeloid leukocyte differentiation | 7/49 | 137/29008 | 3.27E-09 | 1.71E-07 | 7.83E-08 | Tyrobp/C1qc/Id2/Trem2/Mafb/Cd74/Cebpb | 7 |
| Macrophages(M2 like) | GO:0050727 | regulation of inflammatory response | 9/49 | 342/29008 | 5.38E-09 | 2.73E-07 | 1.25E-07 | Ctss/Fcer1g/Fcgr3/Fcgr2b/Alox5ap/Trem2/Ctsc/Apoe/Cebpb | 9 |
| Macrophages(M2 like) | GO:0002573 | myeloid leukocyte differentiation | 8/49 | 238/29008 | 6.16E-09 | 3.05E-07 | 1.40E-07 | Fcer1g/Tyrobp/C1qc/Id2/Trem2/Mafb/Cd74/Cebpb | 8 |
| Macrophages(M2 like) | GO:0050672 | negative regulation of lymphocyte proliferation | 6/49 | 92/29008 | 1.08E-08 | 5.22E-07 | 2.39E-07 | Arg1/Tyrobp/Fcgr2b/Laptm5/H2-Aa/Cebpb | 6 |
| Macrophages(M2 like) | GO:0032945 | negative regulation of mononuclear cell proliferation | 6/49 | 93/29008 | 1.15E-08 | 5.44E-07 | 2.49E-07 | Arg1/Tyrobp/Fcgr2b/Laptm5/H2-Aa/Cebpb | 6 |
| Macrophages(M2 like) | GO:0002274 | myeloid leukocyte activation | 8/49 | 261/29008 | 1.26E-08 | 5.82E-07 | 2.67E-07 | Fcer1g/Tyrobp/Clec4d/Fcgr3/C1qa/Trem2/Ctsc/Hmox1 | 8 |
| Macrophages(M2 like) | GO:1901214 | regulation of neuron death | 9/49 | 384/29008 | 1.46E-08 | 6.58E-07 | 3.02E-07 | Tyrobp/C1qa/Fcgr2b/Trem2/Hmox1/Apoe/Lgmn/Cebpb/Ccl2 | 9 |
| Macrophages(M2 like) | GO:0070664 | negative regulation of leukocyte proliferation | 6/49 | 98/29008 | 1.58E-08 | 6.95E-07 | 3.19E-07 | Arg1/Tyrobp/Fcgr2b/Laptm5/H2-Aa/Cebpb | 6 |
| Macrophages(M2 like) | GO:0006959 | humoral immune response | 9/49 | 416/29008 | 2.91E-08 | 1.25E-06 | 5.74E-07 | Pf4/Wfdc17/C1qc/C1qb/C1qa/Fcgr2b/Trem2/Cxcl2/Rgcc | 9 |
| Macrophages(M2 like) | GO:0001818 | negative regulation of cytokine production | 8/49 | 292/29008 | 3.02E-08 | 1.27E-06 | 5.82E-07 | Arg1/Srgn/Tyrobp/Fcgr2b/Trem2/Hmox1/Laptm5/Rgcc | 8 |
| Macrophages(M2 like) | GO:0002703 | regulation of leukocyte mediated immunity | 8/49 | 297/29008 | 3.44E-08 | 1.42E-06 | 6.49E-07 | Arg1/Fcer1g/Tyrobp/Fcgr3/Fcgr2b/Trem2/Hmox1/Ccl2 | 8 |
| Macrophages(M2 like) | GO:0150146 | cell junction disassembly | 4/49 | 21/29008 | 4.21E-08 | 1.69E-06 | 7.77E-07 | C1qc/C1qb/C1qa/Trem2 | 4 |
| Macrophages(M2 like) | GO:0007162 | negative regulation of cell adhesion | 8/49 | 310/29008 | 4.79E-08 | 1.89E-06 | 8.67E-07 | Arg1/Mmp12/Laptm5/H2-Aa/Cd74/Cebpb/Dusp1/Rgcc | 8 |
| Macrophages(M2 like) | GO:0032963 | collagen metabolic process | 6/49 | 119/29008 | 5.08E-08 | 1.94E-06 | 8.88E-07 | Arg1/Mmp12/Ctss/Ctsb/Rgcc/Ccl2 | 6 |
| DCs | GO:0002478 | antigen processing and presentation of exogenous peptide antigen | 10/48 | 33/29008 | 5.07E-21 | 7.83E-18 | 4.17E-18 | Fcgr1/Fcgr3/Fcer1g/Fcgr2b/Ctss/H2-DMa/H2-Eb1/H2-Ab1/H2-Aa/Cd74 | 10 |
| DCs | GO:0019884 | antigen processing and presentation of exogenous antigen | 10/48 | 40/29008 | 4.61E-20 | 3.55E-17 | 1.89E-17 | Fcgr1/Fcgr3/Fcer1g/Fcgr2b/Ctss/H2-DMa/H2-Eb1/H2-Ab1/H2-Aa/Cd74 | 10 |
| DCs | GO:0019886 | antigen processing and presentation of exogenous peptide antigen via MHC class II | 8/48 | 20/29008 | 3.77E-18 | 1.94E-15 | 1.03E-15 | Fcer1g/Fcgr2b/Ctss/H2-DMa/H2-Eb1/H2-Ab1/H2-Aa/Cd74 | 8 |
| DCs | GO:0002495 | antigen processing and presentation of peptide antigen via MHC class II | 8/48 | 24/29008 | 2.19E-17 | 8.45E-15 | 4.50E-15 | Fcer1g/Fcgr2b/Ctss/H2-DMa/H2-Eb1/H2-Ab1/H2-Aa/Cd74 | 8 |
| DCs | GO:0002460 | adaptive immune response based on somatic recombination of immune receptors built from immunoglobulin superfamily domains | 16/48 | 480/29008 | 3.42E-17 | 1.05E-14 | 5.61E-15 | Fgl2/Fcgr1/Fcgr3/Ctsc/Clec4n/Fcer1g/C1qa/C1qc/Fcgr2b/C1qb/H2-DMa/H2-Eb1/H2-Ab1/H2-Aa/Arg1/Cd74 | 16 |
| DCs | GO:0002504 | antigen processing and presentation of peptide or polysaccharide antigen via MHC class II | 8/48 | 26/29008 | 4.64E-17 | 1.19E-14 | 6.35E-15 | Fcer1g/Fcgr2b/Ctss/H2-DMa/H2-Eb1/H2-Ab1/H2-Aa/Cd74 | 8 |
| DCs | GO:0048002 | antigen processing and presentation of peptide antigen | 10/48 | 78/29008 | 6.54E-17 | 1.44E-14 | 7.67E-15 | Fcgr1/Fcgr3/Fcer1g/Fcgr2b/Ctss/H2-DMa/H2-Eb1/H2-Ab1/H2-Aa/Cd74 | 10 |
| DCs | GO:0019882 | antigen processing and presentation | 11/48 | 126/29008 | 1.31E-16 | 2.53E-14 | 1.35E-14 | Fgl2/Fcgr1/Fcgr3/Fcer1g/Fcgr2b/Ctss/H2-DMa/H2-Eb1/H2-Ab1/H2-Aa/Cd74 | 11 |
| DCs | GO:0016064 | immunoglobulin mediated immune response | 13/48 | 284/29008 | 8.19E-16 | 1.32E-13 | 7.01E-14 | Fgl2/Fcgr1/Fcgr3/Fcer1g/C1qa/C1qc/Fcgr2b/C1qb/H2-DMa/H2-Eb1/H2-Ab1/H2-Aa/Cd74 | 13 |
| DCs | GO:0002449 | lymphocyte mediated immunity | 15/48 | 477/29008 | 9.29E-16 | 1.32E-13 | 7.01E-14 | Fgl2/Fcgr1/Fcgr3/Ctsc/Fcer1g/C1qa/C1qc/Fcgr2b/C1qb/H2-DMa/H2-Eb1/H2-Ab1/H2-Aa/Arg1/Cd74 | 15 |
| DCs | GO:0019724 | B cell mediated immunity | 13/48 | 287/29008 | 9.39E-16 | 1.32E-13 | 7.01E-14 | Fgl2/Fcgr1/Fcgr3/Fcer1g/C1qa/C1qc/Fcgr2b/C1qb/H2-DMa/H2-Eb1/H2-Ab1/H2-Aa/Cd74 | 13 |
| DCs | GO:0032103 | positive regulation of response to external stimulus | 13/48 | 427/29008 | 1.54E-13 | 1.97E-11 | 1.05E-11 | Fcgr1/Ly86/Fcgr3/Ctsc/Clec4n/Fcer1g/Aif1/Tyrobp/Alox5ap/Ctss/Arg1/Cd74/Cxcl10 | 13 |
| DCs | GO:0002683 | negative regulation of immune system process | 13/48 | 460/29008 | 3.95E-13 | 4.68E-11 | 2.49E-11 | Fgl2/Fcer1g/Laptm5/C1qc/Fcgr2b/Tyrobp/Ccl12/H2-Ab1/H2-Aa/Arg1/Cd74/Samhd1/Isg15 | 13 |
| DCs | GO:0034341 | response to interferon-gamma | 9/48 | 139/29008 | 1.47E-12 | 1.62E-10 | 8.60E-11 | Ccl12/Cxcl16/H2-Eb1/H2-Ab1/H2-Aa/Arg1/Cd74/Gbp2/Ccl4 | 9 |
| DCs | GO:0030595 | leukocyte chemotaxis | 10/48 | 226/29008 | 3.41E-12 | 3.50E-10 | 1.87E-10 | Cxcl9/Fcgr3/Fcer1g/Aif1/Ccl12/Cxcl16/Cd74/Cxcl10/Ccl4/Cxcl2 | 10 |
| DCs | GO:0030593 | neutrophil chemotaxis | 8/48 | 102/29008 | 5.94E-12 | 5.72E-10 | 3.05E-10 | Cxcl9/Fcgr3/Fcer1g/Ccl12/Cd74/Cxcl10/Ccl4/Cxcl2 | 8 |
| DCs | GO:0031349 | positive regulation of defense response | 10/48 | 273/29008 | 2.21E-11 | 2.00E-09 | 1.07E-09 | Fcgr1/Fcgr3/Ctsc/Clec4n/Fcer1g/Tyrobp/Alox5ap/Ctss/Arg1/Cd74 | 10 |
| DCs | GO:0002440 | production of molecular mediator of immune response | 11/48 | 380/29008 | 2.47E-11 | 2.12E-09 | 1.13E-09 | Fgl2/Fcer1g/Laptm5/Fcgr2b/H2-DMa/H2-Eb1/H2-Ab1/H2-Aa/Arg1/Cd74/Samhd1 | 11 |
| DCs | GO:0071621 | granulocyte chemotaxis | 8/48 | 127/29008 | 3.52E-11 | 2.86E-09 | 1.52E-09 | Cxcl9/Fcgr3/Fcer1g/Ccl12/Cd74/Cxcl10/Ccl4/Cxcl2 | 8 |
| DCs | GO:1990266 | neutrophil migration | 8/48 | 128/29008 | 3.75E-11 | 2.89E-09 | 1.54E-09 | Cxcl9/Fcgr3/Fcer1g/Ccl12/Cd74/Cxcl10/Ccl4/Cxcl2 | 8 |
| DCs | GO:0019221 | cytokine-mediated signaling pathway | 11/48 | 397/29008 | 3.95E-11 | 2.90E-09 | 1.54E-09 | Cxcl9/Fcer1g/Laptm5/Ccl12/Arg1/Cd74/Cxcl10/Samhd1/Isg15/Ccl4/Cxcl2 | 11 |
| DCs | GO:0060326 | cell chemotaxis | 10/48 | 312/29008 | 8.18E-11 | 5.73E-09 | 3.05E-09 | Cxcl9/Fcgr3/Fcer1g/Aif1/Ccl12/Cxcl16/Cd74/Cxcl10/Ccl4/Cxcl2 | 10 |
| DCs | GO:0050866 | negative regulation of cell activation | 9/48 | 223/29008 | 1.03E-10 | 6.91E-09 | 3.68E-09 | Fgl2/Laptm5/Fcgr2b/Tyrobp/H2-Ab1/H2-Aa/Arg1/Apoe/Cd74 | 9 |
| DCs | GO:0097529 | myeloid leukocyte migration | 9/48 | 226/29008 | 1.16E-10 | 7.46E-09 | 3.97E-09 | Cxcl9/Fcgr3/Fcer1g/Aif1/Ccl12/Cd74/Cxcl10/Ccl4/Cxcl2 | 9 |
| DCs | GO:1903037 | regulation of leukocyte cell-cell adhesion | 10/48 | 333/29008 | 1.54E-10 | 9.52E-09 | 5.07E-09 | Fgl2/Aif1/Laptm5/Cd83/H2-DMa/H2-Eb1/H2-Ab1/H2-Aa/Arg1/Cd74 | 10 |
| DCs | GO:0002819 | regulation of adaptive immune response | 9/48 | 236/29008 | 1.71E-10 | 1.01E-08 | 5.39E-09 | Fcgr1/Fcgr3/Clec4n/Fcer1g/Fcgr2b/H2-DMa/H2-Ab1/Arg1/Cd74 | 9 |
| DCs | GO:0050863 | regulation of T cell activation | 10/48 | 339/29008 | 1.84E-10 | 1.05E-08 | 5.58E-09 | Fgl2/Aif1/Laptm5/Cd83/H2-DMa/H2-Eb1/H2-Ab1/H2-Aa/Arg1/Cd74 | 10 |
| DCs | GO:0097530 | granulocyte migration | 8/48 | 157/29008 | 1.93E-10 | 1.06E-08 | 5.67E-09 | Cxcl9/Fcgr3/Fcer1g/Ccl12/Cd74/Cxcl10/Ccl4/Cxcl2 | 8 |
| DCs | GO:0070664 | negative regulation of leukocyte proliferation | 7/48 | 98/29008 | 2.66E-10 | 1.41E-08 | 7.52E-09 | Laptm5/Fcgr2b/Tyrobp/Ccl12/H2-Ab1/H2-Aa/Arg1 | 7 |
| DCs | GO:0051250 | negative regulation of lymphocyte activation | 8/48 | 168/29008 | 3.32E-10 | 1.70E-08 | 9.08E-09 | Fgl2/Laptm5/Fcgr2b/Tyrobp/H2-Ab1/H2-Aa/Arg1/Cd74 | 8 |
| DCs | GO:0001906 | cell killing | 9/48 | 260/29008 | 4.02E-10 | 2.00E-08 | 1.06E-08 | Cxcl9/Fcgr1/Fcgr3/Ctsc/Tyrobp/Ccl12/Arg1/Lyz2/Cxcl10 | 9 |
| DCs | GO:0070663 | regulation of leukocyte proliferation | 9/48 | 261/29008 | 4.16E-10 | 2.00E-08 | 1.07E-08 | Aif1/Laptm5/Fcgr2b/Tyrobp/Ccl12/H2-Ab1/H2-Aa/Arg1/Cd74 | 9 |
| DCs | GO:0007159 | leukocyte cell-cell adhesion | 10/48 | 373/29008 | 4.64E-10 | 2.11E-08 | 1.12E-08 | Fgl2/Aif1/Laptm5/Cd83/H2-DMa/H2-Eb1/H2-Ab1/H2-Aa/Arg1/Cd74 | 10 |
| DCs | GO:0050900 | leukocyte migration | 10/48 | 373/29008 | 4.64E-10 | 2.11E-08 | 1.12E-08 | Cxcl9/Fcgr3/Fcer1g/Aif1/Ccl12/Cxcl16/Cd74/Cxcl10/Ccl4/Cxcl2 | 10 |
| DCs | GO:0002888 | positive regulation of myeloid leukocyte mediated immunity | 5/48 | 29/29008 | 1.15E-09 | 5.08E-08 | 2.71E-08 | Fcgr1/Fcgr3/Fcer1g/Tyrobp/Arg1 | 5 |
| DCs | GO:0002695 | negative regulation of leukocyte activation | 8/48 | 200/29008 | 1.32E-09 | 5.66E-08 | 3.02E-08 | Fgl2/Laptm5/Fcgr2b/Tyrobp/H2-Ab1/H2-Aa/Arg1/Cd74 | 8 |
| DCs | GO:0071219 | cellular response to molecule of bacterial origin | 9/48 | 313/29008 | 2.05E-09 | 8.53E-08 | 4.54E-08 | Cxcl9/Ly86/Fcgr2b/Ccl12/Cxcl16/Gbp2/Cxcl10/Cxcl2/Zfp36 | 9 |
| DCs | GO:0002399 | MHC class II protein complex assembly | 4/48 | 11/29008 | 2.16E-09 | 8.53E-08 | 4.54E-08 | H2-DMa/H2-Eb1/H2-Ab1/H2-Aa | 4 |
| DCs | GO:0002503 | peptide antigen assembly with MHC class II protein complex | 4/48 | 11/29008 | 2.16E-09 | 8.53E-08 | 4.54E-08 | H2-DMa/H2-Eb1/H2-Ab1/H2-Aa | 4 |
| DCs | GO:0002822 | regulation of adaptive immune response based on somatic recombination of immune receptors built from immunoglobulin superfamily domains | 8/48 | 220/29008 | 2.80E-09 | 1.08E-07 | 5.75E-08 | Fcgr1/Fcgr3/Clec4n/Fcer1g/Fcgr2b/H2-DMa/H2-Ab1/Arg1 | 8 |
| DCs | GO:0022407 | regulation of cell-cell adhesion | 10/48 | 457/29008 | 3.27E-09 | 1.23E-07 | 6.55E-08 | Fgl2/Aif1/Laptm5/Cd83/H2-DMa/H2-Eb1/H2-Ab1/H2-Aa/Arg1/Cd74 | 10 |
| DCs | GO:0002831 | regulation of response to biotic stimulus | 9/48 | 337/29008 | 3.90E-09 | 1.40E-07 | 7.45E-08 | Fgl2/Ly86/Clec4n/Tyrobp/Arg1/Apoe/Cd74/Samhd1/Isg15 | 9 |
| DCs | GO:0071216 | cellular response to biotic stimulus | 9/48 | 337/29008 | 3.90E-09 | 1.40E-07 | 7.45E-08 | Cxcl9/Ly86/Fcgr2b/Ccl12/Cxcl16/Gbp2/Cxcl10/Cxcl2/Zfp36 | 9 |
| DCs | GO:0050727 | regulation of inflammatory response | 9/48 | 342/29008 | 4.43E-09 | 1.55E-07 | 8.28E-08 | Fcgr1/Fcgr3/Ctsc/Fcer1g/Fcgr2b/Alox5ap/Ctss/Apoe/Zfp36 | 9 |
| DCs | GO:0002501 | peptide antigen assembly with MHC protein complex | 4/48 | 13/29008 | 4.67E-09 | 1.60E-07 | 8.51E-08 | H2-DMa/H2-Eb1/H2-Ab1/H2-Aa | 4 |
| DCs | GO:0050670 | regulation of lymphocyte proliferation | 8/48 | 241/29008 | 5.73E-09 | 1.92E-07 | 1.02E-07 | Aif1/Laptm5/Fcgr2b/Tyrobp/H2-Ab1/H2-Aa/Arg1/Cd74 | 8 |
| DCs | GO:0032944 | regulation of mononuclear cell proliferation | 8/48 | 245/29008 | 6.51E-09 | 2.08E-07 | 1.11E-07 | Aif1/Laptm5/Fcgr2b/Tyrobp/H2-Ab1/H2-Aa/Arg1/Cd74 | 8 |
| DCs | GO:0002396 | MHC protein complex assembly | 4/48 | 14/29008 | 6.52E-09 | 2.08E-07 | 1.11E-07 | H2-DMa/H2-Eb1/H2-Ab1/H2-Aa | 4 |
| DCs | GO:0070661 | leukocyte proliferation | 9/48 | 358/29008 | 6.59E-09 | 2.08E-07 | 1.11E-07 | Aif1/Laptm5/Fcgr2b/Tyrobp/Ccl12/H2-Ab1/H2-Aa/Arg1/Cd74 | 9 |
| DCs | GO:0001819 | positive regulation of cytokine production | 10/48 | 500/29008 | 7.70E-09 | 2.33E-07 | 1.24E-07 | Fcgr3/Clec4n/Fcer1g/Aif1/Laptm5/Tyrobp/Cd83/Cd74/Isg15/Ccl4 | 10 |
| T01_Cd8+ T cells | GO:0050863 | regulation of T cell activation | 11/45 | 339/29008 | 3.36E-12 | 3.60E-09 | 1.85E-09 | Ccl5/Thy1/Rac2/Cd3e/Ptprc/Lck/Lat/Gimap3/Coro1a/Laptm5/Il2rg | 11 |
| T01_Cd8+ T cells | GO:0007159 | leukocyte cell-cell adhesion | 11/45 | 373/29008 | 9.42E-12 | 3.69E-09 | 1.90E-09 | Ccl5/Thy1/Rac2/Cd3e/Ptprc/Lck/Gimap3/Coro1a/Selplg/Laptm5/Il2rg | 11 |
| T01_Cd8+ T cells | GO:0002696 | positive regulation of leukocyte activation | 12/45 | 500/29008 | 1.04E-11 | 3.69E-09 | 1.90E-09 | Ccl5/Trbc2/Thy1/Trbc1/Cd3e/Ptprc/Lck/Klrc1/Klrk1/Gimap3/Coro1a/Il2rg | 12 |
| T01_Cd8+ T cells | GO:0051251 | positive regulation of lymphocyte activation | 11/45 | 436/29008 | 5.00E-11 | 1.34E-08 | 6.87E-09 | Ccl5/Trbc2/Thy1/Trbc1/Cd3e/Ptprc/Lck/Klrc1/Gimap3/Coro1a/Il2rg | 11 |
| T01_Cd8+ T cells | GO:0002429 | immune response-activating cell surface receptor signaling pathway | 10/45 | 340/29008 | 9.51E-11 | 1.74E-08 | 8.96E-09 | Trbc2/Thy1/Trbc1/Cd3e/Ptprc/Klrd1/Lck/Klrc1/Klrk1/Laptm5 | 10 |
| T01_Cd8+ T cells | GO:0002757 | immune response-activating signal transduction | 10/45 | 341/29008 | 9.79E-11 | 1.74E-08 | 8.96E-09 | Trbc2/Thy1/Trbc1/Cd3e/Ptprc/Klrd1/Lck/Klrc1/Klrk1/Laptm5 | 10 |
| T01_Cd8+ T cells | GO:0002768 | immune response-regulating cell surface receptor signaling pathway | 10/45 | 351/29008 | 1.30E-10 | 1.98E-08 | 1.02E-08 | Trbc2/Thy1/Trbc1/Cd3e/Ptprc/Klrd1/Lck/Klrc1/Klrk1/Laptm5 | 10 |
| T01_Cd8+ T cells | GO:0002253 | activation of immune response | 10/45 | 439/29008 | 1.13E-09 | 1.40E-07 | 7.22E-08 | Trbc2/Thy1/Trbc1/Cd3e/Ptprc/Klrd1/Lck/Klrc1/Klrk1/Laptm5 | 10 |
| T01_Cd8+ T cells | GO:0030098 | lymphocyte differentiation | 10/45 | 441/29008 | 1.18E-09 | 1.40E-07 | 7.22E-08 | Cd3g/Cd3d/Cd3e/Ptprc/Cd8a/Lck/Gimap3/Id2/Laptm5/Il2rg | 10 |
| T01_Cd8+ T cells | GO:0050870 | positive regulation of T cell activation | 8/45 | 216/29008 | 1.41E-09 | 1.51E-07 | 7.76E-08 | Ccl5/Thy1/Cd3e/Ptprc/Lck/Gimap3/Coro1a/Il2rg | 8 |
| T01_Cd8+ T cells | GO:1903037 | regulation of leukocyte cell-cell adhesion | 9/45 | 333/29008 | 1.92E-09 | 1.86E-07 | 9.57E-08 | Ccl5/Thy1/Cd3e/Ptprc/Lck/Gimap3/Coro1a/Laptm5/Il2rg | 9 |
| T01_Cd8+ T cells | GO:0002449 | lymphocyte mediated immunity | 10/45 | 477/29008 | 2.51E-09 | 2.23E-07 | 1.15E-07 | Trbc2/Trbc1/Ptprc/Klrd1/Cd8a/Klrc1/Klrk1/Gimap3/H2-Q7/Coro1a | 10 |
| T01_Cd8+ T cells | GO:1903131 | mononuclear cell differentiation | 10/45 | 489/29008 | 3.18E-09 | 2.47E-07 | 1.27E-07 | Cd3g/Cd3d/Cd3e/Ptprc/Cd8a/Lck/Gimap3/Id2/Laptm5/Il2rg | 10 |
| T01_Cd8+ T cells | GO:1903039 | positive regulation of leukocyte cell-cell adhesion | 8/45 | 240/29008 | 3.23E-09 | 2.47E-07 | 1.27E-07 | Ccl5/Thy1/Cd3e/Ptprc/Lck/Gimap3/Coro1a/Il2rg | 8 |
| T01_Cd8+ T cells | GO:0001912 | positive regulation of leukocyte mediated cytotoxicity | 6/45 | 89/29008 | 5.20E-09 | 3.71E-07 | 1.90E-07 | Ptprc/Klrd1/Klrc1/Klrk1/Gimap3/H2-Q7 | 6 |
| T01_Cd8+ T cells | GO:0001909 | leukocyte mediated cytotoxicity | 7/45 | 168/29008 | 7.27E-09 | 4.86E-07 | 2.50E-07 | Ptprc/Klrd1/Klrc1/Klrk1/Gimap3/H2-Q7/Coro1a | 7 |
| T01_Cd8+ T cells | GO:0031343 | positive regulation of cell killing | 6/45 | 100/29008 | 1.05E-08 | 6.62E-07 | 3.40E-07 | Ptprc/Klrd1/Klrc1/Klrk1/Gimap3/H2-Q7 | 6 |
| T01_Cd8+ T cells | GO:0045059 | positive thymic T cell selection | 4/45 | 17/29008 | 1.18E-08 | 7.04E-07 | 3.62E-07 | Cd3g/Cd3d/Cd3e/Ptprc | 4 |
| T01_Cd8+ T cells | GO:0022409 | positive regulation of cell-cell adhesion | 8/45 | 288/29008 | 1.34E-08 | 7.56E-07 | 3.88E-07 | Ccl5/Thy1/Cd3e/Ptprc/Lck/Gimap3/Coro1a/Il2rg | 8 |
| T01_Cd8+ T cells | GO:0030101 | natural killer cell activation | 6/45 | 106/29008 | 1.50E-08 | 8.00E-07 | 4.11E-07 | Ptprc/Klrc1/Klrk1/Il2rb/Id2/Coro1a | 6 |
| T01_Cd8+ T cells | GO:0002699 | positive regulation of immune effector process | 8/45 | 296/29008 | 1.66E-08 | 8.46E-07 | 4.35E-07 | Rac2/Ptprc/Klrd1/Klrc1/Klrk1/Gimap3/H2-Q7/Laptm5 | 8 |
| T01_Cd8+ T cells | GO:0030217 | T cell differentiation | 8/45 | 299/29008 | 1.80E-08 | 8.73E-07 | 4.49E-07 | Cd3g/Cd3d/Cd3e/Ptprc/Cd8a/Lck/Gimap3/Il2rg | 8 |
| T01_Cd8+ T cells | GO:0022407 | regulation of cell-cell adhesion | 9/45 | 457/29008 | 2.96E-08 | 1.38E-06 | 7.08E-07 | Ccl5/Thy1/Cd3e/Ptprc/Lck/Gimap3/Coro1a/Laptm5/Il2rg | 9 |
| T01_Cd8+ T cells | GO:0001910 | regulation of leukocyte mediated cytotoxicity | 6/45 | 120/29008 | 3.15E-08 | 1.40E-06 | 7.22E-07 | Ptprc/Klrd1/Klrc1/Klrk1/Gimap3/H2-Q7 | 6 |
| T01_Cd8+ T cells | GO:0045785 | positive regulation of cell adhesion | 9/45 | 471/29008 | 3.84E-08 | 1.64E-06 | 8.43E-07 | Ccl5/Thy1/Cd3e/Ptprc/Lck/Gimap3/Tnfrsf18/Coro1a/Il2rg | 9 |
| T01_Cd8+ T cells | GO:0001819 | positive regulation of cytokine production | 9/45 | 500/29008 | 6.38E-08 | 2.63E-06 | 1.35E-06 | Ccl5/Cd3e/Ptprc/Ltb/Cd2/Klrk1/Gimap3/Laptm5/Ccl4 | 9 |
| T01_Cd8+ T cells | GO:0031341 | regulation of cell killing | 6/45 | 139/29008 | 7.59E-08 | 3.00E-06 | 1.54E-06 | Ptprc/Klrd1/Klrc1/Klrk1/Gimap3/H2-Q7 | 6 |
| T01_Cd8+ T cells | GO:0045061 | thymic T cell selection | 4/45 | 28/29008 | 1.01E-07 | 3.84E-06 | 1.98E-06 | Cd3g/Cd3d/Cd3e/Ptprc | 4 |
| T01_Cd8+ T cells | GO:0001906 | cell killing | 7/45 | 260/29008 | 1.45E-07 | 5.36E-06 | 2.76E-06 | Ptprc/Klrd1/Klrc1/Klrk1/Gimap3/H2-Q7/Coro1a | 7 |
| T01_Cd8+ T cells | GO:0002708 | positive regulation of lymphocyte mediated immunity | 6/45 | 158/29008 | 1.62E-07 | 5.78E-06 | 2.97E-06 | Ptprc/Klrd1/Klrc1/Klrk1/Gimap3/H2-Q7 | 6 |
| T01_Cd8+ T cells | GO:0042267 | natural killer cell mediated cytotoxicity | 5/45 | 81/29008 | 1.68E-07 | 5.78E-06 | 2.97E-06 | Klrd1/Klrc1/Klrk1/Gimap3/Coro1a | 5 |
| T01_Cd8+ T cells | GO:0002228 | natural killer cell mediated immunity | 5/45 | 84/29008 | 2.01E-07 | 6.72E-06 | 3.46E-06 | Klrd1/Klrc1/Klrk1/Gimap3/Coro1a | 5 |
| T01_Cd8+ T cells | GO:0045954 | positive regulation of natural killer cell mediated cytotoxicity | 4/45 | 34/29008 | 2.26E-07 | 7.34E-06 | 3.77E-06 | Klrd1/Klrc1/Klrk1/Gimap3 | 4 |
| T01_Cd8+ T cells | GO:0002697 | regulation of immune effector process | 8/45 | 424/29008 | 2.62E-07 | 8.24E-06 | 4.23E-06 | Rac2/Ptprc/Klrd1/Klrc1/Klrk1/Gimap3/H2-Q7/Laptm5 | 8 |
| T01_Cd8+ T cells | GO:0002717 | positive regulation of natural killer cell mediated immunity | 4/45 | 36/29008 | 2.87E-07 | 8.76E-06 | 4.51E-06 | Klrd1/Klrc1/Klrk1/Gimap3 | 4 |
| T01_Cd8+ T cells | GO:0002703 | regulation of leukocyte mediated immunity | 7/45 | 297/29008 | 3.57E-07 | 1.04E-05 | 5.34E-06 | Rac2/Ptprc/Klrd1/Klrc1/Klrk1/Gimap3/H2-Q7 | 7 |
| T01_Cd8+ T cells | GO:0033077 | T cell differentiation in thymus | 5/45 | 95/29008 | 3.73E-07 | 1.04E-05 | 5.34E-06 | Cd3g/Cd3d/Cd3e/Ptprc/Il2rg | 5 |
| T01_Cd8+ T cells | GO:0002705 | positive regulation of leukocyte mediated immunity | 6/45 | 183/29008 | 3.86E-07 | 1.04E-05 | 5.34E-06 | Ptprc/Klrd1/Klrc1/Klrk1/Gimap3/H2-Q7 | 6 |
| T01_Cd8+ T cells | GO:0042129 | regulation of T cell proliferation | 6/45 | 183/29008 | 3.86E-07 | 1.04E-05 | 5.34E-06 | Ccl5/Rac2/Cd3e/Ptprc/Coro1a/Laptm5 | 6 |
| T01_Cd8+ T cells | GO:0002223 | stimulatory C-type lectin receptor signaling pathway | 3/45 | 10/29008 | 4.15E-07 | 1.04E-05 | 5.34E-06 | Klrd1/Klrc1/Klrk1 | 3 |
| T01_Cd8+ T cells | GO:1990840 | response to lectin | 3/45 | 10/29008 | 4.15E-07 | 1.04E-05 | 5.34E-06 | Klrd1/Klrc1/Klrk1 | 3 |
| T01_Cd8+ T cells | GO:1990858 | cellular response to lectin | 3/45 | 10/29008 | 4.15E-07 | 1.04E-05 | 5.34E-06 | Klrd1/Klrc1/Klrk1 | 3 |
| T01_Cd8+ T cells | GO:0050851 | antigen receptor-mediated signaling pathway | 7/45 | 304/29008 | 4.18E-07 | 1.04E-05 | 5.34E-06 | Trbc2/Thy1/Trbc1/Cd3e/Ptprc/Lck/Laptm5 | 7 |
| T01_Cd8+ T cells | GO:1902107 | positive regulation of leukocyte differentiation | 6/45 | 188/29008 | 4.52E-07 | 1.07E-05 | 5.51E-06 | Ccl5/Ptprc/Lck/Gimap3/Id2/Il2rg | 6 |
| T01_Cd8+ T cells | GO:1903708 | positive regulation of hemopoiesis | 6/45 | 188/29008 | 4.52E-07 | 1.07E-05 | 5.51E-06 | Ccl5/Ptprc/Lck/Gimap3/Id2/Il2rg | 6 |
| T01_Cd8+ T cells | GO:0043368 | positive T cell selection | 4/45 | 41/29008 | 4.91E-07 | 1.14E-05 | 5.86E-06 | Cd3g/Cd3d/Cd3e/Ptprc | 4 |
| T01_Cd8+ T cells | GO:0045588 | positive regulation of gamma-delta T cell differentiation | 3/45 | 11/29008 | 5.71E-07 | 1.27E-05 | 6.53E-06 | Ptprc/Lck/Gimap3 | 3 |
| T01_Cd8+ T cells | GO:0046645 | positive regulation of gamma-delta T cell activation | 3/45 | 11/29008 | 5.71E-07 | 1.27E-05 | 6.53E-06 | Ptprc/Lck/Gimap3 | 3 |
| T01_Cd8+ T cells | GO:0002220 | innate immune response activating cell surface receptor signaling pathway | 3/45 | 12/29008 | 7.60E-07 | 1.66E-05 | 8.52E-06 | Klrd1/Klrc1/Klrk1 | 3 |
| T01_Cd8+ T cells | GO:0006874 | cellular calcium ion homeostasis | 8/45 | 490/29008 | 7.80E-07 | 1.67E-05 | 8.57E-06 | Ccl5/Cd52/Thy1/Ptprc/Cxcr6/Lck/Gimap3/Coro1a | 8 |
| T02_Dnajb1 high | GO:0051251 | positive regulation of lymphocyte activation | 12/45 | 436/29008 | 2.10E-12 | 2.38E-09 | 1.24E-09 | Trbc2/Thy1/Klrc1/Cd28/Gpr183/Cd3e/Ifng/Trbc1/Lck/Ptprc/Ccl5/Coro1a | 12 |
| T02_Dnajb1 high | GO:0050863 | regulation of T cell activation | 11/45 | 339/29008 | 3.36E-12 | 2.38E-09 | 1.24E-09 | Thy1/Lat/Cd28/Cd3e/Ifng/Lck/Rac2/Ptprc/Ccl5/Coro1a/Ctla2a | 11 |
| T02_Dnajb1 high | GO:0002696 | positive regulation of leukocyte activation | 12/45 | 500/29008 | 1.04E-11 | 4.90E-09 | 2.54E-09 | Trbc2/Thy1/Klrc1/Cd28/Gpr183/Cd3e/Ifng/Trbc1/Lck/Ptprc/Ccl5/Coro1a | 12 |
| T02_Dnajb1 high | GO:0030098 | lymphocyte differentiation | 11/45 | 441/29008 | 5.65E-11 | 2.00E-08 | 1.04E-08 | Cd3g/Cd3d/Cd28/Gpr183/Cd3e/Ifng/Tnfaip3/Lck/Ptprc/Id2/Ctla2a | 11 |
| T02_Dnajb1 high | GO:1903131 | mononuclear cell differentiation | 11/45 | 489/29008 | 1.69E-10 | 4.80E-08 | 2.49E-08 | Cd3g/Cd3d/Cd28/Gpr183/Cd3e/Ifng/Tnfaip3/Lck/Ptprc/Id2/Ctla2a | 11 |
| T02_Dnajb1 high | GO:0045061 | thymic T cell selection | 5/45 | 28/29008 | 6.83E-10 | 1.51E-07 | 7.85E-08 | Cd3g/Cd3d/Cd28/Cd3e/Ptprc | 5 |
| T02_Dnajb1 high | GO:0030217 | T cell differentiation | 9/45 | 299/29008 | 7.45E-10 | 1.51E-07 | 7.85E-08 | Cd3g/Cd3d/Cd28/Gpr183/Cd3e/Ifng/Lck/Ptprc/Ctla2a | 9 |
| T02_Dnajb1 high | GO:0060326 | cell chemotaxis | 9/45 | 312/29008 | 1.08E-09 | 1.92E-07 | 9.98E-08 | Cxcr6/Gpr183/Ifng/Rac2/Ccl5/Coro1a/Ccl4/Nr4a1/Ccl3 | 9 |
| T02_Dnajb1 high | GO:0050870 | positive regulation of T cell activation | 8/45 | 216/29008 | 1.41E-09 | 2.14E-07 | 1.11E-07 | Thy1/Cd28/Cd3e/Ifng/Lck/Ptprc/Ccl5/Coro1a | 8 |
| T02_Dnajb1 high | GO:0002366 | leukocyte activation involved in immune response | 9/45 | 324/29008 | 1.51E-09 | 2.14E-07 | 1.11E-07 | Klrc1/Lat/Cd28/Gpr183/Ifng/Tnfaip3/Rac2/Ptprc/Coro1a | 9 |
| T02_Dnajb1 high | GO:0002263 | cell activation involved in immune response | 9/45 | 328/29008 | 1.68E-09 | 2.16E-07 | 1.12E-07 | Klrc1/Lat/Cd28/Gpr183/Ifng/Tnfaip3/Rac2/Ptprc/Coro1a | 9 |
| T02_Dnajb1 high | GO:0002429 | immune response-activating cell surface receptor signaling pathway | 9/45 | 340/29008 | 2.30E-09 | 2.40E-07 | 1.25E-07 | Trbc2/Thy1/Klrc1/Cd28/Cd3e/Ifng/Trbc1/Lck/Ptprc | 9 |
| T02_Dnajb1 high | GO:0002757 | immune response-activating signal transduction | 9/45 | 341/29008 | 2.36E-09 | 2.40E-07 | 1.25E-07 | Trbc2/Thy1/Klrc1/Cd28/Cd3e/Ifng/Trbc1/Lck/Ptprc | 9 |
| T02_Dnajb1 high | GO:0050671 | positive regulation of lymphocyte proliferation | 7/45 | 143/29008 | 2.37E-09 | 2.40E-07 | 1.25E-07 | Cd28/Gpr183/Cd3e/Ifng/Ptprc/Ccl5/Coro1a | 7 |
| T02_Dnajb1 high | GO:0032946 | positive regulation of mononuclear cell proliferation | 7/45 | 145/29008 | 2.61E-09 | 2.47E-07 | 1.28E-07 | Cd28/Gpr183/Cd3e/Ifng/Ptprc/Ccl5/Coro1a | 7 |
| T02_Dnajb1 high | GO:0002768 | immune response-regulating cell surface receptor signaling pathway | 9/45 | 351/29008 | 3.03E-09 | 2.63E-07 | 1.37E-07 | Trbc2/Thy1/Klrc1/Cd28/Cd3e/Ifng/Trbc1/Lck/Ptprc | 9 |
| T02_Dnajb1 high | GO:1903039 | positive regulation of leukocyte cell-cell adhesion | 8/45 | 240/29008 | 3.23E-09 | 2.63E-07 | 1.37E-07 | Thy1/Cd28/Cd3e/Ifng/Lck/Ptprc/Ccl5/Coro1a | 8 |
| T02_Dnajb1 high | GO:0050670 | regulation of lymphocyte proliferation | 8/45 | 241/29008 | 3.34E-09 | 2.63E-07 | 1.37E-07 | Cd28/Gpr183/Cd3e/Ifng/Rac2/Ptprc/Ccl5/Coro1a | 8 |
| T02_Dnajb1 high | GO:0032944 | regulation of mononuclear cell proliferation | 8/45 | 245/29008 | 3.80E-09 | 2.84E-07 | 1.47E-07 | Cd28/Gpr183/Cd3e/Ifng/Rac2/Ptprc/Ccl5/Coro1a | 8 |
| T02_Dnajb1 high | GO:0070665 | positive regulation of leukocyte proliferation | 7/45 | 157/29008 | 4.54E-09 | 3.22E-07 | 1.67E-07 | Cd28/Gpr183/Cd3e/Ifng/Ptprc/Ccl5/Coro1a | 7 |
| T02_Dnajb1 high | GO:0007159 | leukocyte cell-cell adhesion | 9/45 | 373/29008 | 5.14E-09 | 3.47E-07 | 1.80E-07 | Thy1/Cd28/Cd3e/Ifng/Lck/Rac2/Ptprc/Ccl5/Coro1a | 9 |
| T02_Dnajb1 high | GO:0070663 | regulation of leukocyte proliferation | 8/45 | 261/29008 | 6.24E-09 | 4.02E-07 | 2.09E-07 | Cd28/Gpr183/Cd3e/Ifng/Rac2/Ptprc/Ccl5/Coro1a | 8 |
| T02_Dnajb1 high | GO:0050864 | regulation of B cell activation | 8/45 | 275/29008 | 9.38E-09 | 5.78E-07 | 3.00E-07 | Trbc2/Cd28/Gpr183/Ifng/Trbc1/Tnfaip3/Ptprc/Id2 | 8 |
| T02_Dnajb1 high | GO:0045059 | positive thymic T cell selection | 4/45 | 17/29008 | 1.18E-08 | 6.80E-07 | 3.53E-07 | Cd3g/Cd3d/Cd3e/Ptprc | 4 |
| T02_Dnajb1 high | GO:0032680 | regulation of tumor necrosis factor production | 7/45 | 183/29008 | 1.31E-08 | 6.80E-07 | 3.53E-07 | Ifng/Cd2/Tnfaip3/Ptprc/Ccl4/Ccl3/Ifngr1 | 7 |
| T02_Dnajb1 high | GO:0042129 | regulation of T cell proliferation | 7/45 | 183/29008 | 1.31E-08 | 6.80E-07 | 3.53E-07 | Cd28/Cd3e/Ifng/Rac2/Ptprc/Ccl5/Coro1a | 7 |
| T02_Dnajb1 high | GO:0042102 | positive regulation of T cell proliferation | 6/45 | 104/29008 | 1.33E-08 | 6.80E-07 | 3.53E-07 | Cd28/Cd3e/Ifng/Ptprc/Ccl5/Coro1a | 6 |
| T02_Dnajb1 high | GO:0022409 | positive regulation of cell-cell adhesion | 8/45 | 288/29008 | 1.34E-08 | 6.80E-07 | 3.53E-07 | Thy1/Cd28/Cd3e/Ifng/Lck/Ptprc/Ccl5/Coro1a | 8 |
| T02_Dnajb1 high | GO:1903555 | regulation of tumor necrosis factor superfamily cytokine production | 7/45 | 186/29008 | 1.47E-08 | 7.19E-07 | 3.73E-07 | Ifng/Cd2/Tnfaip3/Ptprc/Ccl4/Ccl3/Ifngr1 | 7 |
| T02_Dnajb1 high | GO:0032640 | tumor necrosis factor production | 7/45 | 188/29008 | 1.58E-08 | 7.48E-07 | 3.89E-07 | Ifng/Cd2/Tnfaip3/Ptprc/Ccl4/Ccl3/Ifngr1 | 7 |
| T02_Dnajb1 high | GO:0071706 | tumor necrosis factor superfamily cytokine production | 7/45 | 191/29008 | 1.76E-08 | 8.07E-07 | 4.19E-07 | Ifng/Cd2/Tnfaip3/Ptprc/Ccl4/Ccl3/Ifngr1 | 7 |
| T02_Dnajb1 high | GO:0032760 | positive regulation of tumor necrosis factor production | 6/45 | 110/29008 | 1.87E-08 | 8.29E-07 | 4.31E-07 | Ifng/Cd2/Ptprc/Ccl4/Ccl3/Ifngr1 | 6 |
| T02_Dnajb1 high | GO:0050851 | antigen receptor-mediated signaling pathway | 8/45 | 304/29008 | 2.04E-08 | 8.51E-07 | 4.42E-07 | Trbc2/Thy1/Cd28/Cd3e/Ifng/Trbc1/Lck/Ptprc | 8 |
| T02_Dnajb1 high | GO:1903557 | positive regulation of tumor necrosis factor superfamily cytokine production | 6/45 | 112/29008 | 2.08E-08 | 8.51E-07 | 4.42E-07 | Ifng/Cd2/Ptprc/Ccl4/Ccl3/Ifngr1 | 6 |
| T02_Dnajb1 high | GO:0002253 | activation of immune response | 9/45 | 439/29008 | 2.10E-08 | 8.51E-07 | 4.42E-07 | Trbc2/Thy1/Klrc1/Cd28/Cd3e/Ifng/Trbc1/Lck/Ptprc | 9 |
| T02_Dnajb1 high | GO:0034113 | heterotypic cell-cell adhesion | 5/45 | 56/29008 | 2.57E-08 | 1.01E-06 | 5.26E-07 | Thy1/Cd2/Tnfaip3/Lck/Ptprc | 5 |
| T02_Dnajb1 high | GO:0022407 | regulation of cell-cell adhesion | 9/45 | 457/29008 | 2.96E-08 | 1.12E-06 | 5.82E-07 | Thy1/Cd28/Cd3e/Ifng/Tnfaip3/Lck/Ptprc/Ccl5/Coro1a | 9 |
| T02_Dnajb1 high | GO:1902105 | regulation of leukocyte differentiation | 8/45 | 320/29008 | 3.04E-08 | 1.12E-06 | 5.82E-07 | Cd28/Ifng/Lck/Ptprc/Ccl5/Id2/Ccl3/Ctla2a | 8 |
| T02_Dnajb1 high | GO:0045058 | T cell selection | 5/45 | 58/29008 | 3.08E-08 | 1.12E-06 | 5.82E-07 | Cd3g/Cd3d/Cd28/Cd3e/Ptprc | 5 |
| T02_Dnajb1 high | GO:0046651 | lymphocyte proliferation | 8/45 | 330/29008 | 3.85E-08 | 1.37E-06 | 7.10E-07 | Cd28/Gpr183/Cd3e/Ifng/Rac2/Ptprc/Ccl5/Coro1a | 8 |
| T02_Dnajb1 high | GO:1903037 | regulation of leukocyte cell-cell adhesion | 8/45 | 333/29008 | 4.13E-08 | 1.41E-06 | 7.32E-07 | Thy1/Cd28/Cd3e/Ifng/Lck/Ptprc/Ccl5/Coro1a | 8 |
| T02_Dnajb1 high | GO:0032943 | mononuclear cell proliferation | 8/45 | 334/29008 | 4.23E-08 | 1.41E-06 | 7.32E-07 | Cd28/Gpr183/Cd3e/Ifng/Rac2/Ptprc/Ccl5/Coro1a | 8 |
| T02_Dnajb1 high | GO:0002449 | lymphocyte mediated immunity | 9/45 | 477/29008 | 4.27E-08 | 1.41E-06 | 7.32E-07 | Trbc2/Klrc1/Cd28/Ifng/Gzmb/Trbc1/Ptprc/H2-Q7/Coro1a | 9 |
| T02_Dnajb1 high | GO:0002285 | lymphocyte activation involved in immune response | 7/45 | 219/29008 | 4.52E-08 | 1.46E-06 | 7.56E-07 | Klrc1/Cd28/Gpr183/Ifng/Tnfaip3/Ptprc/Coro1a | 7 |
| T02_Dnajb1 high | GO:0042098 | T cell proliferation | 7/45 | 221/29008 | 4.81E-08 | 1.51E-06 | 7.87E-07 | Cd28/Cd3e/Ifng/Rac2/Ptprc/Ccl5/Coro1a | 7 |
| T02_Dnajb1 high | GO:0030595 | leukocyte chemotaxis | 7/45 | 226/29008 | 5.60E-08 | 1.73E-06 | 8.97E-07 | Gpr183/Ifng/Rac2/Ccl5/Coro1a/Ccl4/Ccl3 | 7 |
| T02_Dnajb1 high | GO:0050852 | T cell receptor signaling pathway | 6/45 | 134/29008 | 6.10E-08 | 1.84E-06 | 9.56E-07 | Thy1/Cd28/Cd3e/Ifng/Lck/Ptprc | 6 |
| T02_Dnajb1 high | GO:0001819 | positive regulation of cytokine production | 9/45 | 500/29008 | 6.38E-08 | 1.89E-06 | 9.80E-07 | Cd28/Cd3e/Ifng/Cd2/Ptprc/Ccl5/Ccl4/Ccl3/Ifngr1 | 9 |
| T02_Dnajb1 high | GO:0070661 | leukocyte proliferation | 8/45 | 358/29008 | 7.21E-08 | 2.09E-06 | 1.08E-06 | Cd28/Gpr183/Cd3e/Ifng/Rac2/Ptprc/Ccl5/Coro1a | 8 |
| T02_Dnajb1 high | GO:0050900 | leukocyte migration | 8/45 | 373/29008 | 9.87E-08 | 2.80E-06 | 1.45E-06 | Thy1/Gpr183/Ifng/Rac2/Ccl5/Coro1a/Ccl4/Ccl3 | 8 |
| T03_Dnajb1 low | GO:0002696 | positive regulation of leukocyte activation | 13/47 | 500/29008 | 8.38E-13 | 1.28E-09 | 6.82E-10 | Xcl1/Ifng/Trbc2/Thy1/Klrc1/Cd3e/Klrk1/Lck/Tnfrsf4/Trbc1/Ptprc/Ccl5/Coro1a | 13 |
| T03_Dnajb1 low | GO:0051251 | positive regulation of lymphocyte activation | 12/47 | 436/29008 | 3.72E-12 | 2.83E-09 | 1.51E-09 | Xcl1/Ifng/Trbc2/Thy1/Klrc1/Cd3e/Lck/Tnfrsf4/Trbc1/Ptprc/Ccl5/Coro1a | 12 |
| T03_Dnajb1 low | GO:0050863 | regulation of T cell activation | 10/47 | 339/29008 | 1.47E-10 | 4.72E-08 | 2.52E-08 | Xcl1/Ifng/Thy1/Cd3e/Lck/Rac2/Ptprc/Ccl5/Coro1a/Ctla2a | 10 |
| T03_Dnajb1 low | GO:0002429 | immune response-activating cell surface receptor signaling pathway | 10/47 | 340/29008 | 1.51E-10 | 4.72E-08 | 2.52E-08 | Ifng/Trbc2/Klrd1/Thy1/Klrc1/Cd3e/Klrk1/Lck/Trbc1/Ptprc | 10 |
| T03_Dnajb1 low | GO:0002757 | immune response-activating signal transduction | 10/47 | 341/29008 | 1.56E-10 | 4.72E-08 | 2.52E-08 | Ifng/Trbc2/Klrd1/Thy1/Klrc1/Cd3e/Klrk1/Lck/Trbc1/Ptprc | 10 |
| T03_Dnajb1 low | GO:0002768 | immune response-regulating cell surface receptor signaling pathway | 10/47 | 351/29008 | 2.06E-10 | 4.72E-08 | 2.52E-08 | Ifng/Trbc2/Klrd1/Thy1/Klrc1/Cd3e/Klrk1/Lck/Trbc1/Ptprc | 10 |
| T03_Dnajb1 low | GO:0002449 | lymphocyte mediated immunity | 11/47 | 477/29008 | 2.17E-10 | 4.72E-08 | 2.52E-08 | Xcl1/Ifng/Trbc2/Klrd1/Klrc1/Cd8a/Klrk1/Trbc1/Ptprc/H2-Q7/Coro1a | 11 |
| T03_Dnajb1 low | GO:0002699 | positive regulation of immune effector process | 9/47 | 296/29008 | 1.03E-09 | 1.96E-07 | 1.05E-07 | Xcl1/Ifng/Klrd1/Klrc1/Klrk1/Tnfrsf4/Rac2/Ptprc/H2-Q7 | 9 |
| T03_Dnajb1 low | GO:0002697 | regulation of immune effector process | 10/47 | 424/29008 | 1.28E-09 | 2.17E-07 | 1.16E-07 | Xcl1/Ifng/Klrd1/Klrc1/Klrk1/Tnfaip3/Tnfrsf4/Rac2/Ptprc/H2-Q7 | 10 |
| T03_Dnajb1 low | GO:0060326 | cell chemotaxis | 9/47 | 312/29008 | 1.63E-09 | 2.37E-07 | 1.27E-07 | Xcl1/Ifng/Cxcr6/Rac2/Ccl4/Ccl5/Coro1a/Ccl3/Nr4a1 | 9 |
| T03_Dnajb1 low | GO:0002253 | activation of immune response | 10/47 | 439/29008 | 1.79E-09 | 2.37E-07 | 1.27E-07 | Ifng/Trbc2/Klrd1/Thy1/Klrc1/Cd3e/Klrk1/Lck/Trbc1/Ptprc | 10 |
| T03_Dnajb1 low | GO:0030098 | lymphocyte differentiation | 10/47 | 441/29008 | 1.87E-09 | 2.37E-07 | 1.27E-07 | Ifng/Cd3g/Cd3d/Cd3e/Cd8a/Lck/Tnfaip3/Ptprc/Id2/Ctla2a | 10 |
| T03_Dnajb1 low | GO:0050870 | positive regulation of T cell activation | 8/47 | 216/29008 | 2.03E-09 | 2.38E-07 | 1.27E-07 | Xcl1/Ifng/Thy1/Cd3e/Lck/Ptprc/Ccl5/Coro1a | 8 |
| T03_Dnajb1 low | GO:0042098 | T cell proliferation | 8/47 | 221/29008 | 2.43E-09 | 2.65E-07 | 1.42E-07 | Xcl1/Ifng/Cd3e/Tnfrsf4/Rac2/Ptprc/Ccl5/Coro1a | 8 |
| T03_Dnajb1 low | GO:0050671 | positive regulation of lymphocyte proliferation | 7/47 | 143/29008 | 3.25E-09 | 3.31E-07 | 1.77E-07 | Xcl1/Ifng/Cd3e/Tnfrsf4/Ptprc/Ccl5/Coro1a | 7 |
| T03_Dnajb1 low | GO:0032946 | positive regulation of mononuclear cell proliferation | 7/47 | 145/29008 | 3.59E-09 | 3.42E-07 | 1.82E-07 | Xcl1/Ifng/Cd3e/Tnfrsf4/Ptprc/Ccl5/Coro1a | 7 |
| T03_Dnajb1 low | GO:1903039 | positive regulation of leukocyte cell-cell adhesion | 8/47 | 240/29008 | 4.65E-09 | 4.02E-07 | 2.15E-07 | Xcl1/Ifng/Thy1/Cd3e/Lck/Ptprc/Ccl5/Coro1a | 8 |
| T03_Dnajb1 low | GO:0050670 | regulation of lymphocyte proliferation | 8/47 | 241/29008 | 4.81E-09 | 4.02E-07 | 2.15E-07 | Xcl1/Ifng/Cd3e/Tnfrsf4/Rac2/Ptprc/Ccl5/Coro1a | 8 |
| T03_Dnajb1 low | GO:1903131 | mononuclear cell differentiation | 10/47 | 489/29008 | 5.01E-09 | 4.02E-07 | 2.15E-07 | Ifng/Cd3g/Cd3d/Cd3e/Cd8a/Lck/Tnfaip3/Ptprc/Id2/Ctla2a | 10 |
| T03_Dnajb1 low | GO:0032944 | regulation of mononuclear cell proliferation | 8/47 | 245/29008 | 5.47E-09 | 4.17E-07 | 2.22E-07 | Xcl1/Ifng/Cd3e/Tnfrsf4/Rac2/Ptprc/Ccl5/Coro1a | 8 |
| T03_Dnajb1 low | GO:0070665 | positive regulation of leukocyte proliferation | 7/47 | 157/29008 | 6.24E-09 | 4.51E-07 | 2.41E-07 | Xcl1/Ifng/Cd3e/Tnfrsf4/Ptprc/Ccl5/Coro1a | 7 |
| T03_Dnajb1 low | GO:0002708 | positive regulation of lymphocyte mediated immunity | 7/47 | 158/29008 | 6.52E-09 | 4.51E-07 | 2.41E-07 | Xcl1/Ifng/Klrd1/Klrc1/Klrk1/Ptprc/H2-Q7 | 7 |
| T03_Dnajb1 low | GO:0007159 | leukocyte cell-cell adhesion | 9/47 | 373/29008 | 7.73E-09 | 5.12E-07 | 2.73E-07 | Xcl1/Ifng/Thy1/Cd3e/Lck/Rac2/Ptprc/Ccl5/Coro1a | 9 |
| T03_Dnajb1 low | GO:0070663 | regulation of leukocyte proliferation | 8/47 | 261/29008 | 8.96E-09 | 5.69E-07 | 3.04E-07 | Xcl1/Ifng/Cd3e/Tnfrsf4/Rac2/Ptprc/Ccl5/Coro1a | 8 |
| T03_Dnajb1 low | GO:0050864 | regulation of B cell activation | 8/47 | 275/29008 | 1.35E-08 | 8.00E-07 | 4.27E-07 | Ifng/Trbc2/Tnfaip3/Tnfrsf4/Samsn1/Trbc1/Ptprc/Id2 | 8 |
| T03_Dnajb1 low | GO:0031343 | positive regulation of cell killing | 6/47 | 100/29008 | 1.38E-08 | 8.00E-07 | 4.27E-07 | Ifng/Klrd1/Klrc1/Klrk1/Ptprc/H2-Q7 | 6 |
| T03_Dnajb1 low | GO:0045059 | positive thymic T cell selection | 4/47 | 17/29008 | 1.42E-08 | 8.00E-07 | 4.27E-07 | Cd3g/Cd3d/Cd3e/Ptprc | 4 |
| T03_Dnajb1 low | GO:0030593 | neutrophil chemotaxis | 6/47 | 102/29008 | 1.56E-08 | 8.47E-07 | 4.52E-07 | Xcl1/Ifng/Rac2/Ccl4/Ccl5/Ccl3 | 6 |
| T03_Dnajb1 low | GO:0042102 | positive regulation of T cell proliferation | 6/47 | 104/29008 | 1.75E-08 | 8.86E-07 | 4.73E-07 | Xcl1/Ifng/Cd3e/Ptprc/Ccl5/Coro1a | 6 |
| T03_Dnajb1 low | GO:0002705 | positive regulation of leukocyte mediated immunity | 7/47 | 183/29008 | 1.80E-08 | 8.86E-07 | 4.73E-07 | Xcl1/Ifng/Klrd1/Klrc1/Klrk1/Ptprc/H2-Q7 | 7 |
| T03_Dnajb1 low | GO:0042129 | regulation of T cell proliferation | 7/47 | 183/29008 | 1.80E-08 | 8.86E-07 | 4.73E-07 | Xcl1/Ifng/Cd3e/Rac2/Ptprc/Ccl5/Coro1a | 7 |
| T03_Dnajb1 low | GO:0022409 | positive regulation of cell-cell adhesion | 8/47 | 288/29008 | 1.93E-08 | 9.06E-07 | 4.84E-07 | Xcl1/Ifng/Thy1/Cd3e/Lck/Ptprc/Ccl5/Coro1a | 8 |
| T03_Dnajb1 low | GO:0030101 | natural killer cell activation | 6/47 | 106/29008 | 1.96E-08 | 9.06E-07 | 4.84E-07 | Klrc1/Klrk1/Il2rb/Ptprc/Coro1a/Id2 | 6 |
| T03_Dnajb1 low | GO:0002703 | regulation of leukocyte mediated immunity | 8/47 | 297/29008 | 2.44E-08 | 1.10E-06 | 5.85E-07 | Xcl1/Ifng/Klrd1/Klrc1/Klrk1/Rac2/Ptprc/H2-Q7 | 8 |
| T03_Dnajb1 low | GO:0030217 | T cell differentiation | 8/47 | 299/29008 | 2.57E-08 | 1.12E-06 | 5.99E-07 | Ifng/Cd3g/Cd3d/Cd3e/Cd8a/Lck/Ptprc/Ctla2a | 8 |
| T03_Dnajb1 low | GO:0071346 | cellular response to interferon-gamma | 6/47 | 112/29008 | 2.73E-08 | 1.16E-06 | 6.17E-07 | Xcl1/Ifng/H2-Q7/Ccl4/Ccl5/Ccl3 | 6 |
| T03_Dnajb1 low | GO:0022407 | regulation of cell-cell adhesion | 9/47 | 457/29008 | 4.43E-08 | 1.83E-06 | 9.74E-07 | Xcl1/Ifng/Thy1/Cd3e/Lck/Tnfaip3/Ptprc/Ccl5/Coro1a | 9 |
| T03_Dnajb1 low | GO:0002683 | negative regulation of immune system process | 9/47 | 460/29008 | 4.69E-08 | 1.88E-06 | 1.00E-06 | Ifng/Pdcd1/Klrd1/Thy1/Tnfaip3/Tnfrsf4/Samsn1/Ptprc/Id2 | 9 |
| T03_Dnajb1 low | GO:0046651 | lymphocyte proliferation | 8/47 | 330/29008 | 5.51E-08 | 2.15E-06 | 1.15E-06 | Xcl1/Ifng/Cd3e/Tnfrsf4/Rac2/Ptprc/Ccl5/Coro1a | 8 |
| T03_Dnajb1 low | GO:0071621 | granulocyte chemotaxis | 6/47 | 127/29008 | 5.80E-08 | 2.15E-06 | 1.15E-06 | Xcl1/Ifng/Rac2/Ccl4/Ccl5/Ccl3 | 6 |
| T03_Dnajb1 low | GO:1903037 | regulation of leukocyte cell-cell adhesion | 8/47 | 333/29008 | 5.91E-08 | 2.15E-06 | 1.15E-06 | Xcl1/Ifng/Thy1/Cd3e/Lck/Ptprc/Ccl5/Coro1a | 8 |
| T03_Dnajb1 low | GO:0032943 | mononuclear cell proliferation | 8/47 | 334/29008 | 6.04E-08 | 2.15E-06 | 1.15E-06 | Xcl1/Ifng/Cd3e/Tnfrsf4/Rac2/Ptprc/Ccl5/Coro1a | 8 |
| T03_Dnajb1 low | GO:1990266 | neutrophil migration | 6/47 | 128/29008 | 6.07E-08 | 2.15E-06 | 1.15E-06 | Xcl1/Ifng/Rac2/Ccl4/Ccl5/Ccl3 | 6 |
| T03_Dnajb1 low | GO:0051651 | maintenance of location in cell | 7/47 | 220/29008 | 6.38E-08 | 2.21E-06 | 1.18E-06 | Xcl1/Thy1/Lck/Ptprc/Srgn/Coro1a/Tmsb4x | 7 |
| T03_Dnajb1 low | GO:0002460 | adaptive immune response based on somatic recombination of immune receptors built from immunoglobulin superfamily domains | 9/47 | 480/29008 | 6.73E-08 | 2.28E-06 | 1.22E-06 | Xcl1/Ifng/Trbc2/Klrd1/Cd8a/Tnfaip3/Trbc1/Ptprc/H2-Q7 | 9 |
| T03_Dnajb1 low | GO:0002706 | regulation of lymphocyte mediated immunity | 7/47 | 225/29008 | 7.43E-08 | 2.43E-06 | 1.30E-06 | Xcl1/Ifng/Klrd1/Klrc1/Klrk1/Ptprc/H2-Q7 | 7 |
| T03_Dnajb1 low | GO:0007204 | positive regulation of cytosolic calcium ion concentration | 8/47 | 344/29008 | 7.58E-08 | 2.43E-06 | 1.30E-06 | Xcl1/Thy1/Cxcr6/Lck/Ptprc/Coro1a/Cd52/Ccl3 | 8 |
| T03_Dnajb1 low | GO:0030595 | leukocyte chemotaxis | 7/47 | 226/29008 | 7.66E-08 | 2.43E-06 | 1.30E-06 | Xcl1/Ifng/Rac2/Ccl4/Ccl5/Coro1a/Ccl3 | 7 |
| T03_Dnajb1 low | GO:0006874 | cellular calcium ion homeostasis | 9/47 | 490/29008 | 8.02E-08 | 2.50E-06 | 1.33E-06 | Xcl1/Thy1/Cxcr6/Lck/Ptprc/Ccl5/Coro1a/Cd52/Ccl3 | 9 |
| T03_Dnajb1 low | GO:0001819 | positive regulation of cytokine production | 9/47 | 500/29008 | 9.52E-08 | 2.90E-06 | 1.55E-06 | Xcl1/Ifng/Cd3e/Klrk1/Ltb/Ptprc/Ccl4/Ccl5/Ccl3 | 9 |
| T04_Cd4+ T cells | GO:0051251 | positive regulation of lymphocyte activation | 12/48 | 436/29008 | 4.89E-12 | 5.16E-09 | 2.88E-09 | Icos/Cd4/Cd3e/Tnfrsf4/Trbc2/Trbc1/Thy1/Ifng/Coro1a/Ptprc/Il2rg/Ccl5 | 12 |
| T04_Cd4+ T cells | GO:0050863 | regulation of T cell activation | 11/48 | 339/29008 | 7.26E-12 | 5.16E-09 | 2.88E-09 | Icos/Ctla4/Cd4/Cd3e/Thy1/Ifng/Rac2/Coro1a/Ptprc/Il2rg/Ccl5 | 11 |
| T04_Cd4+ T cells | GO:0007159 | leukocyte cell-cell adhesion | 11/48 | 373/29008 | 2.03E-11 | 8.52E-09 | 4.76E-09 | Icos/Ctla4/Cd4/Cd3e/Thy1/Ifng/Rac2/Coro1a/Ptprc/Il2rg/Ccl5 | 11 |
| T04_Cd4+ T cells | GO:0002696 | positive regulation of leukocyte activation | 12/48 | 500/29008 | 2.39E-11 | 8.52E-09 | 4.76E-09 | Icos/Cd4/Cd3e/Tnfrsf4/Trbc2/Trbc1/Thy1/Ifng/Coro1a/Ptprc/Il2rg/Ccl5 | 12 |
| T04_Cd4+ T cells | GO:0050870 | positive regulation of T cell activation | 9/48 | 216/29008 | 7.76E-11 | 2.21E-08 | 1.23E-08 | Icos/Cd4/Cd3e/Thy1/Ifng/Coro1a/Ptprc/Il2rg/Ccl5 | 9 |
| T04_Cd4+ T cells | GO:0042098 | T cell proliferation | 9/48 | 221/29008 | 9.52E-11 | 2.26E-08 | 1.26E-08 | Ctla4/Cd4/Cd3e/Tnfrsf4/Ifng/Rac2/Coro1a/Ptprc/Ccl5 | 9 |
| T04_Cd4+ T cells | GO:1903037 | regulation of leukocyte cell-cell adhesion | 10/48 | 333/29008 | 1.54E-10 | 3.14E-08 | 1.75E-08 | Icos/Ctla4/Cd4/Cd3e/Thy1/Ifng/Coro1a/Ptprc/Il2rg/Ccl5 | 10 |
| T04_Cd4+ T cells | GO:1903039 | positive regulation of leukocyte cell-cell adhesion | 9/48 | 240/29008 | 1.98E-10 | 3.25E-08 | 1.81E-08 | Icos/Cd4/Cd3e/Thy1/Ifng/Coro1a/Ptprc/Il2rg/Ccl5 | 9 |
| T04_Cd4+ T cells | GO:0050670 | regulation of lymphocyte proliferation | 9/48 | 241/29008 | 2.05E-10 | 3.25E-08 | 1.81E-08 | Ctla4/Cd4/Cd3e/Tnfrsf4/Ifng/Rac2/Coro1a/Ptprc/Ccl5 | 9 |
| T04_Cd4+ T cells | GO:0032944 | regulation of mononuclear cell proliferation | 9/48 | 245/29008 | 2.38E-10 | 3.38E-08 | 1.89E-08 | Ctla4/Cd4/Cd3e/Tnfrsf4/Ifng/Rac2/Coro1a/Ptprc/Ccl5 | 9 |
| T04_Cd4+ T cells | GO:0070663 | regulation of leukocyte proliferation | 9/48 | 261/29008 | 4.16E-10 | 5.38E-08 | 3.00E-08 | Ctla4/Cd4/Cd3e/Tnfrsf4/Ifng/Rac2/Coro1a/Ptprc/Ccl5 | 9 |
| T04_Cd4+ T cells | GO:0042129 | regulation of T cell proliferation | 8/48 | 183/29008 | 6.54E-10 | 7.21E-08 | 4.03E-08 | Ctla4/Cd4/Cd3e/Ifng/Rac2/Coro1a/Ptprc/Ccl5 | 8 |
| T04_Cd4+ T cells | GO:0050864 | regulation of B cell activation | 9/48 | 275/29008 | 6.59E-10 | 7.21E-08 | 4.03E-08 | Ctla4/Tnfrsf4/Trbc2/Trbc1/Samsn1/Ifng/Ptprc/Id2/Il2rg | 9 |
| T04_Cd4+ T cells | GO:0022409 | positive regulation of cell-cell adhesion | 9/48 | 288/29008 | 9.89E-10 | 1.00E-07 | 5.61E-08 | Icos/Cd4/Cd3e/Thy1/Ifng/Coro1a/Ptprc/Il2rg/Ccl5 | 9 |
| T04_Cd4+ T cells | GO:0046651 | lymphocyte proliferation | 9/48 | 330/29008 | 3.25E-09 | 2.91E-07 | 1.62E-07 | Ctla4/Cd4/Cd3e/Tnfrsf4/Ifng/Rac2/Coro1a/Ptprc/Ccl5 | 9 |
| T04_Cd4+ T cells | GO:0022407 | regulation of cell-cell adhesion | 10/48 | 457/29008 | 3.27E-09 | 2.91E-07 | 1.62E-07 | Icos/Ctla4/Cd4/Cd3e/Thy1/Ifng/Coro1a/Ptprc/Il2rg/Ccl5 | 10 |
| T04_Cd4+ T cells | GO:0032943 | mononuclear cell proliferation | 9/48 | 334/29008 | 3.61E-09 | 3.00E-07 | 1.68E-07 | Ctla4/Cd4/Cd3e/Tnfrsf4/Ifng/Rac2/Coro1a/Ptprc/Ccl5 | 9 |
| T04_Cd4+ T cells | GO:0050671 | positive regulation of lymphocyte proliferation | 7/48 | 143/29008 | 3.79E-09 | 3.00E-07 | 1.68E-07 | Cd4/Cd3e/Tnfrsf4/Ifng/Coro1a/Ptprc/Ccl5 | 7 |
| T04_Cd4+ T cells | GO:0032946 | positive regulation of mononuclear cell proliferation | 7/48 | 145/29008 | 4.18E-09 | 3.10E-07 | 1.73E-07 | Cd4/Cd3e/Tnfrsf4/Ifng/Coro1a/Ptprc/Ccl5 | 7 |
| T04_Cd4+ T cells | GO:0045785 | positive regulation of cell adhesion | 10/48 | 471/29008 | 4.36E-09 | 3.10E-07 | 1.73E-07 | Tnfrsf18/Icos/Cd4/Cd3e/Thy1/Ifng/Coro1a/Ptprc/Il2rg/Ccl5 | 10 |
| T04_Cd4+ T cells | GO:0070661 | leukocyte proliferation | 9/48 | 358/29008 | 6.59E-09 | 4.47E-07 | 2.50E-07 | Ctla4/Cd4/Cd3e/Tnfrsf4/Ifng/Rac2/Coro1a/Ptprc/Ccl5 | 9 |
| T04_Cd4+ T cells | GO:0070665 | positive regulation of leukocyte proliferation | 7/48 | 157/29008 | 7.27E-09 | 4.70E-07 | 2.63E-07 | Cd4/Cd3e/Tnfrsf4/Ifng/Coro1a/Ptprc/Ccl5 | 7 |
| T04_Cd4+ T cells | GO:0050730 | regulation of peptidyl-tyrosine phosphorylation | 8/48 | 266/29008 | 1.24E-08 | 7.65E-07 | 4.27E-07 | Tnfrsf18/Cd4/Cd3e/Thy1/Samsn1/Ifng/Ptprc/Ccl5 | 8 |
| T04_Cd4+ T cells | GO:0045059 | positive thymic T cell selection | 4/48 | 17/29008 | 1.55E-08 | 9.16E-07 | 5.12E-07 | Cd3g/Cd3e/Cd3d/Ptprc | 4 |
| T04_Cd4+ T cells | GO:0042102 | positive regulation of T cell proliferation | 6/48 | 104/29008 | 1.99E-08 | 1.13E-06 | 6.34E-07 | Cd4/Cd3e/Ifng/Coro1a/Ptprc/Ccl5 | 6 |
| T04_Cd4+ T cells | GO:0030217 | T cell differentiation | 8/48 | 299/29008 | 3.06E-08 | 1.68E-06 | 9.36E-07 | Ctla4/Cd4/Cd3g/Cd3e/Cd3d/Ifng/Ptprc/Il2rg | 8 |
| T04_Cd4+ T cells | GO:0042113 | B cell activation | 9/48 | 434/29008 | 3.46E-08 | 1.82E-06 | 1.02E-06 | Ctla4/Tnfrsf4/Trbc2/Trbc1/Samsn1/Ifng/Ptprc/Id2/Il2rg | 9 |
| T04_Cd4+ T cells | GO:0030098 | lymphocyte differentiation | 9/48 | 441/29008 | 3.97E-08 | 2.02E-06 | 1.13E-06 | Ctla4/Cd4/Cd3g/Cd3e/Cd3d/Ifng/Ptprc/Id2/Il2rg | 9 |
| T04_Cd4+ T cells | GO:0060326 | cell chemotaxis | 8/48 | 312/29008 | 4.25E-08 | 2.04E-06 | 1.14E-06 | Ccl1/Cxcr6/Ifng/Rac2/Coro1a/Dusp1/Ccl5/Nr4a1 | 8 |
| T04_Cd4+ T cells | GO:0045058 | T cell selection | 5/48 | 58/29008 | 4.29E-08 | 2.04E-06 | 1.14E-06 | Cd4/Cd3g/Cd3e/Cd3d/Ptprc | 5 |
| T04_Cd4+ T cells | GO:0018108 | peptidyl-tyrosine phosphorylation | 8/48 | 322/29008 | 5.42E-08 | 2.49E-06 | 1.39E-06 | Tnfrsf18/Cd4/Cd3e/Thy1/Samsn1/Ifng/Ptprc/Ccl5 | 8 |
| T04_Cd4+ T cells | GO:0018212 | peptidyl-tyrosine modification | 8/48 | 325/29008 | 5.82E-08 | 2.59E-06 | 1.45E-06 | Tnfrsf18/Cd4/Cd3e/Thy1/Samsn1/Ifng/Ptprc/Ccl5 | 8 |
| T04_Cd4+ T cells | GO:1903131 | mononuclear cell differentiation | 9/48 | 489/29008 | 9.56E-08 | 4.12E-06 | 2.30E-06 | Ctla4/Cd4/Cd3g/Cd3e/Cd3d/Ifng/Ptprc/Id2/Il2rg | 9 |
| T04_Cd4+ T cells | GO:0045061 | thymic T cell selection | 4/48 | 28/29008 | 1.31E-07 | 5.49E-06 | 3.07E-06 | Cd3g/Cd3e/Cd3d/Ptprc | 4 |
| T04_Cd4+ T cells | GO:0050900 | leukocyte migration | 8/48 | 373/29008 | 1.67E-07 | 6.79E-06 | 3.79E-06 | Tnfrsf18/Ccl1/Thy1/Ifng/Rac2/Coro1a/Dusp1/Ccl5 | 8 |
| T04_Cd4+ T cells | GO:0019221 | cytokine-mediated signaling pathway | 8/48 | 397/29008 | 2.68E-07 | 1.05E-05 | 5.87E-06 | Cd4/Ccl1/Ifng/Il1r2/Ptprc/Il2rg/Ccl5/Nfkbia | 8 |
| T04_Cd4+ T cells | GO:1903706 | regulation of hemopoiesis | 8/48 | 398/29008 | 2.73E-07 | 1.05E-05 | 5.87E-06 | Ctla4/Cd4/Ifng/Ptprc/Id2/Il2rg/Ccl5/Nfkbia | 8 |
| T04_Cd4+ T cells | GO:0033077 | T cell differentiation in thymus | 5/48 | 95/29008 | 5.19E-07 | 1.94E-05 | 1.09E-05 | Cd3g/Cd3e/Cd3d/Ptprc/Il2rg | 5 |
| T04_Cd4+ T cells | GO:0043368 | positive T cell selection | 4/48 | 41/29008 | 6.39E-07 | 2.27E-05 | 1.27E-05 | Cd3g/Cd3e/Cd3d/Ptprc | 4 |
| T04_Cd4+ T cells | GO:0050851 | antigen receptor-mediated signaling pathway | 7/48 | 304/29008 | 6.60E-07 | 2.27E-05 | 1.27E-05 | Ctla4/Cd3e/Trbc2/Trbc1/Thy1/Ifng/Ptprc | 7 |
| T04_Cd4+ T cells | GO:1902107 | positive regulation of leukocyte differentiation | 6/48 | 188/29008 | 6.70E-07 | 2.27E-05 | 1.27E-05 | Cd4/Ifng/Ptprc/Id2/Il2rg/Ccl5 | 6 |
| T04_Cd4+ T cells | GO:1903708 | positive regulation of hemopoiesis | 6/48 | 188/29008 | 6.70E-07 | 2.27E-05 | 1.27E-05 | Cd4/Ifng/Ptprc/Id2/Il2rg/Ccl5 | 6 |
| T04_Cd4+ T cells | GO:0050731 | positive regulation of peptidyl-tyrosine phosphorylation | 6/48 | 192/29008 | 7.57E-07 | 2.51E-05 | 1.40E-05 | Tnfrsf18/Cd4/Cd3e/Ifng/Ptprc/Ccl5 | 6 |
| T04_Cd4+ T cells | GO:0002683 | negative regulation of immune system process | 8/48 | 460/29008 | 8.14E-07 | 2.63E-05 | 1.47E-05 | Ctla4/Tnfrsf4/Thy1/Samsn1/Ifng/Ptprc/Id2/Dusp1 | 8 |
| T04_Cd4+ T cells | GO:1902105 | regulation of leukocyte differentiation | 7/48 | 320/29008 | 9.30E-07 | 2.94E-05 | 1.64E-05 | Ctla4/Cd4/Ifng/Ptprc/Id2/Il2rg/Ccl5 | 7 |
| T04_Cd4+ T cells | GO:0051235 | maintenance of location | 7/48 | 335/29008 | 1.26E-06 | 3.90E-05 | 2.18E-05 | Cd4/Thy1/Srgn/Coro1a/Ptprc/Tmsb4x/Nfkbia | 7 |
| T04_Cd4+ T cells | GO:0006874 | cellular calcium ion homeostasis | 8/48 | 490/29008 | 1.31E-06 | 3.95E-05 | 2.21E-05 | Cd4/Ccl1/Thy1/Cxcr6/Coro1a/Ptprc/Cd52/Ccl5 | 8 |
| T04_Cd4+ T cells | GO:0002429 | immune response-activating cell surface receptor signaling pathway | 7/48 | 340/29008 | 1.39E-06 | 4.12E-05 | 2.30E-05 | Ctla4/Cd3e/Trbc2/Trbc1/Thy1/Ifng/Ptprc | 7 |
| T04_Cd4+ T cells | GO:0002757 | immune response-activating signal transduction | 7/48 | 341/29008 | 1.42E-06 | 4.12E-05 | 2.30E-05 | Ctla4/Cd3e/Trbc2/Trbc1/Thy1/Ifng/Ptprc | 7 |
| T04_Cd4+ T cells | GO:0007204 | positive regulation of cytosolic calcium ion concentration | 7/48 | 344/29008 | 1.50E-06 | 4.28E-05 | 2.39E-05 | Cd4/Ccl1/Thy1/Cxcr6/Coro1a/Ptprc/Cd52 | 7 |

**Table S3: Potential target proteins for Lobeline.**

| Protein Group | Protein ID | Protein Accession |  | Peptide | P value |
| --- | --- | --- | --- | --- | --- |
| 89 | 95 | Q03265 | ATP synthase subunit alpha mitochondrial OS=Mus musculus OX=10090 GN=Atp5f1a PE=1 SV=1 | R.ALPRRAGLVSK.N | 0.009134223 |
| 1890 | 1860 | Q9WVQ5 | Methylthioribulose-1-phosphate dehydratase OS=Mus musculus OX=10090 GN=Apip PE=1 SV=1 | R.IQPEDMFVC(+57.02)DINEQDISGPPASK.K | 0.003750443 |
| 260 | 162 | Q3TXS7 | 26S proteasome non-ATPase regulatory subunit 1 OS=Mus musculus OX=10090 GN=Psmd1 PE=1 SV=1 | R.NNNTDLMILK.N | 0.000717828 |
| 310 | 151 | P25206 | DNA replication licensing factor MCM3 OS=Mus musculus OX=10090 GN=Mcm3 PE=1 SV=2 | R.VTIAK(+32.06)AGIHAR.L | 0.002144237 |
| 574 | 279 | Q8CI94 | Glycogen phosphorylase brain form OS=Mus musculus OX=10090 GN=Pygb PE=1 SV=3 | R.RMSVIEEGDC(+57.02)K.R | 0.003112099 |
| 1122 | 1478 | P53996 | Cellular nucleic acid-binding protein OS=Mus musculus OX=10090 GN=Cnbp PE=1 SV=2 | R.EQC(+57.02)C(+57.02)YNC(+57.02)GK(+16.03)PGHLAR.D | 0.007293643 |
| 788 | 560 | Q6R0H7 | Guanine nucleotide-binding protein G(s) subunit alpha isoforms XLas OS=Mus musculus OX=10090 GN=Gnas PE=1 SV=1 | E.EEKMDYMC(+57.02)THR.L | 0.005305881 |
| 2485 | 2458 | P97770 | THUMP domain-containing protein 3 OS=Mus musculus OX=10090 GN=Thumpd3 PE=1 SV=1 | K.TTWGLPIDAVQWDIC(+57.02)NLPLR.T | 0.002578865 |
| 1192 | 682 | P47811 | Mitogen-activated protein kinase 14 OS=Mus musculus OX=10090 GN=Mapk14 PE=1 SV=3 | R.SLEEFNDVYLVTHLMGADLNNIVK.C | 0.000948009 |
| 559 | 380 | Q9D1A2 | Cytosolic non-specific dipeptidase OS=Mus musculus OX=10090 GN=Cndp2 PE=1 SV=1 | K.QKLPDGSEIPLPPILLGK.L | 0.007201628 |
| 64 | 160 | Q8VHX6 | Filamin-C OS=Mus musculus OX=10090 GN=Flnc PE=1 SV=3 | R.DTVEVALEDKGDNTFR.C | 0.001987419 |
| 29 | 71 | P26039 | Talin-1 OS=Mus musculus OX=10090 GN=Tln1 PE=1 SV=2 | K.EVANSTANLVK(+32.06)TIK(+32.06)ALDGDFTEENR.A | 0.005660151 |
| 761 | 1114 | Q9D8S4 | Oligoribonuclease mitochondrial OS=Mus musculus OX=10090 GN=Rexo2 PE=1 SV=2 | R.QQTPPGLC(+57.02)PLAGNSVHADKK.F | 0.005851111 |
| 642 | 1197 | Q8BP92 | Reticulocalbin-2 OS=Mus musculus OX=10090 GN=Rcn2 PE=1 SV=1 | K.K(+32.06)LSEEEILENQDLFLTSEATDYGR.Q | 0.009616628 |
| 4312 | 6994 | A2AMM0 | Caveolae-associated protein 4 OS=Mus musculus OX=10090 GN=Cavin4 PE=1 SV=1 | K.QEEIMK(+32.06)KNK.F | 0.009353095 |
| 90 | 328 | P58771 | Tropomyosin alpha-1 chain OS=Mus musculus OX=10090 GN=Tpm1 PE=1 SV=1 | K.HIAEDADRK(+32.06)YEEVAR.K | 0.004328623 |

| Mutants | Deleting region (kb) | Deleting sequences |
| --- | --- | --- |
| 1 | -900 ~ -891 | AGACCAGGCA |
| 2 | -843 ~ -832 | AGGCTGGTAT |
| 3 | -795 ~ -786 | AGGCAGGCAT |
| 4 | -761 ~ -752 | GGGCACGCAG |
| 5 | -734 ~ -725 | AACCTTGTCA |
| 6 | -605 ~ -596 | AACCTTGTCA |
| 7 | -581 ~ -572 | TAGCTGGCCC |
| 8 | -555~-546 | ACACCTGCTT |

**Table S4：The mutation sequence of the P53 binding site in the promoter region of slurp1.**

**Table S5：Primer and si-RNA sequences**

| Gene name | Forward (5’ – 3’) | Reverse (5’ – 3’) |
| --- | --- | --- |
| *Slurp1*  *Il1b* | AGCCCACGGCCATTAACTC  GAAATGCCACCTTTTGACAGTG | CCAATGCCATCAGGGTCGG  TGGATGCTCTCATCAGGACAG |
| *Tnf-α* | CAGGCGGTGCCTATGTCTC | CGATCACCCCGAAGTTCAGTAG |
| *Cxcl9* | GGAGTTCGAGGAACCCTAGTG | GGGATTTGTAGTGGATCGTGC |
| *Il23* | CAGCAGCTCTCTCGGAATCTC | TGGATACGGGGCACATTATTTTT |
| *Il12a* | CAATCACGCTACCTCCTCTTTT | CAGCAGTGCAGGAATAATGTTTC |
| *Egf* | AGAGCATCTCTCGGATTGACC | CCCGTTAAGGAAAACTCTTAGCA |
| *Retnla* | CCAATCCAGCTAACTATCCCTCC | ACCCAGTAGCAGTCATCCCA |
| *Chrna 2* | ACCGCCTGTTCAAACACCTC | CCACATCTATGAGCTGTGCAA |
| *Chrna 4* | ACTCTTCTCTGGCTACAACAAGT | CCACACGTTGGTCGTCATCAT |
| si-Chrna 2 | GACCAACAUGGAUGCUGAA(dT)(dT) | UUCAGCAUCCAUGUUGGUC(dT)(dT) |
| si-Chrna 4 | GGAGACUUAUCGAAUCCAU(dT)(dT) | AUGGAUUCGAUAAGUCUCC(dT)(dT) |
| si-p53 | GAAUGAGGCCUUAGAGUUA(dT)(dT) | UAACUCUAAGGCCUCAUUC(dT)(dT) |
| si-MAPK14 | CAACCUAGCUGUGAACGAA(dT)(dT) | UUCGUUCACAGCUAGGUUG(dT)(dT) |


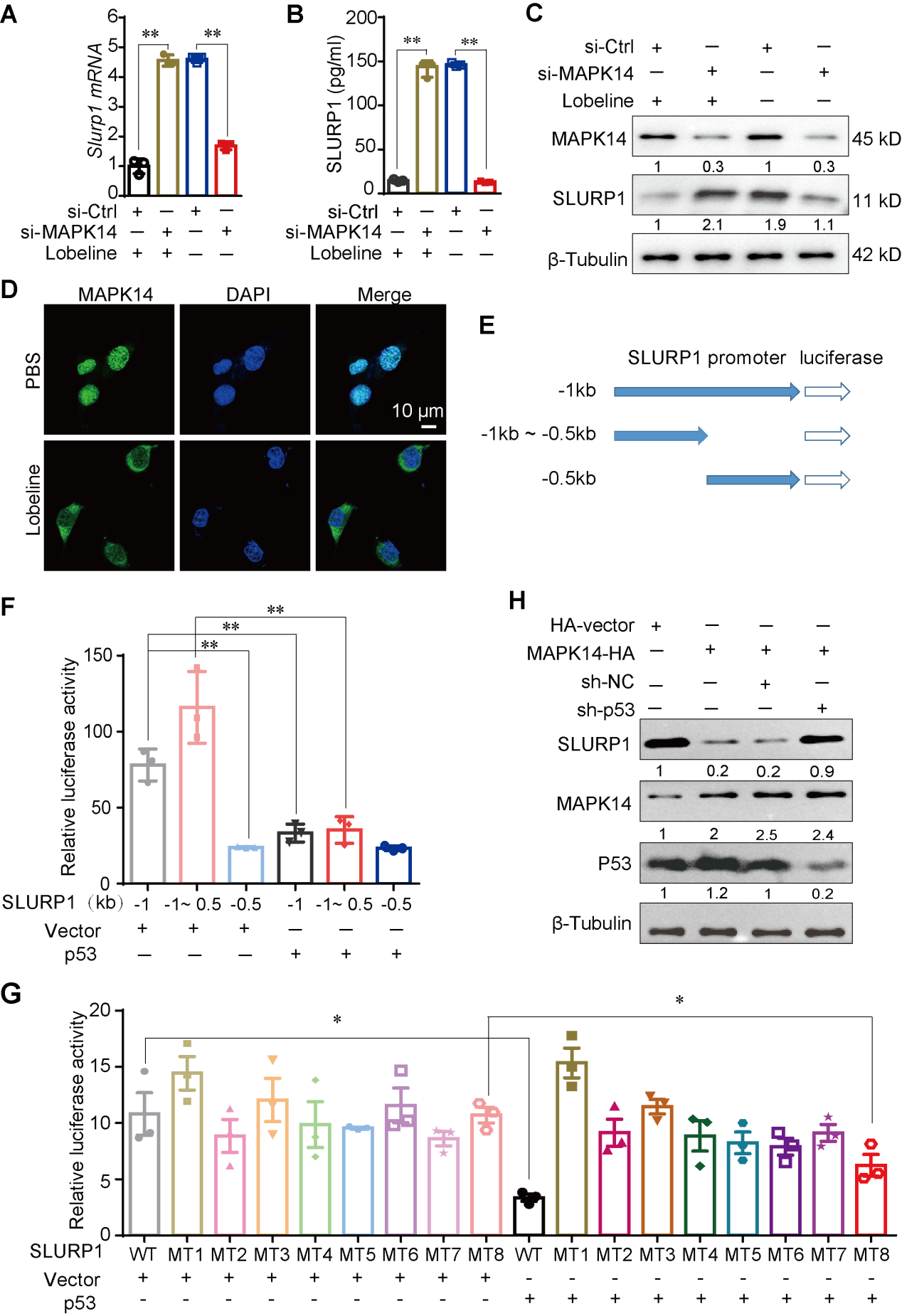

Supplement: Supplementary file 1 — Supporting Information [file ADVS-12-2407900-s001.docx]
